# Synthesis and Biological Evaluation of 3-Amino-4,4-Dimethyl Lithocholic Acid Derivatives as Novel, Selective, and Cellularly Active Allosteric SHP1 Activators

**DOI:** 10.3390/molecules28062488

**Published:** 2023-03-08

**Authors:** Huiqing Chen, Zekun Liu, Lixin Gao, Li-Fang Yu, Yubo Zhou, Jie Tang, Jia Li, Fan Yang

**Affiliations:** 1 Shanghai Engineering Research Center of Molecular Therapeutics and New Drug Development, School of Chemistry and Molecular Engineering, East China Normal University, Shanghai 200062, China; 2 National Center for Drug Screening, Shanghai Institute of Material Medica, Chinese Academy of Sciences, Shanghai 201203, China; 3 School of Pharmaceutical Science, Jiangnan University, Wuxi 214122, China; 4 Zhongshan Institute for Drug Discovery, Shanghai Institute of Materia Medica, Chinese Academy of Sciences, Shanghai 201203, China; 5 Shanghai Greenchem & Biotech Co., Ltd., Shanghai 200062, China

**Keywords:** 3-amino-4,4-dimethyl lithocholic acid derivatives, SHP1, selective activators, structure–activity relationships, anti-tumor

## Abstract

Src homology 2 domain-containing protein tyrosine phosphatase 1 (SHP1), a non-receptor member of the protein tyrosine phosphatase (PTP) family, negatively regulates several signaling pathways that are responsible for pathological cell processes in cancers. In this study, we report a series of 3-amino-4,4-dimethyl lithocholic acid derivatives as SHP1 activators. The most potent compounds, **5az-ba**, showed low micromolar activating effects (EC_50_: 1.54–2.10 μM) for SHP1, with 7.63–8.79-fold maximum activation and significant selectivity over the closest homologue Src homology 2 domain-containing protein tyrosine phosphatase 2 (SHP2) (>32-fold). **5az-ba** showed potent anti-tumor effects with IC_50_ values of 1.65–5.51 μM against leukemia and lung cancer cells. A new allosteric mechanism of SHP1 activation, whereby small molecules bind to a central allosteric pocket and stabilize the active conformation of SHP1, was proposed. The activation mechanism was consistent with the structure–activity relationship (SAR) data. This study demonstrates that 3-amino-4,4-dimethyl lithocholic acid derivatives can be selective SHP1 activators with potent cellular efficacy.

## 1. Introduction

No commercial PTP-targeted drug has been approved for decades. The biological and clinical discovery of PTP inhibitors is hampered by off-target profiles, poor pharmacokinetic properties, or the emergence of drug resistance. In contrast, SHP1 was found to negatively regulate various pathological processes, including hematopoietic lineage differentiation, tumorigenesis, and immune response, which has led to the development of SHP1 activators. SHP1 is localized mainly in the cytoplasm and is expressed on multiple cell types, including hematopoietic, epithelial, and mesenchymal cells. Low expression of SHP1 protein was observed in patients with hepatocellular carcinoma [1], prostate cancer [2], multiple sclerosis [3], and a variety of hematopoietic diseases, such as leukemia [4], lymphoma [5], and multiple myeloma [6], as well as myelodysplastic syndrome [7], whereas SHP1 is up-regulated in patients with nasopharyngeal carcinoma [8] and ovarian cancer [9].

SHP1 dephosphorylates the signal transducer and activator of transcription 3 (STAT3) [10], extracellular signal-regulated kinase (ERK) [11], protein kinase B (Akt) [12], and other signaling components [13,14,15,16], thereby contributing to a decrease in cancer cell proliferation and survival. SHP1 suppresses angiogenesis [17,18] and enhances the anti-tumor efficacy of chemotherapeutic agents [16,19]. SHP1 also inhibits inflammation [20] and osteoclastogenesis [21,22], suggesting that it has therapeutic potential for treating inflammation-mediated diseases and bone diseases. Thus, targeting SHP1 activation could represent a promising therapeutic approach for the above diseases and open a new avenue for PTP drug discovery.

**Figure 1:**
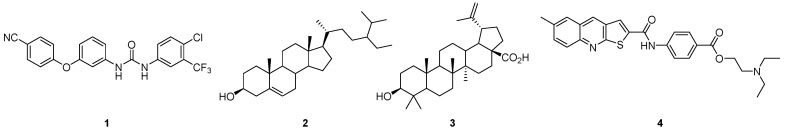
Representative compounds that have been reported to activate SHP1.

The reported molecules with SHP1-activating effects mainly include diphenyl urea [23,24,25,26], steroid [27,28,29,30,31,32], pentacyclic triterpenoid [33,34,35], and indole scaffolds [36,37,38,39,40] (Figure 1). SC-43 (**1**) enhanced SHP1 activity to inactivate STAT3, thereby inhibiting the proliferation of hepatocellular carcinoma and colorectal cancer cells [23,24]. *β*-Sitosterol (**2**) elevated SHP1 activity, suppressing the subsequent nuclear factor kappa-B (NF-κB) and signal transducer and activator of transcription 1 (STAT1) signaling pathways to exert anti-inflammatory effects [27]. SHP1 activated by betulinic acid (**3**) induced apoptosis and enhanced the cytotoxic effects of chemotherapeutic agents in multiple myeloma cells via suppressing the STAT3 signaling pathway [33]. Thieno [2,3-b]quinoline-procaine hybrid **4** can directly activate SHP1 and inhibit activated B-cell-like diffuse large B-cell lymphoma (ABC-DLBCL) cell proliferation via blocking the STAT3 pathway [41]. However, most of the molecules described above have been identified as inhibitors for other targets. There are still few studies on selective SHP1 activators, which limits the investigations of SHP1 as a pharmacological target for cancer treatment.

Lithocholic acid derivatives, which contain the above steroid scaffold, have been found to inhibit the growth of cancer cells [42,43,44,45]. Two lithocholic acid derivatives, ursodeoxycholic acid and obeticholic acid, are approved drugs for the treatment of primary biliary cholangitis. Furthermore, obeticholic acid is currently in clinical trials for nonalcoholic steatohepatitis (NASH) as a farnesoid X receptor (FXR) agonist. Here, we replaced the carboxyl group of lithocholic acid with a hydroxyl group, modified the C-3, C-7 positions to obtain a series of novel structures, and evaluated their biological activities for SHP1 activation.

## 2. Results and Discussion

**Scheme 1:**
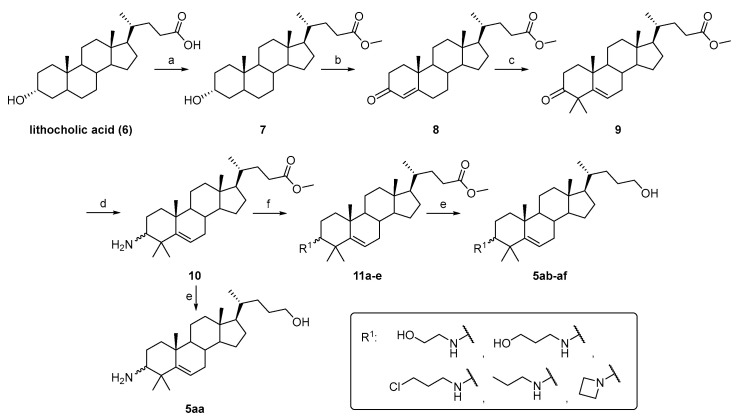
Synthesis of compounds **5aa-af**. Reagents and conditions: (a) TsOH^.^H_2_O, MeOH, reflux, 100%. (b) IBX, TFA, DMSO, 50 °C, 53%. (c) CH_3_I, KO*^t^*Bu, *^t^*BuOH, r.t.; then, TsOH^.^H_2_O, MeOH, reflux, 50%. (d) NH_4_OAc, NaBH_3_CN, NH_3_^.^H_2_O, EtOH, reflux, 75%. (e) LiAlH_4_, THF, r.t., 52–90%. (f) K_2_CO_3_, DMF, r.t. or 80 °C, 47–86%.

We initiated our efforts by investigating C-3 substituents. Usually, compounds containing the aliphatic –NH– group have better water solubility and stronger hydrogen-bonding ability. We replaced the C-3 hydroxyl group with an amino group to afford the compounds **5aa-af** (Scheme 1). Lithocholic acid (**6**) was subjected to esterification and oxidation via a combination of 2-iodoxybenzoic acid (IBX) and trifluoroacetic acid to afford *α,β*-unsaturated ketone compound **8**. Compound **9** was obtained by C-4 methylation accompanied by a rearrangement of the double bond. Reductive amination of the C-3 carbonyl group afforded compound **10**. Compound **5aa** was prepared by reducing the C-24 carboxylate group of compound **10**. The *N*-alkylation of compound **10** and the subsequent LiAlH_4_ reduction formed compounds **5ab-af**.

The SHP1-activating effects of the synthetic compounds were examined by the 6,8-difluoro-4-methylumbelliferyl phosphate (DiFMUP) assay. As shown in Table 1, the compounds **5aa-ac** bearing the C-3 hydrophilic amino or extended hydroxyl groups showed better activity. The C-3 alkylamino substitution of compounds **5ad-af** showed a decline in the activity. These results indicated that the C-3 free amino group benefits activity.

**Scheme 2:**
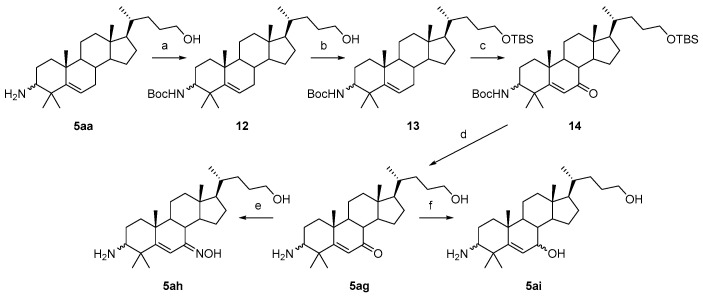
Synthesis of compounds **5ag-ai**. Reagents and conditions: (a) (Boc)_2_O, Et_3_N, DCM, r.t., 97%. (b) TBSCl, imidazole, DMF, 80 °C, 100%. (c) *N*-hydroxyphthalimide, Na_2_Cr_2_O_7_^.^2H_2_O, HOAc, acetone, 50 °C, 92%. (d) aq. HCl, THF, reflux, 94%. (e) HONH_2_^.^HCl, NaOAc, EtOH, reflux, 93%. (f) NaBH_4_, MeOH, r.t., 93%.

**Scheme 3:**
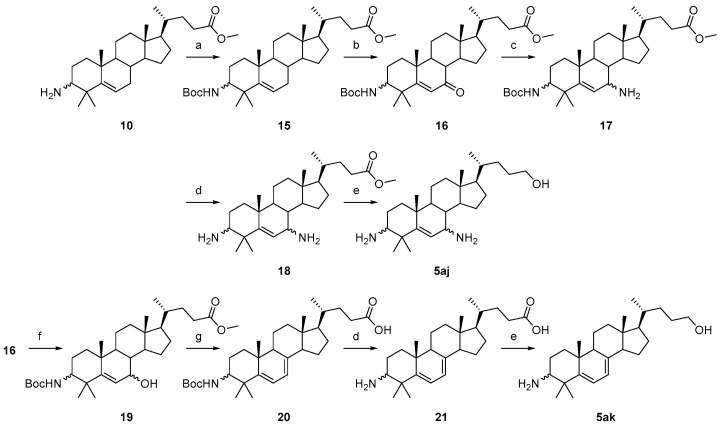
Synthesis of compounds **5aj-ak**. Reagents and conditions: (a) (Boc)_2_O, Et_3_N, DCM, r.t., 99%. (b) *N*-hydroxyphthalimide, Na_2_Cr_2_O_7_^.^2H_2_O, HOAc, acetone, 50 °C, 90%. (c) NH_4_OAc, NaBH_3_CN, NH_3_^.^H_2_O, EtOH, reflux, 74%. (d) EtOAc/HCl, 92–95%. (e) LiAlH_4_, THF, r.t., 66–73%. (f) NaBH_4_, MeOH, r.t., 92%. (g) MeI, NaH, DMF, 0 °C -r.t., 61%.

Given that C-3 free amino substitution appeared to have a better effect on potency, we next synthesized 3-amino compounds, **5ag-ak**, with different C-7 substituents (Scheme 2 and Scheme 3). Compound **5aa** underwent C-3 Boc protection and C-24 TBS protection to afford compound **13**, which then oxidized into compound **14** using sodium dichromate. Compound **5ag** was finally produced through deprotection. Compounds **5ah-ai** were prepared by C-7 oximation or the reduction of compound **5ag**. Compound **10** underwent C-3 Boc protection and C-7 oxidation to afford compound **16**. The reductive amination of the C-7 carbonyl group resulted in compound **17**. Compound **5aj** was prepared by the C-3 deprotection and C-24 reduction of compound **17**. The treatment of compound **16** with NaBH_4_ gave compound **19**. The reaction with NaH and MeI failed to obtain a 7-methoxy substituted product but afforded 5,7-diene compound **20**. Subsequent similar deprotection and reduction resulted in compound **5ak**. As shown in Table 2, the C-7 substituted compounds **5ag-aj** were significantly less potent than compound **5aa**. 5,7-Diene compound **5ak** led to only a small change in SHP1 potency compared to compound **5aa**, while the maximum activation was increased 7.74-fold.

**Scheme 4:**
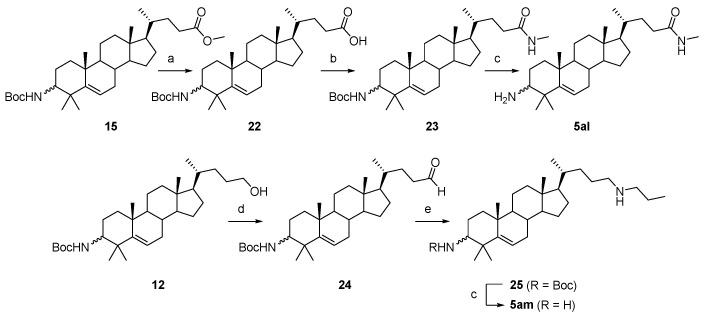
Synthesis of compounds **5al-am**. Reagents and conditions: (a) NaOH, MeOH/H_2_O, r.t., 94%. (b) HOBt, EDCI, DIPEA, DMF, r.t., 83%. (c) EtOAc/HCl, r.t., 90–93%. (d) IBX, DMSO, r.t., 62%. (e) *^n^* PrNH_2_, NaBH(OAc)_3_, DCM, r.t., 75%.

In addition, we replaced the hydroxyl group at C-24 with amide or amine to form compounds **5al-am** (Scheme 4). Compound **15** was hydrolyzed into carboxylic acid **22**, which then reacted with methylamine hydrochloride in the presence of *N*-(3-dimethylaminopropyl)-*N*’-ethylcarbodiimide hydrochloride (EDCI) to afford amide **23**. Compound **5al** was finally produced through deprotection. Compound **12** was transformed into aldehyde **24** by IBX oxidation. The subsequent reductive amination of **24** with *n*-propylamine and C-3 deprotection resulted in compound **5am**.

**Scheme 5:**
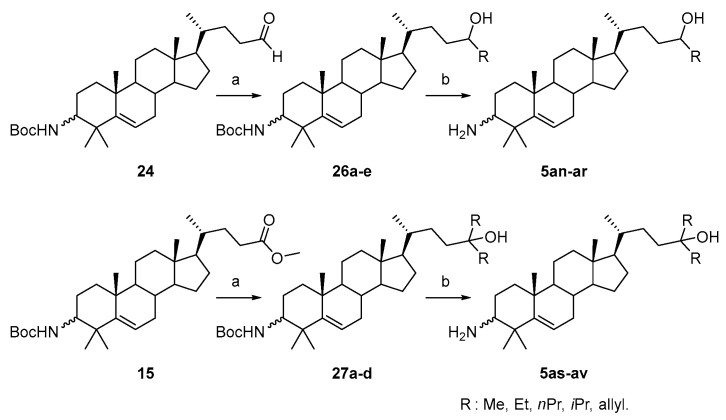
Synthesis of compounds **5an-av**. Reagents and conditions: (a) R^4^MgCl, THF, 0 °C-r.t., 45–68%. (b) EtOAc/HCl, r.t., 90–93%.

We envisioned that the introduction of secondary/tertiary alcohol groups into the side chain would probably increase the activity. The compounds **5an-av** were designed and synthesized, as described in Scheme 5. The treatment of compounds **24** and **15** with Grignard reagents, followed by C-3 deprotection, furnished compounds **5an-av**.

**Scheme 6:**
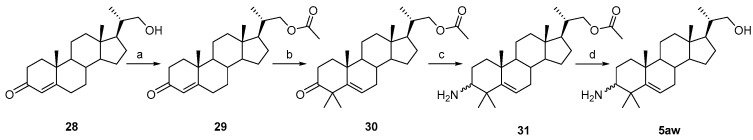
Synthesis of compound **5aw**. Reagents and conditions: (a) Ac_2_O, DMAP, DCM, r.t., 97%. (b) CH_3_I, KO*^t^*Bu, *^t^*BuOH, r.t., 67%. (c) NH_4_OAc, NaBH_3_CN, NH_3_^.^H_2_O, EtOH, reflux, 76%. (d) NaOH, MeOH/H_2_O, r.t., 90%.

To verify the influence of changing the side chain length on a compound’s biological activity, we removed two ethylene spacers between the hydroxyl group and the remainder of the scaffold to design compound **5aw** (Scheme 6). Commercially available 21-hydroxy-20-methylpregn-4-en-3- one (**28**) was initially protected with an Ac- group, methylated with KO*^t^*Bu and MeI to furnish compound **30**. The following reductive amination and C-22 deprotection resulted in compound **5aw**.

Table 3 shows that the replacement of the C-24 hydroxyl group with an amide or alkylamino moiety (compounds **5al-am**) attenuated the activity. Most of the secondary and tertiary alcohols exerted good efficacies, with EC_50_ values from 12.88 μM to 38.11 μM and 5.73- to 6.94-fold maximum activation. Ethyl substitution (compounds **5ao** and **5at**) exhibited more potent biological activity than methyl substitution (compounds **5an** and **5as**). The activation property of compound **5aw** was decreased compared to compound **5aa**. It appeared that compounds with different side chain lengths affected the activity. These data implied that the C-24 terminal position deserves further modification, and the introduction of proper groups may increase biological activity.

**Scheme 7:**
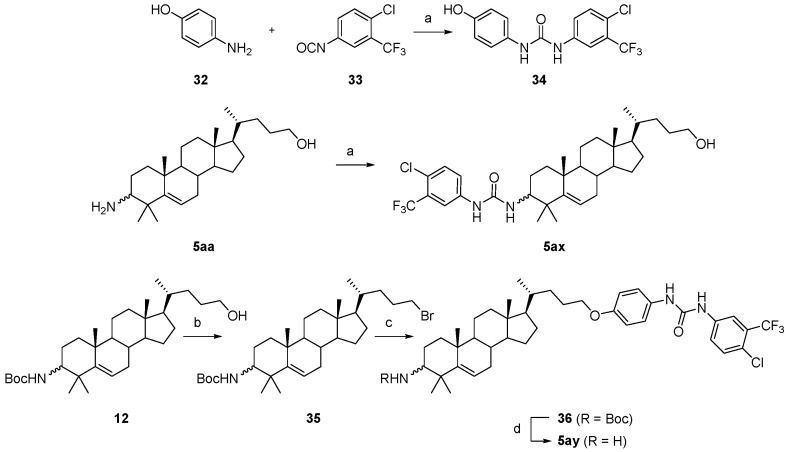
Synthesis of compounds **5ax-ay**. Reagents and conditions: (a) DCM, r.t., 96–99%. (b) CBr_4_, PPh_3_, imidazole, DMF, r.t., 76%. (c) K_2_CO_3_, DMF, 80 °C, 68%. (d) EtOAc/HCl, r.t., 94%.

**Scheme 8:**
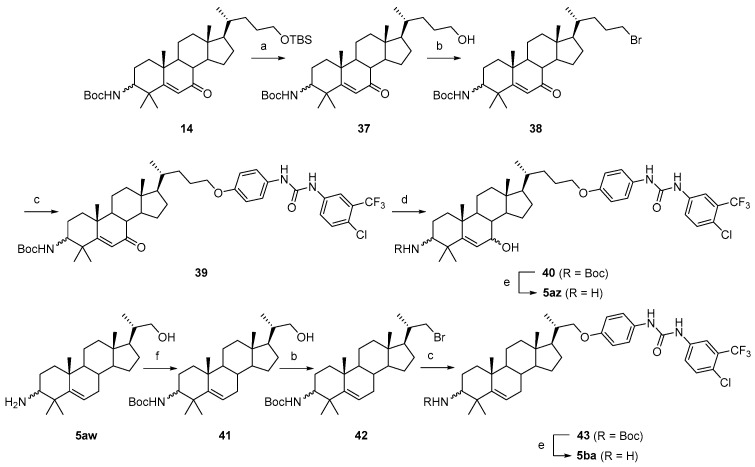
Synthesis of compounds **5az-ba**. Reagents and conditions: (a) aq. HCl, THF, r.t., 96%. (b) CBr_4_, PPh_3_, imidazole, DMF, r.t., 72–78%. (c) K_2_CO_3_, DMF, 80 °C, 77–86%. (d) NaBH_4_, MeOH, r.t., 94%. (e) EtOAc/HCl, r.t., 86–96%. (f) (Boc)_2_O, Et_3_N, DCM, r.t., 98%.

Urea exhibits unique hydrogen-binding capabilities and becomes an important functional group for drug–target interactions. We employed the rational design strategy of incorporating a diphenyl urea active substructure into the steroids to obtain the merged structures **5ax-ba**. The syntheses of compounds **5ax-ba** are outlined in Scheme 7 and Scheme 8. Treating compound **32** with isocyanate compound **33** produced diphenyl urea intermediate **34**. Compound **5ax** was made in one step from compounds **5aa** and **34** in the presence of Et_3_N. Compound **12** underwent C-24 bromination, coupling with compound **35,** and then deprotection to generate compound **5ay**.

Compound **14** was selectively deprotected and brominated with CBr_4_ and PPh_3_ to furnish compound **38**. Next, coupling with compound **34**, followed by C-7 NaBH_4_ reduction, yielded compound **40**. Finally, removal of the Boc protecting group resulted in compound **5az**. The C-3 protection and C-22 bromination of compound **5aw** gave compound **42**. The subsequent coupling and deprotection afforded compound **5ba**.

It is evident from Table 4 that the position in the steroid scaffold for the urea substituents specifies the SHP1 activity. With the placing of 4-chloro-3-(trifluoromethyl)phenyl urea in the C-3 position, the compound **5ax** showed no activity. As the diphenyl urea group was placed in the C-24 position and the other positions were kept constant, the activating effects of compounds **5ay-ba** were increased several-fold compared to those of compound **5aa**. These data support the idea that the urea group probably contacts with both the hydrophilic and the hydrophobic halves of the SHP1 active site and that a flexible side chain may enhance proper orientation. Interestingly, the 7-hydroxyl compound **5az** displayed 3-fold higher maximum activation than compound **5ay** though their EC_50_ values were similar. Compound **5ba** showed better activity than compound **5ay**, which indicates that the short length of the side chain benefits the activity. In particular, **5ba** is more than 32-fold more selective for SHP1 over the closest homologue SHP2 (EC_50_ > 50 μM). In order to ameliorate the solubility of the most potent compound, **5ba**, this class of derivatives was prepared in a hydrochloride salt form for further modifications.

The structure–activity relationships (SARs) for the 3-amino-4,4-dimethyl lithocholic acid derivatives are summarized below. The presence of a primary amine at the C-3 position (**5aa**) seems to be important for the interaction of the compound with the binding site of SHP1. The substitutions of the aliphatic amine groups at the C-3 position (**5ab-af**) exhibited lower activities, and the compound with bulky phenyl urea substitution at the C-3 position (**5ax**) was inactive, indicating that the binding site cavity of SHP1 near the C-3 position is relatively small. The compounds with substitutions at the C-7 position (**5ag-aj**) had weaker potency. The primary alcohol moiety in a side chain can be replaced by other chemical groups while maintaining or increasing potency. The compounds with diphenyl urea moieties in a side chain (**5ay-ba**) markedly increased their potency, indicating that the hydrogen bond and hydrophobic interactions play an important role in the binding pocket.

The anti-tumor effects of compounds **5az-ba** which showed higher maximum activation ability were examined by cell viability assay using acute lymphoblastic leukemia (ALL) cell line RS4;11, acute promyelocytic leukemia (APL) cell line NB4, and non-small cell lung cancer (NSCLC) cell line NCI-H1299. As shown in Table 5, **5az-ba** exhibited significant cytotoxicity to effectively inhibit cancer cell viability; this was particularly the case with compound **5az** against RS4;11 cells, with an IC_50_ value of less than 2 μM.

**Figure 2:**
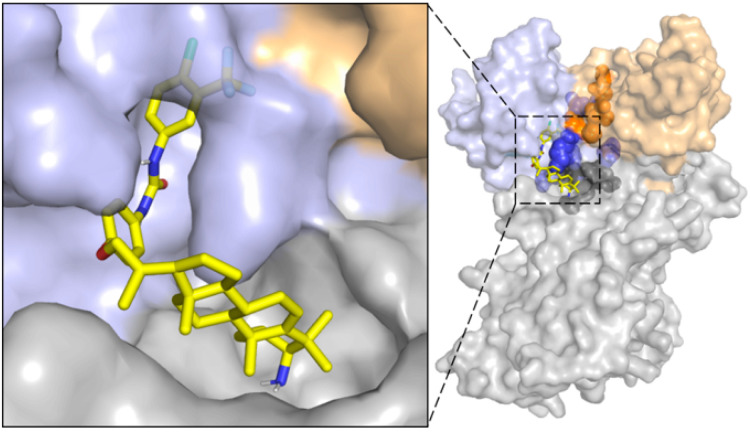
Proposed binding mode of **5ba** and SHP1 in open conformation. Reported residues on the interfaces to stabilize active SHP1 are shown as spheres. Compound is shown in yellow. N-SH2, C-SH2, and PTP domains are shown in blue, orange, and gray, respectively.

Based on these activity data, we selected the crystal structure of SHP1 in the open conformation (PDB ID: 3PS5) and the 3-β-diastereomer of **5ba** for the docking study (see Supplementary Materials). Compound **5ba** binds at the central allosteric site through a network of hydrogen bonds and hydrophobic interactions (Figure 2). More importantly, this site is closest to the reported interface of three domains [46], and the binding of compounds tightens the inter-domain connection, which thereby stabilizes the active conformation of SHP1.

**Figure 3:**
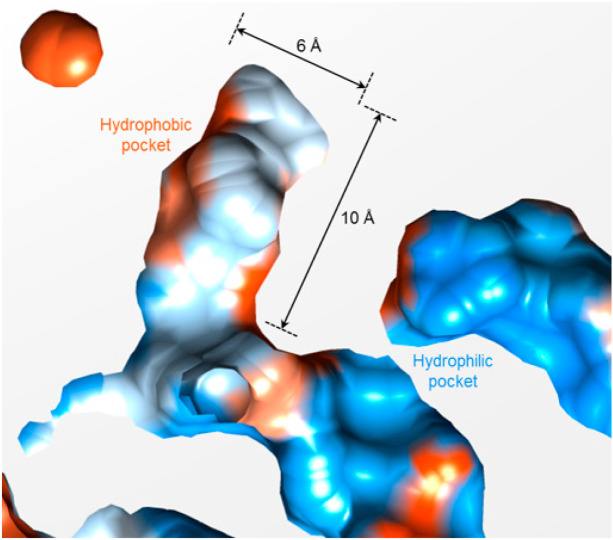
Hydrophobic and geometric properties of the binding pocket. Hydrophilic and hydrophobic regions are shown in blue and orange, respectively.

**Figure 4:**
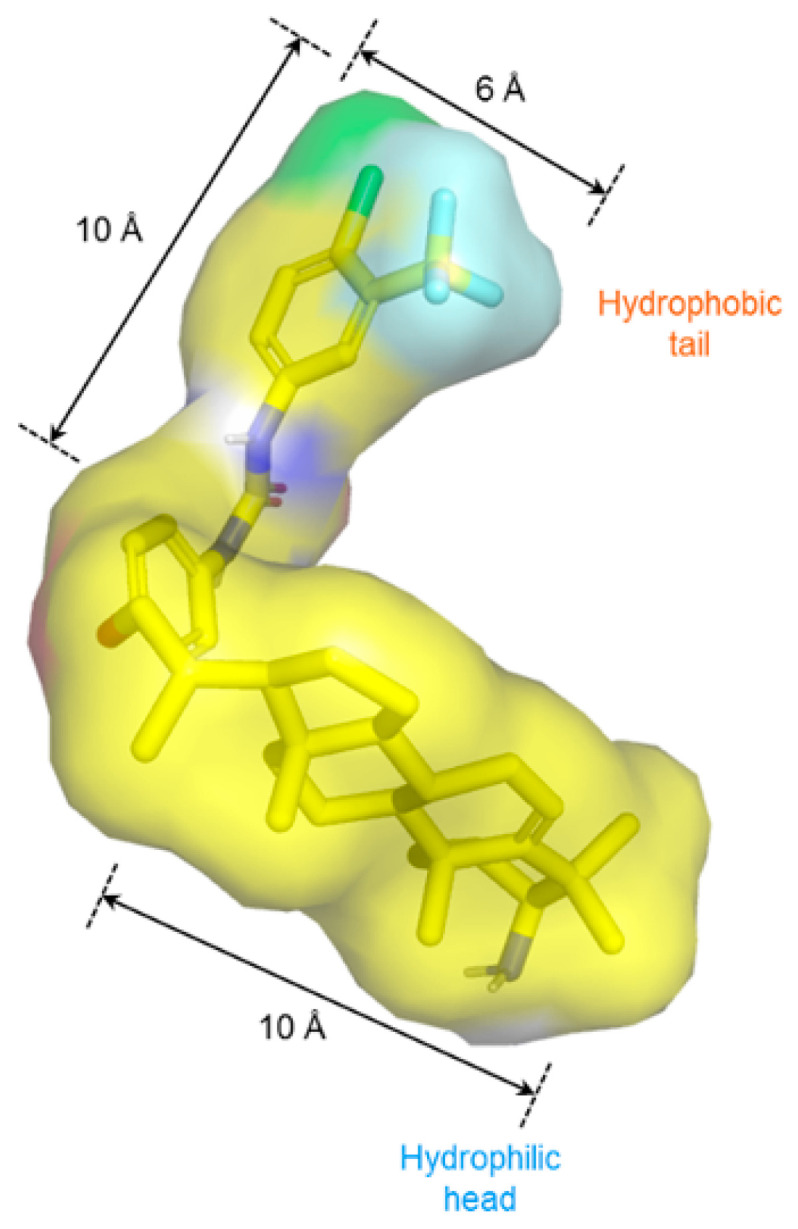
Geometric property of compound **5ba**.

The binding pocket is mainly composed of a hydrophobic pocket and a hydrophilic pocket (Figure 3). The narrow hydrophobic pocket is made up of Val2, Trp4, Phe27, and Val76, with Met1 as gatekeeper. The depth of the hydrophobic pocket is approximately 10 Å, and the width is approximately 6 Å. Four hydrophilic residues, namely Arg217, Asp481, Asp483, and Lys486, embrace the larger hydrophilic pocket from one side. The hydrophilic pocket provides the interaction between the protein and the amino head of the compound. Compound **5ba** possesses an L-shaped structure, and the phenyl urea tail fits well in the hydrophobic pocket (Figure 4).

**Figure 5:**
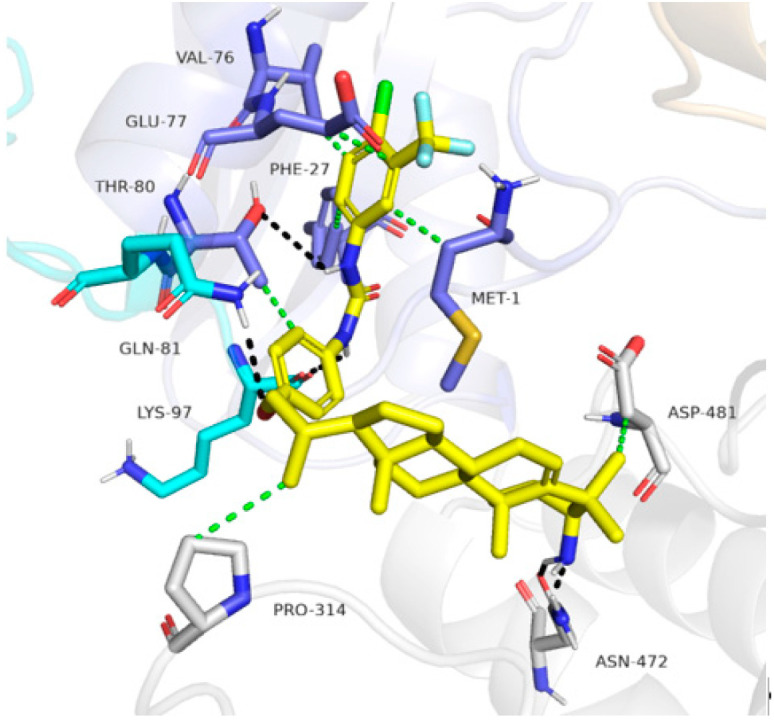
Predicted interactions of **5ba** and SHP1 in open conformation. Adjacent amino acids of N-SH2 and PTP domains are shown blue and gray, respectively. BG loop is shown in cyan. Black dashed lines indicate hydrogen bond interactions. Green dashed lines indicate hydrophobic interactions.

As shown in Figure 5, the 4-chloro-3-(trifluoromethyl)phenyl ring occupies the hydrophobic pocket through hydrophobic interactions with Met1, Phe27, Val76, and Glu77. The urea group of **5ba** forms two hydrogen bonds with Thr80 (3.7 Å) and Lys97 (2.9 Å), which could explain why the potency of **5ba** increased ∼23 times than **5aw**. The phenoxy group forms a hydrogen bond with Gln81 (3.1 Å) and forms a hydrophobic interaction with Thr80. The 3-amino group of the steroid parent nucleus creates two hydrogen bonds with Asn472 (1.9, 2.9 Å), which accounts for the potency of the series of newly synthesized compounds. The C-4 methyl group forms a hydrophobic interaction with Asp481. The C-21 methyl group forms a hydrophobic interaction with Pro314.

**Figure 6:**
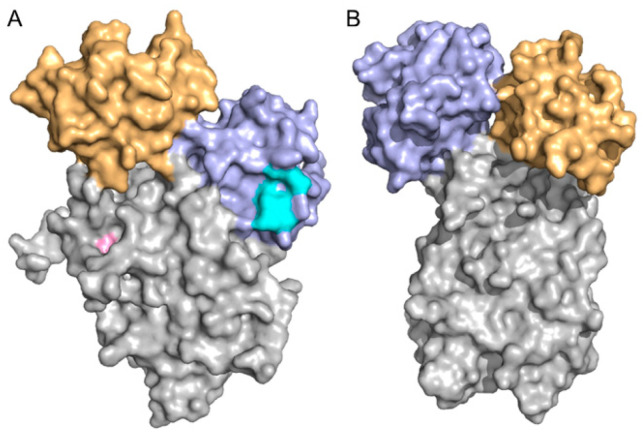
(**A**) SHP1 in auto-inhibitory conformation (PDB ID: 2B3O). Thr80, Gln81, and Lys97 are shown in cyan. Asn472 is shown in pink. (**B**) SHP2 in open conformation (PDB ID: 6CRF). N-SH2, C-SH2, and PTP domains are shown in blue, orange and gray, respectively.

Structure modification at the C-3 position (**5ab-af**) dramatically attenuated the potency, suggesting that -NH_2_ is the key group for activity retention. This could also be explained by the reduced hydrogen bond interactions of the amino group with Asn472. Those compounds lacking a urea group only occupy and bind the entrance of the pocket with less interactions, leading to reduced potency compared to **5ay-ba**. The three methyl groups at C-4 and C-19 incur steric hindrance to the urea 3-NH of compound **5ax**, making it energetically unfavorable to engage Asn472, which is consistent with the loss of potency. As shown in Figure 6A, Thr80, Gln81, and Lys97 are on the surface of the auto-inhibited SHP1, which allows the free access and binding of compounds. The long and flexible BG loop plays a pivotal role in the conformational change and activation of SH2 domain-containing proteins [46,47]. The positively charged head group of the compound may interact with the negatively charged LQDRDG motif on the BG loop of the N-SH2 domain, which causes movement of the BG loop. Then, the “switch” N-SH2 domain shifts away, moving from one side of the PTP domain to the other, leading to open conformation and thus the activation of SHP1. In contrast, no similar pockets were observed in the center of the homologue SHP2 in open conformation (Figure 6B). These observations probably explain the selectivity of **5ba** for SHP1 over SHP2.

## 3. Materials and Methods

### 3.1. Chemical Synthesis

All reagents and solvents were obtained from commercial suppliers and used without further purification unless otherwise indicated. Melting points (mps) were taken in open capillaries on a WRS-2 melting point system. The ^1^H NMR and ^13^C NMR spectra were recorded using TMS as the internal standard on a Bruker Ascend 400 spectrometer at 400 and 100 MHz, respectively. The ^1^H NMR chemical shifts were reported in parts per million (ppm) relative to the centerline of the singlet signal of the solvent molecule (7.26 ppm for CDCl_3_ or 3.31 ppm for CD_3_OD). The ^13^C NMR chemical shifts were reported in ppm relative to the centerline at 77.16 ppm for CDCl_3_ or at 49.00 ppm for CD_3_OD. The data were reported as follows: chemical shift, multiplicity (s = singlet, d = doublet, t = triplet, dd = doublet of doublets, m = multiplet), coupling constant (Hz), and integration. Evaporation was carried out in vacuo using a rotating evaporator. Silica gel chromatography was performed using silica gel (200–300 mesh). Reactions were monitored by TLC with detection by phosphomolybdic acid visualization or UV light (λ = 254 nm). All reactions involving air- or moisture-sensitive reagents were performed under a nitrogen atmosphere using anhydrous solvents. The purity of the final compounds was >95%, as deduced by ^1^H NMR spectra. High-resolution mass spectroscopy (HRMS) was performed on a time-of-flight instrument with electrospray ionization (ESI) in the positive ionization mode.

#### 3.1.1. Methyl (4R)-4-((3R,10S,13R,17R)-3-hydroxy-10,13-dimethylhexadecahydro-1H-cyclopenta[a]phenanthren-17-yl)pentanoate (**7**)

TsOH^.^H_2_O (47.5 mg, 0.25 mmol) was added to a stirred solution of lithocholic acid **6** (1.88 g, 5 mmol) in MeOH (20 mL). The mixture was refluxed for 2 h, and TLC indicated the consumption of the starting material. MeOH was removed in vacuo, and aqueous NaHCO_3_ solution was added. The mixture was extracted with EtOAc (3 × 30 mL). The combined organic layer was washed with brine, dried over Na_2_SO_4_, filtered, and concentrated to afford compound **7** (1.95 g, 100% yield) as a white solid. The mp was 105.3–106.5 °C. ^1^H-NMR (400 MHz, CDCl_3_) *δ* 3.65 (s, 3H), 3.62–3.59 (m, 1H), 2.38–2.30 (m, 1H), 2.24–2.17 (m, 1H), 1.96–1.93 (m, 1H), 1.87–0.96 (m, other aliphatic ring protons), 0.90 (s, 3H), 0.89 (d, *J* = 6.0 Hz, 3H), 0.63 (s, 3H). HRMS (ESI): calcd for C_25_H_42_NaO_3_ [M + Na]^+^ 413.3026; found 413.3037.

#### 3.1.2. Methyl(4R)-4-((10R,13R,17R)-10,13-dimethyl-3-oxo-tetradecahydro-1H-cyclopenta[a]phenanthren-17-yl)pentanoate (**8**)

IBX (3.5 g, 12.5 mmol), TFA (0.2 mL) and a solution of compound **7** (1.95 g, 5 mmol) in DMSO (20 mL) were combined. The mixture was warmed to 50 °C, and all the solids were dissolved. After stirring for 2 h under a N_2_ atmosphere, the reaction was completed, as indicated by TLC. The reaction mixture was cooled and added to an aqueous NaHSO_3_ solution (20 mL). The mixture was extracted with EtOAc (3 × 30 mL). The combined organic layer was washed with water, brine, dried over Na_2_SO_4_, filtered, and concentrated. The crude material was purified by silica gel column chromatography using PE/EtOAc (10:1, *v*/*v*) to afford compound **8** (1.02 g, 53% yield) as a white solid. The mp was 124.2–126.1 °C. ^1^H-NMR (400 MHz, CDCl_3_) *δ* 5.71 (s, 1H), 3.65 (s, 3H), 2.45–2.30 (m, 4H), 2.27–2.17 (m, 2H), 2.02–1.98 (m, 2H), 1.85–0.99 (m, other aliphatic ring protons), 0.90 (d, *J* = 6.4 Hz, 3H), 0.70 (s, 3H). HRMS (ESI): calcd for C_25_H_38_NaO_3_ [M + Na]^+^ 409.2713; found 409.2732.

#### 3.1.3. Methyl(4R)-4-((10R,13R,17R)-4,4,10,13-tetramethyl-3-oxo-tetradecahydro-1H-cyclopenta[a]phenanthren-17-yl)pentanoate (**9**)

Potassium *tert*-butoxide (1.18 g, 10.56 mmol) was added in batches to the suspension of compound **8** (1.02 g, 2.64 mmol) in absolute *tert*-butyl alcohol (50 mL). The mixture was stirred at r.t. for 30 min to form a clear yellow solution and then added to MeI (1.5 g, 10.56 mmol) dropwise. The reaction was stirred at r.t. for 12 h in the dark under a N_2_ atmosphere, and the starting material disappeared, as monitored by TLC. Upon addition of an aqueous Na_2_S_2_O_3_ solution (5 mL) for quenching the excess MeI, the mixture was evaporated and water (30 mL) was added. The suspension was acidified to pH < 6 with an aqueous HCl solution. The mixture was extracted with EtOAc (3 × 30 mL). The combined organic layer was washed with brine, dried over Na_2_SO_4_, filtered, and concentrated. The crude material was dissolved in MeOH (20 mL), and TsOH^.^H_2_O (47.5 mg, 0.25 mmol) was added. The mixture was refluxed for 2 h, and the MeOH was removed in vacuo. An aqueous NaHCO_3_ solution was added, and it was extracted with EtOAc (3 × 30 mL). The combined organic layer was washed with brine, dried over Na_2_SO_4_, filtered, concentrated, and purified by silica gel column chromatography using PE/EtOAc (10:1, *v*/*v*) to obtain compound **9** (547 mg, 50% yield) as a white solid. The mp was 133.6–135.8 °C. ^1^H-NMR (400 MHz, CDCl_3_) *δ* 5.55–5.53 (m, 1H), 3.66 (s, 3H), 2.59–2.43 (m, 2H), 2.39–2.31 (m, 1H), 2.26–2.18 (m, 1H), 2.13–2.06 (m, 1H), 2.02–1.97 (m, 2H), 1.90–1.76 (m, 2H), 1.68–1.02 (m, other aliphatic ring protons), 0.91 (d, *J* = 6.4 Hz, 3H), 0.84 (s, 3H), 0.68 (s, 3H). HRMS (ESI): calcd for C_27_H_42_NaO_3_ [M+Na]^+^ 437.3026; found 437.3059.

#### 3.1.4. Methyl(4R)-4-((10R,13R,17R)-3-amino-4,4,10,13-tetramethyl-tetradecahydro-1H-cyclopenta[a]phenanthren-17-yl)pentanoate (**10**)

Compound **9** (547 mg, 1.32 mmol) was dissolved in a saturated NH_4_OAc/EtOH solution (20 mL). It was added to NaBH_3_CN (166 mg, 2.64 mmol) and NH_3_^.^H_2_O (0.8 mL). The mixture was refluxed for 12 h under a N_2_ atmosphere, and TLC indicated the consumption of the starting material. The solvents were removed in vacuo and water (20 mL) was added. The mixture was extracted with EtOAc (3 × 30 mL). The combined organic layer was washed with brine, dried over Na_2_SO_4_, filtered, concentrated, and purified by silica gel column chromatography using DCM/MeOH/NH_3_^.^H_2_O (100:3:0.5, *v*/*v*/*v*) to obtain compound **10** (412 mg, 75% yield) as a white solid. The mp was 145.0–147.3 °C. ^1^H-NMR (400 MHz, CDCl_3_) *δ* 5.55–5.51 (m, 1H), 3.65 (s, 3H), 2.38–2.31 (m, 2H), 2.25–2.17 (m, 1H), 2.11–2.04 (m, 1H), 1.99–1.96 (m, 1H), 1.86–1.77 (m, 2H), 1.73–1.69 (m, 1H), 1.63–0.95 (m, other aliphatic ring protons), 0.91 (d, *J* = 6.4 Hz, 3H), 0.66 (s, 3H).

#### 3.1.5. (4R)-4-((10R,13R,17R)-3-amino-4,4,10,13-tetramethyl-tetradecahydro-1H-cyclopenta[a]phenanthren-17-yl)pentan-1-ol (**5aa**)

LiAlH_4_ (11 mg, 0.3 mmol) was added in batches at 0 °C to a dry THF solution (20 mL) of compound **10** (116 mg, 0.28 mmol). The mixture was stirred at r.t. for 12 h under a N_2_ atmosphere. Water (11 μL) was added to quench the reaction, then a 15% NaOH solution (11 μL) and then water (33 μL). The suspension was filtrated, and the solid residue was washed with THF (10 mL). The solvents were removed in vacuo, and water (30 mL) was added to the residue. The mixture was extracted with DCM (3 × 30 mL). The combined organic layer was washed with brine, dried over Na_2_SO_4_, filtered, concentrated, and purified by silica gel column chromatography using DCM/MeOH/NH_3_^.^H_2_O (100:5:0.5, *v*/*v*/*v*) to obtain compound **5aa** (81 mg, 75% yield) as a white solid. The mp was 163.5–166.3 °C. ^1^H-NMR (400 MHz, CDCl_3_) *δ* 5.55–5.52 (m, 1H), 3.59–3.57 (m, 2H), 2.37–2.33 (m, 1H), 2.10–2.05 (m, 1H), 2.00–1.97 (m, 1H), 1.87–1.80 (m, 1H), 1.73–1.70 (m, 1H), 1.65–0.89 (m, other aliphatic ring protons), 0.66 (s, 3H). ^13^C-NMR (100 MHz, CDCl_3_) *δ* 150.52, 119.58, 63.52, 58.37, 57.43, 56.02, 51.22, 42.33, 41.01, 39.91, 37.79, 37.04, 35.71, 32.80, 32.05, 30.93, 29.54, 28.43, 28.35, 27.73, 24.29, 23.76, 21.38, 20.65, 18.81, 12.00. HRMS (ESI) calcd for C_26_H_46_NO [M+H]^+^ 388.3501; found 388.3585.

#### 3.1.6. Methyl(4R)-4-((10R,13R,17R)-3-((2-hydroxyethyl)amino)-4,4,10,13-tetramethyl-tetradecahydro-1H-cyclopenta[a]phenanthren-17-yl)pentanoate (**11a**)

Potassium carbonate (166 mg, 1.2 mmol) and 2-bromoethanol (82 mg, 0.66 mmol) were added to a solution of compound **10** (249 mg, 0.6 mmol) in DMF (10 mL), and the mixture was heated to 80 °C and stirred for 2 h under a N_2_ atmosphere. When the reaction was completed, as indicated by TLC, water (30 mL) was added to the residue. The mixture was filtered, concentrated, and purified by silica gel column chromatography using DCM/MeOH/NH_3_^.^H_2_O (100:5:0.5, *v*/*v*/*v*) to obtain compound **11a** (168 mg, 61% yield) as a white solid. The mp was 165.4–166.8 °C. ^1^H-NMR (400 MHz, CDCl_3_) *δ* 5.56–5.54 (m, 1H), 3.65 (s, 3H), 3.62–3.51 (m, 2H), 3.00–2.95 (m, 1H), 2.67–2.61 (m, 1H), 2.38–2.31 (m, 1H), 2.24–0.87 (m, other aliphatic ring protons), 0.65 (s, 3H). HRMS (ESI) calcd for C_29_H_50_NO_3_ [M + H]^+^ 460.3785; found 460.3795.

#### 3.1.7. Methyl(4R)-4-((10R,13R,17R)-3-((3-hydroxypropyl)amino)-4,4,10,13-tetramethyl-tetradecahydro-1H-cyclopenta[a]phenanthren-17-yl)pentanoate (**11b**)

Compound **11b** was synthesized in a 63% yield as a white solid using a similar procedure to that in 3.1.6. ^1^H-NMR (400 MHz, CDCl_3_) *δ* 5.56–5.54 (m, 1H), 3.82 (t, *J* = 5.2 Hz, 2H), 3.65 (s, 3H), 3.59–3.47 (m, 1H), 3.18–3.13 (m, 1H), 2.69–2.63 (m, 1H), 2.38–2.30 (m, 2H), 2.25–2.17 (m, 2H), 2.10–0.87 (m, other aliphatic ring protons), 0.66 (s, 3H). HRMS (ESI): calcd for C_30_H_52_NO_3_ [M + H]^+^ 474.3942; found 474.3973.

#### 3.1.8. Methyl(4R)-4-((10R,13R,17R)-3-((3-chloropropyl)amino)-4,4,10,13-tetramethyl-tetradecahydro-1H-cyclopenta[a]phenanthren-17-yl)pentanoate (**11c**)

Compound **11c** was synthesized in a 47% yield as a white solid using a similar procedure to that in 3.1.6, and the reaction temperature was r.t. The mp was 119.7–121.3 °C. ^1^H-NMR (400 MHz, CDCl_3_) *δ* 5.56–5.53 (m, 1H), 3.65 (s, 3H), 3.63 (t, *J* = 6.4 Hz, 2H), 2.95–2.88 (m, 2H), 2.61–2.56 (m, 1H), 2.39–2.31 (m, 1H), 2.25–2.17 (m, 1H), 2.11–2.04 (m, 1H), 1.99–0.87 (m, other aliphatic ring protons), 0.66 (s, 3H). HRMS (ESI): calcd for C_30_H_51_ClNO_2_ [M + H]^+^ 492.3603; found 492.3646.

#### 3.1.9. Methyl(4R)-4-((10R,13R,17R)-4,4,10,13-tetramethyl-3-(propylamino)-tetradecahydro-1H-cyclopenta[a]phenanthren-17-yl)pentanoate (**11d**)

Compound **11d** was synthesized in an 86% yield as a white solid using a similar procedure to that in 3.1.6. ^1^H-NMR (400 MHz, CDCl_3_) *δ* 5.57–5.53 (m, 1H), 3.66 (s, 3H), 2.83–2.76 (m, 1H), 2.54–2.48 (m, 1H), 2.39–2.31 (m, 1H), 2.25–2.20 (m, 1H), 2.11–2.05 (m, 2H), 2.00–1.97 (m, 1H), 1.88–0.89 (m, other aliphatic ring protons), 0.66 (s, 3H). HRMS (ESI): calcd for C_30_H_52_NO_2_ [M+H]^+^ 458.3993; found 458.4004.

#### 3.1.10. Methyl(4R)-4-((10R,13R,17R)-3-(azetidin-1-yl)-4,4,10,13-tetramethyl-tetradecahydro-1H-cyclopenta[a]phenanthren-17-yl)pentanoate (**11e**)

Compound **11e** was synthesized in a 73% yield as a white solid using a similar procedure to that in 3.1.6. The mp was 173.7–175.5 °C. ^1^H-NMR (400 MHz, CDCl_3_) *δ* 5.56–5.54 (m, 1H), 3.65 (s, 3H), 3.60–3.63 (m, 4H), 2.38–2.31 (m, 1H), 2.25–0.84 (m, other aliphatic ring protons), 0.65 (s, 3H). HRMS (ESI): calcd for C_30_H_50_NO_2_ [M + H]^+^ 456.3836; found 456.3853.

#### 3.1.11. (4R)-4-((10R,13R,17R)-3-((2-hydroxyethyl)amino)-4,4,10,13-tetramethyl-tetradecahydro-1H-cyclopenta[a]phenanthren-17-yl)pentan-1-ol (**5ab**)

Compound **5ab** was synthesized in a 68% yield as a white solid using a similar procedure to that in 3.1.5. The mp was 184.3–187.5 °C. ^1^H-NMR (400 MHz, CDCl_3_:CD_3_OD = 3:1) *δ* 5.54–5.52 (m, 1H), 3.66–3.56 (m, 2H), 3.52–3.48 (m, 2H), 2.87–2.81 (m, 1H), 2.60–2.54 (m, 1H), 2.09–2.01 (m, 2H), 2.00–1.96 (m, 1H), 1.83–1.78 (m, 1H), 1.77–1.71 (m, 2H), 1.62–0.84 (m, other aliphatic ring protons), 0.64 (s, 3H). ^13^C-NMR (100 MHz, CDCl_3_:CD_3_OD = 3:1) *δ* 150.56, 119.88, 65.41, 63.12, 60.80, 57.54, 56.19, 51.27, 50.24, 42.42, 40.73, 40.00, 37.38, 37.11, 35.87, 32.82, 32.13, 31.03, 29.35, 28.44, 27.52, 24.89, 24.36, 24.33, 21.40, 20.78, 18.76, 12.00. HRMS (ESI): calcd for C_28_H_50_NO_2_ [M + H]^+^ 432.3836; found 432.3840.

#### 3.1.12. (4R)-4-((10R,13R,17R)-3-((3-hydroxypropyl)amino)-4,4,10,13-tetramethyl-tetradecahydro-1H-cyclopenta[a]phenanthren-17-yl)pentan-1-ol (**5ac**)

Compound **5ac** was synthesized in a 62% yield as a white solid using a similar procedure to that in 3.1.5. The mp was 183.5–186.8 °C. ^1^H-NMR (400 MHz, CDCl_3_) *δ* 5.57–5.55 (m, 1H), 3.82 (t, *J* = 5.2 Hz, 2H), 3.60 (t, *J* = 6.4 Hz, 2H), 3.18–3.13 (m, 1H), 2.69–2.63 (m, 1H), 2.11–2.06 (m, 2H), 2.02–1.98 (m, 1H), 1.88–0.87 (m, other aliphatic ring protons), 0.66 (s, 3H). ^13^C-NMR (100 MHz, CDCl_3_) *δ* 150.46, 119.85, 65.74, 64.88, 63.70, 57.44, 56.03, 51.15, 49.39, 42.35, 40.72, 39.91, 37.51, 36.98, 35.71, 32.76, 31.99, 31.36, 30.94, 29.53, 28.42, 27.81, 24.98, 24.27, 21.35, 20.71, 18.81, 12.02. HRMS (ESI): calcd for C_29_H_51_NNaO_2_ [M + Na]^+^ 468.3812; found 468.3805.

#### 3.1.13. (4R)-4-((10R,13R,17R)-3-((3-chloropropyl)amino)-4,4,10,13-tetramethyl-tetradecahydro-1H-cyclopenta[a]phenanthren-17-yl)pentan-1-ol (**5ad**)

Compound **5ad** was synthesized in a 79% yield as a white solid using a similar procedure to that in 3.1.5. The mp was 162.7–165.5 °C. ^1^H-NMR (400 MHz, CDCl_3_) *δ* 5.56–5.52 (m, 1H), 3.66–3.60 (m, 4H), 2.96–2.92 (m, 1H), 2.64–2.59 (m, 1H), 2.10–0.91 (m, other aliphatic ring protons), 0.66 (s, 3H). ^13^C-NMR (100 MHz, CDCl_3_) *δ* 150.98, 119.55, 64.98, 63.70, 57.45, 56.02, 51.17, 45.44, 43.42, 42.35, 41.02, 39.92, 37.70, 36.99, 35.71, 33.29, 32.76, 31.98, 30.97, 29.52, 28.42, 27.61, 24.90, 24.81, 24.27, 21.40, 20.70, 18.80, 12.02. HRMS (ESI): calcd for C_29_H_51_ClNO [M + H]^+^ 464.3654; found 464.3676.

#### 3.1.14. (4R)-4-((10R,13R,17R)-4,4,10,13-tetramethyl-3-(propylamino)-tetradecahydro-1H-cyclopenta[a]phenanthren-17-yl)pentan-1-ol (**5ae**)

Compound **5ae** was synthesized in a 90% yield as a white solid using a similar procedure to that in 3.1.5. The mp was 147.5–150.4 °C. ^1^H-NMR (400 MHz, CDCl_3_) *δ* 5.56–5.53 (m, 1H), 3.62–3.59 (m, 2H), 2.82–2.76 (m, 1H), 2.54–2.47 (m, 1H), 2.10–2.05 (m, 2H), 2.01–1.98 (m, 1H), 1.84–1.80 (m, 2H), 1.79–1.74 (m, 1H), 1.62–0.87 (m, other aliphatic ring protons), 0.66 (s, 3H). ^13^C-NMR (100 MHz, CDCl_3_) *δ* 150.71, 119.74, 65.25, 63.68, 57.45, 56.03, 51.16, 50.50, 42.35, 40.77, 39.92, 37.53, 37.00, 35.71, 32.77, 31.99, 30.94, 29.53, 28.42, 27.58, 24.99, 24.32, 24.27, 22.82, 21.39, 20.70, 18.81, 12.02, 11.89. HRMS (ESI): calcd for C_29_H_52_NO [M + H]^+^ 430.4043; found 430.4068.

#### 3.1.15. (4R)-4-((10R,13R,17R)-3-(azetidin-1-yl)-4,4,10,13-tetramethyl-tetradecahydro-1H-cyclopenta[a]phenanthren-17-yl)pentan-1-ol (**5af**)

Compound **5af** was synthesized in a 77% yield as a white solid using a similar procedure to that in 3.1.5. The mp was 174.9–177.6 °C. ^1^H-NMR (400 MHz, CDCl_3_) *δ* 5.53–5.51 (m, 1H), 3.60 (t, *J* = 6.4 Hz, 2H), 3.37 (dd, *J* = 11.2, 4.4 Hz, 2H), 3.27 (dd, *J* = 11.2, 4.4 Hz, 2H), 2.09–1.97 (m, 4H), 1.87–1.84 (m, 2H), 1.78–1.75 (m, 1H), 1.64–0.86 (m, other aliphatic ring protons), 0.65 (s, 3H). ^13^C-NMR (100 MHz, CDCl_3_) *δ* 150.70, 119.22, 72.83, 63.70, 57.46, 56.02, 55.32 (2C), 51.30, 42.32, 41.75, 39.92, 37.68, 37.02, 35.70, 32.87, 31.99, 30.90, 29.53, 28.92, 28.42, 26.06, 24.26, 21.38, 20.70, 20.08, 18.81, 17.99, 12.01. HRMS (ESI): calcd for C_29_H_50_NO [M + H]^+^ 428.3887; found 428.3866.

#### 3.1.16. Tert-butyl((10R,13R,17R)-17-((R)-5-hydroxypentan-2-yl)-4,4,10,13-tetramethyl-tetradecahydro-1H-cyclopenta[a]phenanthren-3-yl)carbamate (**12**)

Boc_2_O (524 mg, 2.4 mmol) and Et_3_N (404 mg, 4 mmol) were added to a solution of compound **5aa** (776 mg, 2 mmol) in dry DCM (30 mL), and the reaction was stirred at r.t. for 2 h; TLC indicated the consumption of the starting material. Water (30 mL) was added, and the mixture was extracted with DCM (3 × 30 mL). The combined organic layer was washed with aqueous NaOH solution, brine, dried over Na_2_SO_4_, filtered, concentrated, and recrystallized in 5 mL of EtOAc to obtain compound **12** (945 mg, 97% yield) as a white solid. ^1^H-NMR (400 MHz, CDCl_3_) *δ* 5.58–5.55 (m, 1H), 4.44 (d, *J* = 10.0 Hz, 1H), 3.61 (t, *J* = 6.0 Hz, 2H), 3.32–3.27 (m, 1H), 2.09–2.04 (m, 1H), 2.00–1.97 (m, 1H), 1.86–1.79 (m, 1H), 1.73–0.92 (m, other aliphatic ring protons), 0.92 (d, *J* = 6.0 Hz, 3H), 0.66 (s, 3H).

#### 3.1.17. Tert-butyl((10R,13R,17R)-17-((R)-5-((tert-butyldimethylsilyl)oxy)pentan-2-yl)-4,4,10,13-tetramethyl-tetradecahydro-1H-cyclopenta[a]phenanthren-3-yl)carbamate (**13**)

TBSCl (226 mg, 1.5 mmol) was added to a solution of compound **12** (366 mg, 0.75 mmol) and imidazole (204 mg, 3 mmol) in dry DMF (8 mL). The reaction mixture was stirred at 80 °C for 6 h under a N_2_ atmosphere, and the starting material disappeared, as monitored by TLC. The mixture was added with an aqueous NaOH solution (20 mL) and extracted with EtOAc (3 × 30 mL). The combined organic layer was washed with water, brine, dried over Na_2_SO_4_, filtered, concentrated, and recrystallized in 5 mL of EtOAc to obtain compound **13** (451 mg, 100% yield) as a white solid. The mp was 183.4–185.1 °C. ^1^H-NMR (400 MHz, CD_3_OD) *δ* 5.56–5.54 (m, 1H), 4.43 (d, *J* = 10.0 Hz, 1H), 3.56 (t, *J* = 6.4 Hz, 2H), 3.33–3.27 (m, 1H), 2.09–1.97 (m, 2H), 1.85–1.78 (m, 1H), 1.73–0.86 (m, other aliphatic ring protons), 0.66 (s, 3H), 0.04 (s, 6H).

#### 3.1.18. Tert-butyl((10R,13R,17R)-17-((R)-5-((tert-butyldimethylsilyl)oxy)pentan-2-yl)-4,4,10,13-tetramethyl-7-oxo-tetradecahydro-1H-cyclopenta[a]phenanthren-3-yl)carbamate (**14**)

Na_2_Cr_2_O_7_^.^2H_2_O (243 mg, 0.81 mmol) was added to a solution of compound **13** (406 mg, 0.74 mmol), *N*-hydroxyphthalimide (241 mg, 1.48 mmol), and HOAc (0.2 mL) in acetone (30 mL). The reaction was heated to 50 °C and stirred for 12 h under a N_2_ atmosphere, and then, another quantity of Na_2_Cr_2_O_7_^.^2H_2_O (110 mg, 0.37 mmol) was added. The reaction was continued for 6 h. When the reaction was completed, as indicated by TLC, the mixture was concentrated and purified by silica gel column chromatography using PE/EtOAc (5:1, *v*/*v*) to obtain compound **14** (419 mg, 92% yield) as a white solid. mp 190.5–192.6 °C. ^1^H-NMR (400 MHz, CD_3_OD) *δ* 5.95 (s, 1H), 4.48 (d, *J* = 10.0 Hz, 1H), 3.56 (t, *J* = 6.4 Hz, 2H), 3.49–3.44 (m, 1H), 2.31–2.29 (m, 1H), 2.22–2.17 (m, 1H), 2.03–1.01 (m, other aliphatic ring protons), 0.91 (d, *J* = 6.0 Hz, 3H), 0.88 (s, 9H), 0.67 (s, 3H), 0.04 (s, 6H). HRMS (ESI): calcd for C_37_H_65_NNaO_4_Si [M + Na]^+^ 638.4575; found 638.4576.

#### 3.1.19. (10R,13R,17R)-3-amino-17-((R)-5-hydroxypentan-2-yl)-4,4,10,13-tetramethyl-tetradecahydro-7H-cyclopenta[a]phenanthren-7-one (**5ag**)

An aqueous 4N HCl solution (5 mL) was added to the solution of compound **14** (167 mg, 0.32 mmol) in THF (5 mL). The reaction was refluxed for 2 h, and TLC indicated the consumption of the starting material. The suspension was basified to pH > 8 with aqueous NaOH solution. The solvent THF was removed in vacuo, and water (30 mL) was added. The mixture was extracted with DCM (3 × 30 mL). The combined organic layer was washed with brine, dried over Na_2_SO_4_, filtered, and concentrated to obtain compound **5ag** (121 mg, 94% yield) as a white solid. The mp was 151.3–153.7 °C. ^1^H-NMR (400 MHz, CDCl_3_) *δ* 5.93 (s, 1H), 3.57 (t, *J* = 6.4 Hz, 2H), 2.53–2.49 (m, 1H), 2.30–2.26 (m, 1H), 2.22–2.17 (m, 1H), 2.02–1.99 (m, 1H), 1.88–1.85 (m, 1H), 1.80–1.76 (m, 1H), 1.66–1.02 (m, other aliphatic ring protons), 0.91 (d, *J* = 6.0 Hz, 3H), 0.67 (s, 3H). ^13^C-NMR (100 MHz, CDCl_3_) *δ* 203.23, 176.64, 124.91, 63.48, 57.21, 54.82, 52.00, 50.80, 45.20, 43.40, 42.10, 38.95, 38.77, 36.54, 35.67, 32.00, 29.52, 28.67, 27.88, 26.66, 26.48, 23.20, 20.91, 19.59, 18.92, 12.04. HRMS (ESI): calcd for C_26_H_44_NO_2_ [M + H]^+^ 402.3367; found 402.3372.

#### 3.1.20. (10R,13R,17R)-3-amino-17-((R)-5-hydroxypentan-2-yl)-4,4,10,13-tetramethyl-tetradecahydro-7H-cyclopenta[a]phenanthren-7-oxime (**5ah**)

NH_2_OH^.^HCl (35 mg, 0.5 mmol) and NaOAc (136 mg, 1 mmol) was added to a solution of compound **5ag** (40 mg, 0.1 mmol) in EtOH (5 mL). The mixture was refluxed for 2 h. When the reaction was completed, as indicated by TLC, the solvent EtOH was removed in vacuo, and an aqueous NaOH solution (10 mL) was added. The mixture was extracted with DCM (3 × 30 mL). The combined organic layer was washed with brine, dried over Na_2_SO_4_, filtered, concentrated, and recrystallized in MeCN to obtain compound **5ah** (39 mg, 93% yield) as a white solid. The mp was 218.3–219.9 °C. ^1^H-NMR (400 MHz, CDCl_3_:CD_3_OD = 10:1) *δ* 6.86 (s, 1H), 3.53 (t, *J* = 6.4 Hz, 2H), 2.46–2.42 (m, 1H), 2.31–2.27 (m, 1H), 2.21–2.18 (m, 1H), 2.00–1.97 (m, 1H), 1.80–0.82 (m, other aliphatic ring protons), 0.66 (s, 3H). ^13^C-NMR (100 MHz, CDCl_3_:CD_3_OD = 10:1) *δ* 163.70, 157.64, 110.97, 63.30, 57.53, 54.83, 51.89, 51.67, 43.17, 41.96, 38.97, 38.57, 37.50, 36.79, 35.59, 31.94, 29.31, 28.42, 27.59, 27.32, 26.95, 23.38, 20.68, 19.91, 18.90, 12.25. HRMS (ESI): calcd for C_26_H_45_N_2_O_2_ [M + H]^+^ 417.3476; found 417.3486.

#### 3.1.21. (10R,13R,17R)-3-amino-17-((R)-5-hydroxypentan-2-yl)-4,4,10,13-tetramethyl-tetradecahydro-1H-cyclopenta[a]phenanthren-7-ol (**5ai**)

NaBH_4_ (19 mg, 0.5 mmol) was added to the solution of compound **5ag** (40 mg, 0.1 mmol) in MeOH (10 mL). The mixture was stirred at r.t. for 2 h under a N_2_ atmosphere. When the reaction was completed, as indicated by TLC, HOAc (0.1 mL) was added, and the solvent was removed in vacuo. An aqueous NaOH solution (10 mL) was added, and the mixture was extracted with DCM (3 × 30 mL). The combined organic layer was washed with brine, dried over Na_2_SO_4_, filtered, concentrated, and recrystallized in EtOAc to obtain compound **5ai** (37 mg, 73% yield) as a white solid. The mp was 171.4–173.2 °C. ^1^H-NMR (400 MHz, CDCl_3_) *δ* 5.52–5.48 (m, 1H), 3.81–3.79 (m, 1H), 3.51 (t, *J* = 6.4 Hz, 2H), 2.40–2.37 (m, 1H), 2.06–2.03 (m, 1H), 1.88–1.02 (m, other aliphatic ring protons), 0.97 (d, *J* = 5.6 Hz, 3H), 0.73 (s, 3H). ^13^C-NMR (100 MHz, CDCl_3_) *δ* 153.20, 123.49, 73.77, 63.02, 57.80, 56.31, 55.37, 49.98, 42.63, 40.46, 39.84, 39.47, 37.09, 36.96, 35.57, 31.90, 29.16, 28.46, 27.28 (2C), 25.98, 23.23, 21.08, 20.63, 18.63, 11.71. HRMS (ESI): calcd for C_26_H_46_NO_2_ [M + H]^+^ 404.3523; found 404.3548.

#### 3.1.22. Methyl(4R)-4-((10R,13R,17R)-3-((tert-butoxycarbonyl)amino)-4,4,10,13-tetramethyl-tetradecahydro-1H-cyclopenta[a]phenanthren-17-yl)pentanoate (**15**)

Compound **15** was synthesized in a 99% yield as a white solid using a similar procedure to that in 3.1.16. The mp was 190.6–191.8 °C. ^1^H-NMR (400 MHz, CDCl_3_) *δ* 5.56–5.53 (m, 1H), 4.43 (d, *J* = 10.0 Hz, 1H), 3.65 (s, 3H), 3.32–3.27 (m, 1H), 2.39–2.31 (m, 1H), 2.25–2.17 (m, 1H), 2.09–2.04 (m, 1H), 1.88–1.96 (m, 1H), 1.85–0.95 (m, other aliphatic ring protons), 0.91 (d, *J* = 6.0 Hz, 3H), 0.66 (s, 3H). HRMS (ESI): calcd for C_32_H_53_NNaO_4_ [M + Na]^+^ 538.3867; found 538.3880.

#### 3.1.23. Methyl(4R)-4-((10R,13R,17R)-3-((tert-butoxycarbonyl)amino)-4,4,10,13-tetramethyl-7-oxo-tetradecahydro-1H-cyclopenta[a]phenanthren-17-yl)pentanoate (**16**)

Compound **16** was synthesized in a 90% yield as a white solid using a similar procedure to that in 3.1.18. ^1^H-NMR (400 MHz, CDCl_3_) *δ* 5.95 (s, 1H), 4.48 (d, *J* = 9.6 Hz, 1H), 3.65 (s, 3H), 3.49–3.44 (m, 1H), 2.38–2.30 (m, 2H), 2.25–2.17 (m, 2H), 2.02–1.99 (m, 1H), 1.91–1.89 (m, 1H), 1.81–1.05 (m, other aliphatic ring protons), 0.91 (d, *J* = 6.4 Hz, 3H), 0.68 (s, 3H). HRMS (ESI): calcd for C_32_H_52_NO_5_ [M + H]^+^ 530.3840; found 530.3835.

#### 3.1.24. Methyl(4R)-4-((10R,13R,17R)-7-amino-3-((tert-butoxycarbonyl)amino)-4,4,10,13-tetramethyl-tetradecahydro-1H-cyclopenta[a]phenanthren-17-yl)pentanoate (**17**)

Compound **17** was synthesized in a 74% yield as a white solid using a similar procedure to that in 3.1.4. and was used for the next reaction without further purification.

#### 3.1.25. Methyl(4R)-4-((10R,13R,17R)-3,7-diamino-4,4,10,13-tetramethyl-tetradecahydro-1H-cyclopenta[a]phenanthren-17-yl)pentanoate (**18**)

Compound **17** (160 mg, 0.3 mmol) was dissolved in an anhydrous EtOAc/HCl solution (10 mL). The mixture was stirred at r.t. for 4 h. Water (20 mL) was added, and the suspension was basified to pH > 8 with an aqueous NaOH solution. The mixture was extracted with EtOAc (3 × 30 mL). The combined organic layer was washed with brine, dried over Na_2_SO_4_, filtered, and concentrated to obtain compound **18** (118 mg, 92% yield) as a white solid. ^1^H-NMR (400 MHz, CDCl_3_) *δ* 5.42–5.40 (m, 1H), 3.65 (s, 3H), 3.03 (dd, *J* = 8.0, 2.8 Hz, 1H), 2.40–2.32 (m, 2H), 2.25–2.19 (m, 1H), 1.99–0.98 (m, other aliphatic ring protons), 0.91 (d, *J* = 6.0 Hz, 3H), 0.68 (s, 3H). HRMS (ESI): calcd for C_27_H_46_N_2_NaO_2_ [M + Na]^+^ 453.3451; found 453.3447.

#### 3.1.26. (4R)-4-((10R,13R,17R)-3,7-diamino-4,4,10,13-tetramethyl-tetradeca hydro-1H-cyclopenta[a]phenanthren-17-yl)pentan-1-ol (**5aj**)

Compound **5aj** was synthesized in a 66% yield as a white solid using a similar procedure to that in 3.1.5. The mp was 178.7–180.2 °C. ^1^H-NMR (400 MHz, CDCl_3_) *δ* 5.40–5.35 (m, 1H), 3.55 (t, *J* = 4.0 Hz, 2H), 3.03–3.00 (m, 1H), 2.38–2.34 (m, 1H), 1.99–1.96 (m, 1H), 1.89–1.82 (m, 1H), 1.77–0.95 (m, other aliphatic ring protons), 0.91 (d, *J* = 6.0 Hz, 3H), 0.66 (s, 3H). ^13^C-NMR (100 MHz, CDCl_3_) *δ* 152.40, 124.81, 63.21, 58.04, 56.93, 55.37, 54.59, 51.07, 43.07, 41.74, 40.76, 39.79, 37.45, 36.80, 35.66, 32.08, 29.55, 28.66, 28.19, 27.39, 26.41, 23.68, 21.82, 20.89, 18.82, 12.02.

#### 3.1.27. Methyl(4R)-4-((10R,13R,17R)-3-((tert-butoxycarbonyl)amino)-7-hydroxy-4,4,10,13-tetramethyl-tetradecahydro-1H-cyclopenta[a]phenanthren-17-yl)pentanoate (**19**)

Compound **19** was synthesized in a 92% yield as a white solid using a similar procedure to that in 3.1.21. The mp was 198.3–200.4 °C. ^1^H-NMR (400 MHz, CDCl_3_) *δ* 5.51 (d, *J* = 2.0 Hz, 1H), 4.45 (d, *J* = 10.0 Hz, 1H), 3.91–3.89 (m, 1H), 3.65 (s, 3H), 3.35–3.29 (m, 1H), 2.38–2.31 (m, 1H), 2.25–2.17 (m, 1H), 1.99–1.00 (m, other aliphatic ring protons), 0.91 (d, *J* = 6.0 Hz, 3H), 0.67 (s, 3H). HRMS (ESI): calcd for C_32_H_53_NNaO_5_ [M + Na]^+^ 554.3816; found 554.3831.

#### 3.1.28. (4R)-4-((10R,13R,17R)-3-((tert-butoxycarbonyl)amino)-4,4,10,13-tetramethyl-dodecahydro-1H-cyclopenta[a]phenanthren-17-yl)pentanoic acid (**20**)

Sixty percent NaH (30 mg, 0.75 mmol) and MeI (107 mg, 0.75 mmol) were added to a solution of compound **19** (133 mg, 0.25 mmol) in dry DMF (10 mL). The mixture was stirred at r.t. for 2 h under a N_2_ atmosphere, and the starting material disappeared. An aqueous Na_2_S_2_O_3_ solution (5 mL) was added to quench the excess MeI. Water (30 mL) was added, and the mixture was extracted with EtOAc (3 × 30 mL). The combined organic layer was washed with water, brine, dried over Na_2_SO_4_, filtered, concentrated, and recrystallized in 5 mL of EtOH to obtain compound **20** (76 mg, 61% yield) as a white solid and used for the next reaction without further purification.

#### 3.1.29. (4R)-4-((10R,13R,17R)-3-amino-4,4,10,13-tetramethyl-2,3,4,9,10,11,12,13,14,15,16,17-dodecahydro-1H-cyclopenta[a]phenanthren-17-yl)pentanoic acid (**21**)

Compound **21** was synthesized in a 92% yield as white solid using a similar procedure to that in 3.1.25 and used for the next reaction without further purification.

#### 3.1.30. (4R)-4-((10R,13R,17R)-3-amino-4,4,10,13-tetramethyl-2,3,4,9,10,11,12,13,14,15,16,17-dodecahydro-1H-cyclopenta[a]phenanthren-17-yl)pentan-1-ol (**5ak**)

Compound **5ak** was synthesized in a 92% yield as a white solid using a similar procedure to that in 3.1.5. The mp was 179.5–181.3 °C. ^1^H-NMR (400 MHz, CDCl_3_) *δ* 6.21 (d, *J* = 10.0 Hz, 1H), 5.63 (d, *J* = 10.0 Hz, 1H), 3.61 (t, *J* = 6.4 Hz, 2H),2.47–2.36 (m, 2H), 2.33–2.25 (m, 1H), 2.00–1.97 (m, 1H), 1.89–0.95 (m, other aliphatic ring protons), 0.88 (s, 3H), 0.76 (s, 3H), 0.65 (s, 3H). ^13^C-NMR (100 MHz, CDCl_3_) *δ* 146.41, 126.76, 126.66, 125.27, 63.61, 59.86, 55.99, 55.90, 50.35, 43.72, 38.10, 37.15, 36.74, 36.16, 34.74, 31.76, 29.45, 28.78, 28.21, 27.53, 24.94, 19.54, 19.28, 19.00, 16.08, 13.13. HRMS (ESI): calcd for C_26_H_44_NO [M + H]^+^ 386.3417; found 386.3429.

#### 3.1.31. (4R)-4-((10R,13R,17R)-3-((tert-butoxycarbonyl)amino)-4,4,10,13-tetramethyl-tetradecahydro-1H-cyclopenta[a]phenanthren-17-yl)pentanoic acid (**22**)

An aqueous 2N NaOH solution (1 mL) was added to the solution of compound **15** (516 mg, 1.0 mmol) in MeOH (5 mL). The reaction was stirred at r.t. for 2 h, and TLC indicated the consumption of the starting material. Water (20 mL) was added, and the suspension was acidified to pH < 6 with aqueous HCl solution. The solvent MeOH was removed in vacuo. The mixture was extracted with DCM (3 × 30 mL). The combined organic layer was washed with brine, dried over Na_2_SO_4_, filtered, and concentrated to obtain compound **22** (470 mg, 94% yield) as a white solid. The mp was 243.1–245.5 °C. ^1^H-NMR (400 MHz, CDCl_3_) *δ* 5.57–5.52 (m, 1H), 4.45 (d, *J* = 10.0 Hz, 1H), 3.33–3.27 (m, 1H), 2.43–2.35 (m, 1H), 2.29–2.21 (m, 1H), 2.09–2.05 (m, 1H), 2.00–1.97 (m, 1H), 1.89–1.77 (m, 2H), 1.73–0.92 (m, other aliphatic ring protons), 0.67 (s, 3H). HRMS (ESI): calcd for C_31_H_51_NNaO_4_ [M + Na]^+^ 524.3710; found 524.3726.

#### 3.1.32. Tert-butyl((10R,13R,17R)-4,4,10,13-tetramethyl-17-((R)-5-(methylamino)-5-oxopentan-2-yl)-tetradecahydro-1H-cyclopenta[a]phenanthren-3-yl)carbamate (**23**)

MeNH_2_^.^HCl (27 mg, 0.4 mmol), HOBt (54 mg, 0.4 mmol), EDCI (77 mg, 0.4 mmol), and DIPEA (52 mg, 0.4 mmol) were added to a solution of compound **22** (100 mg, 0.2 mmol) in DMF (10 mL). The reaction mixture was stirred at r.t. for 12 h under a N_2_ atmosphere, and the starting material disappeared, as monitored by TLC. Water (30 mL) was added, and the mixture was extracted with EtOAc (3 × 30 mL). The combined organic layer was washed with water, brine, dried over Na_2_SO_4_, filtered, concentrated, and purified by silica gel column chromatography using DCM/MeOH (30:1, *v*/*v*) to obtain compound **23** (85 mg, 83% yield) as a white solid. The mp was 228.8–230.2 °C. ^1^H-NMR (400 MHz, CDCl_3_) *δ* 5.60–5.58 (m, 1H), 5.54–5.51 (m, 1H), 4.44 (d, *J* = 10.0 Hz, 1H), 3.30–3.25 (m, 1H), 2.78 (d, *J* = 4.4 Hz, 3H), 2.26–2.19 (m, 1H), 2.08–2.00 (m, 2H), 1.98–1.95 (m, 1H), 1.87–0.95 (m, other aliphatic ring protons), 0.91 (d, *J* = 6.4 Hz, 3H), 0.65 (s, 3H). HRMS (ESI): calcd for C_32_H_54_N_2_NaO_3_ [M + Na]^+^ 537.4027; found 537.4039.

#### 3.1.33. (4R)-4-((10R,13R,17R)-3-amino-4,4,10,13-tetramethyl-tetradecahydro-1H-cyclopenta[a]phenanthren-17-yl)-N-methylpentanamide (**5al**)

Compound **5al** was synthesized in a 93% yield as a white solid using a similar procedure to that in 3.1.25. The mp was >300 °C. ^1^H-NMR (400 MHz, CD_3_OD) *δ* 5.69–5.65 (m, 1H), 3.28–3.26 (m, 2H), 2.86–2.83 (m, 1H), 2.66 (s, 3H), 2.19–0.97 (m, other aliphatic ring protons), 0.92 (d, *J* = 6.4 Hz, 3H), 0.68 (s, 3H). ^13^C-NMR (100 MHz, CD_3_OD) *δ* 177.31, 148.94, 122.93, 60.46, 58.55, 57.15, 52.23, 43.39, 40.95, 39.86, 37.84, 37.55, 36.87, 33.99, 33.60, 33.31, 32.01, 29.20, 27.79, 26.33, 25.13, 25.03, 24.58, 21.64, 21.63, 18.86, 12.32. HRMS (ESI): calcd for C_27_H_47_N_2_O [M + H]^+^ 415.3683; found 415.3668.

#### 3.1.34. Tert-butyl((10R,13R,17R)-4,4,10,13-tetramethyl-17-((R)-5-oxopentan-2-yl)-tetradecahydro-1H-cyclopenta[a]phenanthren-3-yl)carbamate (**24**)

IBX (616 mg, 2.2 mmol) was added to a solution of compound **12** (976 mg, 2 mmol) in DMSO (10 mL). The mixture was stirred at r.t. for 2 h under a N_2_ atmosphere. When the reaction was completed, as indicated by TLC, the reaction mixture was added to an aqueous NaHSO_3_ solution (20 mL). The mixture was extracted with EtOAc (3 × 30 mL). The combined organic layer was washed with water, brine, dried over Na_2_SO_4_, filtered, and concentrated under reduced pressure. The crude material was purified by silica gel column chromatography using PE/EtOAc (10:1, *v*/*v*) to obtain compound **24** (602 mg, 62% yield) as a white solid. The mp was 173.3–174.8 °C. ^1^H-NMR (400 MHz, CD_3_OD) *δ* 9.75 (s, 1H), 5.56–5.53 (m, 1H), 4.43 (d, *J* = 9.6 Hz, 1H), 3.31–3.2 (m, 1H), 2.47–2.40 (m, 1H), 2.38–2.29 (m, 1H), 2.09–2.04 (m, 1H), 1.99–1.96 (m, 1H), 1.90–0.95 (m, other aliphatic ring protons), 0.91 (d, *J* = 6.4 Hz, 3H), 0.66 (s, 3H). HRMS (ESI): calcd for C_31_H_51_NNaO_3_ [M + Na]^+^ 508.3761; found 508.3770.

#### 3.1.35. Tert-butyl((10R,13R,17R)-4,4,10,13-tetramethyl-17-((R)-5-(propylamino)pentan-2-yl)-tetradecahydro-1H-cyclopenta[a]phenanthren-3-yl)carbamate (**25**)

NaBH(OAc)_3_ (85 mg, 0.4 mmol) at 0 °C was added to a solution of compound **24** (92 mg, 0.2 mmol) and *n*-propylamine (18 mg, 0.3 mmol) in DCM (10 mL). The mixture was stirred at r.t. for 2 h under a N_2_ atmosphere, and the starting material disappeared. Water (30 mL) was added, and the mixture was extracted with DCM (3 × 30 mL). The combined organic layer was washed with brine, dried over Na_2_SO_4_, filtered, concentrated, and purified by silica gel column chromatography using DCM/MeOH/NH_3_^.^H_2_O (100:5:0.5, *v*/*v*/*v*) to obtain compound **25** (80 mg, 75% yield) as a white solid. ^1^H-NMR (400 MHz, CD_3_OD) *δ* 5.55–5.51 (m, 1H), 4.43 (d, *J* = 9.6 Hz, 1H), 3.30–3.27 (m, 1H), 2.55 (t, *J* = 7.2 Hz, 4H), 2.08–2.1.97 (m, 2H), 1.87–1.78 (m, 1H), 1.72–0.88 (m, other aliphatic ring protons), 0.65 (s, 3H). HRMS (ESI): calcd for C_34_H_61_N_2_O_2_ [M + H]^+^ 529.4728; found 529.4728.

#### 3.1.36. (10R,13R,17R)-4,4,10,13-tetramethyl-17-((R)-5-(propylamino)pentan-2-yl)-tetradecahydro-1H-cyclopenta[a]phenanthren-3-amine (**5am**)

The compound **5am** was synthesized in a 90% yield as a white solid using a similar procedure to that in 3.1.25. mp >300 °C. ^1^H-NMR (400 MHz, CD_3_OD) *δ* 5.63–5.57 (m, 1H), 2.66 (t, *J* = 7.2 Hz, 4H), 2.42–2.39 (m, 1H), 2.13–2.08 (m, 1H), 2.07–2.04 (m, 1H), 1.91–1.84 (m, 1H), 1.80–1.77 (m, 1H), 1.69–0.94 (m, other aliphatic ring protons), 0.72 (s, 3H). ^13^C-NMR (100 MHz, CD_3_OD) *δ* 151.19, 121.08, 59.59, 58.71, 57.29, 52.58, 51.96, 50.68, 43.40, 41.50, 41.11, 38.66, 38.01, 36.99, 34.49, 33.76, 32.15, 29.33, 28.05, 27.70, 26.05, 25.19, 24.46, 22.60, 21.77, 21.66, 19.16, 12.35, 11.80. HRMS (ESI): calcd for C_29_H_53_N_2_ [M + H]^+^ 429.4203; found 429.4237.

#### 3.1.37. Tert-butyl((10R,13R,17R)-17-((2R)-5-hydroxyhexan-2-yl)-4,4,10,13-tetramethyl-tetradecahydro-1H-cyclopenta[a]phenanthren-3-yl)carbamate (**26a**)

A 1M MeMgCl solution in THF (1.1 mL, 1.1 mmol) was added dropwise at 0 °C to a dry THF solution (20 mL) of compound **24** (243 mg, 0.5 mmol) under a N_2_ atmosphere. The mixture was stirred at r.t. for 12 h under a N_2_ atmosphere. When the reaction was completed, as indicated by TLC, an aqueous NH_4_Cl solution (2 mL) was added, and the solvents were removed in vacuo. Water (30 mL) was added, and the mixture was extracted with DCM (3 × 30 mL). The combined organic layer was washed with brine, dried over Na_2_SO_4_, filtered, concentrated, and purified by silica gel column chromatography using DCM/MeOH (30:1, *v*/*v*) to obtain compound **26a** (130 mg, 52% yield) as a white solid. The mp was 200.8–202.5 °C. ^1^H-NMR (400 MHz, CDCl_3_) *δ* 5.57–5.55 (m, 1H), 4.43 (d, *J* = 10.0 Hz, 1H), 3.77–3.71 (m, 1H), 3.33–3.27 (m, 1H), 2.10–2.04 (m, 1H), 2.00–1.97 (m, 1H), 1.88–1.81 (m, 1H), 1.73–0.83 (m, other aliphatic ring protons), 0.66 (s, 3H). HRMS (ESI): calcd for C_32_H_55_NNaO_3_ [M + Na]^+^ 524.4074; found 524.4083.

#### 3.1.38. Tert-butyl((10R,13R,17R)-17-((2R)-5-hydroxyheptan-2-yl)-4,4,10,13-tetramethyl-tetradecahydro-1H-cyclopenta[a]phenanthren-3-yl)carbamate (**26b**)

Compound **26b** was synthesized in a 57% yield as a white solid using a similar procedure to that in 3.1.37. The mp was 187.3–188.8 °C. ^1^H-NMR (400 MHz, CDCl_3_) *δ* 5.47–5.45 (m, 1H), 4.45 (d, *J* = 9.2 Hz, 1H), 3.54–3.52 (m, 1H), 3.48–3.46 (m, 1H), 2.09–1.99 (m, 3H), 1.88–1.82 (m, 1H), 1.74–0.82 (m, other aliphatic ring protons), 0.66 (s, 3H). HRMS (ESI): calcd for C_33_H_57_NNaO_3_ [M + Na]^+^ 538.4231; found 538.4248.

#### 3.1.39. Tert-butyl((10R,13R,17R)-17-((2R)-5-hydroxyoctan-2-yl)-4,4,10,13-tetramethyl-tetradecahydro-1H-cyclopenta[a]phenanthren-3-yl)carbamate (**26c**)

Compound **26c** was synthesized in a 49% yield as a white solid using a similar procedure to that in 3.1.37. The mp was 173.1–174.5 °C. ^1^H-NMR (400 MHz, CDCl_3_) *δ* 5.47–5.45 (m, 1H), 4.45 (d, *J* = 9.2 Hz, 1H), 3.54–3.53 (m, 1H), 3.52–3.49 (m, 1H), 2.10–1.99 (m, 3H), 1.88–1.82 (m, 1H), 1.66–0.82 (m, other aliphatic ring protons), 0.66 (s, 3H). HRMS (ESI): calcd for C_34_H_59_NNaO_3_ [M + Na]^+^ 552.4387; found 552.4389.

#### 3.1.40. Tert-butyl((10R,13R,17R)-17-((2R)-5-hydroxy-6-methylheptan-2-yl)-4,4,10,13-tetramethyl-tetradecahydro-1H-cyclopenta[a]phenanthren-3-yl)carbamate (**26d**)

Compound **26d** was synthesized in a 45% yield as a white solid using a similar procedure to that in 3.1.37. and was used for the next reaction without further purification.

#### 3.1.41. Tert-butyl((10R,13R,17R)-17-((2R)-5-hydroxyoct-7-en-2-yl)-4,4,10,13-tetramethyl-tetradecahydro-1H-cyclopenta[a]phenanthren-3-yl)carbamate (**26e**)

Compound **26e** was synthesized in a 48% yield as a white solid using a similar procedure to that in 3.1.37. The mp was 191.4–192.5 °C. ^1^H-NMR (400 MHz, CDCl_3_) *δ* 5.88–5.78 (m, 1H), 5.56–5.53 (m, 1H), 5.15–5.11 (m, 2H), 4.44 (d, *J* = 10.0 Hz, 1H), 3.60–3.59 (m, 1H), 3.33–3.27 (m, 1H), 2.35–2.28 (m, 1H), 2.18–0.86 (m, other aliphatic ring protons), 0.66 (s, 3H). HRMS (ESI): calcd for C_34_H_57_NNaO_3_ [M + Na]^+^ 550.4231; found 550.4247.

#### 3.1.42. (5R)-5-((10R,13R,17R)-3-amino-4,4,10,13-tetramethyl-tetradecahydro-1H-cyclopenta[a]phenanthren-17-yl)hexan-2-ol (**5an**)

Compound **5an** was synthesized in a 92% yield as a white solid using a similar procedure to that in 3.1.25. The mp was >300 °C. ^1^H-NMR (400 MHz, CD_3_OD) *δ* 5.70–5.65 (m, 1H), 3.68–3.62 (m, 1H), 2.78–2.75 (m, 1H), 2.16–2.11 (m, 1H), 2.07–2.04 (m, 1H), 1.93–0.95 (m, other aliphatic ring protons), 0.72 (s, 3H). ^13^C-NMR (100 MHz, CD_3_OD) *δ* 149.56, 122.44, 69.19, 60.25, 58.62, 57.33, 52.35, 43.35, 41.03, 40.30, 37.89, 37.14, 36.70, 33.66, 33.19, 32.06, 29.27, 27.88, 25.42, 25.16, 24.89, 23.65, 23.40, 21.70, 21.65, 19.27, 12.37. HRMS (ESI): calcd for C_27_H_48_NO [M + H]^+^ 402.3730; found 402.3752.

#### 3.1.43. (6R)-6-((10R,13R,17R)-3-amino-4,4,10,13-tetramethyl-tetradecahydro-1H-cyclopenta[a]phenanthren-17-yl)heptan-3-ol (**5ao**)

Compound **5ao** was synthesized in a 91% yield as a white solid using a similar procedure as that in 3.1.25. The mp was >300 °C. ^1^H-NMR (400 MHz, CD_3_OD) *δ* 5.72–5.64 (m, 1H), 3.39–3.35 (m, 1H), 2.89–2.86 (m, 1H), 2.17–2.12 (m, 1H), 2.07–2.04 (m, 1H), 1.96–0.93 (m, other aliphatic ring protons), 0.72 (s, 3H). ^13^C-NMR (100 MHz, CD_3_OD) *δ* 148.87, 122.87, 74.46, 60.44, 58.50, 57.27, 52.17, 43.28, 40.91, 39.81, 37.79, 37.52, 37.15, 34.29, 33.56, 33.04, 31.95, 31.03, 30.72, 29.23, 27.79, 25.09, 24.54, 21.65, 21.58, 19.29, 12.35, 10.42. HRMS (ESI): calcd for C_28_H_49_NNaO [M + Na]^+^ 438.3706; found 438.3692.

#### 3.1.44. (7R)-7-((10R,13R,17R)-3-amino-4,4,10,13-tetramethyl-tetradecahydro-1H-cyclopenta[a]phenanthren-17-yl)octan-4-ol (**5ap**)

Compound **5ap** was synthesized in a 92% yield as a white solid using a similar procedure to that in 3.1.25. The mp was >300 °C. ^1^H-NMR (400 MHz, CD_3_OD) *δ* 5.72–5.65 (m, 1H), 3.48–3.46 (m, 1H), 2.83–2.80 (m, 1H), 2.17–2.12 (m, 1H), 2.07–2.04 (m, 1H), 1.92–0.93 (m, other aliphatic ring protons), 0.72 (s, 3H). ^13^C-NMR (100 MHz, CD_3_OD) *δ* 149.25, 122.72, 72.83, 60.36, 58.62, 57.36, 52.33, 43.35, 41.02, 40.80, 40.50, 40.10, 37.88, 37.73, 37.22, 37.00, 34.93, 33.63, 33.11, 32.05, 29.29, 27.82, 25.15, 24.92, 21.67, 19.97, 19.30, 14.51, 12.34. HRMS (ESI): calcd for C_29_H_51_NNaO [M + Na]^+^ 452.3863; found 452.3856.

#### 3.1.45. (6R)-6-((10R,13R,17R)-3-amino-4,4,10,13-tetramethyl-tetradecahydro-1H-cyclopenta[a]phenanthren-17-yl)-2-methylheptan-3-ol (**5aq**)

Compound **5aq** was synthesized in a 90% yield as a white solid using a similar procedure to that in 3.1.25. The mp was >300 °C. ^1^H-NMR (400 MHz, CD_3_OD) *δ* 5.71–5.66 (m, 1H), 3.22–3.20 (m, 1H), 2.79 (d, *J* = 11.2 Hz, 1H), 2.16–2.12 (m, 1H), 2.08–2.04 (m, 1H), 1.91–0.90 (m, other aliphatic ring protons), 0.72 (s, 3H). ^13^C-NMR (100 MHz, CD_3_OD) *δ* 149.36, 122.62, 78.11, 60.32, 58.63, 57.48, 52.34, 43.37, 41.03, 40.16, 37.88, 37.78, 37.36, 34.87, 33.65, 33.36, 32.06, 31.67, 29.32, 27.84, 25.17, 24.91, 21.68, 21.65, 19.56, 19.36, 18.00, 17.53, 12.37. HRMS (ESI): calcd for C_29_H_52_NO [M + H]^+^ 430.4043; found 430.4068.

#### 3.1.46. (7R)-7-((10R,13R,17R)-3-amino-4,4,10,13-tetramethyl-tetradecahydro-1H-cyclopenta[a]phenanthren-17-yl)oct-1-en-4-ol (**5ar**)

Compound **5ar** was synthesized in a 90% yield as a white solid using a similar procedure to that in 3.1.25. The mp was 250.4–252.6 °C. ^1^H-NMR (400 MHz, CD_3_OD) *δ* 5.91–5.81 (m, 1H), 5.68–5.63 (m, 1H), 5.06 (d, *J* = 15.6 Hz, 1H), 5.03 (d, *J* = 8.4 Hz, 1H), 3.54–3.52 (m, 1H), 2.63 (d, *J* = 11.2 Hz, 1H), 2.27–2.19 (m, 2H), 2.17–2.09 (m, 1H), 2.07–2.04 (m, 1H), 1.91–0.95 (m, other aliphatic ring protons), 0.72 (s, 3H). ^13^C-NMR (100 MHz, CD_3_OD) *δ* 150.11, 136.54, 121.98, 117.16, 72.74, 60.01, 58.66, 57.37, 52.44, 43.36, 43.09, 41.06, 40.72, 38.15, 37.93, 37.23, 34.23, 33.70, 33.01, 32.09, 29.31, 27.93, 26.23, 25.18, 24.72, 21.73, 21.66, 19.32, 12.37. HRMS (ESI): calcd for C_29_H_50_NO [M + H]^+^ 428.3887; found 428.3886.

#### 3.1.47. Tert-butyl((10R,13R,17R)-17-((R)-5-hydroxy-5-methylhexan-2-yl)-4,4,10,13-tetramethyl-tetradecahydro-1H-cyclopenta[a]phenanthren-3-yl)carbamate (**27a**)

Compound **27a** was synthesized in a 68% yield as a white solid using a similar procedure to that in 3.1.37. The mp was 220.4–223.2 °C. ^1^H-NMR (400 MHz, CDCl_3_) *δ* 5.56–5.51 (m, 1H), 4.43 (d, *J* = 10.0 Hz, 1H), 3.30–3.26 (m, 1H), 2.09–2.04 (m, 1H), 2.00–1.97 (m, 1H), 1.89–1.80 (m, 1H), 1.73–0.95 (m, other aliphatic ring protons), 0.92 (d, *J* = 6.4 Hz, 3H), 0.66 (s, 3H). HRMS (ESI): calcd for C_33_H_57_NNaO_3_ [M + Na]^+^ 538.4231; found 538.4234.

#### 3.1.48. Tert-butyl((10R,13R,17R)-17-((R)-5-ethyl-5-hydroxyheptan-2-yl)-4,4,10,13-tetramethyl-tetradecahydro-1H-cyclopenta[a]phenanthren-3-yl)carbamate (**27b**)

Compound **27b** was synthesized in a 64% yield as a white solid using a similar procedure to that in 3.1.37. The mp was 242.2–243.7 °C. ^1^H-NMR (400 MHz, CDCl_3_) *δ* 5.56–5.53 (m, 1H), 4.43 (d, *J* = 10.0 Hz, 1H), 3.33–3.26 (m, 1H), 2.09–2.04 (m, 1H), 2.00–1.97 (m, 1H), 1.90–1.80 (m, 1H), 1.73–0.96 (m, other aliphatic ring protons), 0.92 (d, *J* = 6.0 Hz, 3H), 0.85 (t, *J* = 7.2 Hz, 3H), 0.84 (t, *J* = 7.2 Hz, 3H), 0.66 (s, 3H). HRMS (ESI): calcd for C_35_H_61_NNaO_3_ [M + Na]^+^ 566.4544; found 566.4538.

#### 3.1.49. Tert-butyl((10R,13R,17R)-17-((R)-5-hydroxy-5-propyloctan-2-yl)-4,4,10,13-tetramethyl-tetradecahydro-1H-cyclopenta[a]phenanthren-3-yl)carbamate (**27c**)

Compound **27c** was synthesized in a 55% yield as a white solid using a similar procedure to that in 3.1.37. The mp was 205.2–207.6 °C. ^1^H-NMR (400 MHz, CDCl_3_) *δ* 5.57–5.54 (m, 1H), 5.29 (d, *J* = 10.0 Hz, 1H), 3.75–3.69 (m, 1H), 2.18–2.14 (m, 1H), 2.10–2.05 (m, 1H), 1.99–1.97 (m, 1H), 1.89–1.81 (m, 1H), 1.70–0.91 (m, other aliphatic ring protons), 0.66 (s, 3H). HRMS (ESI): calcd for C_37_H_65_NNaO_3_ [M + Na]^+^ 594.4857; found 594.4842.

#### 3.1.50. Tert-butyl((10R,13R,17R)-17-((R)-5-allyl-5-hydroxyoct-7-en-2-yl)-4,4,10,13-tetramethyl-tetradecahydro-1H-cyclopenta[a]phenanthren-3-yl)carbamate (**27d**)

Compound **27d** was synthesized in a 61% yield as a white solid using a similar procedure to that in 3.1.37. The mp was 181.5–183.2 °C. ^1^H-NMR (400 MHz, CDCl_3_) *δ* 5.89–5.79 (m, 2H), 5.56–5.53 (m, 1H), 5.13 (d, *J* = 8.0 Hz, 2H), 5.10 (d, *J* = 16.0 Hz, 2H), 4.43 (d, *J* = 10.0 Hz, 1H), 3.33–3.27 (m, 1H), 2.20 (d, *J* = 7.2 Hz, 4H), 2.09–2.04 (m, 1H), 1.99–1.96 (m, 1H), 1.88–1.81 (m, 1H), 1.73–0.96 (m, other aliphatic ring protons), 0.91 (d, *J* = 6.4 Hz, 3H), 0.65 (s, 3H).

#### 3.1.51. (5R)-5-((10R,13R,17R)-3-amino-4,4,10,13-tetramethyl-tetradecahydro-1H-cyclopenta[a]phenanthren-17-yl)-2-methylhexan-2-ol (**5as**)

Compound **5as** was synthesized in a 93% yield as a white solid using a similar procedure to that in 3.1.25. The mp was 215.2–217.6 °C. ^1^H-NMR (400 MHz, CDCl_3_:CD_3_OD = 5:1) *δ* 5.47–5.43 (m, 1H), 2.34–2.31 (m, 1H), 2.00–1.96 (m, 1H), 1.90–1.87 (m, 1H), 1.75–1.69 (m, 1H), 1.66–1.63 (m, 1H), 1.53–0.80 (m, other aliphatic ring protons), 0.56 (s, 3H). ^13^C-NMR (100 MHz, CDCl_3_:CD_3_OD = 5:1) *δ* 149.37, 120.11, 70.95, 58.20, 57.19, 55.61, 50.94, 42.09, 40.20, 39.78, 39.64, 37.23, 36.76, 35.85, 32.54, 30.68, 30.13, 28.77, 28.43, 28.08, 27.29, 26.63, 24.05, 23.58, 21.07, 20.42, 18.58, 11.72. HRMS (ESI): calcd for C_28_H_50_NO [M + H]^+^ 416.3887; found 416.3898.

#### 3.1.52. (6R)-6-((10R,13R,17R)-3-amino-4,4,10,13-tetramethyl-tetradecahydro-1H-cyclopenta[a]phenanthren-17-yl)-3-ethylheptan-3-ol (**5at**)

Compound **5at** was synthesized in a 93% yield as a white solid using a similar procedure to that in 3.1.25. The mp was >300 °C. ^1^H-NMR (400 MHz, CDCl_3_:CD_3_OD = 2:1) *δ* 5.47–5.45 (m, 2H), 2.47–2.43 (m, 1H), 1.99–1.92 (m, 1H), 1.87–1.84 (m, 1H), 1.76–0.74 (m, other aliphatic ring protons), 0.70 (t, *J* = 7.2 Hz, 3H), 0.69 (t, *J* = 7.2 Hz, 3H), 0.52 (s, 3H). ^13^C-NMR (100 MHz, CDCl_3_:CD_3_OD = 2:1) *δ* 148.45, 120.73, 74.68, 58.55, 57.08, 55.59, 50.78, 42.03, 39.52, 39.49, 36.80, 36.63, 36.03, 33.79, 32.42, 30.77, 30.58, 30.47, 29.08, 28.10, 27.08, 25.25, 23.98, 23.71, 20.95, 20.35, 18.56, 11.61, 7.43, 7.32. ESI-MS (*m*/*z*): 444.65 (M + H)^+^.

#### 3.1.53. (7R)-7-((10R,13R,17R)-3-amino-4,4,10,13-tetramethyl-tetradecahydro-1H-cyclopenta[a]phenanthren-17-yl)-4-propyloctan-4-ol (**5au**)

Compound **5au** was synthesized in a 92% yield as a white solid using a similar procedure to that in 3.1.25. The mp was 242.6–244.9 °C. ^1^H-NMR (400 MHz, CDCl_3_) *δ* 5.62–5.60 (m, 1H), 2.89–2.86 (m, 1H), 2.11–2.07 (m, 1H), 2.00–1.98 (m, 2H), 1.84–1.78 (m, 2H), 1.66–1.60 (m, 2H), 1.49–0.91 (m, other aliphatic ring protons), 0.66 (s, 3H). ^13^C-NMR (100 MHz, CDCl_3_) *δ* 148.14, 121.47, 74.73, 59.83, 57.29, 55.81, 50.88, 42.32, 42.00, 41.73, 39.77, 39.26, 36.89, 36.68, 36.26, 35.50, 32.67, 30.84, 29.85, 29.45, 28.39, 28.07, 25.36, 24.29, 24.08, 21.27, 20.66, 18.94, 16.91, 16.82, 14.89, 11.99. ESI-MS (*m*/*z*): 472.71 (M + H)^+^.

#### 3.1.54. (7R)-4-allyl-7-((10R,13R,17R)-3-amino-4,4,10,13-tetramethyl-tetradecahydro-1H-cyclopenta[a]phenanthren-17-yl)oct-1-en-4-ol (**5av**)

Compound **5av** was synthesized in a 90% yield as a white solid using a similar procedure to that in 3.1.25. The mp was 235.5–237.4 °C. ^1^H-NMR (400 MHz, CDCl_3_) *δ* 5.89–5.79 (m, 2H), 5.56–5.53 (m, 1H), 5.13 (d, *J* = 8.0 Hz, 2H), 5.15 (d, *J* = 16.0 Hz, 2H), 2.47–2.43 (m, 1H), 2.20 (d, *J* = 7.2 Hz, 4H), 2.10–2.05 (m, 1H), 1.99–1.96 (m, 1H), 1.87–1.82 (m, 1H), 1.74–0.94 (m, other aliphatic ring protons), 0.90 (d, *J* = 6.4 Hz, 3H), 0.65 (s, 3H). ^13^C-NMR (100 MHz, CDCl_3_) *δ* 150.07, 133.96, 133.93, 119.91, 118.73, 118.64, 73.74, 58.62, 57.39, 55.80, 51.12, 43.93, 43.71, 42.31, 40.68, 39.84, 37.56, 36.99, 36.21, 35.52, 32.77, 30.90, 29.38, 28.44, 27.78, 27.58, 24.29, 24.06, 21.35, 20.63, 18.92, 11.97. HRMS (ESI): calcd for C_32_H_54_NO [M + H]^+^ 468.4200; found 468.4200.

#### 3.1.55. (2S)-2-((10R,13S,17R)-4,4,10,13-tetramethyl-3-oxo-tetradecahydro-1H-cyclopenta[a]phenanthren-17-yl)propylacetate (**29**)

Ac_2_O (286 mg, 2.8 mmol), Et_3_N (1 g, 10 mmol), and DMAP (12 mg, 0.1 mmol) were added to a solution of 21-hydroxy-20-methylpregn-4-en-3-one **28** (717 mg, 2 mmol) in dry DCM (30 mL), and the mixture was stirred at r.t. for 12 h under a N_2_ atmosphere; TLC indicated the consumption of the starting material. Water (30 mL) was added, and the mixture was extracted with DCM (3 × 30 mL). The combined organic layer was washed with aqueous NaOH solution, brine, dried over Na_2_SO_4_, filtered, concentrated, and recrystallized in 5 mL of EtOAc to obtain compound **29** (780 mg, 97% yield) as a white solid, which was used for the next reaction without further purification.

#### 3.1.56. (10R,13S,17R)-17-((S)-1-hydroxypropan-2-yl)-4,4,10,13-tetramethyl-tetradecahydro-3H-cyclopenta[a]phenanthren-3-one (**30**)

Compound **30** was synthesized in a 67% yield as a white solid using a similar procedure to that in 3.1.3., except for the esterification procedure, and was used for the next reaction without further purification.

#### 3.1.57. (2S)-2-((10R,13S,17R)-3-amino-4,4,10,13-tetramethyl-tetradecahydro-1H-cyclopenta[a]phenanthren-17-yl)propylacetate (**31**)

Compound **31** was synthesized in a 76% yield as a white solid using a similar procedure to that in 3.1.4. The mp was 198.3–199.9 °C. ^1^H-NMR (400 MHz, CDCl_3_) *δ* 5.56–5.55 (m, 1H), 4.07 (dd, *J* = 10.8, 3.2 Hz, 1H), 3.76 (dd, *J* = 10.8, 3.2 Hz, 1H), 2.42–2.38 (m, 1H), 2.12–2.06 (m, 1H), 2.04 (s, 3H), 2.00–1.97 (m, 1H), 1.83–0.89 (m, other aliphatic ring protons), 0.68 (s, 3H). HRMS (ESI): calcd for C_26_H_43_NNaO_2_ [M + Na]^+^ 424.3186; found 424.3199.

#### 3.1.58. (2S)-2-((10R,13S,17R)-3-amino-4,4,10,13-tetramethyl-tetradecahydro-1H-cyclopenta[a]phenanthren-17-yl)propan-1-ol (**5aw**)

Compound **5aw** was synthesized in a 90% yield as a white solid using a similar procedure to that in 3.1.31. The mp was 170.6–173.8 °C. ^1^H-NMR (400 MHz, CDCl_3_:CD_3_OD = 5:1) *δ* 5.47–5.45 (m, 1H), 3.52–3.49 (m, 1H), 3.21–3.16 (m, 1H), 2.29–2.25 (m, 1H), 2.03–1.96 (m, 1H), 1.93–1.90 (m, 1H), 1.77–1.70 (m, 1H), 1.67–1.63 (m, 1H), 1.57–0.76 (m, other aliphatic ring protons), 0.60 (s, 3H). ^13^C-NMR (100 MHz, CDCl_3_:CD_3_OD = 5:1) *δ* 149.84, 119.76, 67.37, 58.02, 56.99, 52.35, 51.00, 42.25, 40.56, 39.56, 38.77, 37.44, 36.82, 32.58, 30.75, 27.73, 27.40, 27.31, 24.23, 23.52, 21.14, 20.45, 16.60, 11.82. HRMS (ESI) calcd for C_24_H_42_NO [M + H]^+^ 360.3261; found 360.3267.

#### 3.1.59. 1-(4-chloro-3-(trifluoromethyl)phenyl)-3-(4-hydroxyphenyl)urea (**34**)

Compound **33** (443 mg, 2 mmol) was added to a solution of compound **32** (218 mg, 2 mmol) in dry DCM (10 mL). The reaction was stirred at r.t. for 10 min, and the starting material disappeared. Water (30 mL) was added, and the mixture was extracted with DCM (3 × 30 mL). The combined organic layer was washed with brine, dried over Na_2_SO_4_, filtered, concentrated, and recrystallized in 2 mL of MeCN to obtain compound **34** (635 mg, 99% yield) as a brown solid. The mp was 232.1–233.6 °C. ^1^H-NMR (400 MHz, CDCl_3_:CD_3_OD = 5:1) *δ* 7.74 (d, *J* = 2.0 Hz, 1H), 7.53 (dd, *J* = 8.4, 2.0 Hz, 1H), 7.33 (d, *J* = 8.4 Hz, 1H), 7.13 (d, *J* = 8.8 Hz, 2H), 6.73 (d, *J* = 8.8 Hz, 2H). HRMS (ESI): calcd for C_14_H_11_ClF_3_N_2_O_2_ [M + H]^+^ 331.0456; found 331.0451.

#### 3.1.60. 1-(4-chloro-3-(trifluoromethyl)phenyl)-3-((10R,13R,17R)-17-((R)-5-hydroxypentan-2-yl)-4,4,10,13-tetramethyl-2,3,4,7,8,9,10,11,12,13,14,15,16,17-tetradecahydro-1H-cyclopenta[a]phenanthren-3-yl)urea (**5ax**)

Compound **5ax** was synthesized in a 96% yield as a white solid using a similar procedure to that in 3.1.59. The mp was 221.1–223.7 °C. ^1^H-NMR (400 MHz, CDCl_3_:CD_3_OD = 3:1) *δ* 7.72 (s, 1H), 7.47 (d, *J* = 8.8 Hz, 1H), 7.29 (d, *J* = 8.8 Hz, 1H), 5.73 (d, *J* = 10.0 Hz, 1H), 5.54–5.50 (m, 1H), 3.49 (t, *J* = 4.8 Hz, 2H), 3.47–3.40 (m, 1H), 2.07–2.00 (m, 1H), 1.98–1.94 (m, 1H), 1.85–1.76 (m, 1H), 1.72–0.86 (m, other aliphatic ring protons), 0.63 (s, 3H). ^13^C-NMR (100 MHz, CDCl_3_:CD_3_OD = 3:1) *δ* 156.05, 149.73, 139.29, 131.82, 128.50 (q, *J* = 31.2 Hz), 123.94, 122.81 (q, *J* = 228.0 Hz), 122.31, 120.48, 117.27 (d, *J* = 5.8 Hz), 63.13, 57.41, 56.41, 56.18, 51.08, 42.39, 40.54, 39.91, 37.62, 36.87, 35.88, 32.73, 32.11, 31.03, 29.37, 28.43, 27.89, 26.43, 24.80, 24.32, 21.36, 20.72, 18.74, 11.97. ^19^F-NMR (375 MHz, CDCl_3_:CD_3_OD = 3:1) *δ*−63.05. HRMS (ESI): calcd for C_34_H_48_ClF_3_N_2_NaO_2_ [M + Na]^+^ 631.3249; found 631.3247.

#### 3.1.61. Tert-butyl((10R,13R,17R)-17-((R)-5-bromopentan-2-yl)-4,4,10,13-tetramethyl-tetradecahydro-1H-cyclopenta[a]phenanthren-3-yl)carbamate (**35**)

PPh_3_ (131 mg, 0.5 mmol), imidazole (33 mg, 0.5 mmol), and CBr_4_ (150 mg, 0.45 mmol) were added to a solution of compound **12** (146 mg, 0.3 mmol) in dry DMF (10 mL). The reaction was stirred at r.t. for 2 h, and TLC indicated the consumption of the starting material. Upon the addition of MeOH (0.2 mL) for quenching, water (30 mL) was added and the mixture was extracted with EtOAc (3 × 30 mL). The combined organic layer was washed with water, brine, dried over Na_2_SO_4_, filtered, concentrated, and purified by silica gel column chromatography using PE/EtOAc (50:1, *v*/*v*) to obtain compound **35** (126 mg, 76% yield) as a white solid. The mp was 192.3–194.7 °C. ^1^H-NMR (400 MHz, CDCl_3_) *δ* 5.59–5.57 (m, 1H), 4.43 (d, *J* = 10.0 Hz, 1H), 3.43–3.35 (m, 2H), 3.33–3.27 (m, 1H), 2.11–2.04 (m, 1H), 2.00–1.97 (m, 1H), 1.92–1.83 (m, 2H), 1.80–0.95 (m, other aliphatic ring protons), 0.92 (d, *J* = 6.4 Hz, 3H), 0.66 (s, 3H).

#### 3.1.62. Tert-butyl((10R,13R,17R)-17-((R)-5-(4-(3-(4-chloro-3-(trifluoromethyl)phenyl)ureido)phenoxy)pentan-2-yl)-4,4,10,13-tetramethyl-tetradecahydro-1H-cyclopenta[a]phenanthren-3-yl)carbamate (**36**)

Compound **36** was synthesized in a 68% yield as a white solid using a similar procedure to that in 3.1.6 and used for the next reaction without further purification.

#### 3.1.63. 1-(4-(((4R)-4-((10R,13R,17R)-3-amino-4,4,10,13-tetramethyl-tetradecahydro-1H-cyclopenta[a]phenanthren-17-yl)pentyl)oxy)phenyl)-3-(4-chloro-3-(trifluoromethyl)phenyl)urea (**5ay**)

Compound **5ay** was synthesized in a 94% yield as a white solid using a similar procedure to that in 3.1.25. The mp was 192.3–194.5 °C. ^1^H-NMR (400 MHz, CDCl_3_:CD_3_OD = 5:1) *δ* 7.65 (s, 1H), 7.54 (d, *J* = 8.4 Hz, 1H), 7.31 (d, *J* = 8.4 Hz, 1H), 7.19 (d, *J* = 8.4 Hz, 2H), 6.79 (d, *J* = 8.4 Hz, 2H), 5.54–5.50 (m, 1H), 3.84 (t, *J* = 7.6 Hz, 2H), 2.36–2.32 (m, 1H), 2.00–1.97 (m, 1H), 1.88–1.79 (m, 2H), 1.71–0.86 (m, other aliphatic ring protons), 0.66 (s, 3H). ^13^C-NMR (100 MHz, CDCl_3_:CD_3_OD = 5:1) *δ* 155.68, 153.57, 149.85, 138.40, 131.76, 131.02, 128.43 (d, *J* = 31.2 Hz), 124.57, 122.78 (q, *J* = 271.9 Hz), 122.65, 122.09, 119.89 (2C), 117.59 (d, *J* = 4.4 Hz), 115.02 (2C), 68.96, 58.15, 57.30, 55.89, 51.06, 42.24, 40.60, 39.77, 37.48, 36.89, 35.59, 32.66, 32.09, 30.80, 28.29, 27.49, 27.38, 25.91, 24.18, 23.62, 21.22, 20.53, 18.65, 11.88. ^19^F-NMR (375 MHz, CDCl_3_:CD_3_OD = 5:1) *δ* -62.77. HRMS (ESI): calcd for C_40_H_54_ClF_3_N_3_O_2_ [M + H]^+^ 700.3851; found 700.3900.

#### 3.1.64. Tert-butyl((10R,13R,17R)-17-((R)-5-hydroxypentan-2-yl)-4,4,10,13-tetramethyl-7-oxo-tetradecahydro-1H-cyclopenta[a]phenanthren-3-yl)carbamate (**37**)

Compound **37** was synthesized in a 96% yield as a white solid using a similar procedure to that in 3.1.19., and the reaction temperature was r.t. The mp was 225.5–227.2 °C. ^1^H-NMR (400 MHz, CDCl_3_) *δ* 5.94 (s, 1H), 4.49 (d, *J* = 10.0 Hz, 1H), 3.60 (t, *J* = 6.0 Hz, 2H), 3.49–3.43 (m, 1H), 2.31–2.26 (m, 1H), 2.22–2.17 (m, 1H), 2.03–2.00 (m, 1H), 1.89–1.85 (m, 1H), 1.81–1.00 (m, other aliphatic ring protons), 0.93 (d, *J* = 6.4 Hz, 3H), 0.68 (s, 3H). HRMS (ESI): calcd for C_31_H_51_NNaO_4_ [M + Na]^+^ 524.3710; found 524.3728.

#### 3.1.65. Tert-butyl((10R,13R,17R)-17-((R)-5-bromopentan-2-yl)-4,4,10,13-tetramethyl-7-oxo-tetradecahydro-1H-cyclopenta[a]phenanthren-3-yl)carbamate (**38**)

Compound **38** was synthesized in a 72% yield as a white solid using a similar procedure to that in 3.1.61. The mp was 226.3–228.8 °C. ^1^H-NMR (400 MHz, CDCl_3_) *δ* 5.95 (s, 1H), 4.48 (d, *J* = 10.0 Hz, 1H), 3.49–3.42 (m, 1H), 3.40–3.32 (m, 2H), 2.31–2.28 (m, 1H), 2.23–2.17 (m, 1H), 2.02–1.09 (m, other aliphatic ring protons), 0.93 (d, *J* = 6.4 Hz, 3H), 0.68 (s, 3H). HRMS (ESI): calcd for C_31_H_50_BrNNaO_3_ [M + Na]^+^ 586.2866; found 586.2864.

#### 3.1.66. Tert-butyl((10R,13R,17R)-17-((R)-5-(4-(3-(4-chloro-3-(trifluoromethyl) phenyl)ureido)phenoxy)pentan-2-yl)-4,4,10,13-tetramethyl-7-oxo-tetradecahydro-1H-cyclopenta[a]phenanthren-3-yl)carbamate (**39**)

Compound **39** was synthesized in a 77% yield as a white solid using a similar procedure to that in 3.1.6. The mp was 223.1–224.3 °C. ^1^H-NMR (400 MHz, CDCl_3_) *δ* 7.86 (s, 1H), 7.55 (s, 1H), 7.51 (d, *J* = 8.8 Hz, 1H), 7.47 (s, 1H), 7.29 (d, *J* = 8.8 Hz, 1H), 7.16 (d, *J* = 8.4 Hz, 2H), 6.77 (d, *J* = 8.4 Hz, 2H), 5.93 (s, 1H), 4.58 (d, *J* = 10.0 Hz, 1H), 3.81 (t, *J* = 6.0 Hz, 2H), 3.47–3.41 (m, 1H), 2.28–2.18 (m, 2H), 2.05–1.08 (m, other aliphatic ring protons), 0.95 (d, *J* = 6.4 Hz, 3H), 0.68 (s, 3H). HRMS (ESI): calcd for C_45_H_59_ClF_3_N_3_NaO_5_ [M + Na]^+^ 836.3988; found 836.3964.

#### 3.1.67. Tert-butyl((10R,13R,17R)-17-((R)-5-(4-(3-(4-chloro-3-(trifluoromethyl)phenyl)ureido)phenoxy)pentan-2-yl)-7-hydroxy-4,4,10,13-tetramethyl-tetradecahydro-1H-cyclopenta[a]phenanthren-3-yl)carbamate (**40**)

Compound **40** was synthesized in a 94% yield as a white solid using a similar procedure to that in 3.1.21. The mp was 248.7–249.4 °C. ^1^H-NMR (400 MHz, CDCl_3_) *δ* 7.84 (s, 1H), 7.55–7.53 (m, 2H), 7.49 (d, *J* = 8.8 Hz, 1H), 7.28 (d, *J* = 8.8 Hz, 1H), 7.12 (d, *J* = 8.4 Hz, 2H), 6.76 (d, *J* = 8.4 Hz, 2H), 5.46–5.44 (m, 1H), 4.59 (d, *J* = 10.0 Hz, 1H), 3.85–3.83 (m, 1H), 3.80 (t, *J* = 6.0 Hz, 2H), 3.28–3.22 (m, 1H), 1.99–0.86 (m, other aliphatic ring protons), 0.66 (s, 3H). HRMS (ESI): calcd for C_45_H_61_ClF_3_N_3_NaO_5_ [M + Na]^+^ 838.4150; found 838.4134.

#### 3.1.68. 1-(4-(((4R)-4-((10R,13R,17R)-3-amino-7-hydroxy-4,4,10,13-tetramethyl-tetradecahydro-1H-cyclopenta[a]phenanthren-17-yl)pentyl)oxy)phenyl)-3-(4-chloro-3-(trifluoromethyl)phenyl)urea (**5az**)

Compound **5az** was synthesized in a 96% yield as a white solid using a similar procedure to that in 3.1.25. The mp was 198.1–199.7 °C. ^1^H-NMR (400 MHz, CDCl_3_:CD_3_OD = 20:1) *δ* 7.65 (s, 1H), 7.52 (d, *J* = 8.4 Hz, 1H), 7.29 (d, *J* = 8.4 Hz, 1H), 7.19 (d, *J* = 8.4 Hz, 2H), 6.77 (d, *J* = 8.4 Hz, 2H), 5.73 (d, *J* = 4.4 Hz, 1H), 3.88–3.82 (m, 3H), 2.36–2.32 (m, 1H), 1.96–1.94 (m, 1H), 1.85–0.91 (m, other aliphatic ring protons), 0.93 (d, *J* = 6.0 Hz, 3H), 0.64 (s, 3H). ^13^C-NMR (100 MHz, CDCl_3_:CD_3_OD = 20:1) *δ* 155.43, 154.73, 153.56, 138.41, 131.65, 131.07, 128.31 (d, *J* = 31.3 Hz), 124.38, 122.71 (q, *J* = 271.1 Hz), 122.54, 122.14, 121.77 (2C), 117.50, 114.90 (2C), 68.85, 65.78, 57.75, 55.57, 49.22, 43.33, 41.88, 40.59, 38.96, 37.89, 37.02, 36.59, 35.53, 32.01, 28.28, 27.45, 27.03, 25.78, 24.15, 23.41, 20.00, 19.69, 18.57, 11.43. ^19^F-NMR (375 MHz, CDCl_3_:CD_3_OD = 20:1) *δ* -62.73. HRMS (ESI): calcd for C_40_H_54_ClF_3_N_3_O_3_ [M + H]^+^ 716.3800; found 716.3761.

#### 3.1.69. Tert-butyl((10R,13S,17R)-17-((S)-1-hydroxypropan-2-yl)-4,4,10,13-tetramethyl-tetradecahydro-1H-cyclopenta[a]phenanthren-3-yl)carbamate (**41**)

Compound **41** was synthesized in a 98% yield as a white solid using a similar procedure to that in 3.1.16. The mp was 213.0–214.3 °C. ^1^H-NMR (400 MHz, CDCl_3_) *δ* 5.56–5.52 (m, 1H), 4.44 (d, *J* = 9.6 Hz, 1H), 3.65–3.62 (m, 1H), 3.37–3.26 (m, 2H), 2.10–2.05 (m, 1H), 2.00–1.97 (m, 1H), 1.86–1.77 (m, 1H), 1.69–0.85 (m, other aliphatic ring protons), 0.68 (s, 3H). HRMS (ESI): calcd for C_29_H_49_NNaO_3_ [M + Na]^+^ 482.3605; found 482.3615.

#### 3.1.70. Tert-butyl((10R,13S,17R)-17-((S)-1-bromopropan-2-yl)-4,4,10,13-tetramethyl-tetradecahydro-1H-cyclopenta[a]phenanthren-3-yl)carbamate (**42**)

Compound **42** was synthesized in a 78% yield as a white solid using a similar procedure to that in 3.1.61. The mp was 232.2–234.8 °C. ^1^H-NMR (400 MHz, CDCl_3_) *δ* 5.56–5.53 (m, 1H), 4.43 (d, *J* = 10.0 Hz, 1H), 3.51–3.49 (m, 1H), 3.35–3.31 (m, 2H), 2.10–2.05 (m, 1H), 1.97–1.94 (m, 1H), 1.88–1.86 (m, 1H), 1.73–0.85 (m, other aliphatic ring protons), 0.68 (s, 3H). HRMS (ESI): calcd for C_29_H_48_BrNNaO_2_ [M + Na]^+^ 544.2761; found 544.2759.

#### 3.1.71. Tert-butyl((10R,13S,17R)-17-((S)-1-(4-(3-(4-chloro-3-(trifluoromethyl)phenyl)ureido)phenoxy)propan-2-yl)-4,4,10,13-tetramethyl-tetradecahydro-1H-cyclopenta[a]phenanthren-3-yl)carbamate (**43**)

Compound **43** was synthesized in an 86% yield as a white solid using a similar procedure to that in 3.1.6 and used for the next reaction without further purification.

#### 3.1.72. 1-(4-((2S)-2-((10R,13S,17R)-3-amino-4,4,10,13-tetramethyl-tetradecahydro-1H-cyclopenta[a]phenanthren-17-yl)propoxy)phenyl)-3-(4-chloro-3-(trifluoromethyl)phenyl)urea (**5ba**)

Compound **5ba** was synthesized in an 86% yield as a white solid using a similar procedure to that in 3.1.25. The mp was 197.1–198.6 °C. ^1^H-NMR (400 MHz, CDCl_3_:CD_3_OD = 10:1) *δ* 7.65 (s, 1H), 7.48 (d, *J* = 8.4 Hz, 1H), 7.27 (d, *J* = 8.4 Hz, 1H), 7.17 (d, *J* = 8.4 Hz, 2H), 6.76 (d, *J* = 8.4 Hz, 2H), 5.52–5.50 (m, 1H), 3.81 (dd, *J* = 8.8, 2.4 Hz, 1H), 3.55 (dd, *J* = 8.8, 8.0 Hz, 1H), 2.35–2.31 (m, 1H), 2.08–1.97 (m, 2H), 1.80–1.77 (m, 2H), 1.70–0.80 (m, other aliphatic ring protons), 0.68 (s, 3H). ^13^C-NMR (100 MHz, CDCl_3_:CD_3_OD = 10:1) *δ* 155.86, 153.64, 149.71, 138.39 (d, *J* = 2.5 Hz), 131.64, 130.90, 128.32 (d, *J* = 31.2 Hz), 124.41, 122.71 (q, *J* = 271.4 Hz), 122.57, 121.88, 119.81 (2C), 117.51 (d, *J* = 5.2 Hz), 114.90 (2C), 73.42, 58.05, 56.92, 52.61, 50.97, 42.35, 40.48, 39.53, 37.38, 36.80, 36.46, 32.55, 30.75, 27.78, 27.36, 27.18, 24.21, 23.50, 21.11, 20.44, 17.25, 11.84. ^19^F-NMR (375 MHz, CDCl_3_:CD_3_OD = 10:1) *δ* -62.78. HRMS (ESI): calcd for C_38_H_50_ClF_3_N_3_O_2_ [M + H]^+^ 672.3538; found 672.3574.

### 3.2. SHP1 and SHP2 Enzyme Assay

The proteins SHP1 and SHP2, containing a GST-tag, were expressed in *E. coli*. 6,8-Difluoro-4-methylumbelliferyl phosphate (DiFMUP) was used as the fluorogenic substrate. Fluorescence data were collected by the Envision (PerkinElmer, Waltham, MA, USA) plate reader, using excitation and emission wavelengths of 358 and 455 nm, respectively. A sigmoidal dose-response curve was fitted to the data, and the EC_50_ values were calculated using GraphPad Prism. In the data processing, the fitting and curves were calculated using nonlinear regression, and the fold value of the compound was calculated using the software GraphPad Prism 8. The folds were obtained according to the equation: Y = Bottom + (Top-Bottom)/(1 + 10^((LogEC_50_-X) × HillSlope)). The data represent mean values ± standard error of the mean (SEM) of eight-point experiments, each performed in triplicates from three independent experiments.

### 3.3. CCK8 Cell Viability Assay

Acute lymphoblastic leukemia (ALL) cell line RS4;11 (2.5 × 10^4^ cells per well), acute promyelocytic leukemia (APL) cell line NB4 (1 × 10^4^ cells per well), and non-small cell lung cancer (NSCLC) cell line NCI-H1299 (1.5 × 10^3^ cells per well) were seeded into 96-well plates. After treatment with different concentrations of drugs, the cells were incubated in quadruple for 72 h. Thereafter, a 10 μL CCK8 solution was added to each well. After a 3 h incubation at 37 °C, the plates were read for absorbance at 450 nm and 650 nm using a SpectraMax Molecular Devices microplate reader. The final data were calibrated by OD450 nm-OD650 nm. The inhibition rates of proliferation were calculated with the following equation:

Inhibition ratio = (OD_DMSO_-OD_Compd_)/(OD_DMSO_-OD_blank_) × 100%. (1)

The concentrations of the compounds that inhibited cell growth by 50% (IC_50_) were calculated using GraphPad Prism version 5.0. NVP-2 was used as a positive control.

### 3.4. Molecular Docking

The Glide (Maestro 10.2) package of Schrödinger suite 2015 (https://www.schrodinger.com, accessed on 1 September 2015) was employed for the molecular docking investigation in SP mode. The SHP1 crystal structure (open conformation, PDB ID: 3PS5, 3.10 Å of resolution) was downloaded from the Protein Data Bank (https://www.rcsb.org, accessed on 1 March 2021). The receptors and ligands were prepared using the Protein Preparation Wizard and LigPrep modules embedded in the Schrödinger suite, respectively. All water molecules and sulfate ions were removed, and hydrogen atoms were added. The optimized geometry of the ligands with minimum energy was attained using an OPLS 2005 force field. The grid box was set to search all over the protein for binding sites. All the other parameters were adjusted as a default. The graphics were generated by PyMOL 2.6.0 (https://pymol.org/2, accessed on 1 November 2022) and UCSF Chimera 1.16.

### 3.5. Statistical Analysis

The data are presented as the mean ± SEM unless otherwise noted. Statistical significance was calculated using the two-tailed Student’s t-test; a *p*-value < 0.05 was considered statistically significant. Statistical analysis was conducted using GraphPad Prism.

## 4. Conclusions

In summary, a new series of 3-amino-4,4-dimethyl lithocholic acid derivatives were designed, synthesized, and evaluated for SHP1 activation ability. The SAR data presented herein demonstrated that the introduction of a free amino group at the C-3 position was essential for its potency. The primary alcohol moiety in the side chain can be replaced by other chemical groups while maintaining or significantly increasing potency. A large, >9-fold increase in potency was observed with the hybrid of compound **5aa** and the diphenyl urea group. Combining these observations led to the synthesis of the diphenyl urea hybrid molecules **5az-ba**, which proved to be highly potent SHP1 activators, with EC_50_ values of 2.10 and 1.54 μM, respectively. The compounds **5az-ba** also showed good selectivity in the SHP2 activation assay. In the in vitro cellular assays, **5az-ba** showed potent activities with IC_50_ values up to 1.65 μM against acute lymphoblastic leukemia cell line RS4;11, acute promyelocytic leukemia cell line NB4, and lung cancer cell line NCI-H1299. The molecular modeling study revealed that compound **5ba** gains hydrogen bond interactions with Thr80, Gln81, Lys97, and Asn472. The deeper and closer binding of the compound to the central allosteric pocket of SHP1 may explain the stronger activation. All of the studies presented here support the proposal that **5az-ba** may provide good references for the development of new anticancer drugs targeting SHP1 activation, and they deserve further research.

## Figures and Tables

**Table 1:** SHP1-activating effects of compounds **5aa-af**.

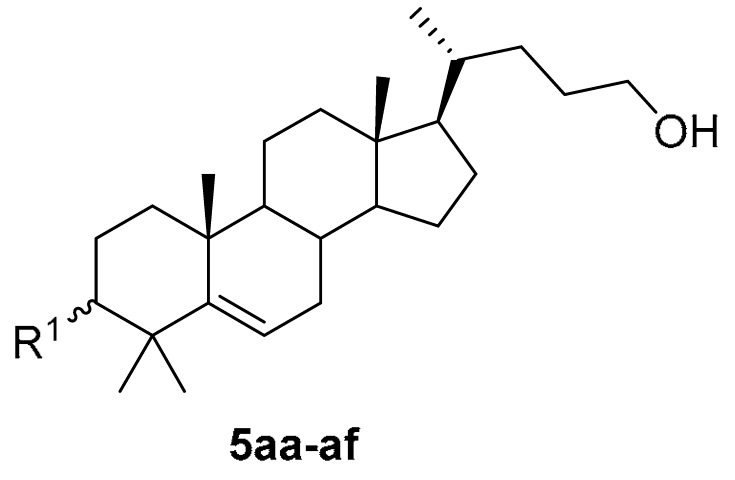			
Compd	R^1^	EC_50_ (μM)	Maximum Activation Fold
**5aa**	-NH_2_	19.23 ± 4.59	6.03 ± 0.87
**5ab**	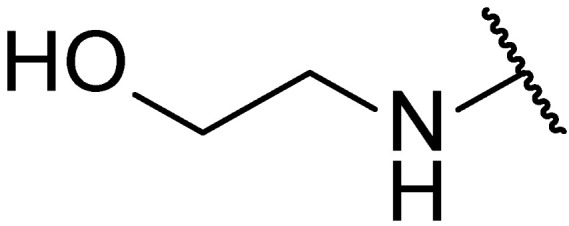	39.15 ± 3.7	ND ^a^
**5ac**	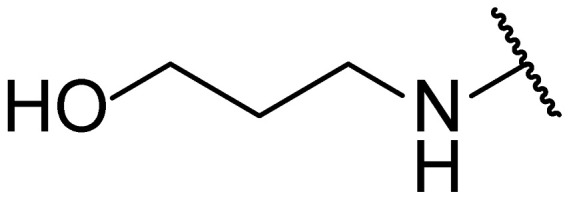	30.73 ± 3.8	ND
**5ad**	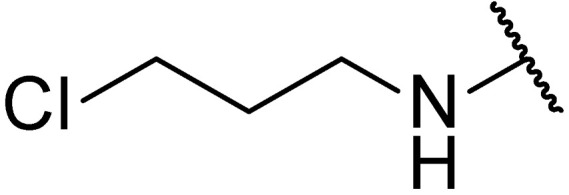	47.34 ± 0.34	ND
**5ae**	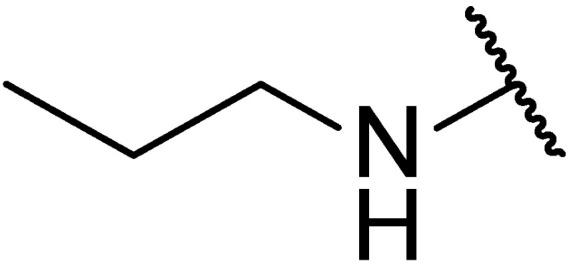	>50	ND
**5af**	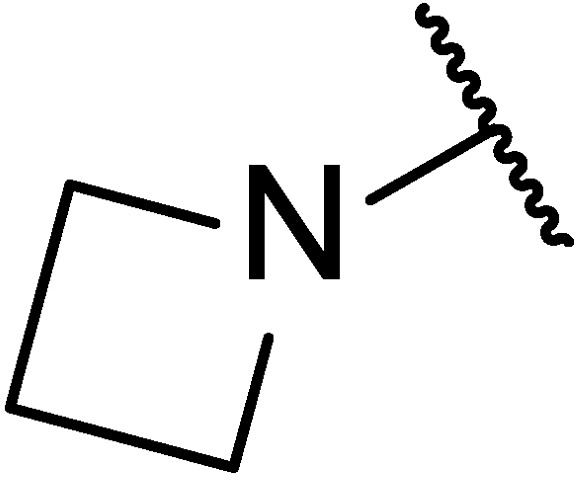	>50	ND

^a^ ND: not determined.

**Table 2:** SHP1-activating effects of compounds **5ag-ak**.

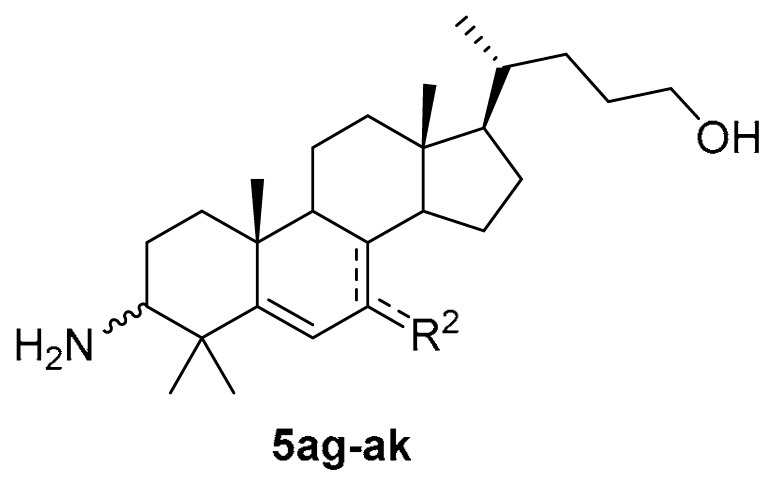			
Compd	R^2^	EC_50_ (μM)	Maximum Activation Fold
**5ag**	=O	33.76 ± 2.93	1.84 ± 0.11
**5ah**	=NOH	34.38 ± 2.47	2.13 ± 0.28
**5ai**	-OH	>50	ND ^a^
**5aj**	-NH_2_	>50	ND
**5ak** ^b^	/	19.95 ± 3.34	7.74 ± 0.20

^a^ ND: not determined. ^b^ 5,7-diene compound.

**Table 3:** SHP1-activating effects of compounds **5al-aw**.

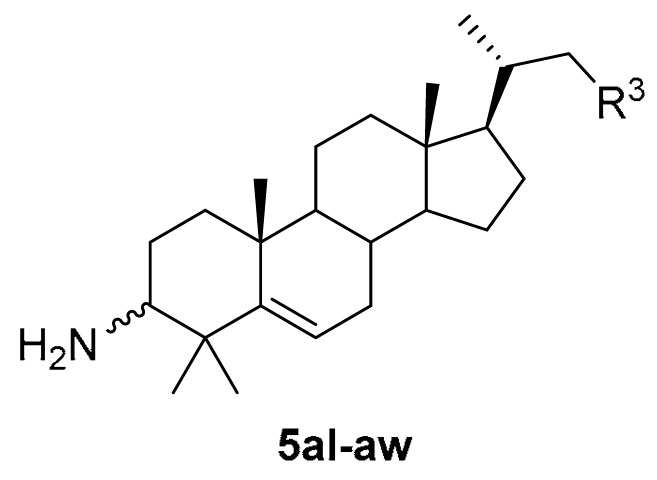			
Compd	R^3^	EC_50_ (μM)	Maximum Activation Fold
**5al**	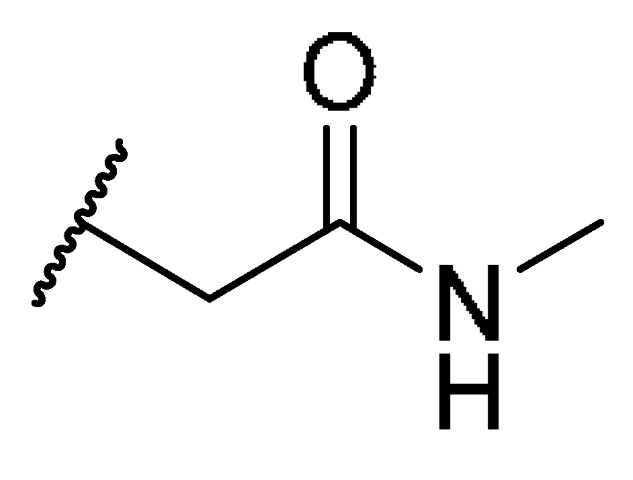	40.54 ± 6.58	6.42 ± 0.28
**5am**	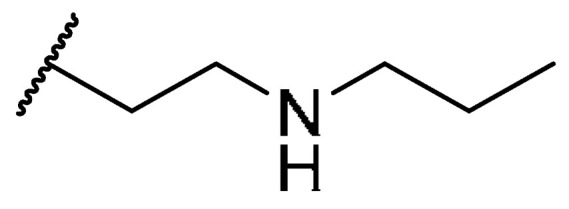	>50	ND ^a^
**5an**	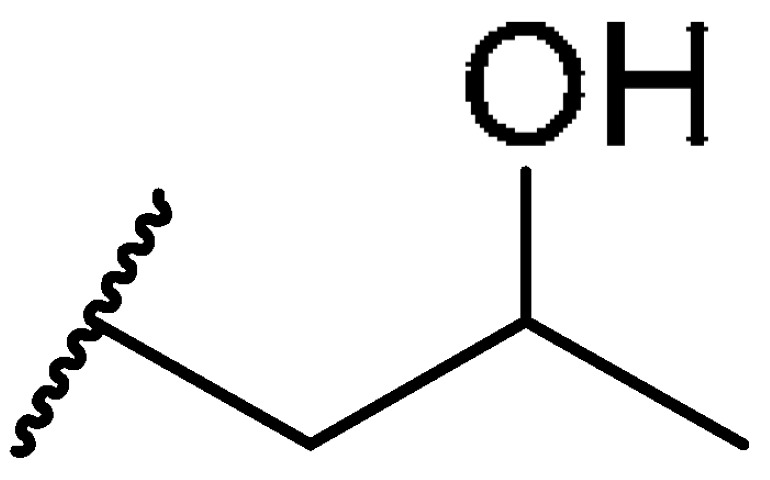	17.61 ± 1.75	6.33 ± 0.01
**5ao**	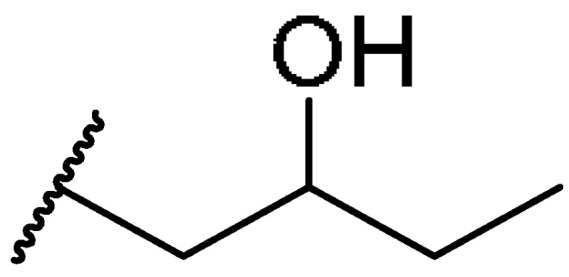	15.52 ± 4.38	6.59 ± 0.48
**5ap**	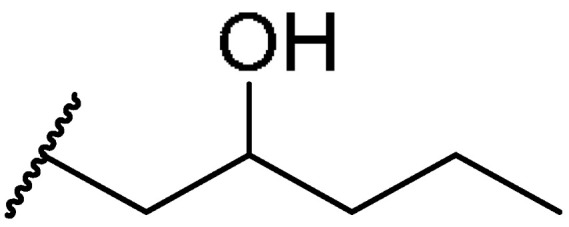	38.11 ± 3.76	6.65 ± 0.35
**5aq**	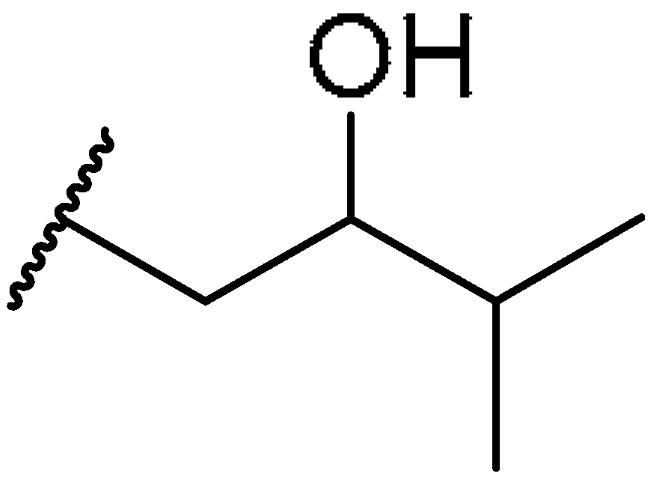	22.15 ± 3.73	6.32 ± 0.34
**5ar**	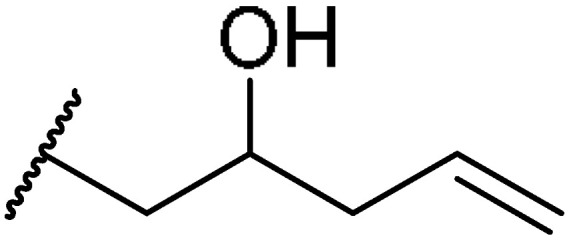	12.88 ± 0.75	6.94 ± 0.17
**5as**	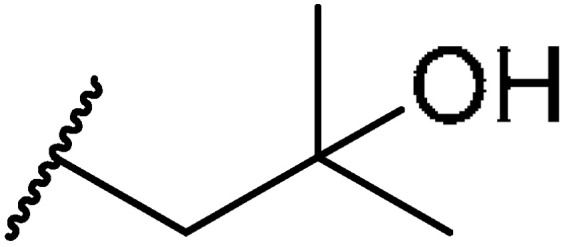	22.00 ± 3.84	5.73 ± 0.32
**5at**	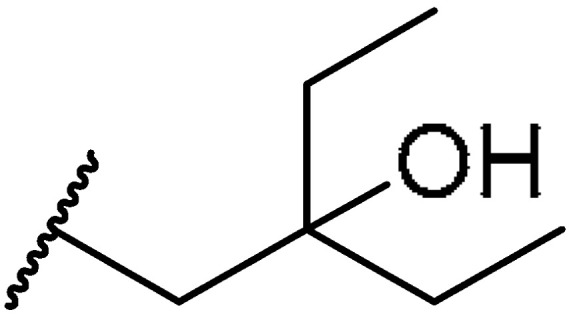	12.88 ± 0.30	6.50 ± 0.12
**5au**	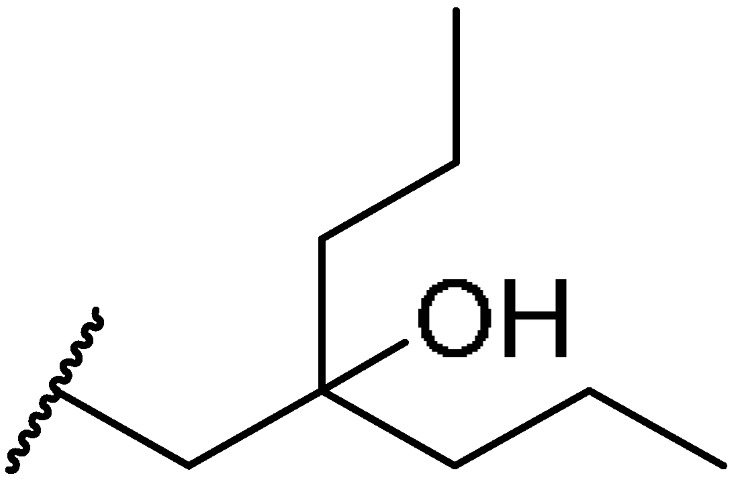	24.40 ± 3.61	6.42 ± 0.28
**5av**	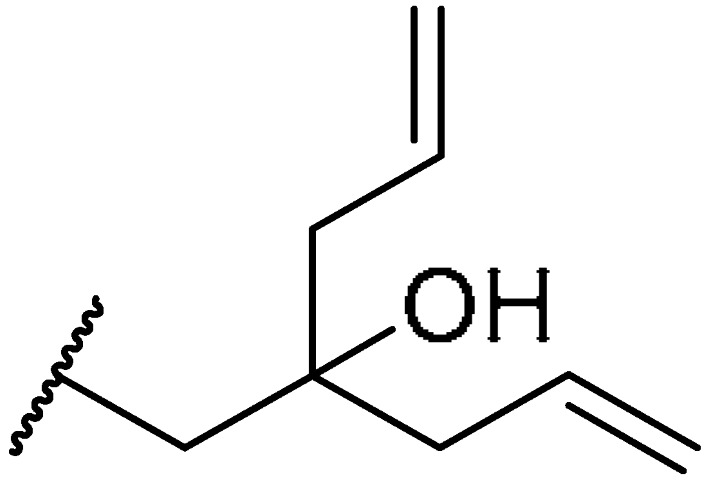	35.80 ± 1.92	5.90 ± 0.42
**5aw**	OH	35.49 ± 3.15	ND

^a^ ND: not determined.

**Table 4:** SHP1-activating effects of compounds **5ax-ba**.

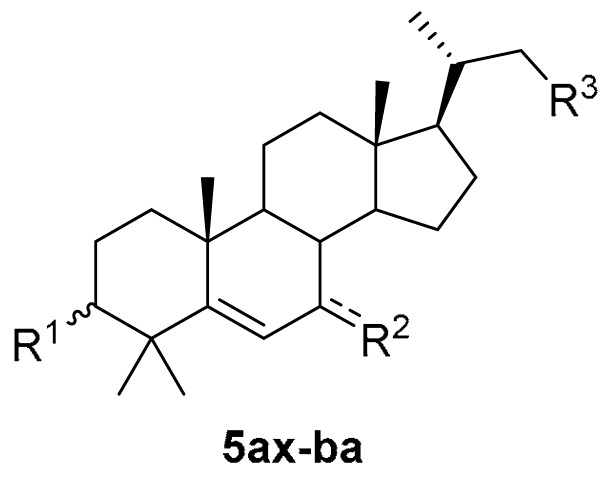					
Compd	R^1^	R^2^	R^3^	EC_50_ (μM)	Maximum Activation Fold
**5ax**	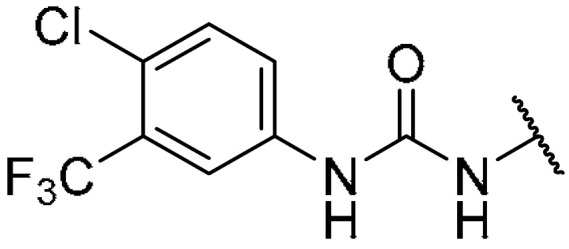	H	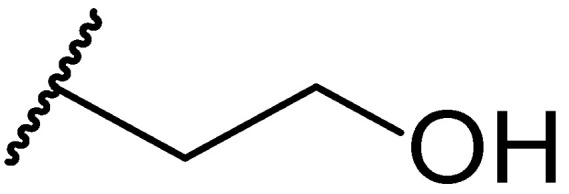	>50	ND ^a^
**5ay**	-NH_2_	H	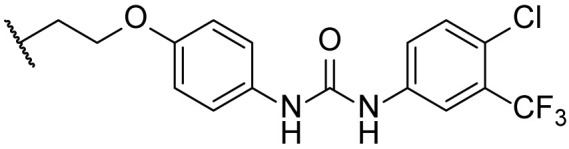	2.05 ± 0.14	5.54 ± 0.24
**5az**	-NH_2_	-OH	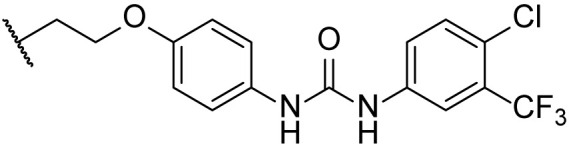	2.10 ± 0.00	8.79 ± 0.17
**5ba**	-NH_2_	H	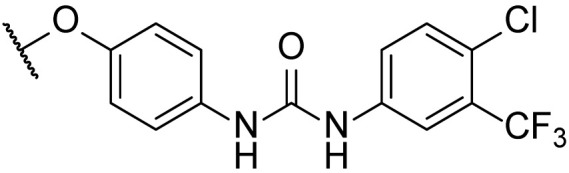	1.54 ± 0.24 ^b^	7.63 ± 0.20

^a^ ND: not determined. ^b^ Poor solubility in 10 mM DMSO.

**Table 5:** Anti-tumor effects of compounds **5az-ba**.

Compd	IC_50_ (μM) ^a^		
	RS4;11	NB4	NCI-H1299
5az	1.65 ± 0.05	2.04 ± 0.11	2.48 ± 0.20
5ba	4.00 ± 0.22	3.74 ± 0.39	5.51 ± 0.23

^a^ Cell viability was assayed in cultured cells incubated with indicated concentrations of compounds for 72 h by CCK8 assay. Data are given as mean values of three independent experiments.

## Data Availability

The date presents in study are available in Supplementary Materials.

## Supplementary Materials

The following supporting information can be downloaded at: https://www.mdpi.com/article/10.3390/molecules28062488/s1, dose response curves for SHP1 enzyme assay and CCK8 cell viability assay. ^1^H-NMR, ^13^C-NMR and HRMS spectra for all new compounds. Molecular docking of SHP1 (PDB ID: 3PS5) with compound **5ba**.

## Sample Availability

Samples of the compounds are not available from the authors.
