# Supplementary material for: Synthesis and Biological Evaluation of 3-Amino-4,4-Dimethyl Lithocholic Acid Derivatives as Novel, Selective, and Cellularly Active Allosteric SHP1 Activators
Source: Molecules. 2023 Mar 8;28(6):2488. doi: 10.3390/molecules28062488 (PMC10056611; doi:10.3390/molecules28062488)
Supplement: Supplementary file 1 [file molecules-28-02488-s001.zip › Supplementary File.pdf]

Synthesis and biological evaluation of 3-amino-4,4-dimethyl lithocholic acid derivatives as novel, selective, and cellularly active allosteric SHP1 activators

Huiqing Chen [1], Zekun Liu [1], Lixin Gao [2,3], Li-Fang Yu [1], Yubo Zhou [2,4], Jie Tang [1,5], Jia Li [2,4,*], Fan Yang [1,*]

[1] Shanghai Engineering Research Center of Molecular Therapeutics and New Drug Development, School of Chemistry and Molecular Engineering, East China Normal University, Shanghai 200062, China

[2] National Center for Drug Screening, Shanghai Institute of Material Medica, Chinese Academy of Science, Shanghai 201203, China

[3] School of Pharmaceutical Science, Jiangnan University, Wuxi 214122, China

[4] Zhongshan Institute for Drug Discovery, Shanghai Institute of Materia Medica, Chinese Academy of Sciences, Zhongshan Tsuihang New District, Guangdong 528400, China

[5] Shanghai Greenchem & Biotech Co., Ltd., Shanghai 200062, China

* Correspondence: fyang@chem.ecnu.edu.cn (F. Y.); jli@simm.ac.cn (J. L.)

Contents

Dose response curves for SHP1 enzyme assay S2
Cell viability curves S3
NMR Spectra S4
High-resolution mass spectroscopy S52

Dose response curves for SHP1 enzyme assay

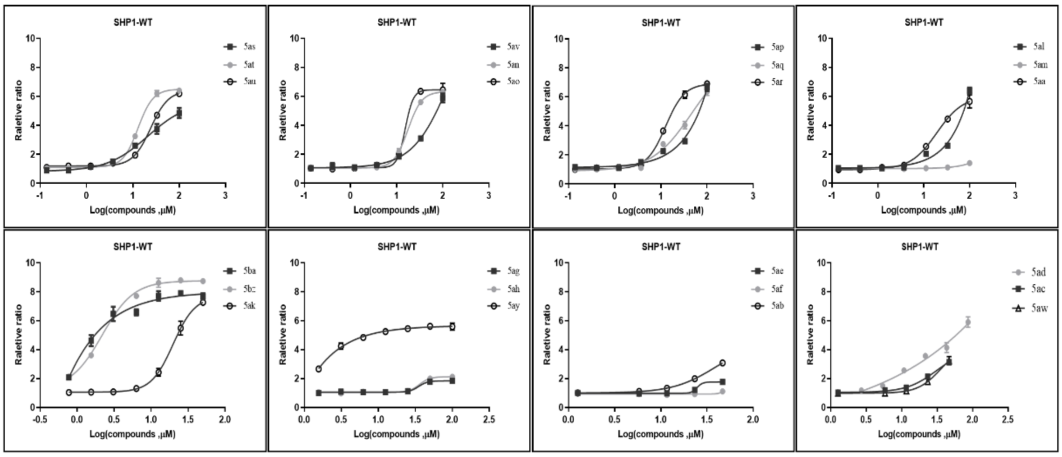

Figure S1. Dose responses of compound **5aa-ba** for SHP1 activation. Data are mean ± S.E.M. (n=3).

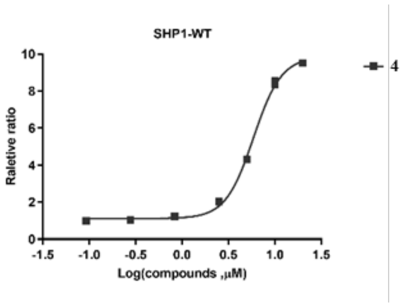

Figure S2. Dose responses of positive control compound **4** for SHP1 activation. Data are mean ± S.E.M. (n=3).

Cell viability curves

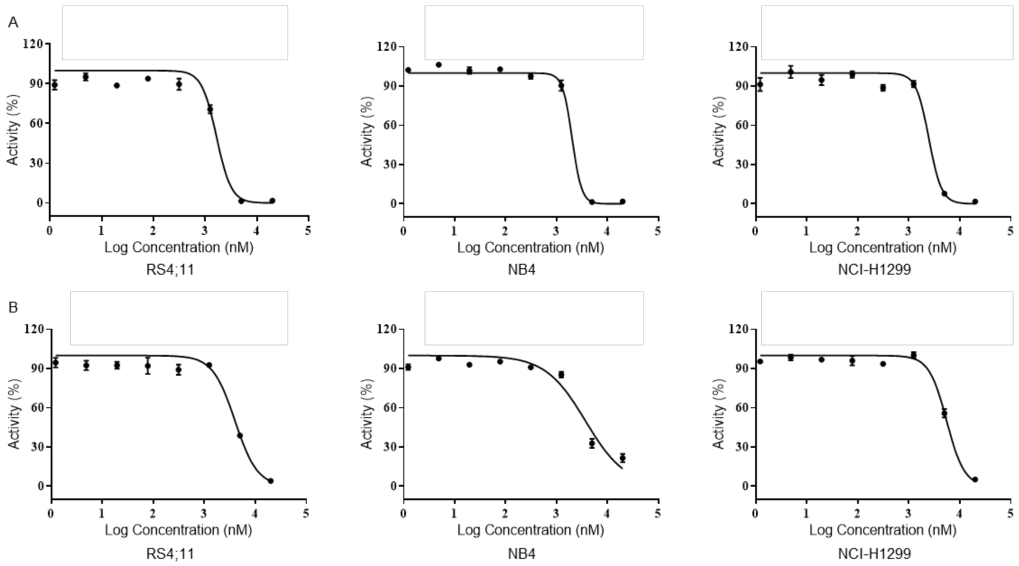

Figure S3. Cytotoxic activity of compound **5az** (A) and **5ba** (B) against RS4;11, NB4, NCI-H1299 cell lines. Data are mean ± S.E.M. (n=3).

NMR Spectra

Compound **7** (^{1}H NMR)

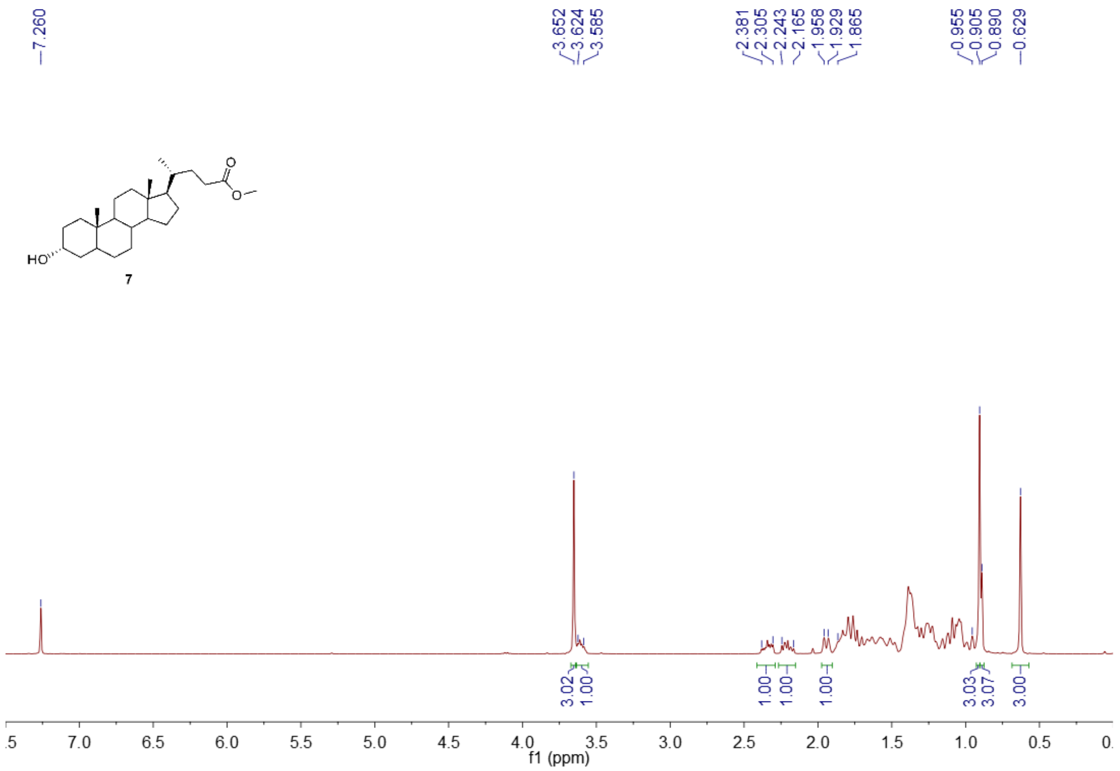

Compound **8** (^{1}H NMR)

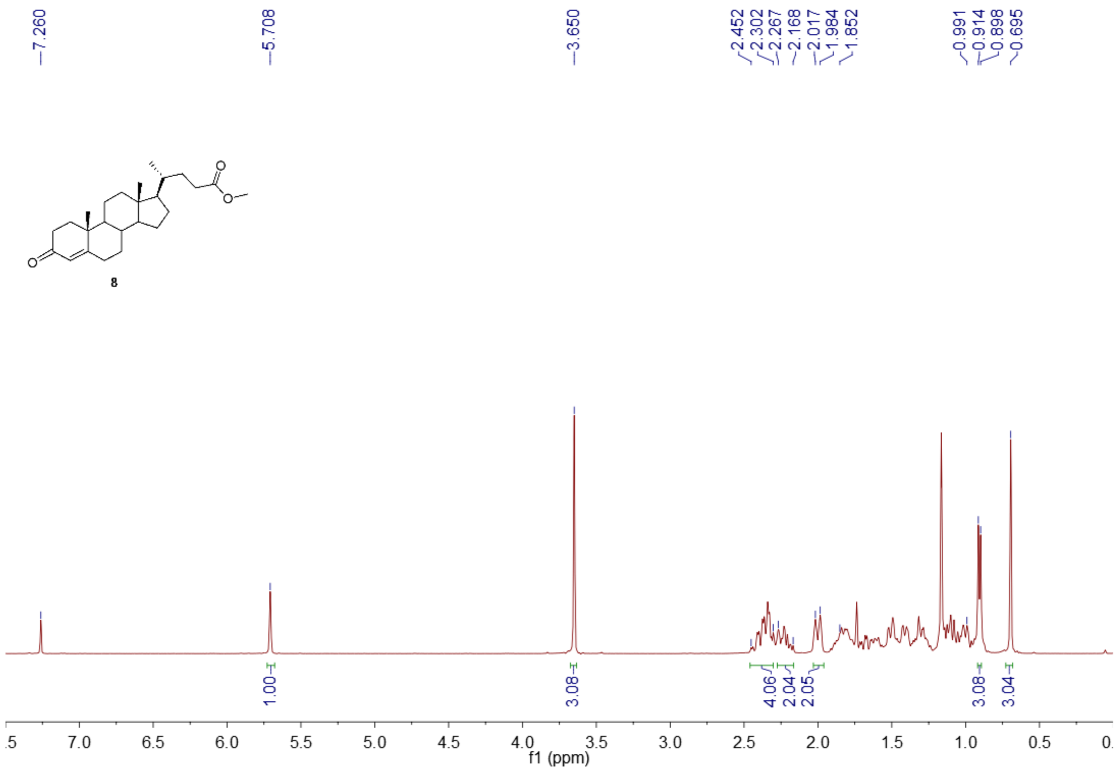

Compound **9** (^{1}H NMR)

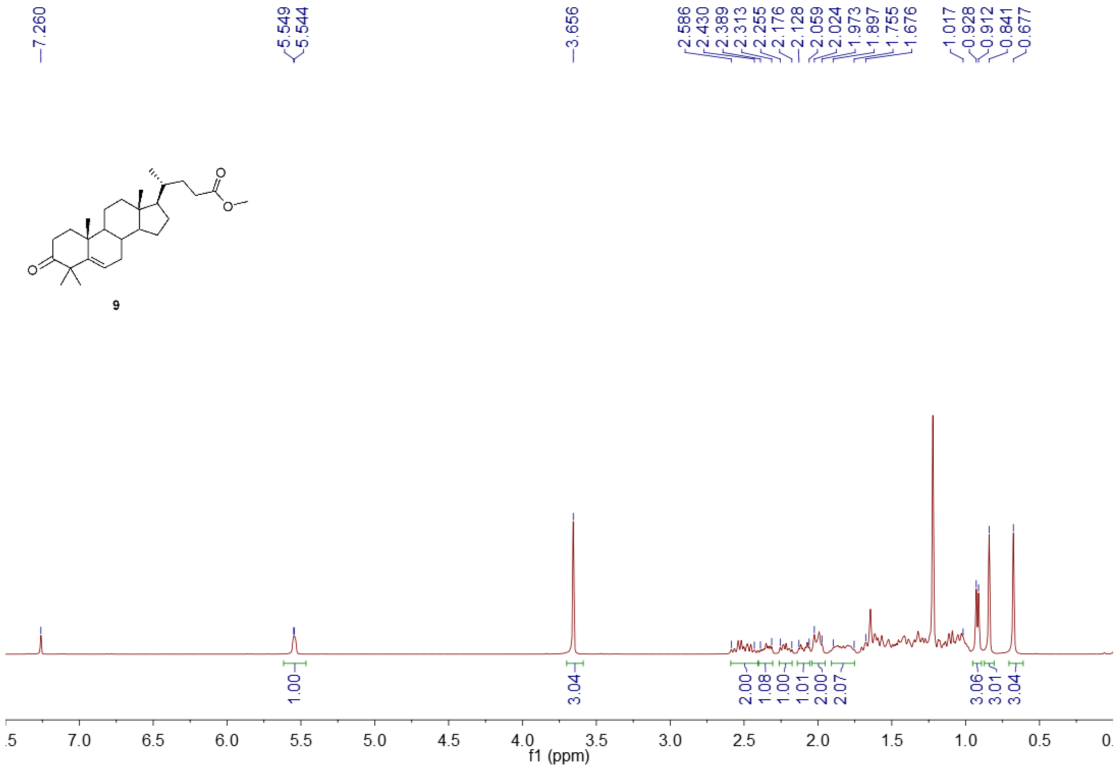

Compound **10** (^{1}H NMR)

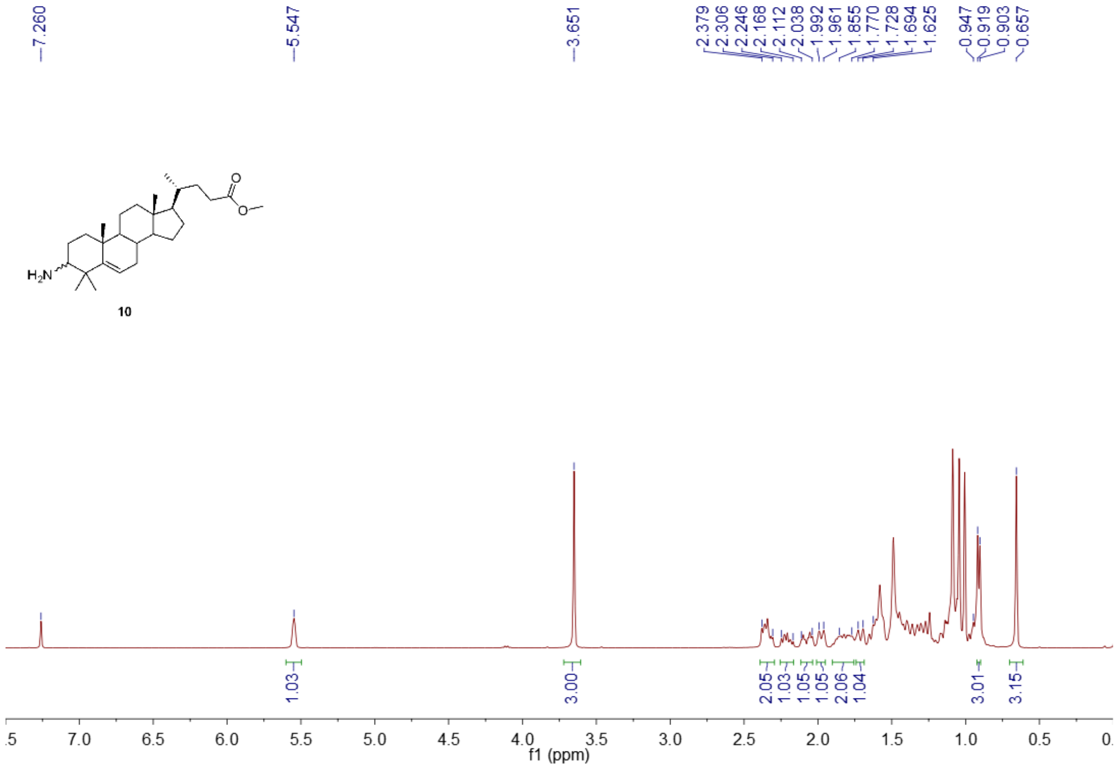

Compound **5aa** (^{1}H NMR)

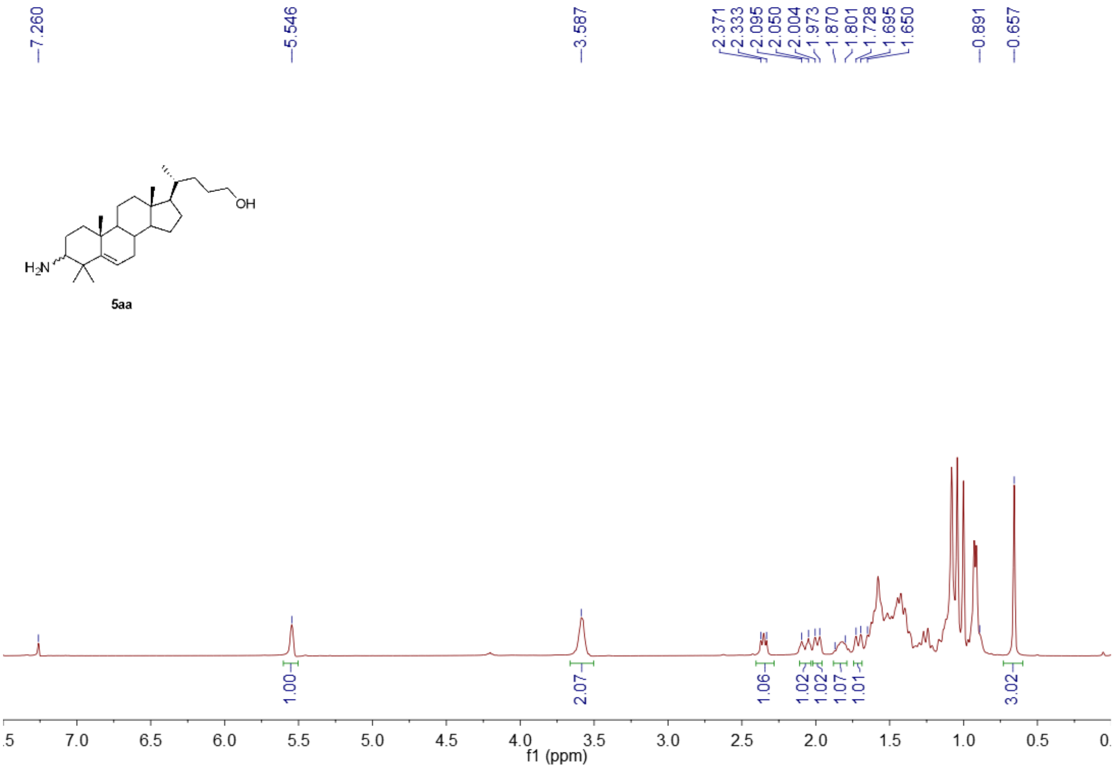

Compound **5aa** (^{13}C NMR)

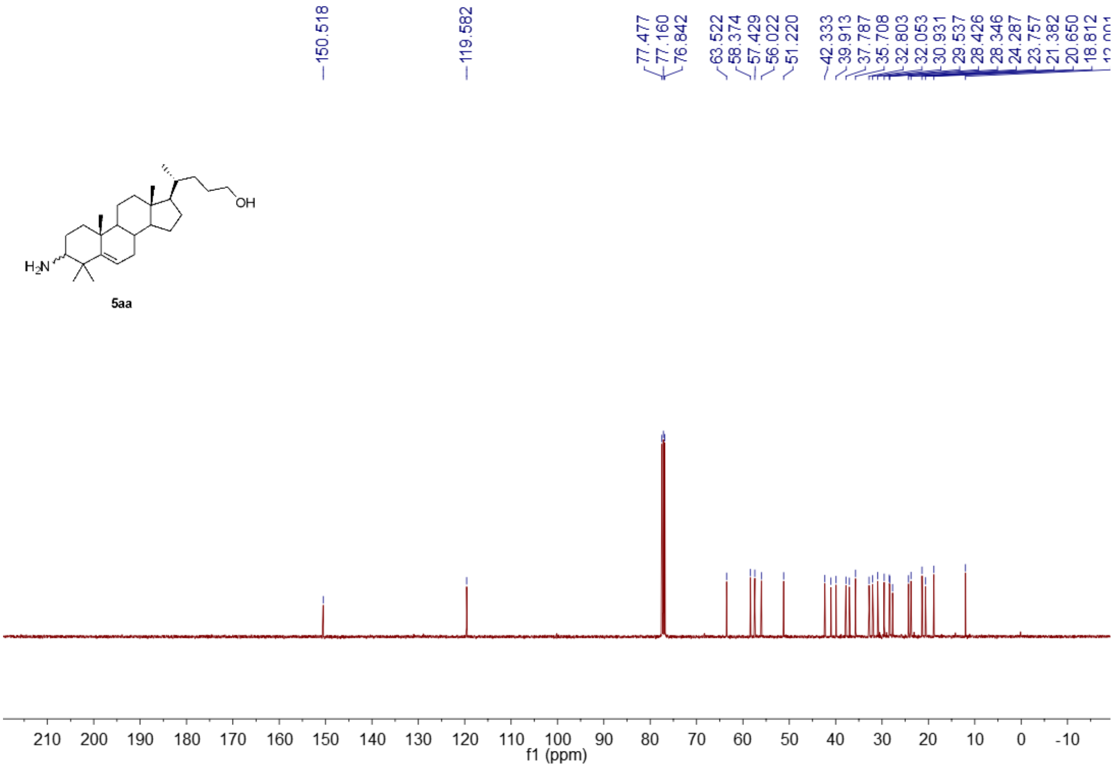

Compound **11a** (^{1}H NMR)

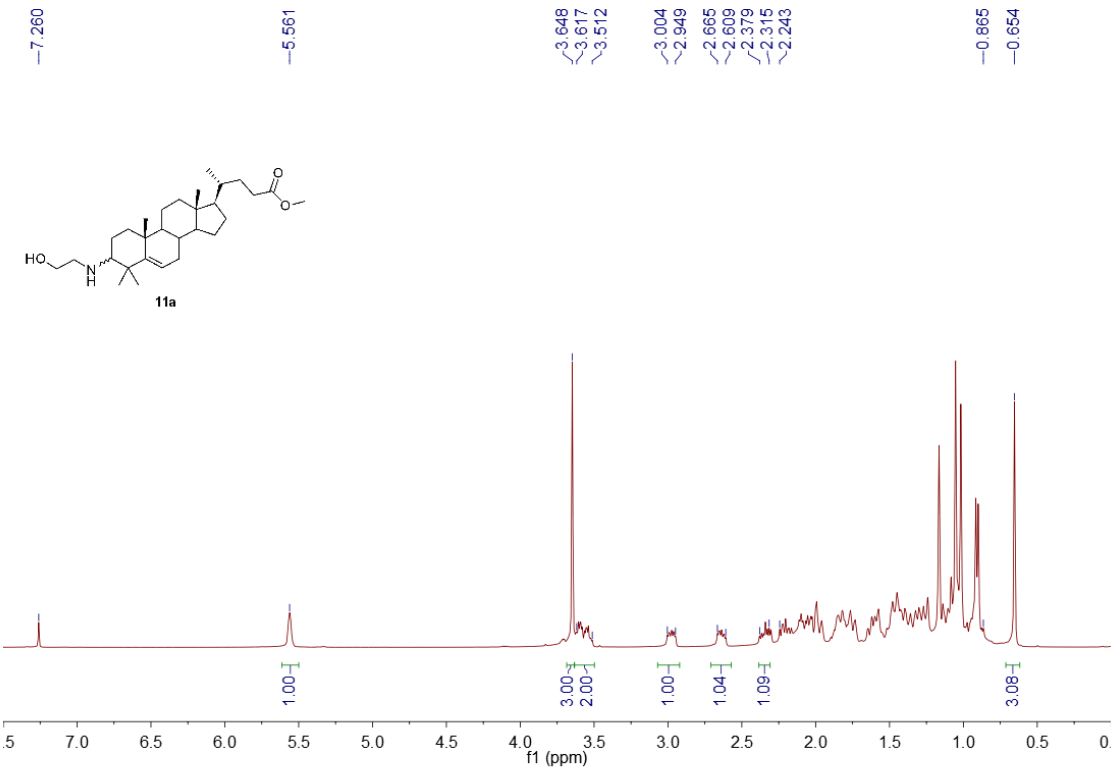

Compound **11b** (^{1}H NMR)

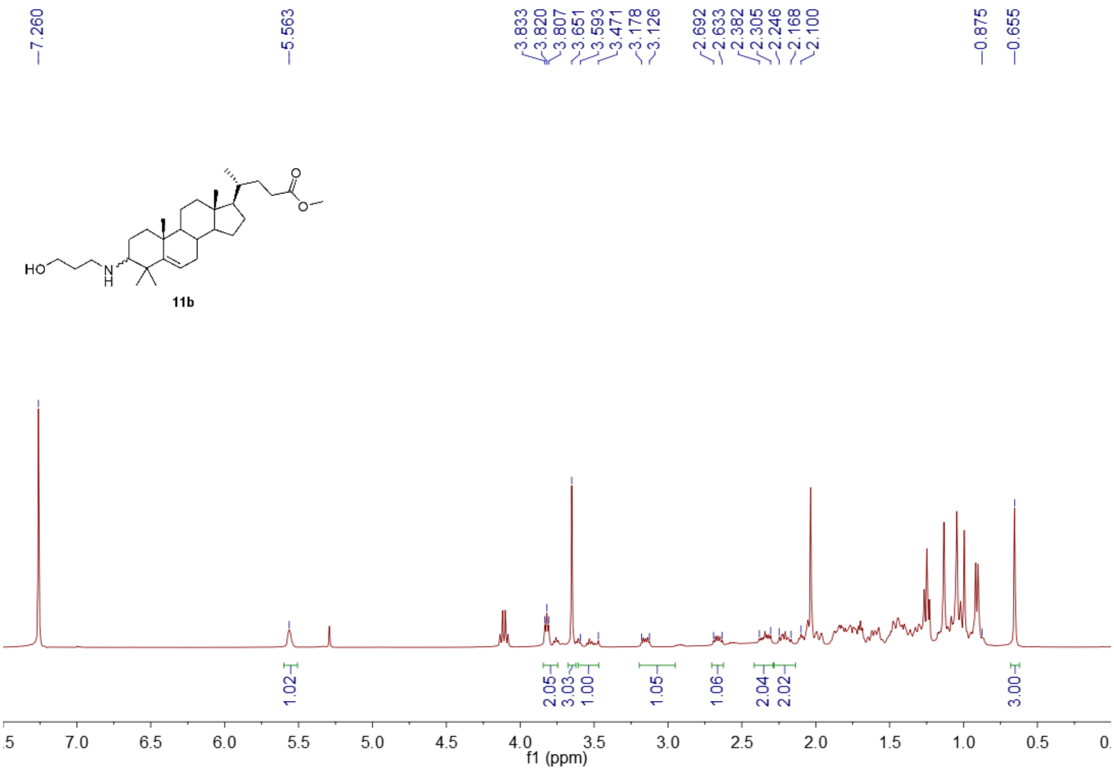

Compound **11c** (^{1}H NMR)

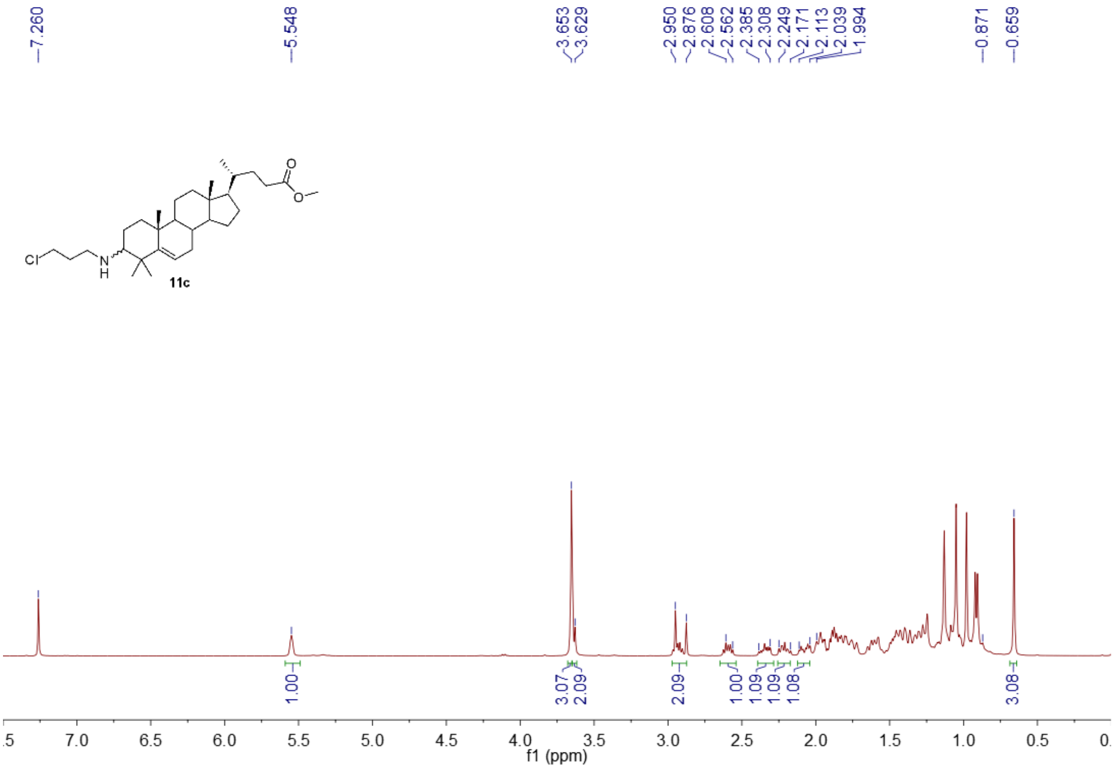

Compound **11d** (^{1}H NMR)

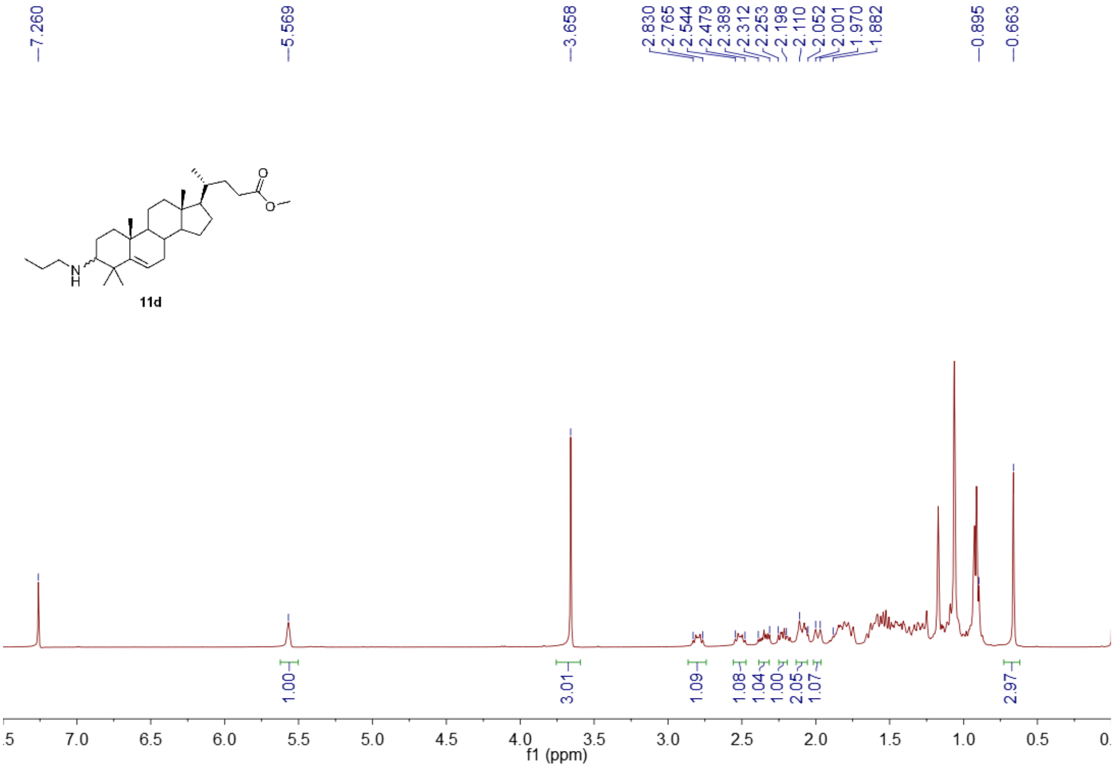

Compound **11e** (^{1}H NMR)

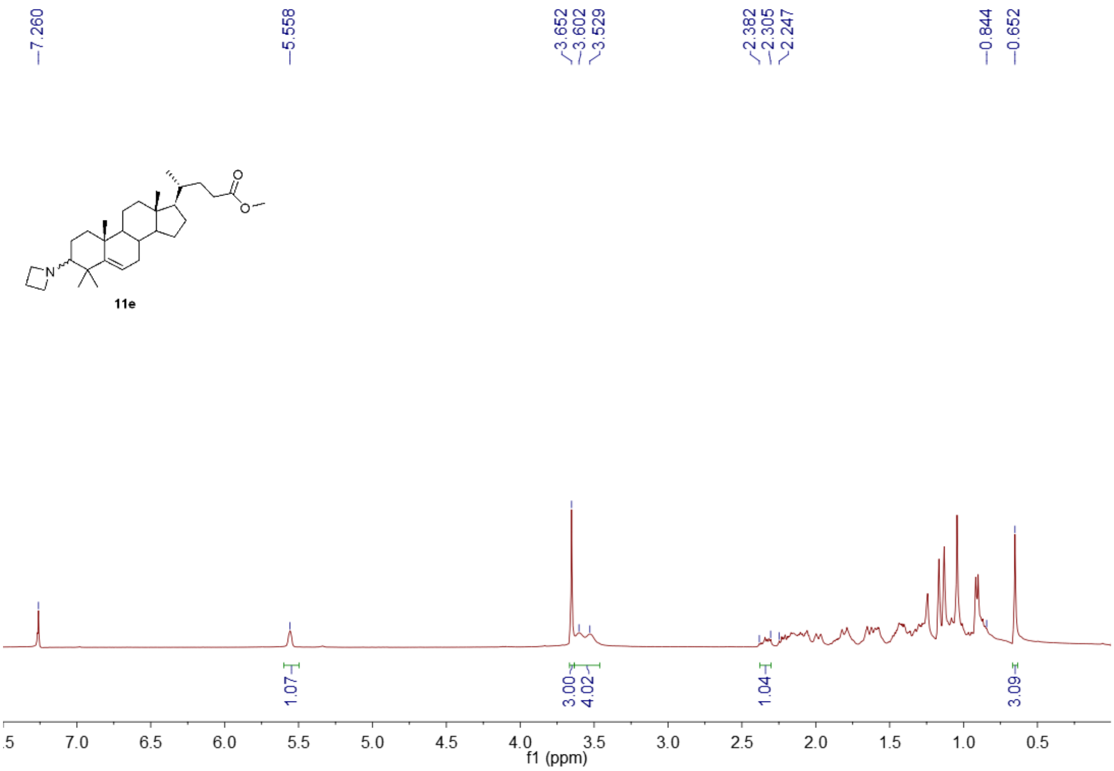

Compound **5ab** (^{1}H NMR)

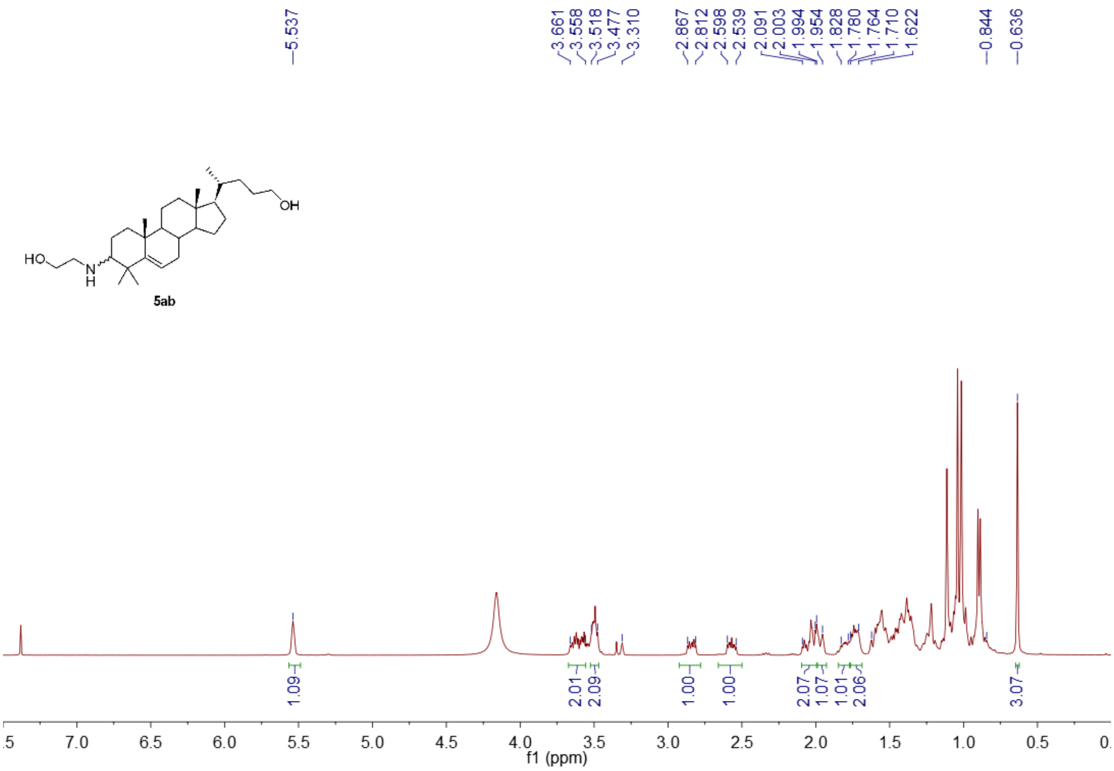

Compound **5ab** (^{13}C NMR)

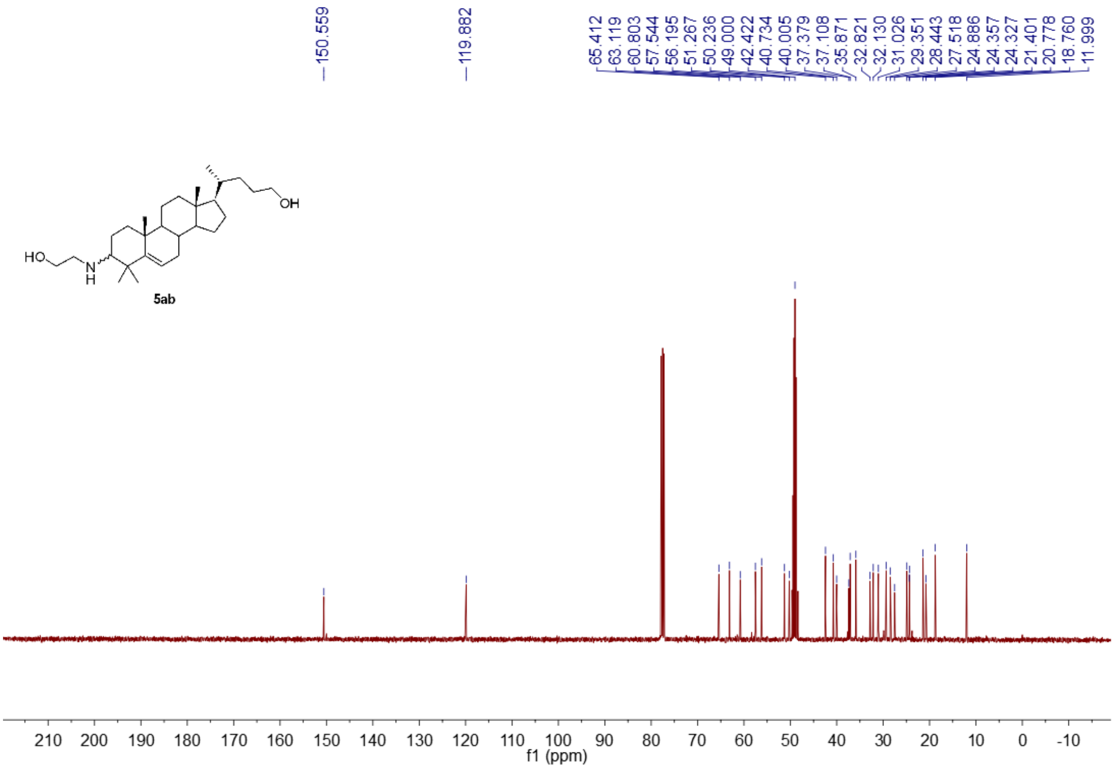

Compound **5ac** (^{1}H NMR)

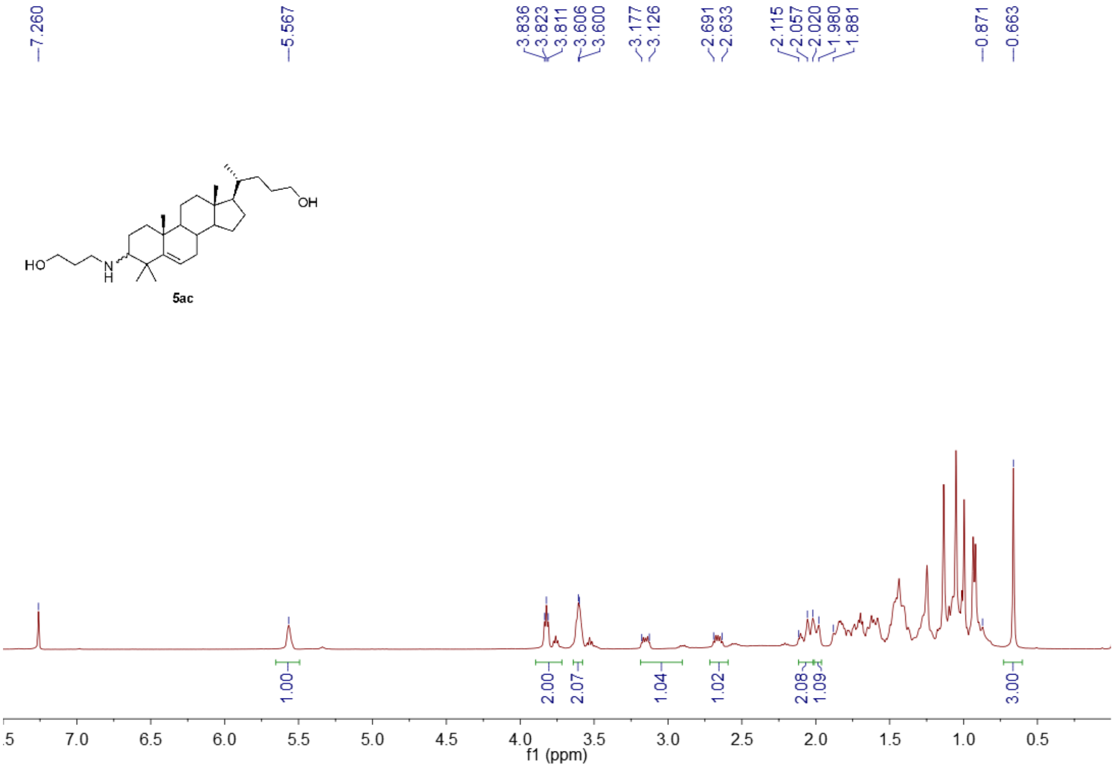

Compound **5ac** (^{13}C NMR)

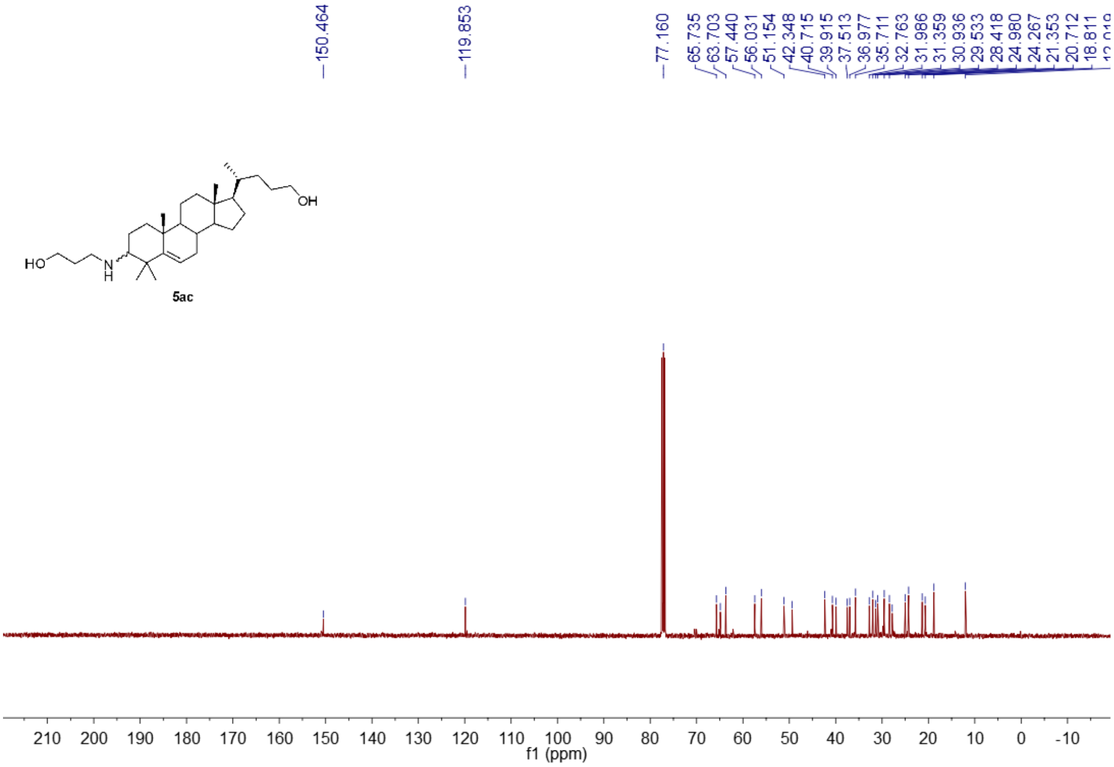

Compound **5ad** (^{1}H NMR)

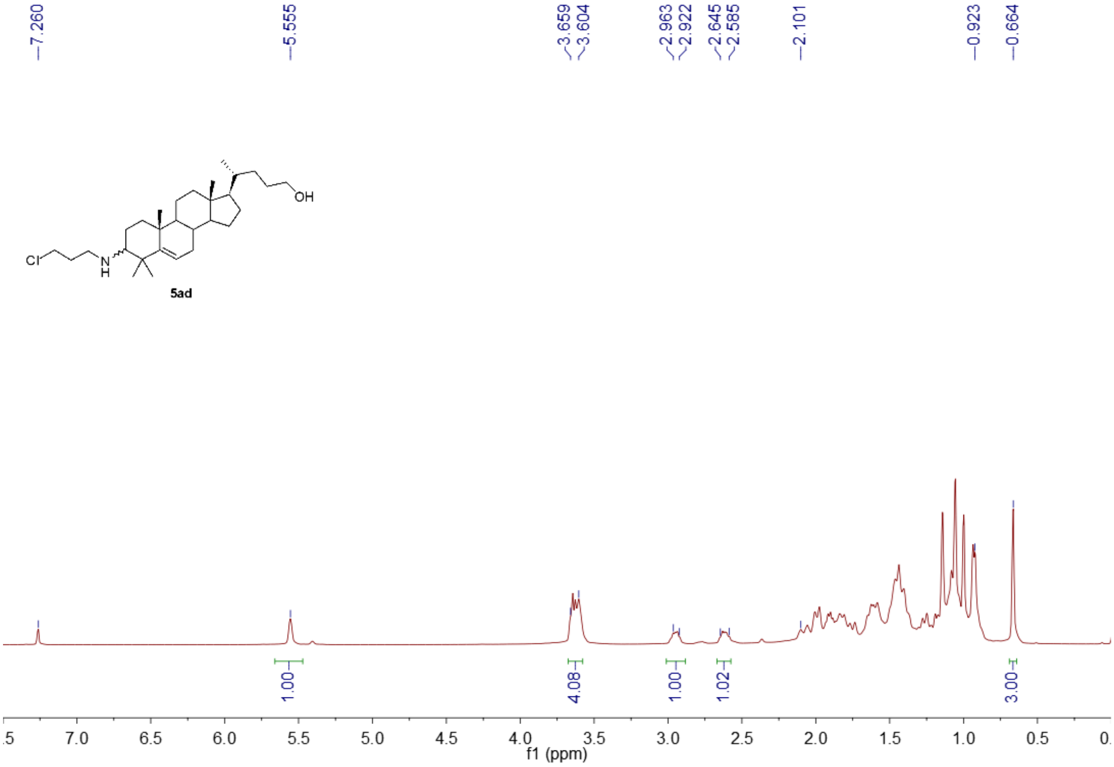

Compound **5ad** (^{13}C NMR)

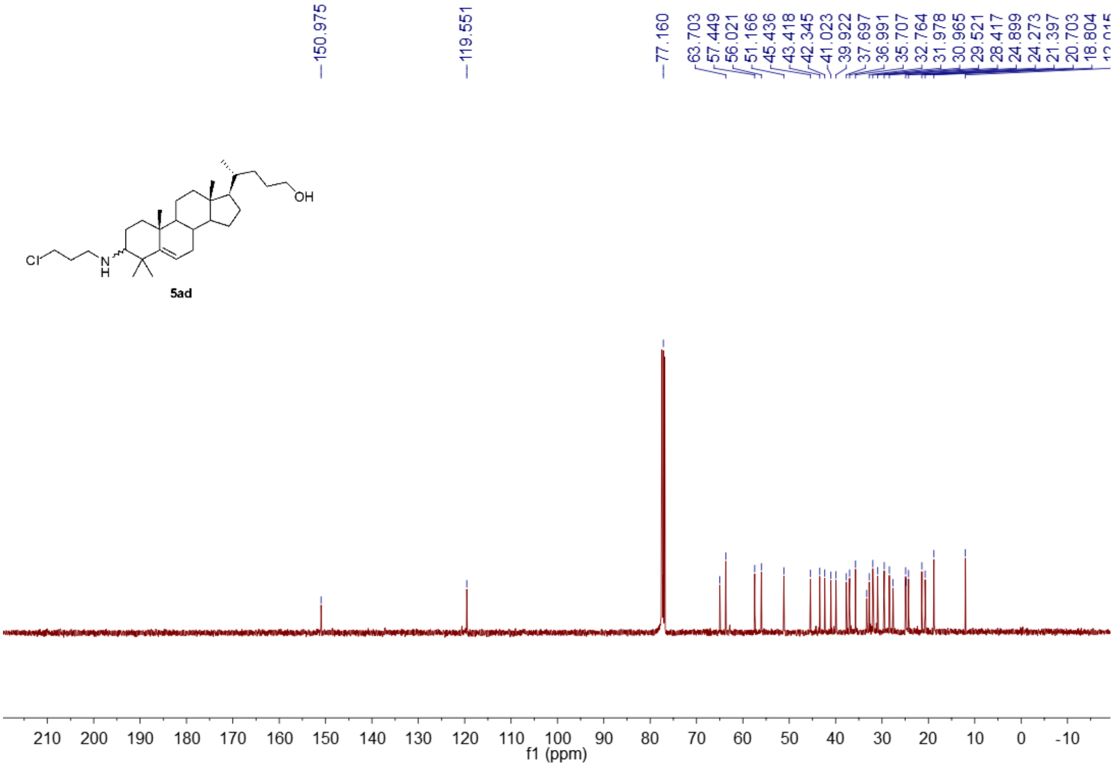

Compound **5ae** (^{1}H NMR)

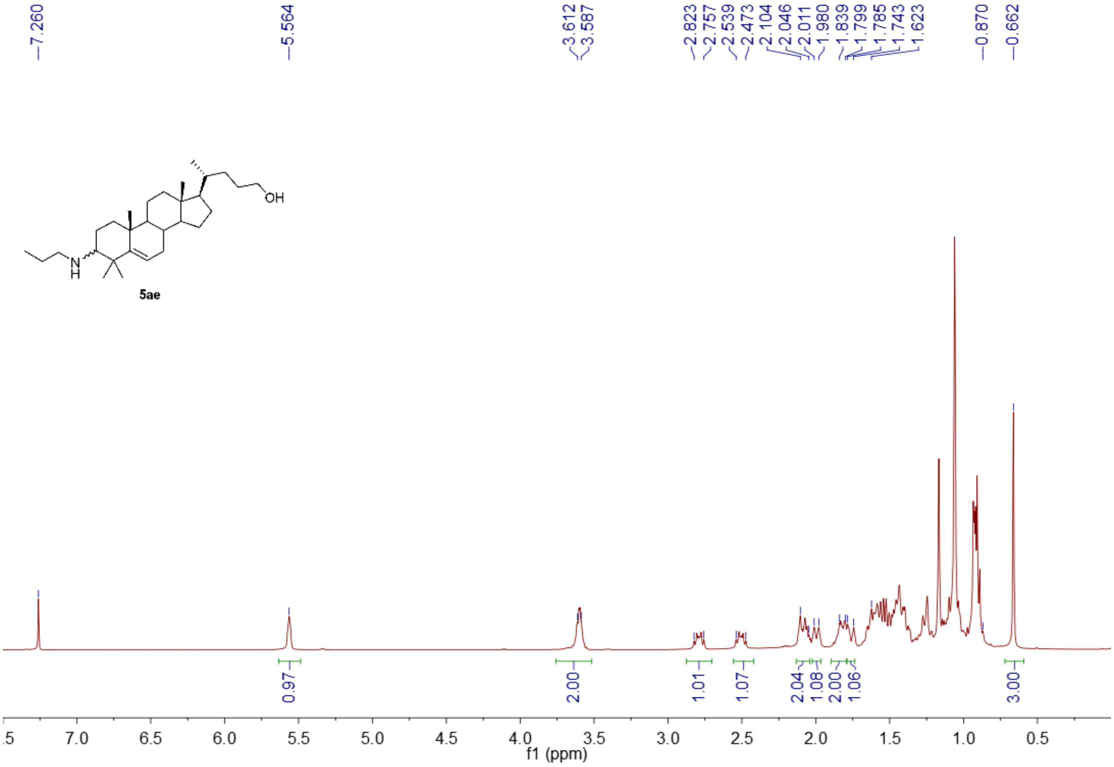

Compound **5ae** (^{13}C NMR)

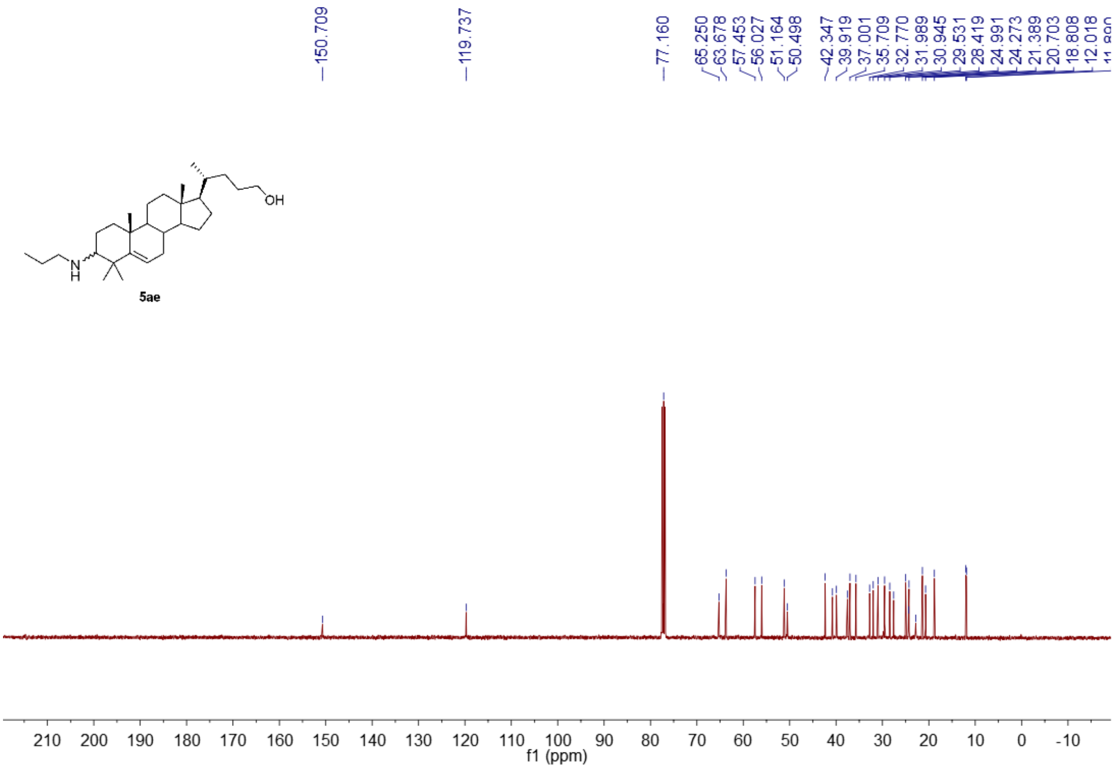

Compound **5af** (^{1}H NMR)

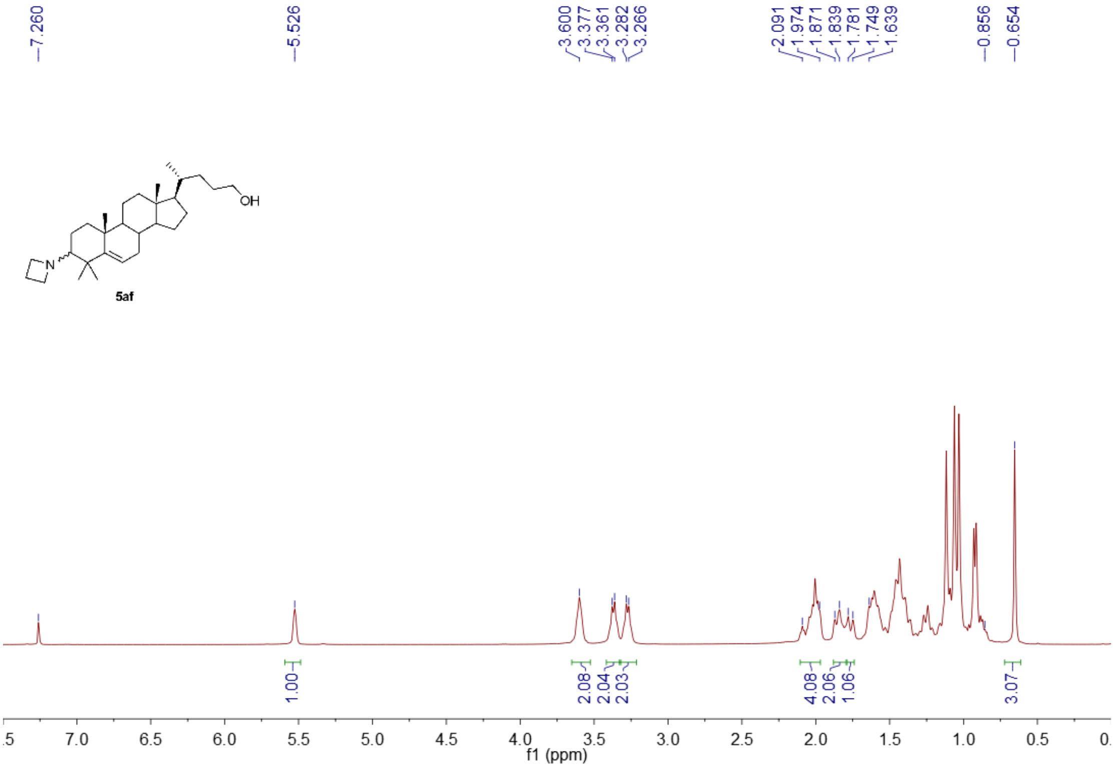

Compound **5af** (^{13}C NMR)

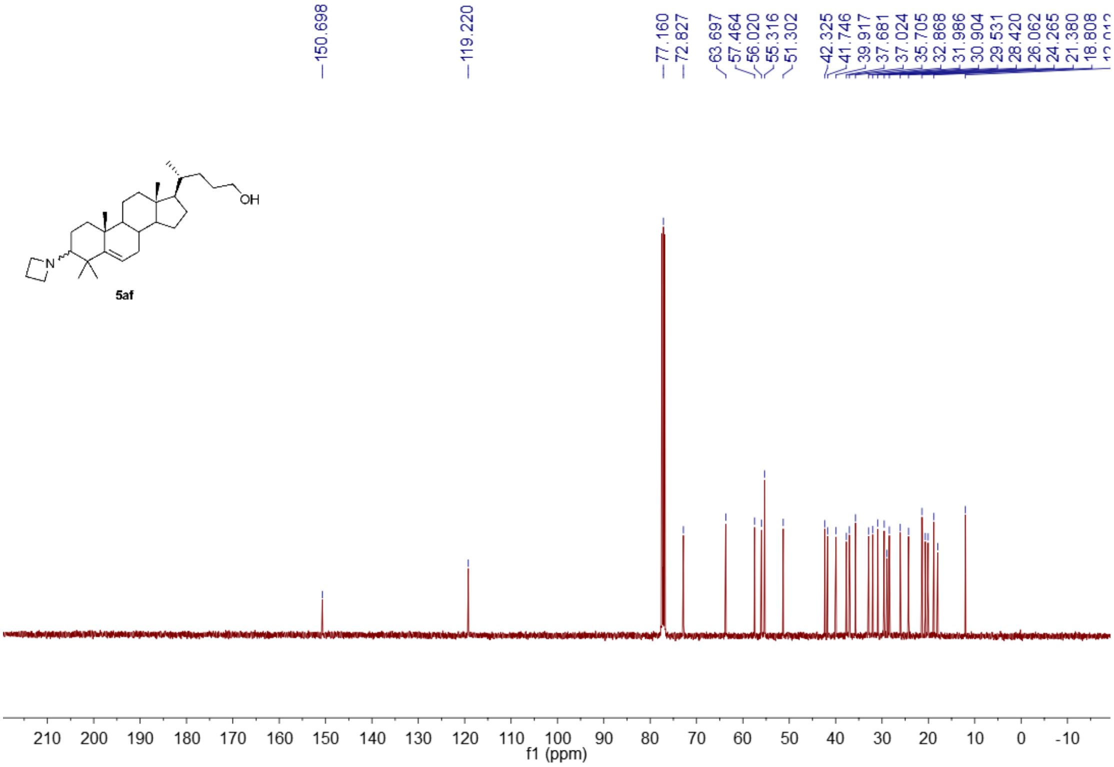

Compound **12** (^{1}H NMR)

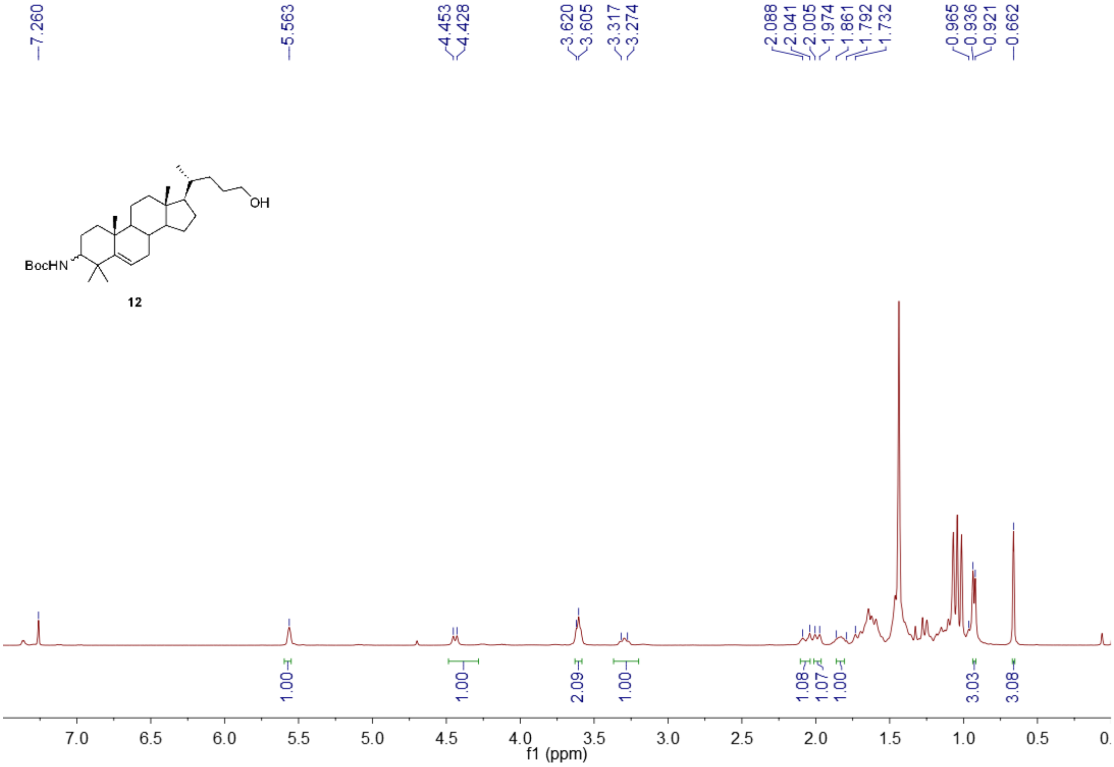

Compound **13** (^{1}H NMR)

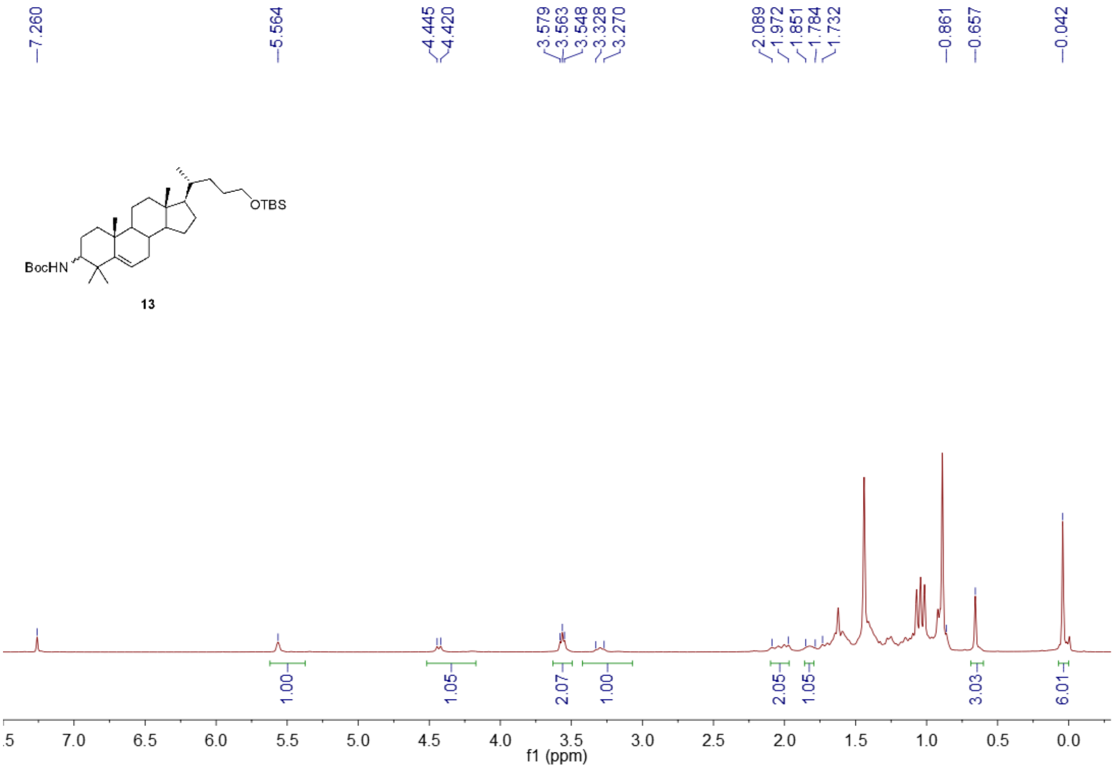

Compound **14** (^{1}H NMR)

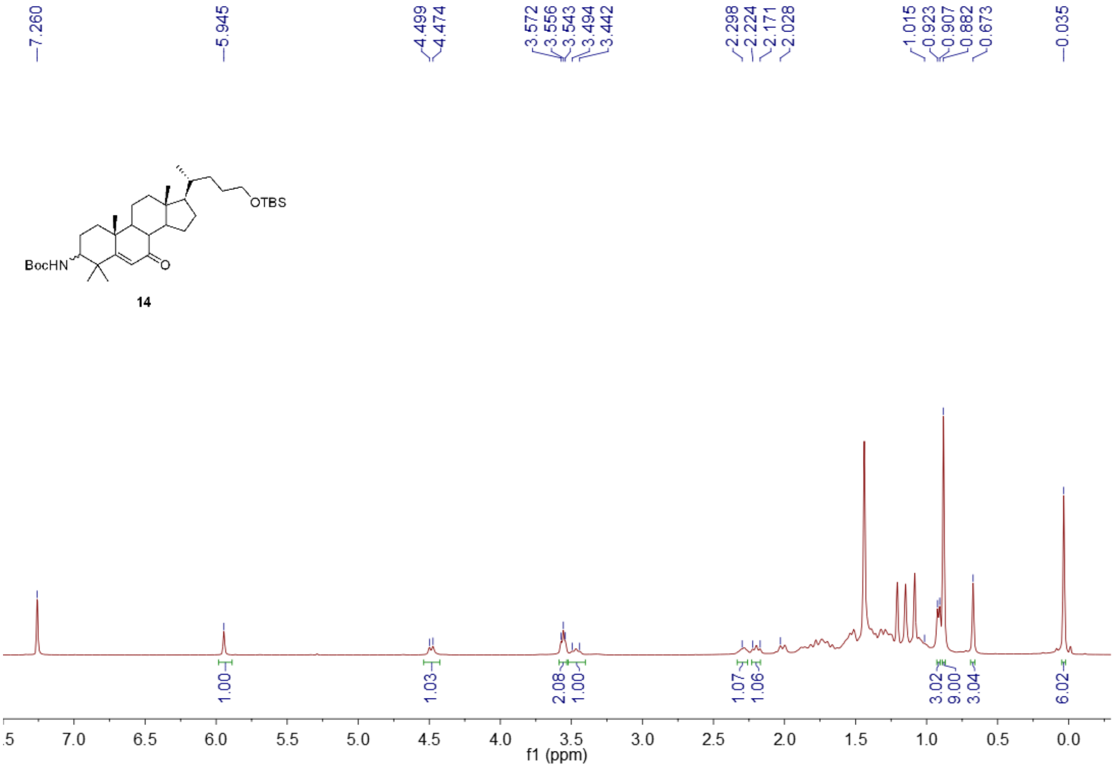

Compound **5ag** (^{1}H NMR)

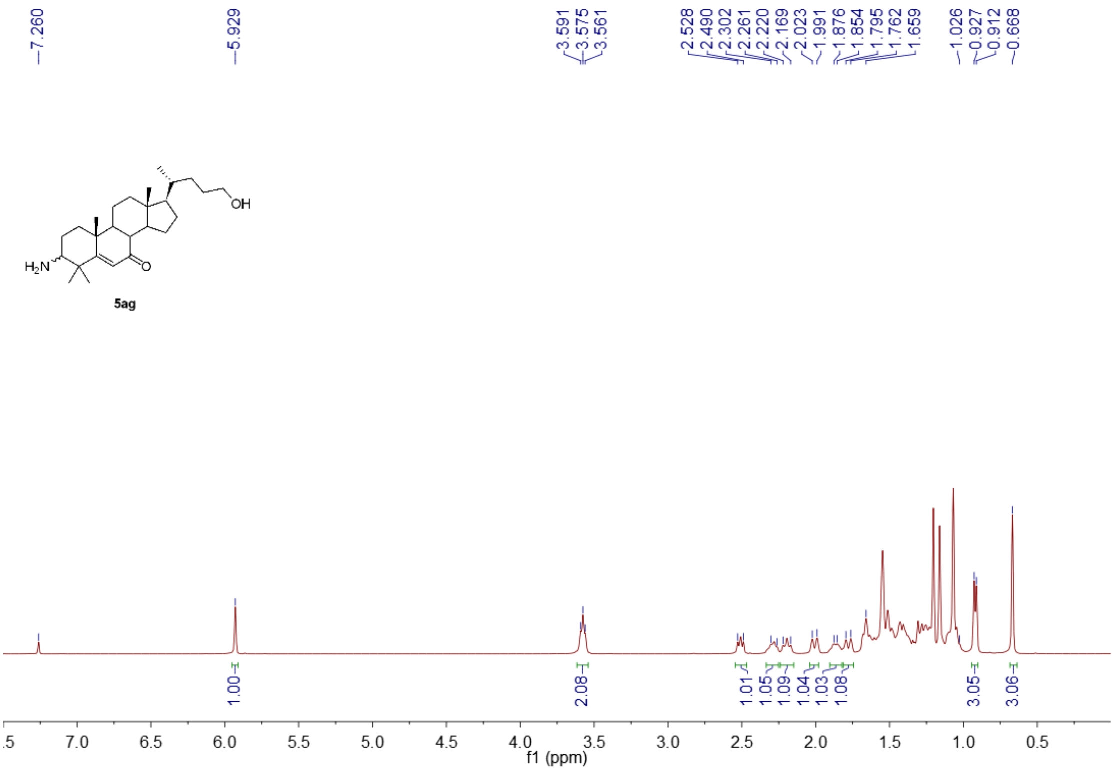

Compound **5ag** (^{13}C NMR)

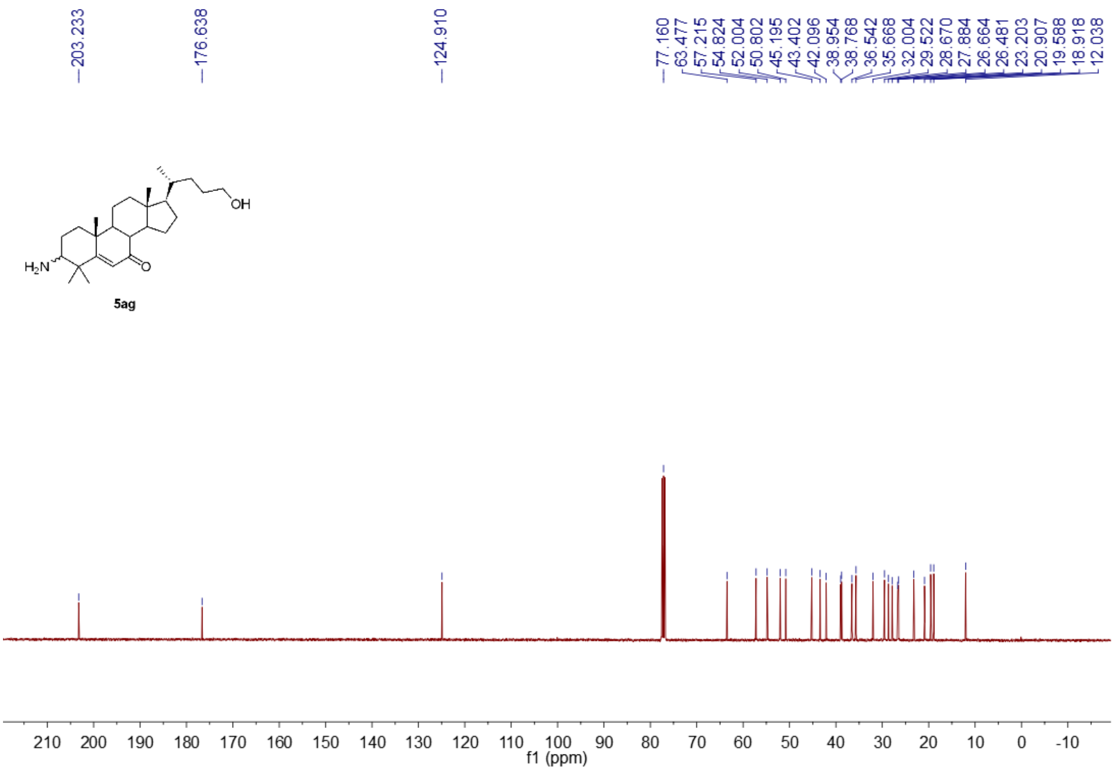

Compound **5ah** (^{1}H NMR)

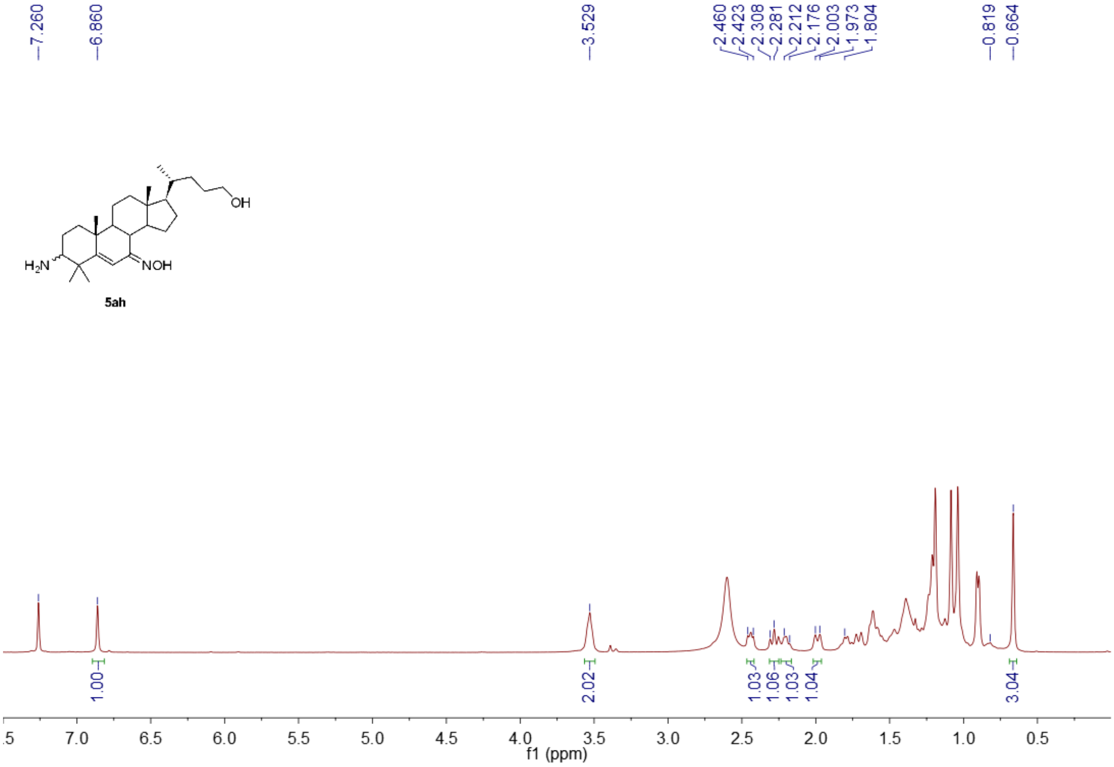

Compound **5ah** (^{13}C NMR)

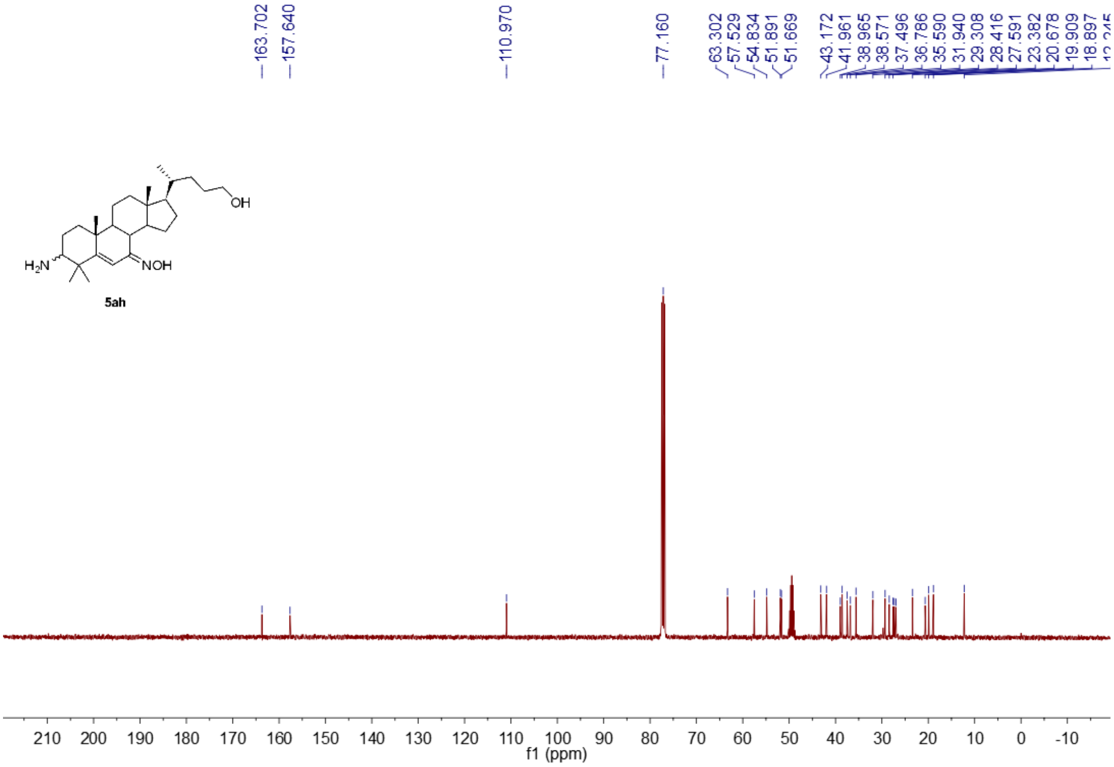

Compound **5ai** (^{1}H NMR)

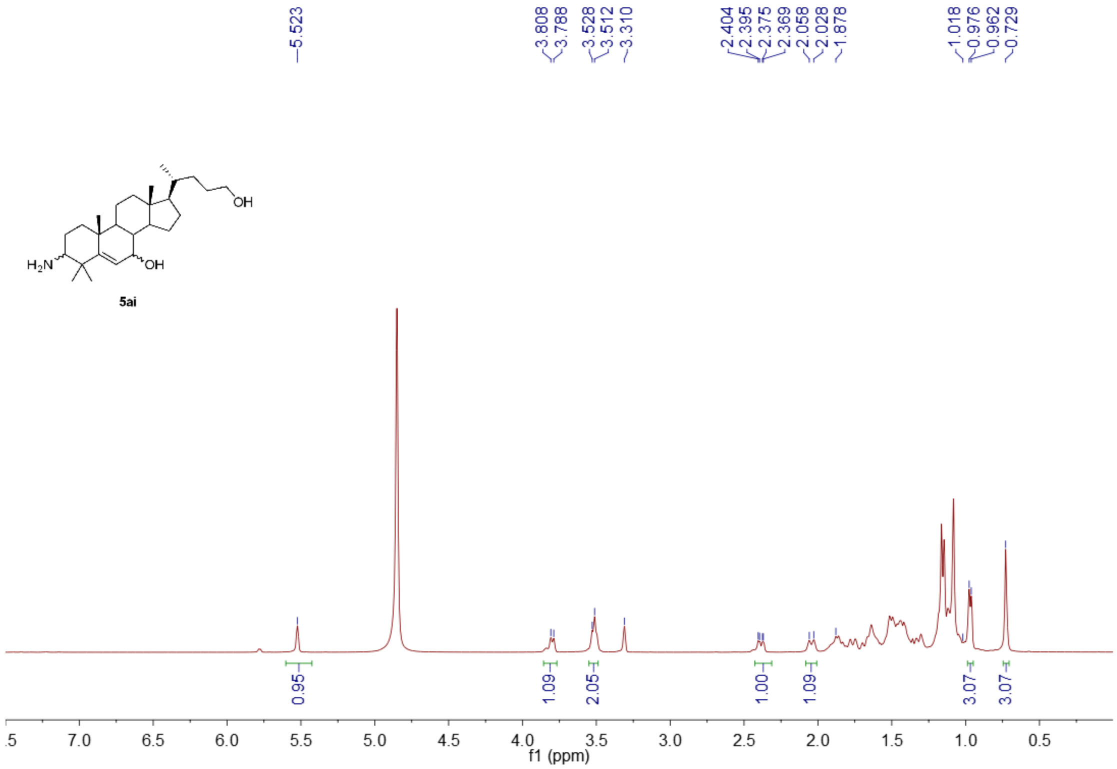

Compound **5ai** (^{13}C NMR)

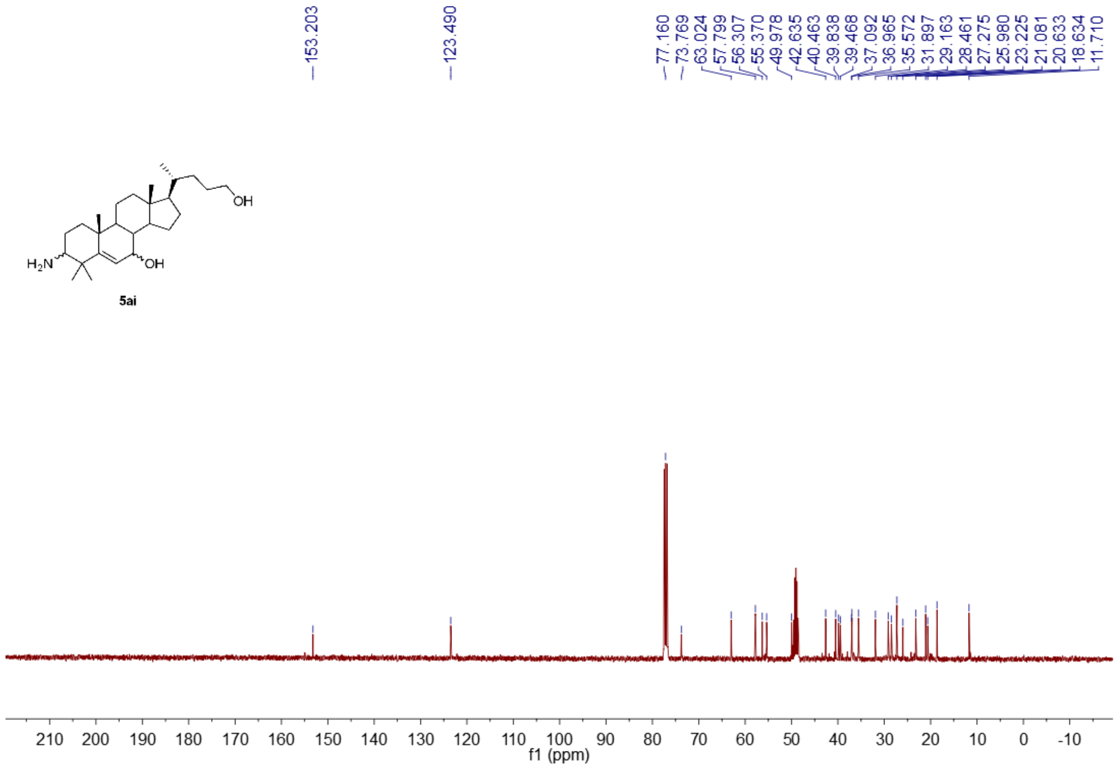

Compound **15** (^{1}H NMR)

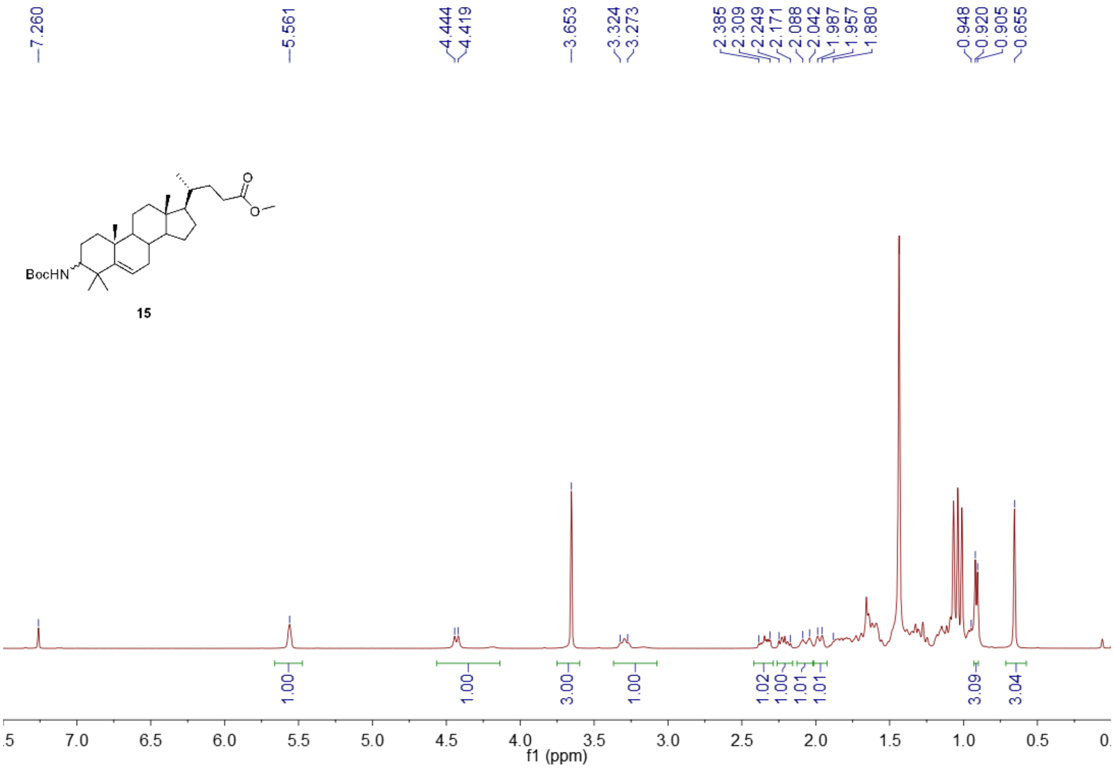

Compound **16** (^{1}H NMR)

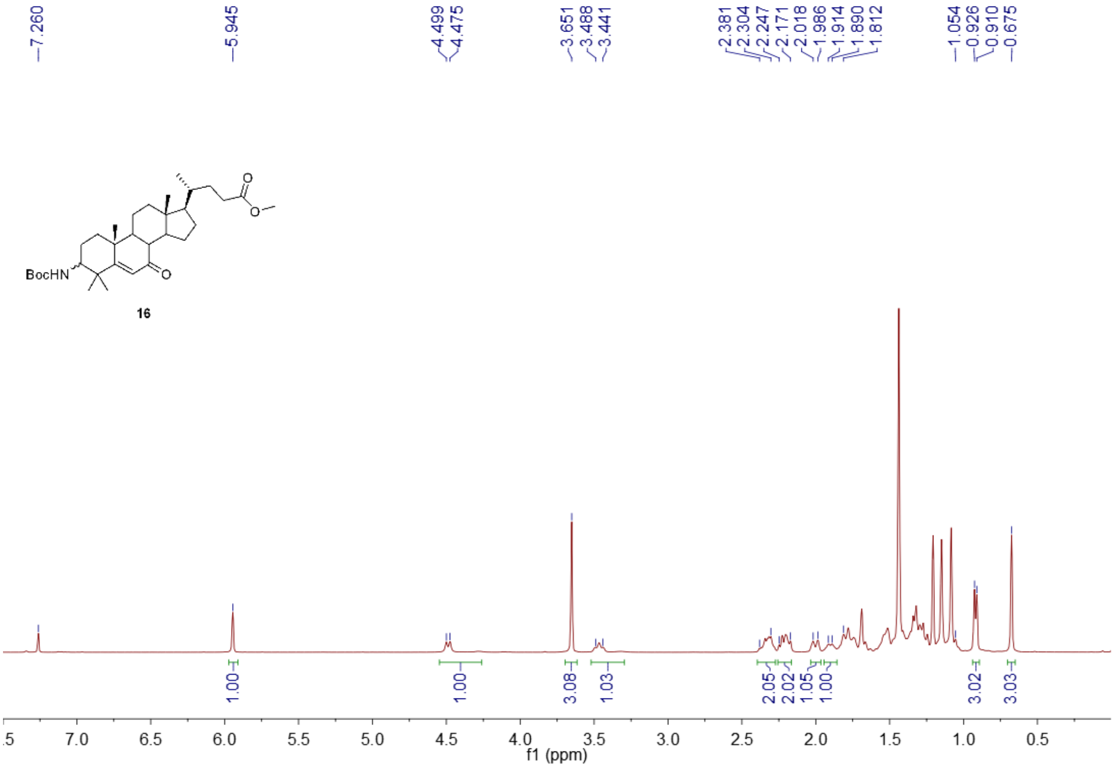

Compound **18** (^{1}H NMR)

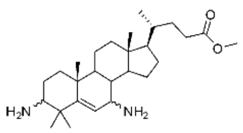

18

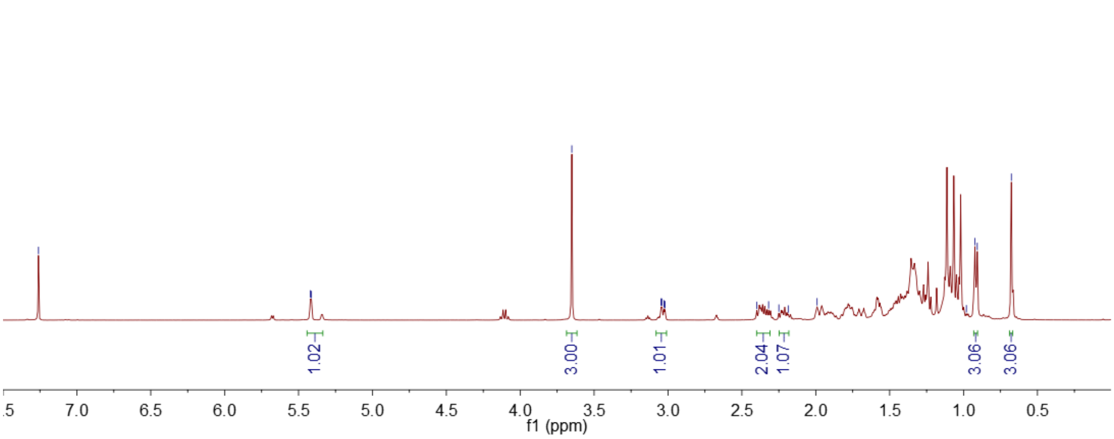

Compound **5aj** (^{1}H NMR)

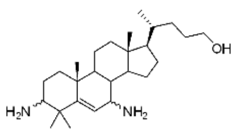

5aj

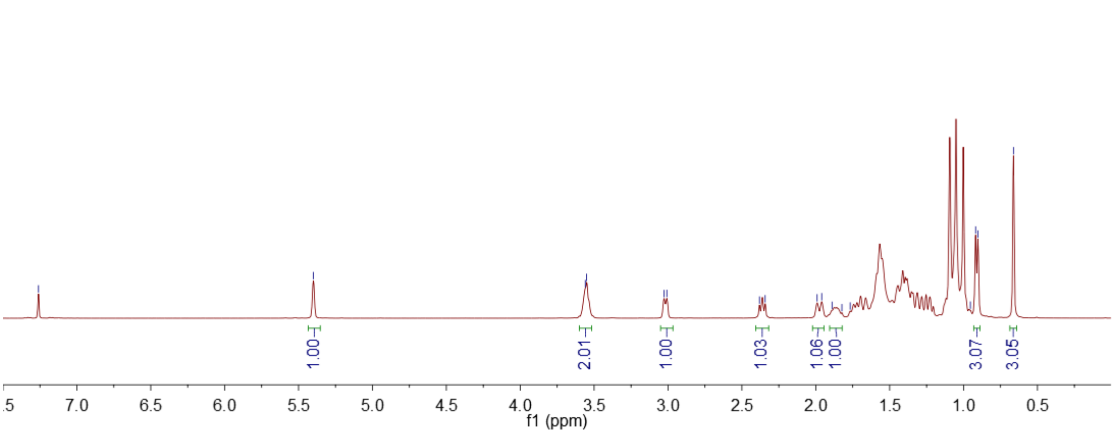

Compound **5aj** (^{13}C NMR)

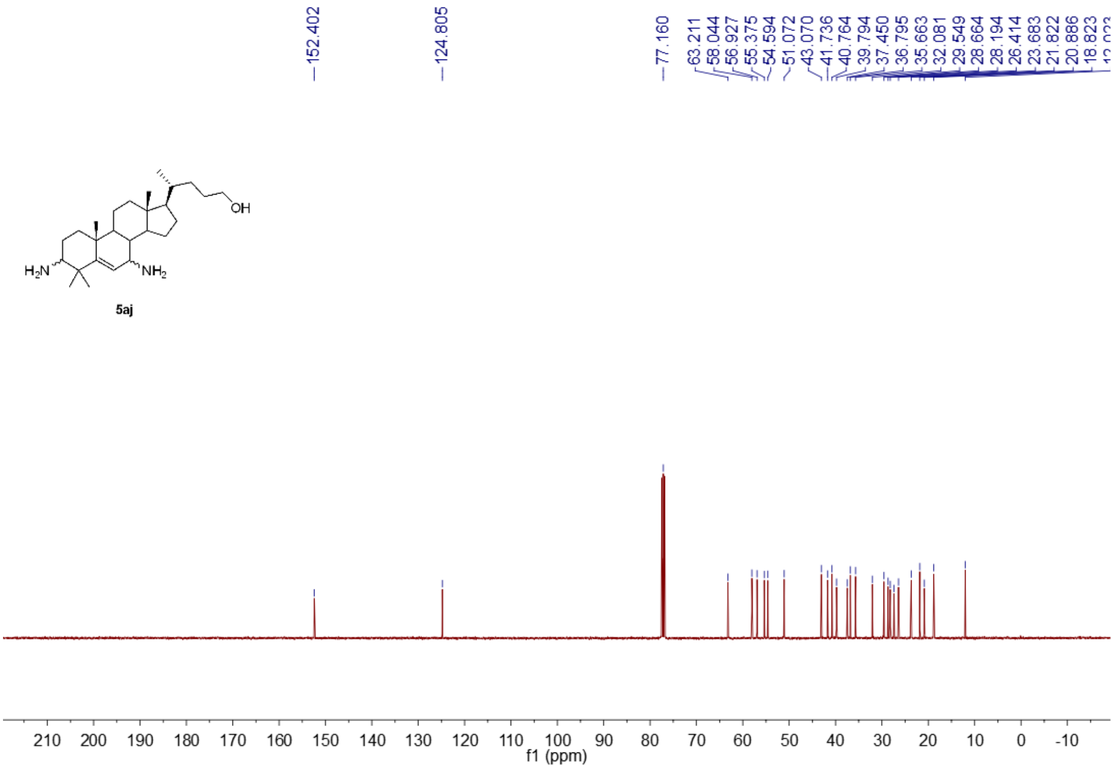

Compound **19** (^{1}H NMR)

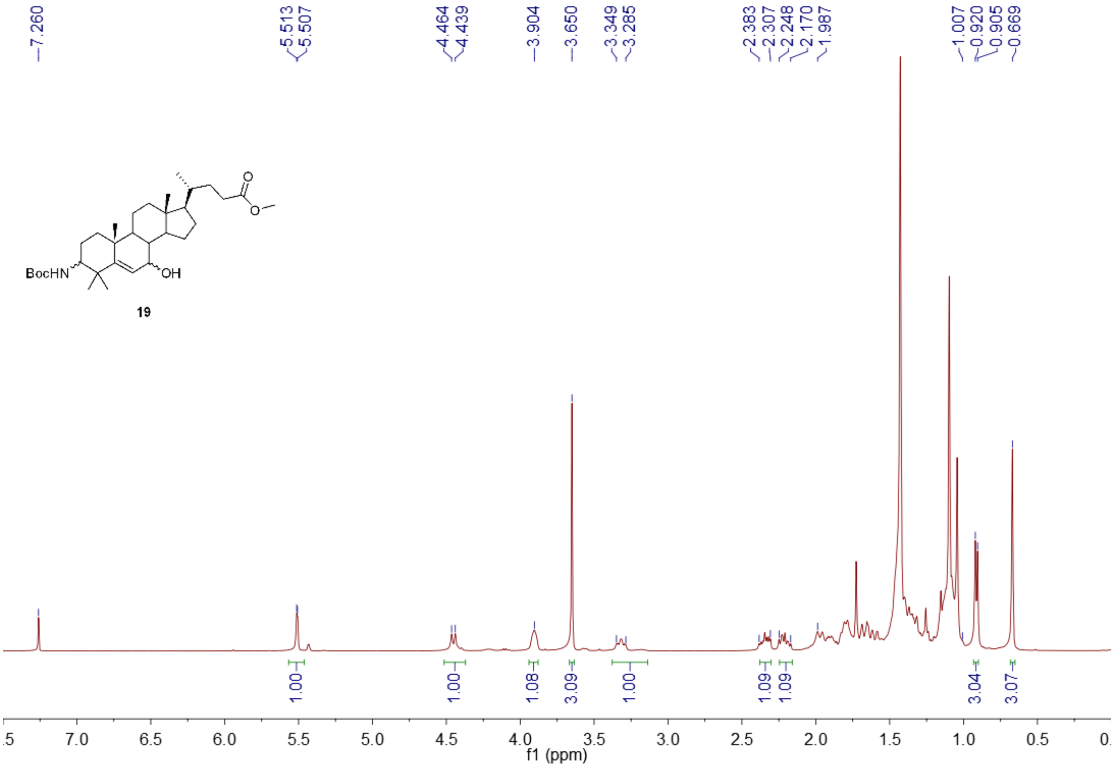

Compound **5ak** (^{1}H NMR)

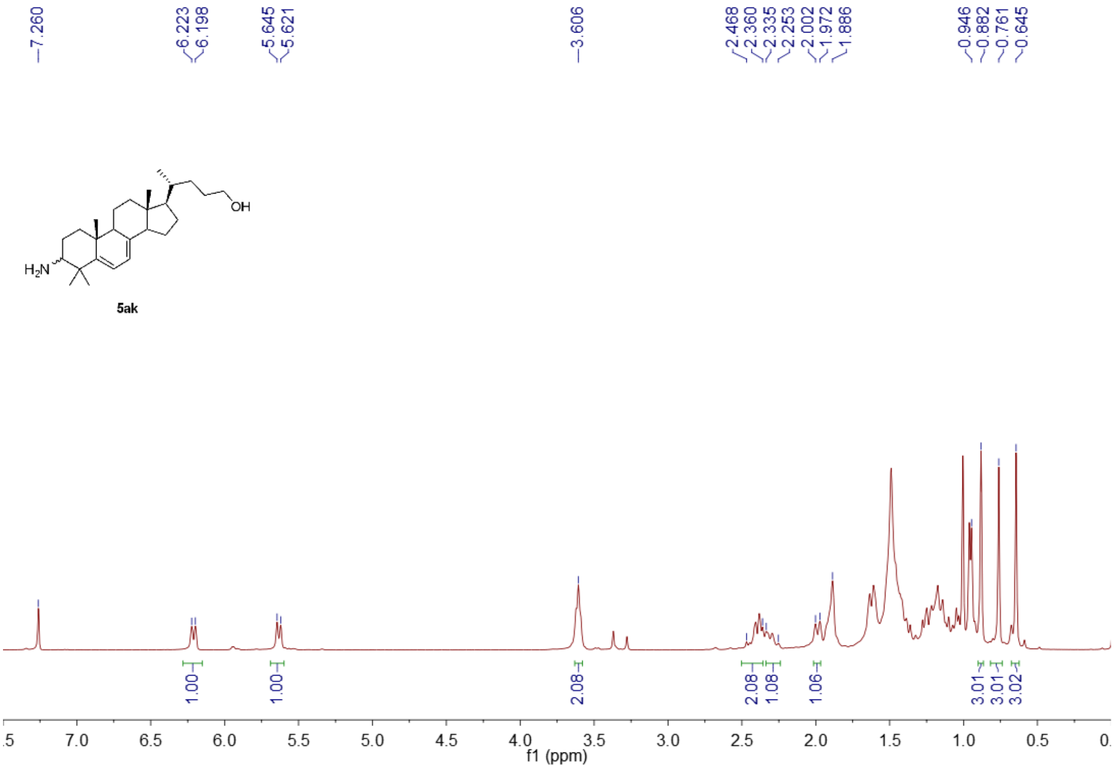

Compound **5ak** (^{13}C NMR)

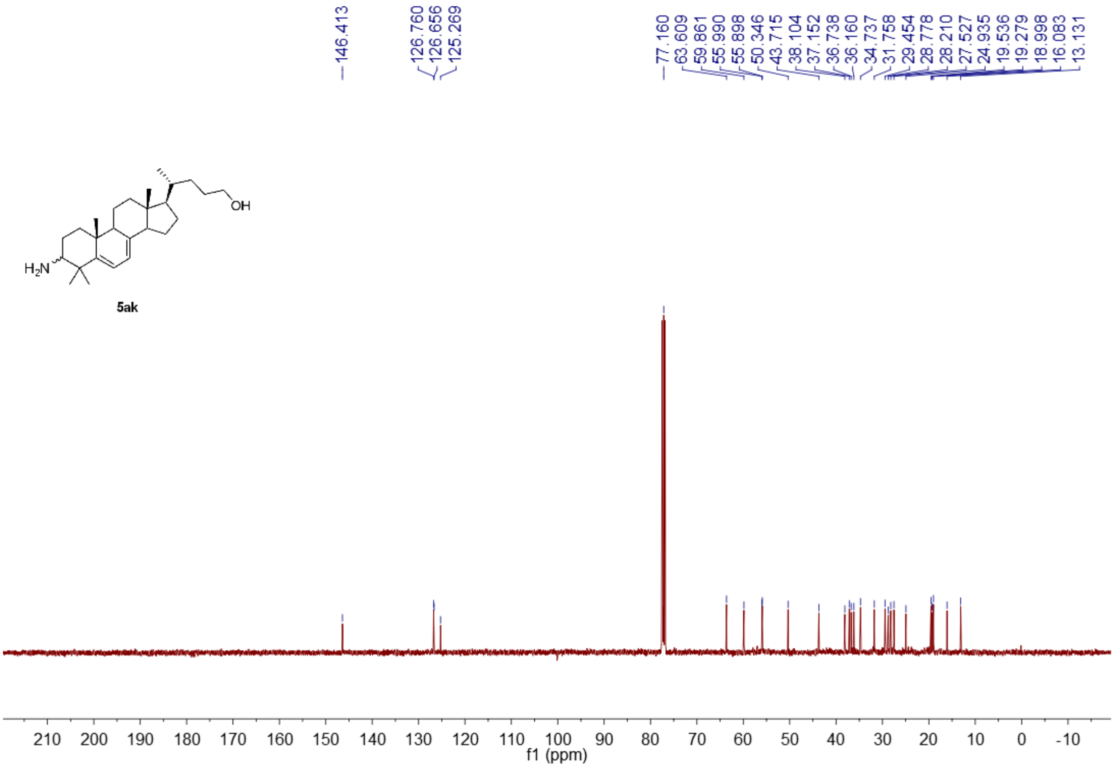

Compound **22** (^{1}H NMR)

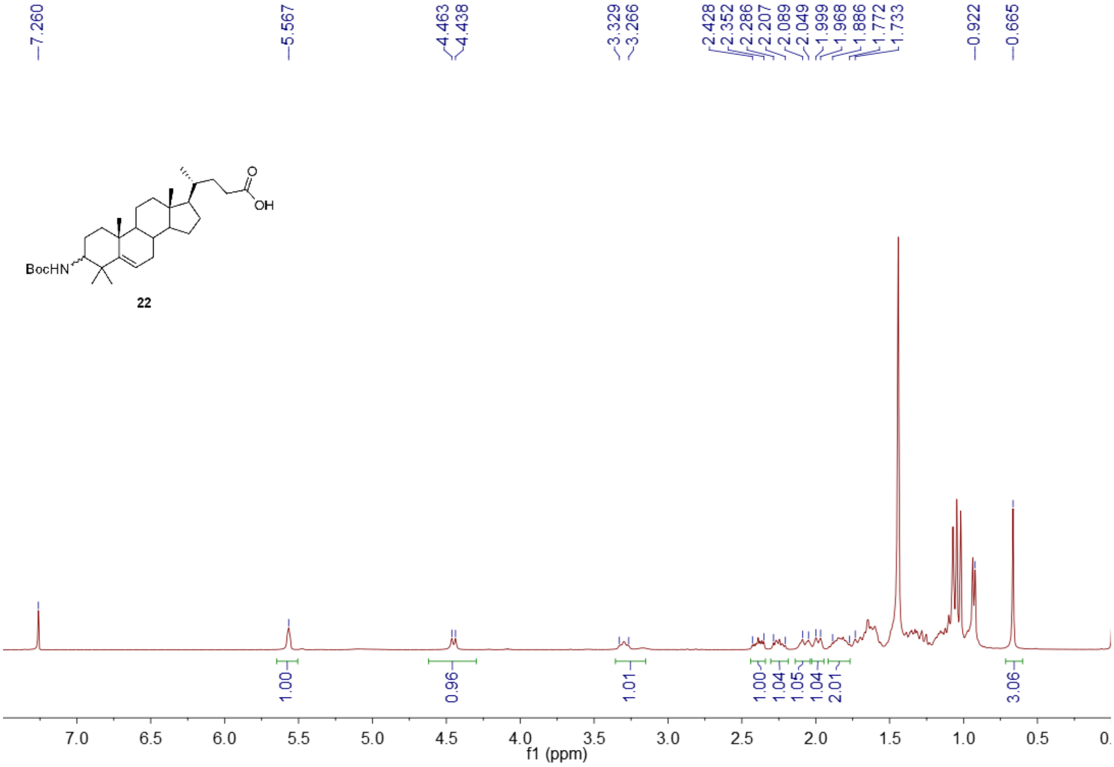

Compound **23** (^{1}H NMR)

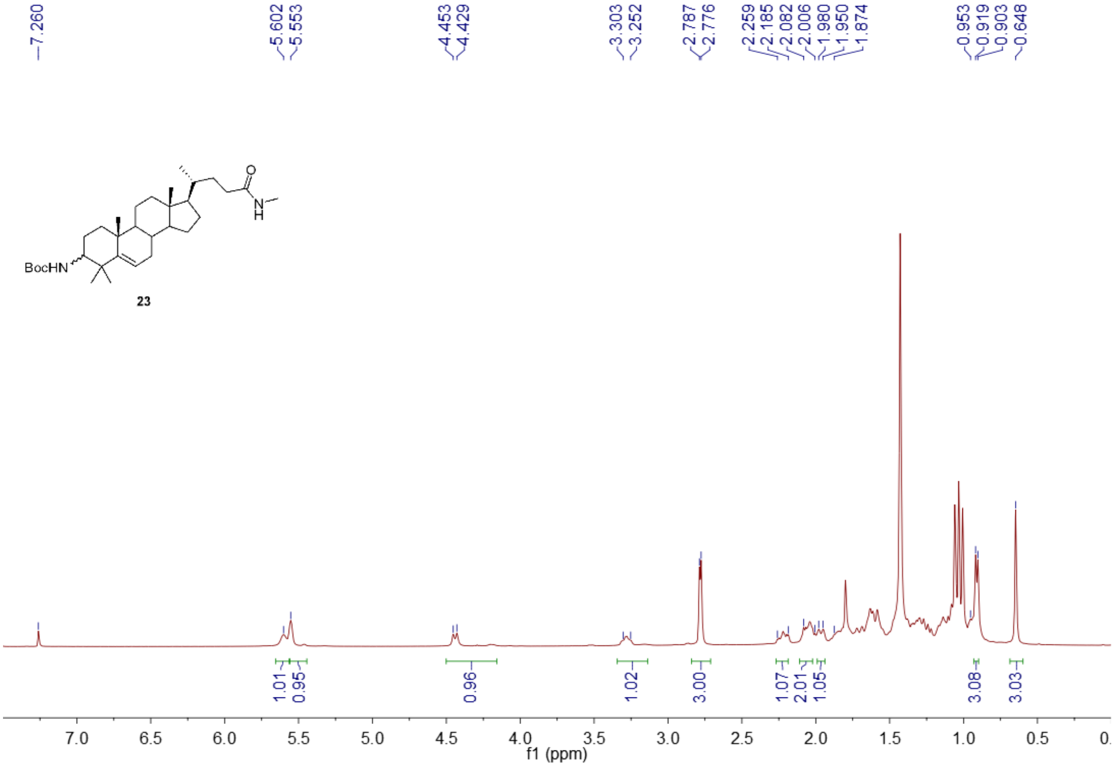

Compound **5al** (^{1}H NMR)

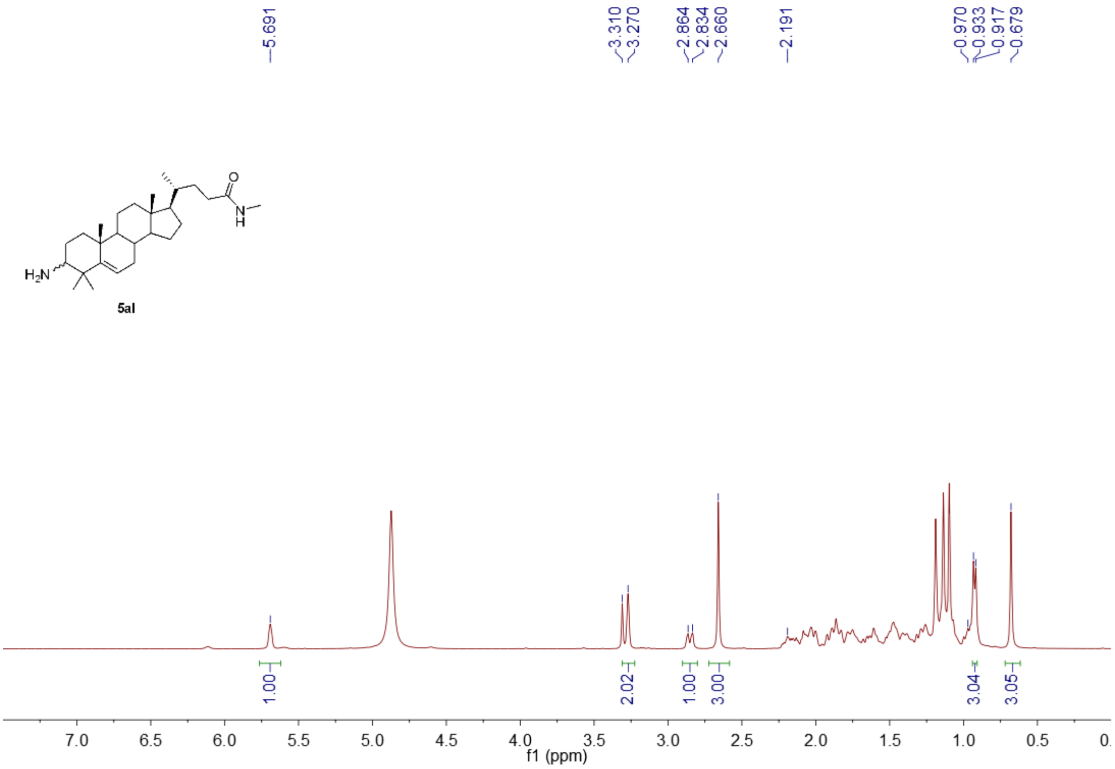

Compound **5al** (^{13}C NMR)

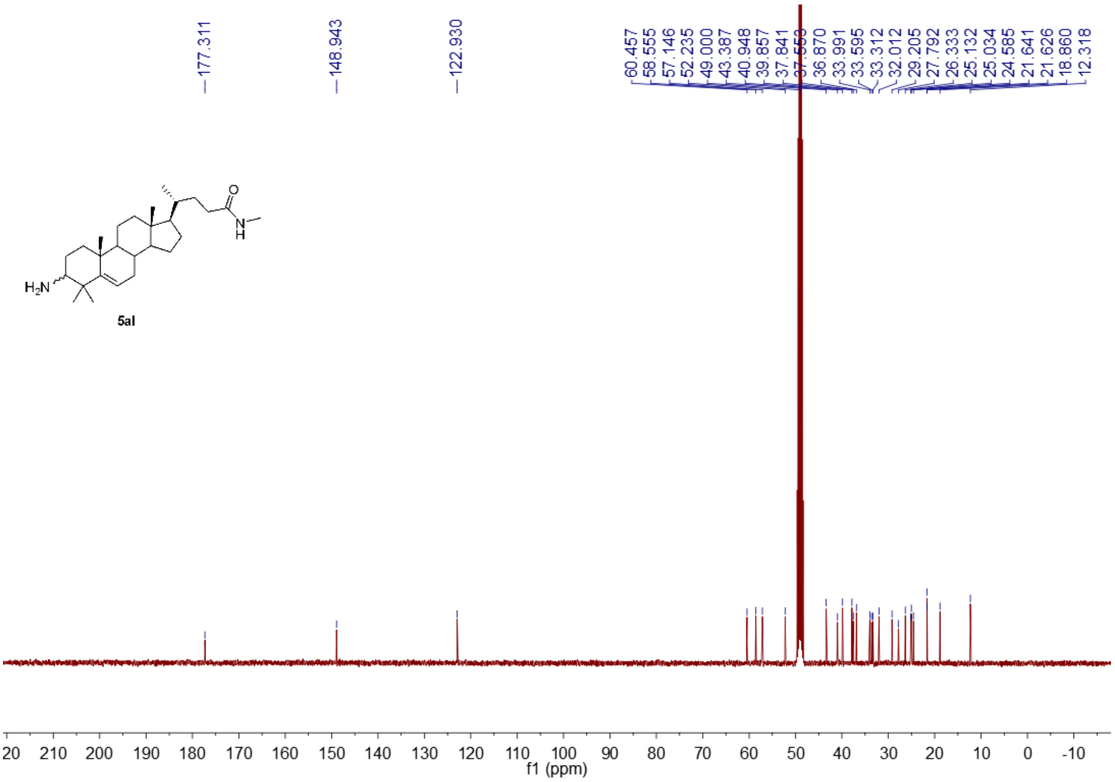

Compound **24** (^{1}H NMR)

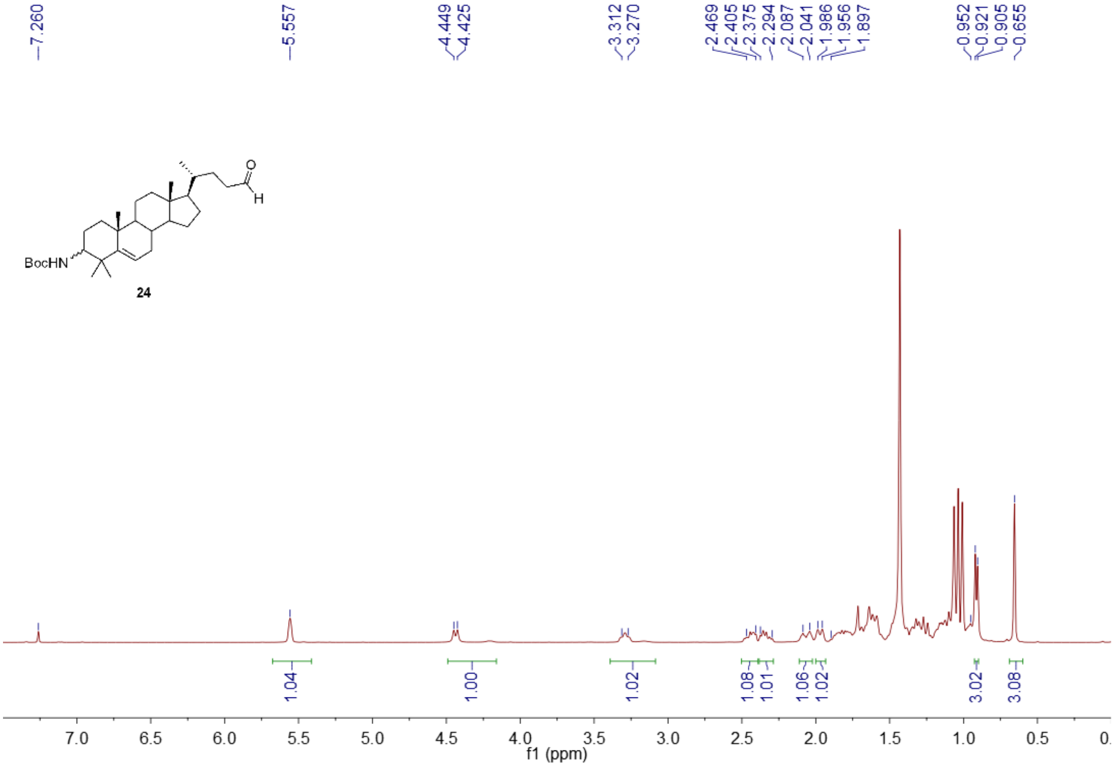

Compound **25** (^{1}H NMR)

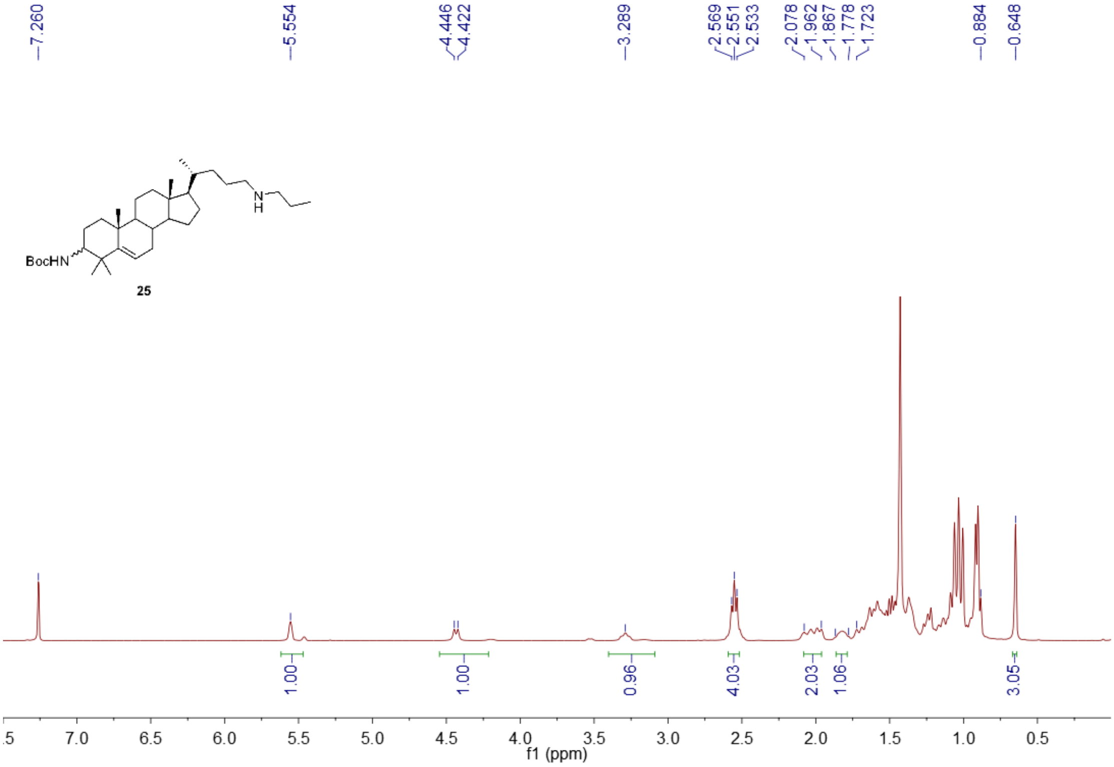

Compound **5am** (^{1}H NMR)

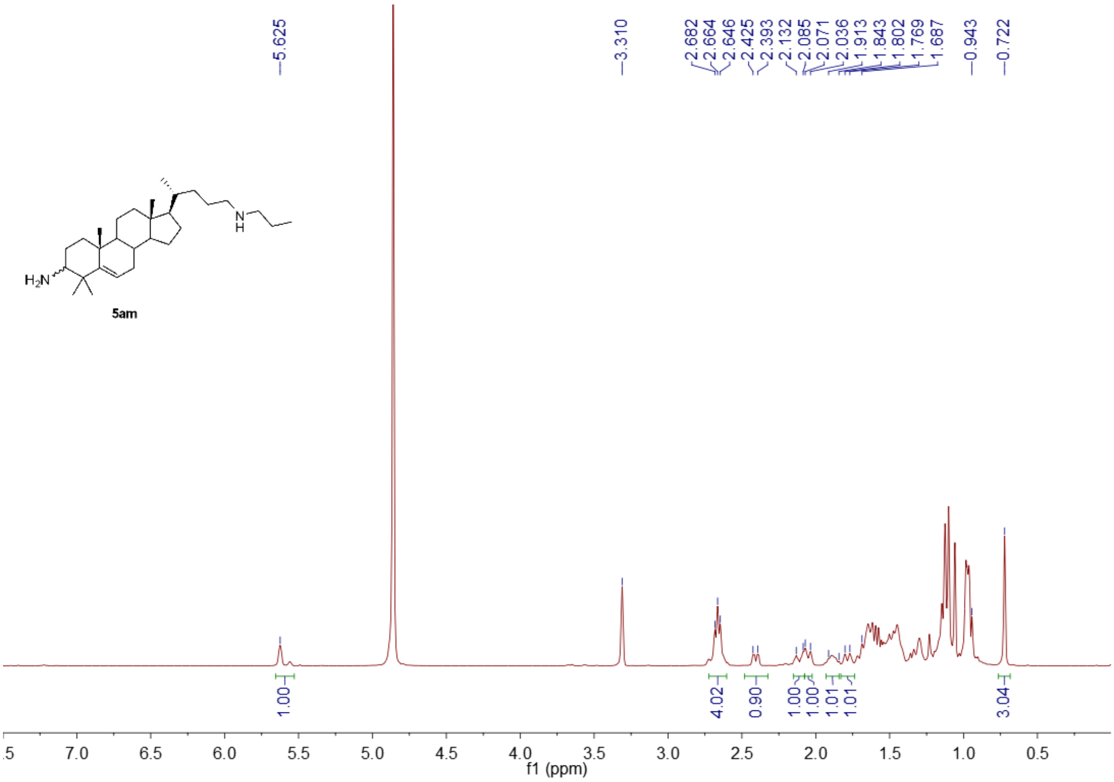

Compound **5am** (^{13}C NMR)

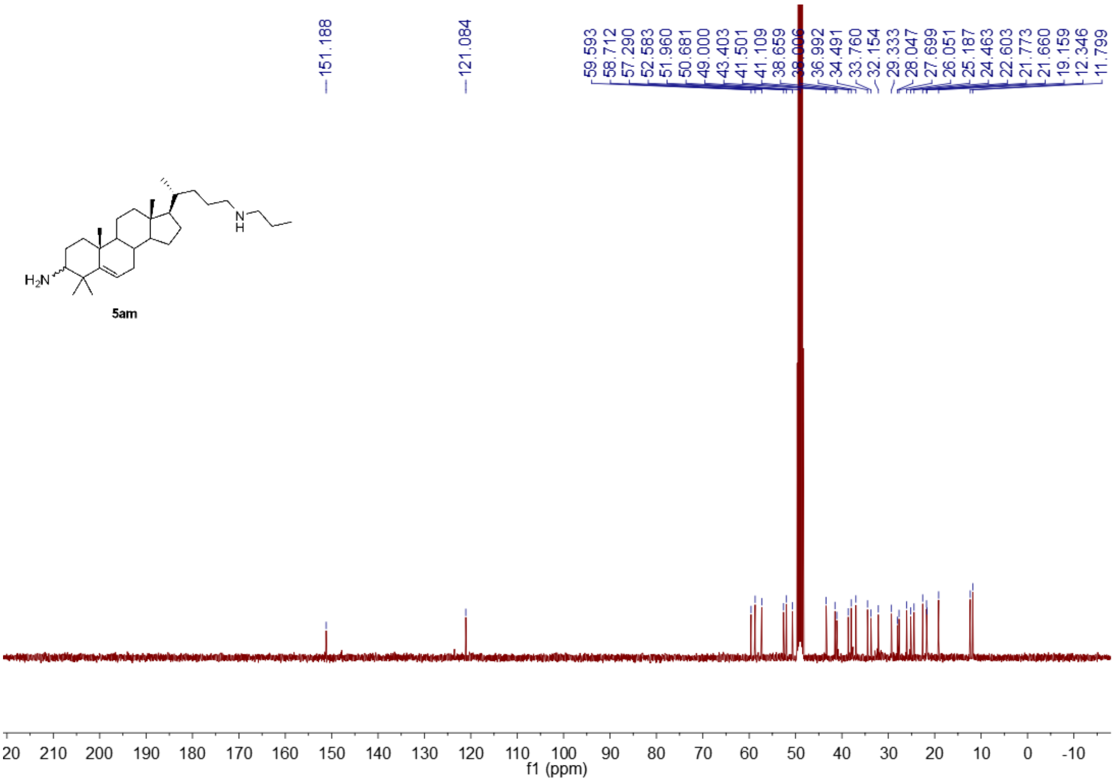

Compound **25a** (^{1}H NMR)

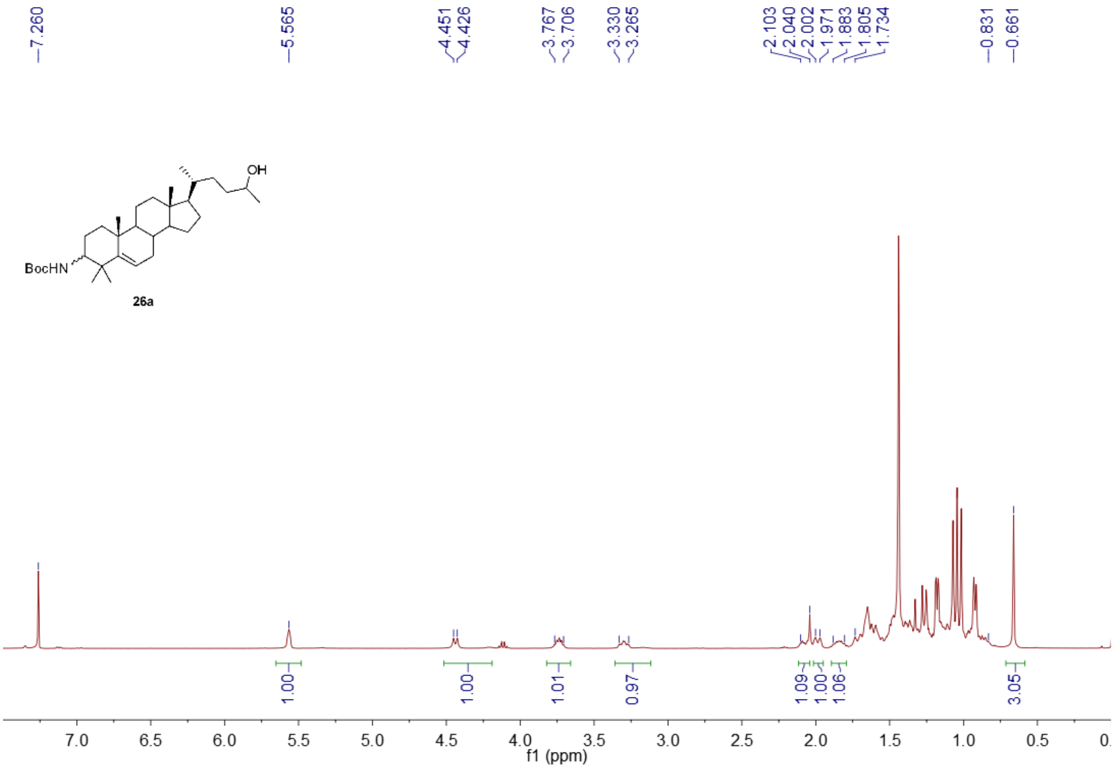

Compound **25b** (^{1}H NMR)

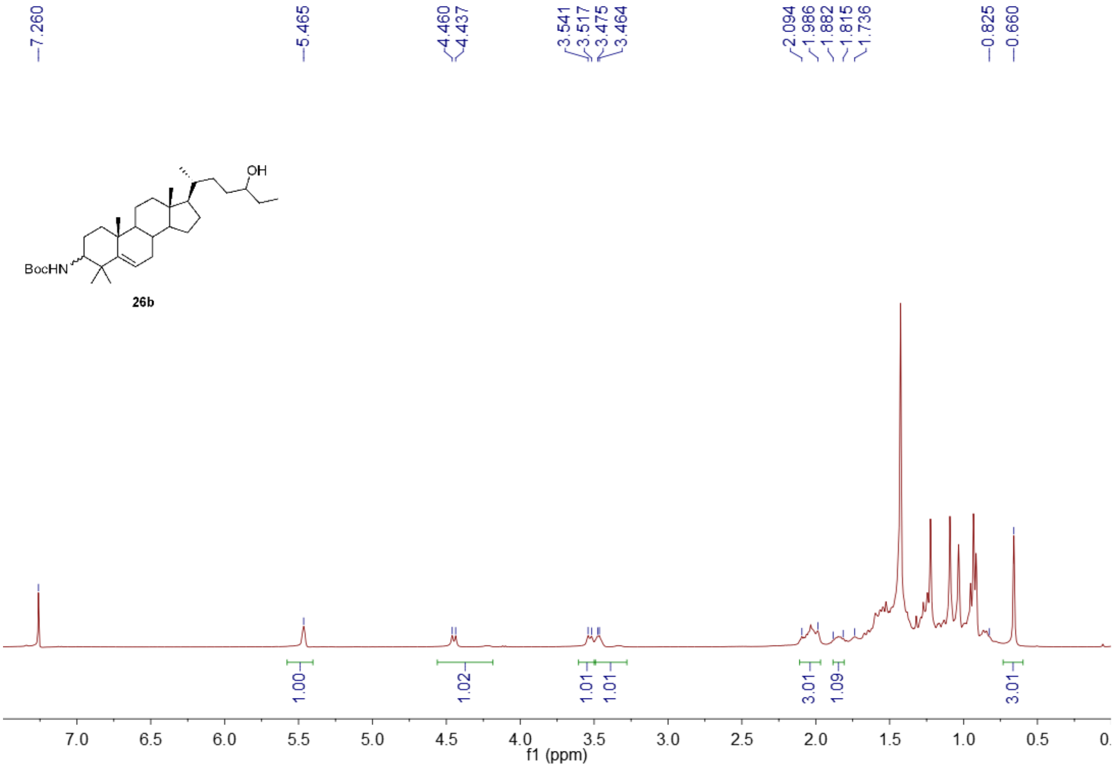

Compound **25c** (^{1}H NMR)

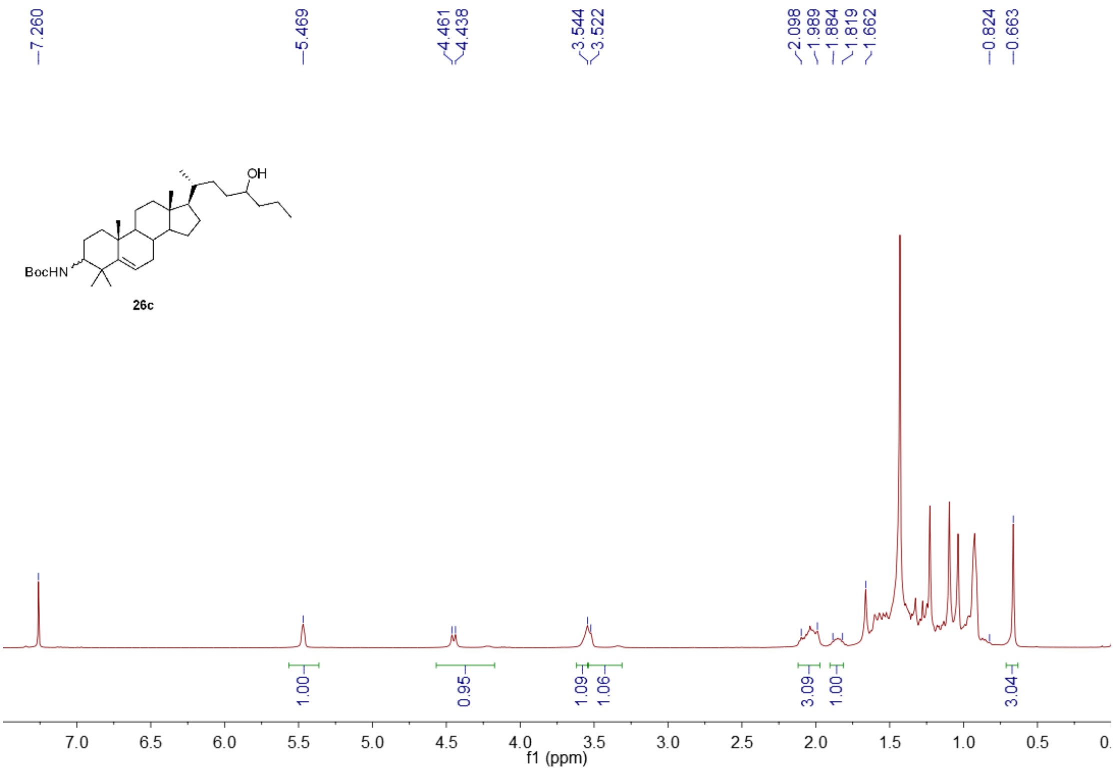

Compound **25e** (^{1}H NMR)

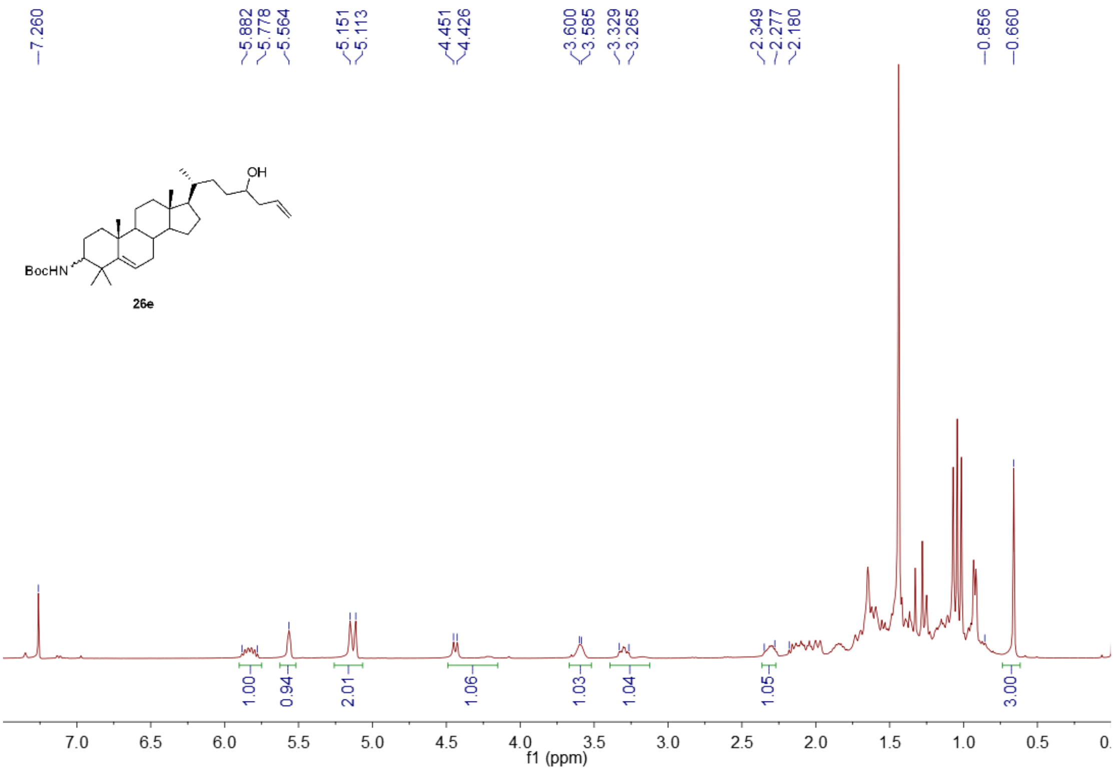

Compound **5an** (^{1}H NMR)

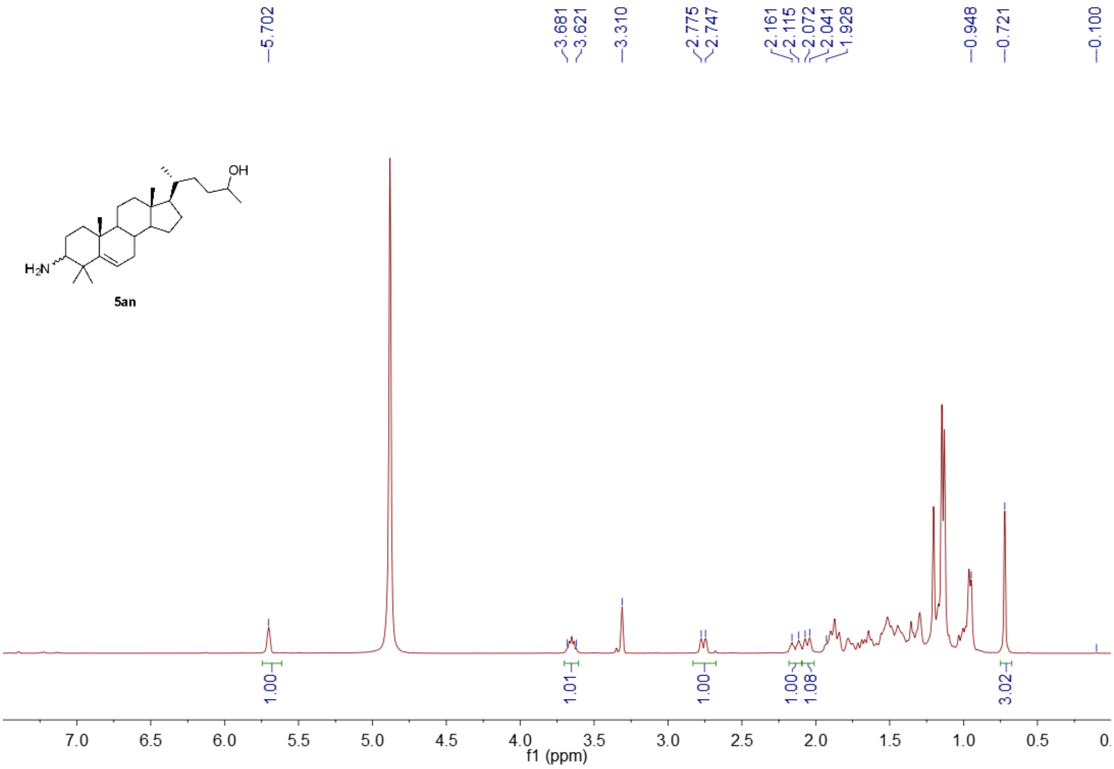

Compound **5an** (^{13}C NMR)

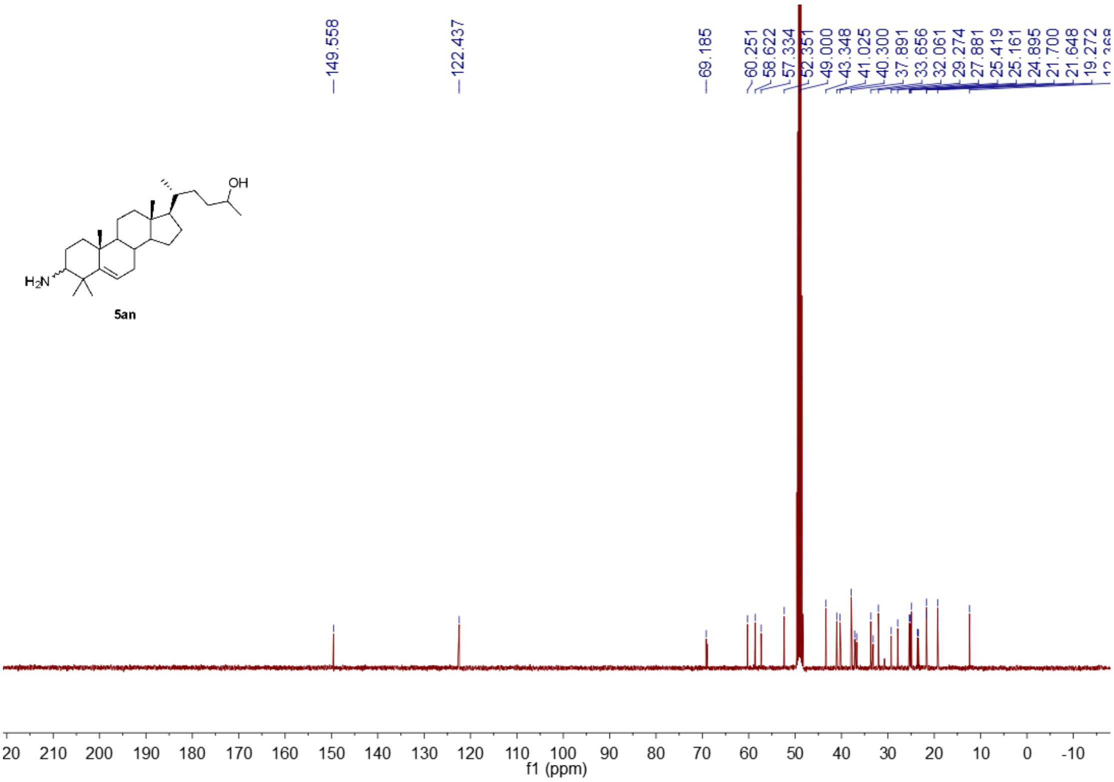

Compound **5ao** (^{1}H NMR)

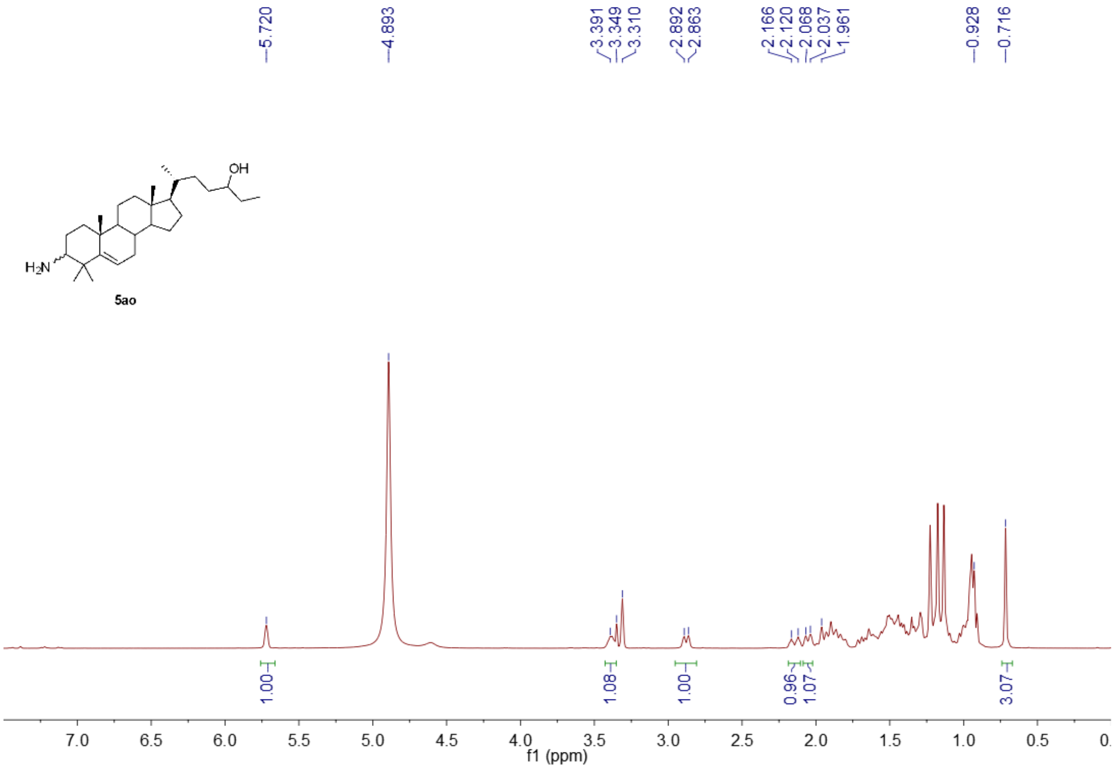

Compound **5ao** (^{13}C NMR)

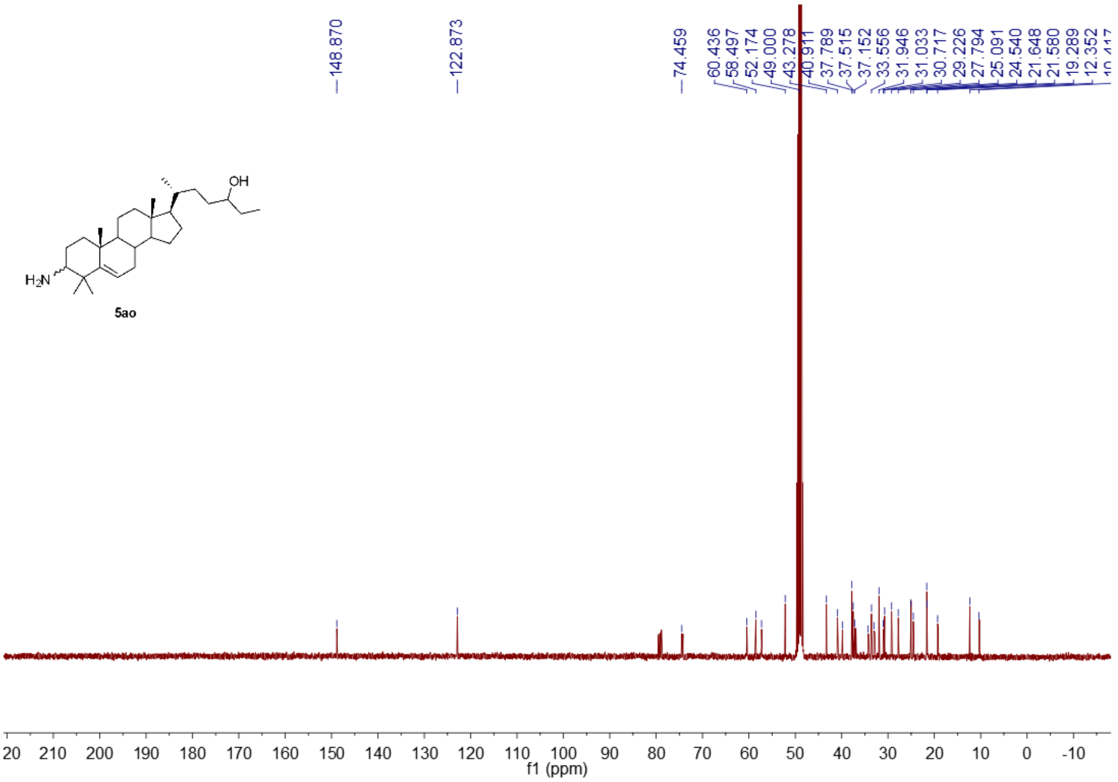

Compound **5ap** (^{1}H NMR)

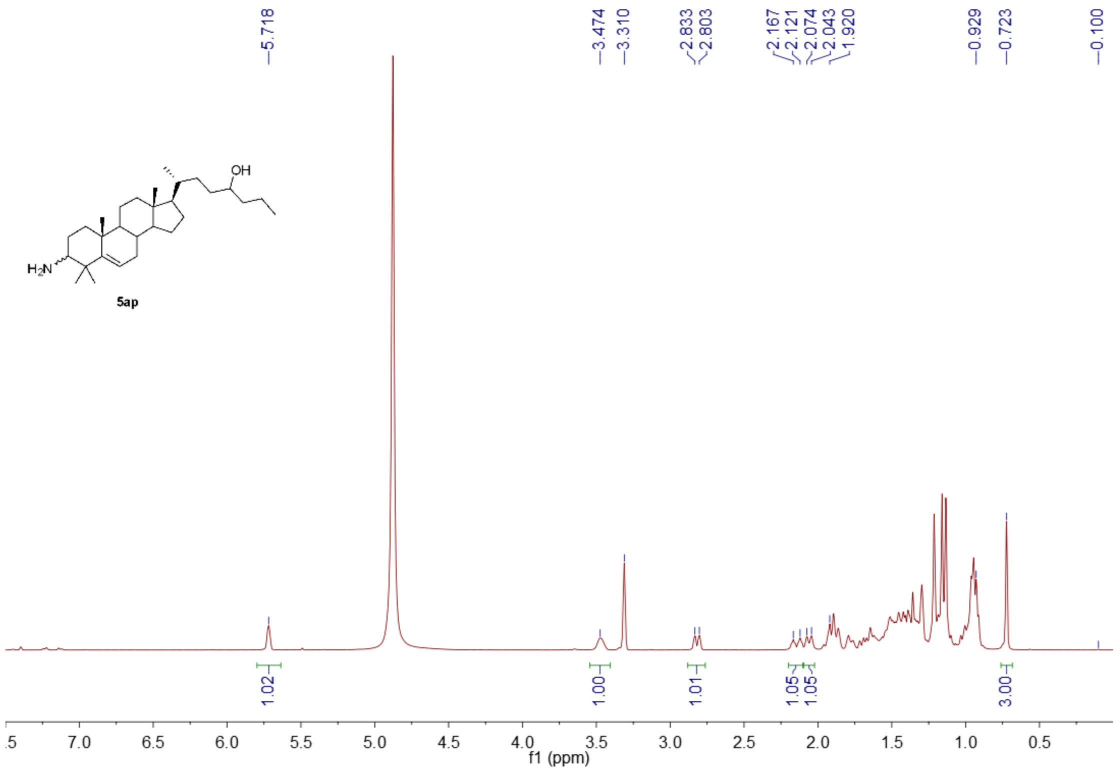

Compound **5ap** (^{13}C NMR)

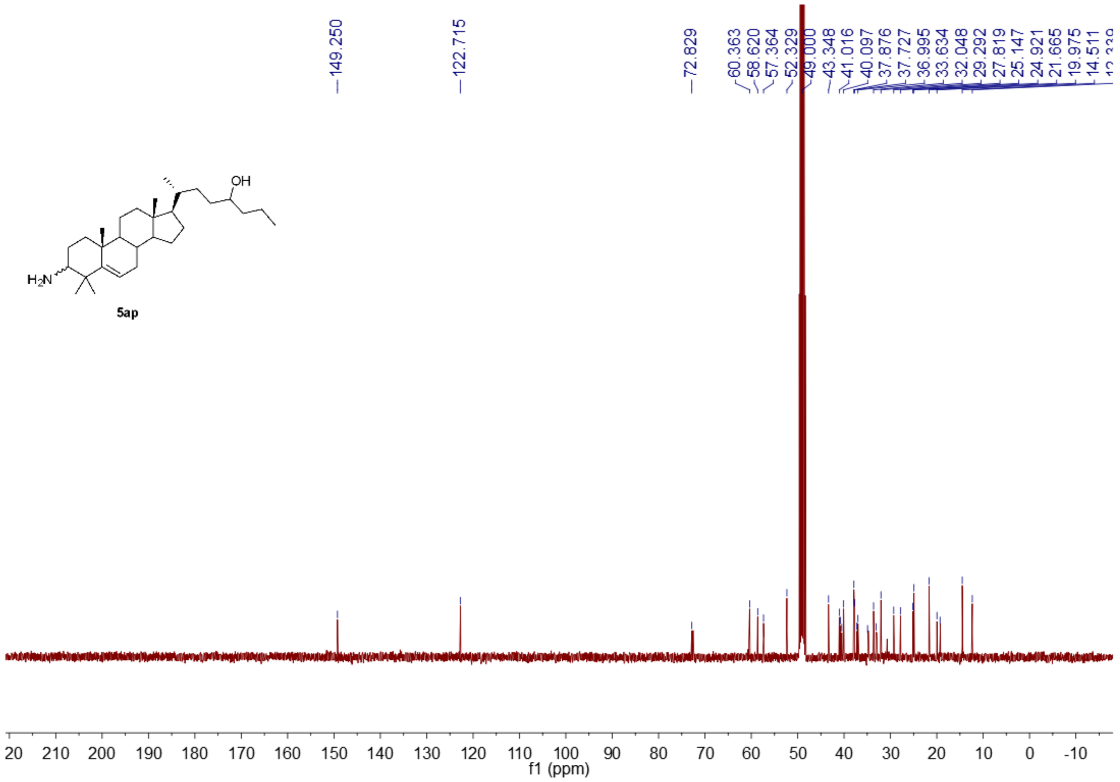

Compound **5aq** (^{1}H NMR)

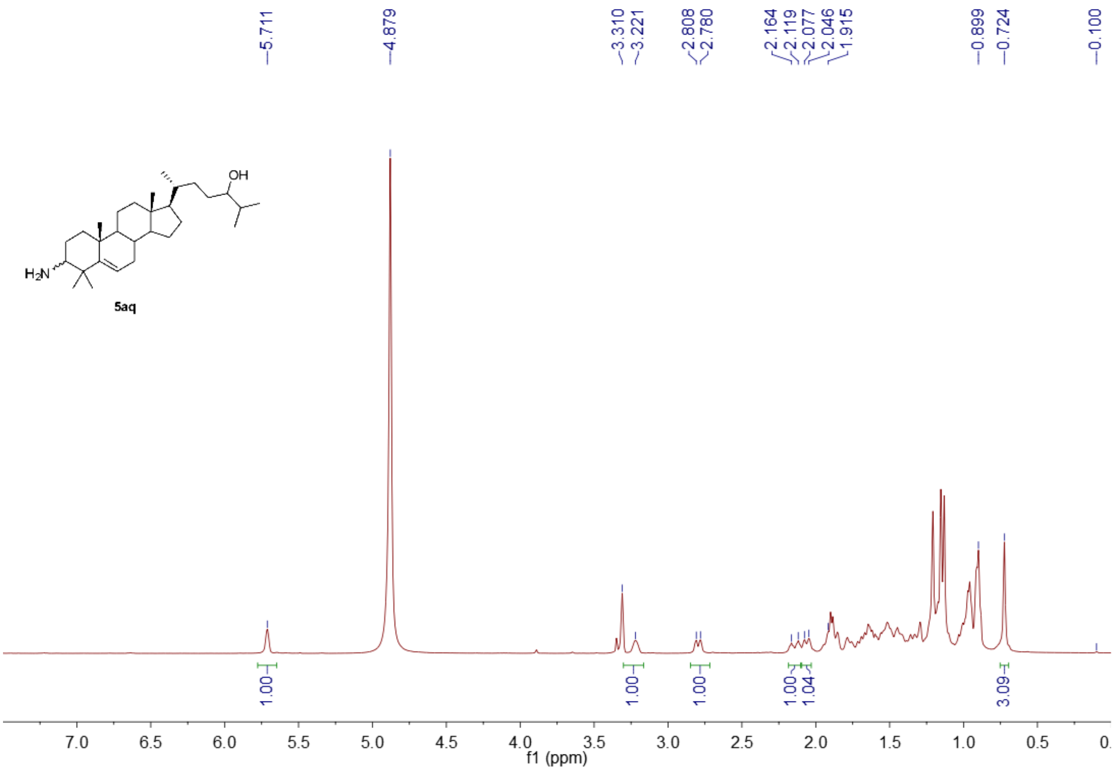

Compound **5aq** (^{13}C NMR)

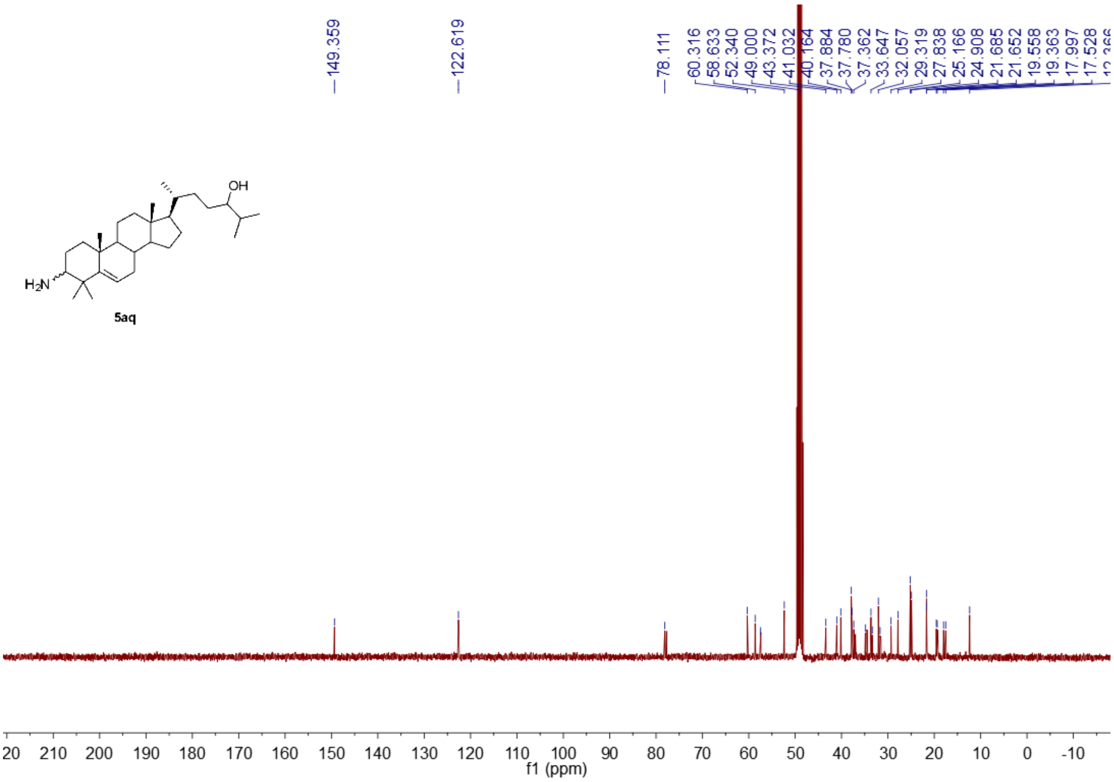

Compound **5ar** (^{1}H NMR)

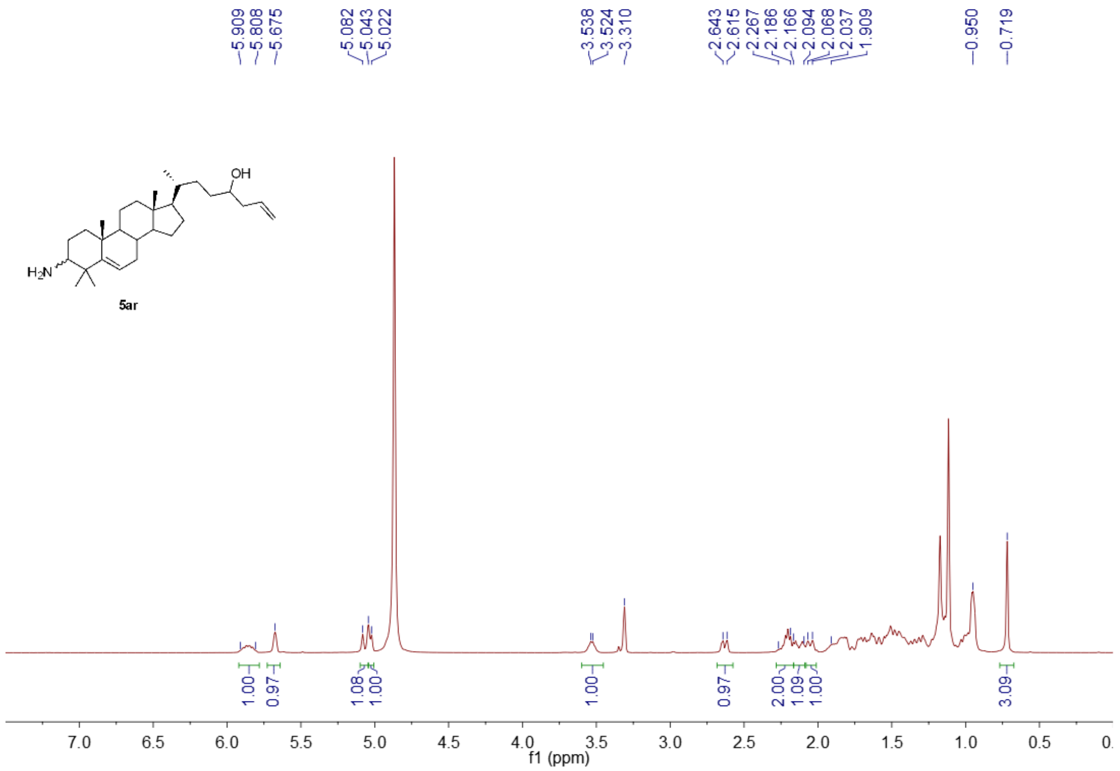

Compound **5ar** (^{13}C NMR)

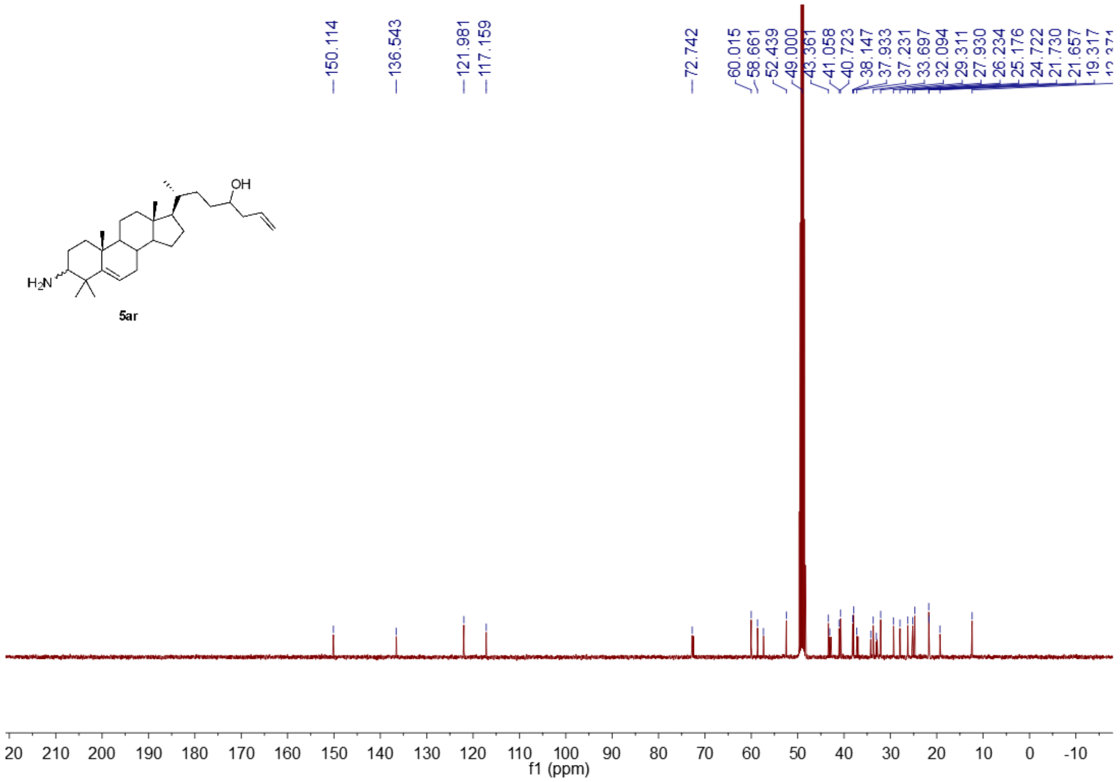

Compound **27a** (^{1}H NMR)

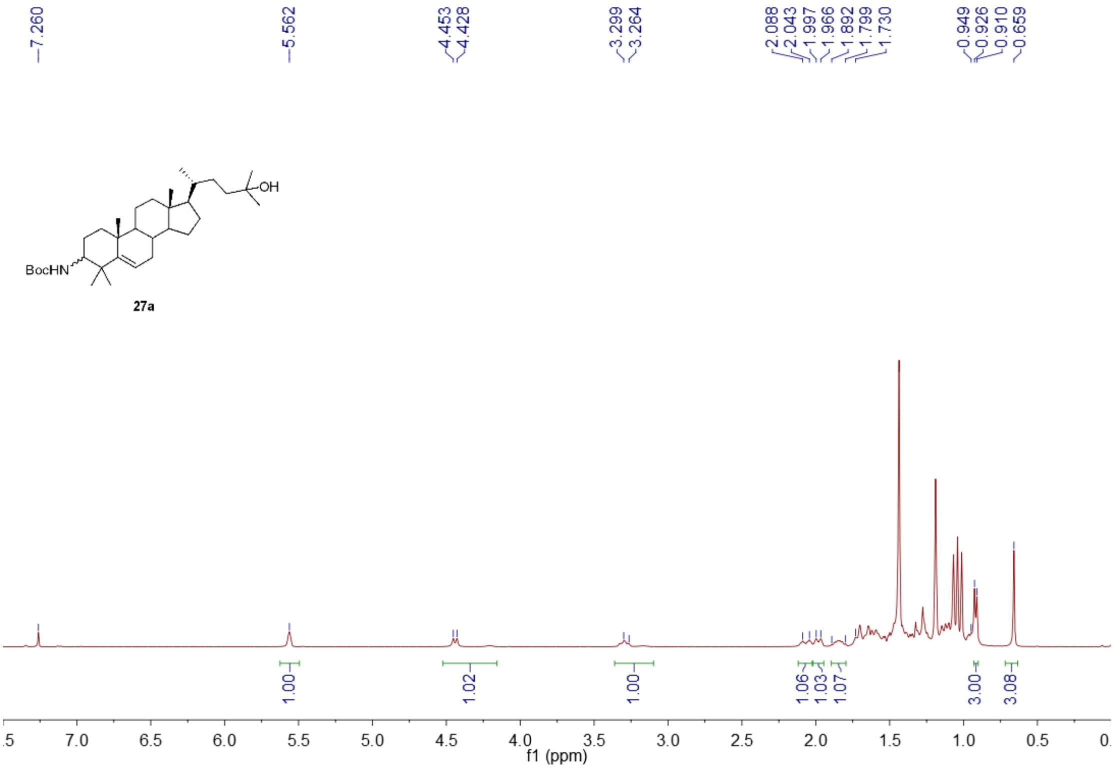

Compound **27b** (^{1}H NMR)

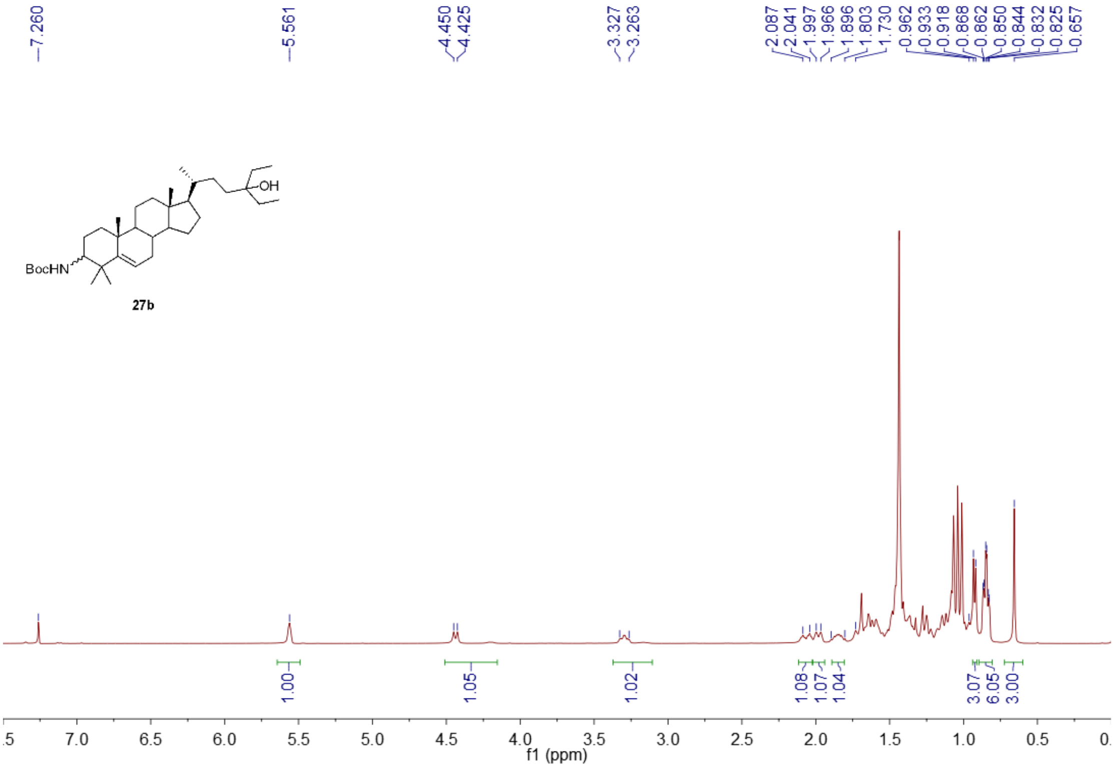

Compound **27c** (^{1}H NMR)

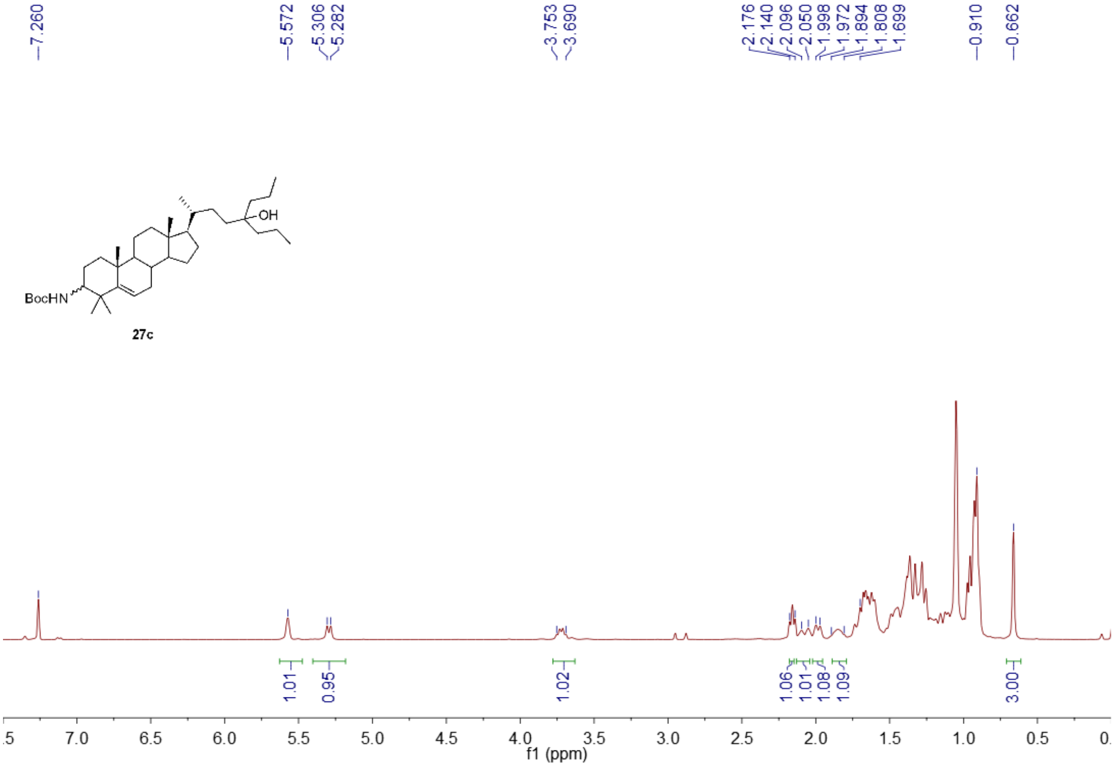

Compound **27d** (^{1}H NMR)

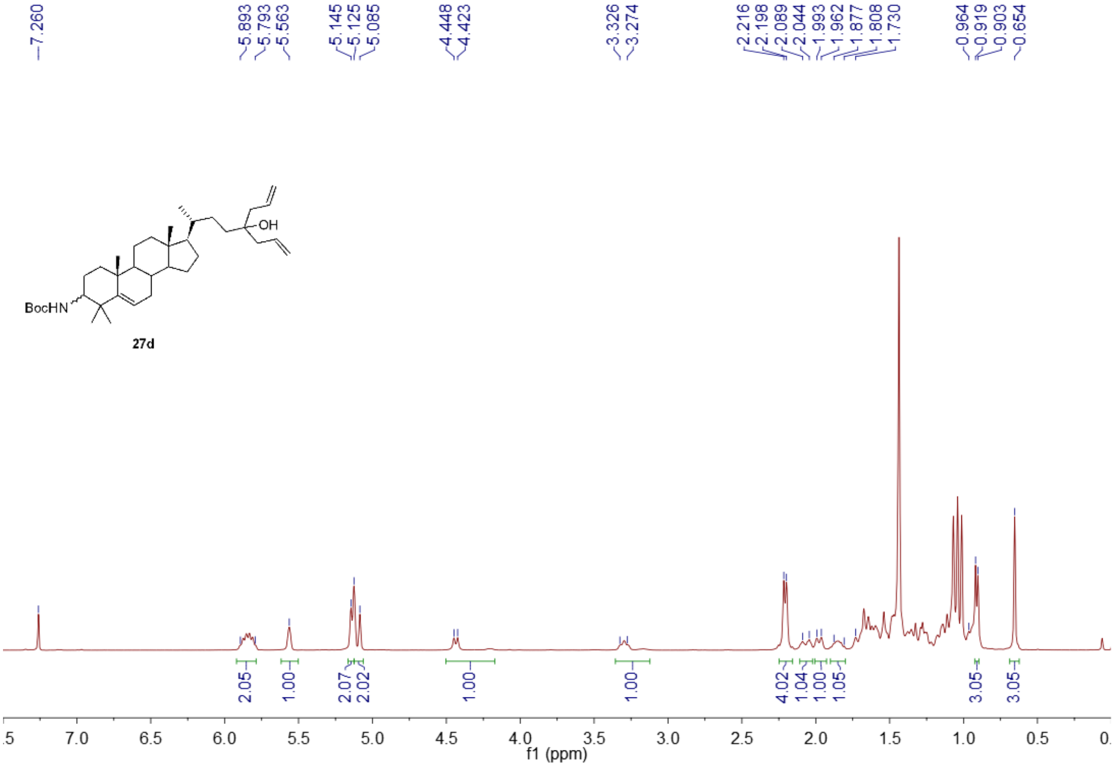

Compound **5as** (^{1}H NMR)

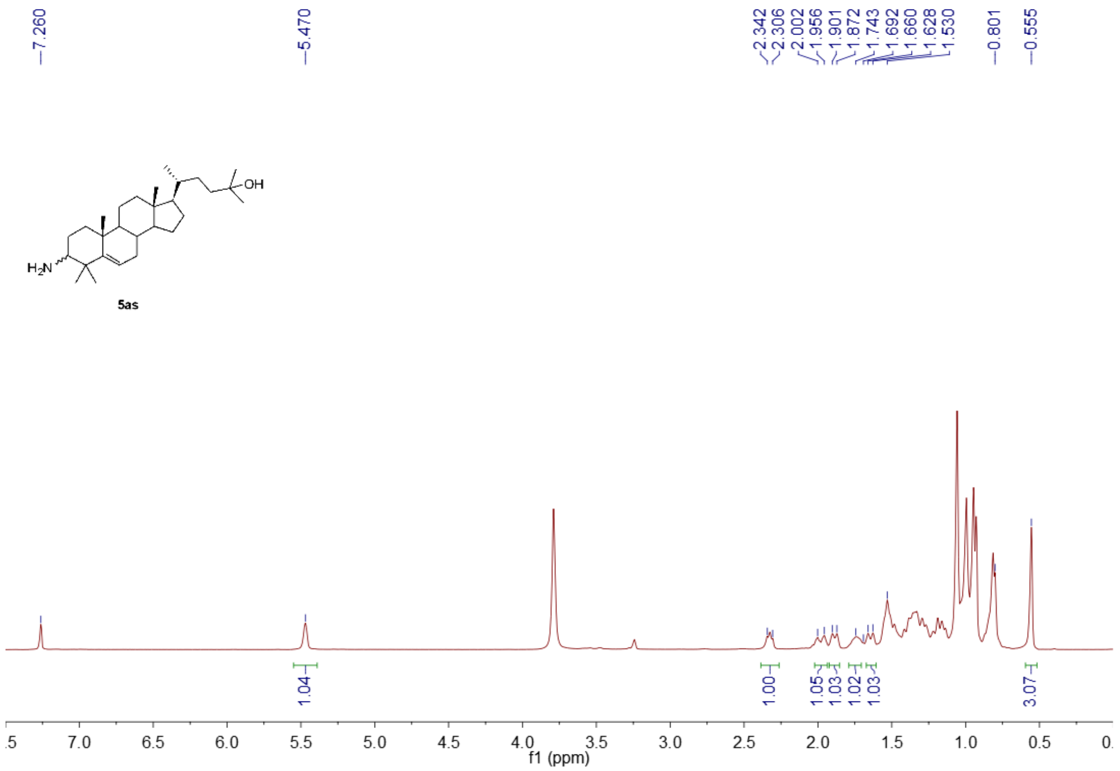

Compound **5as** (^{13}C NMR)

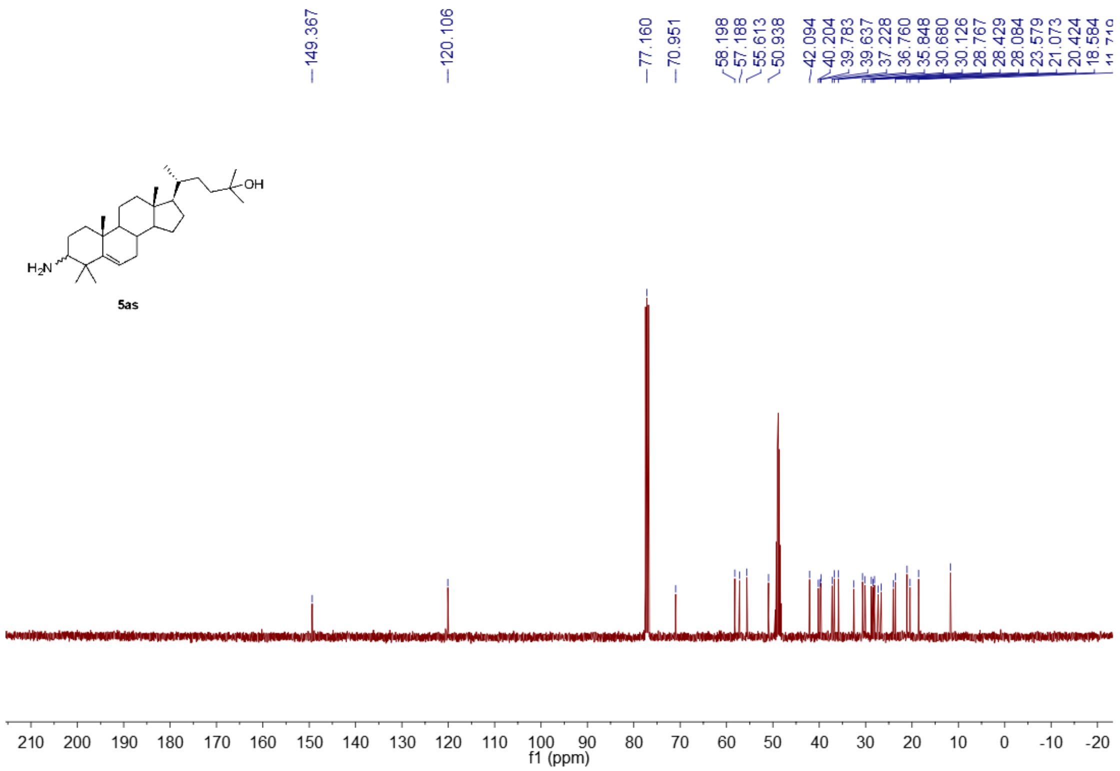

Compound **5at** (^{1}H NMR)

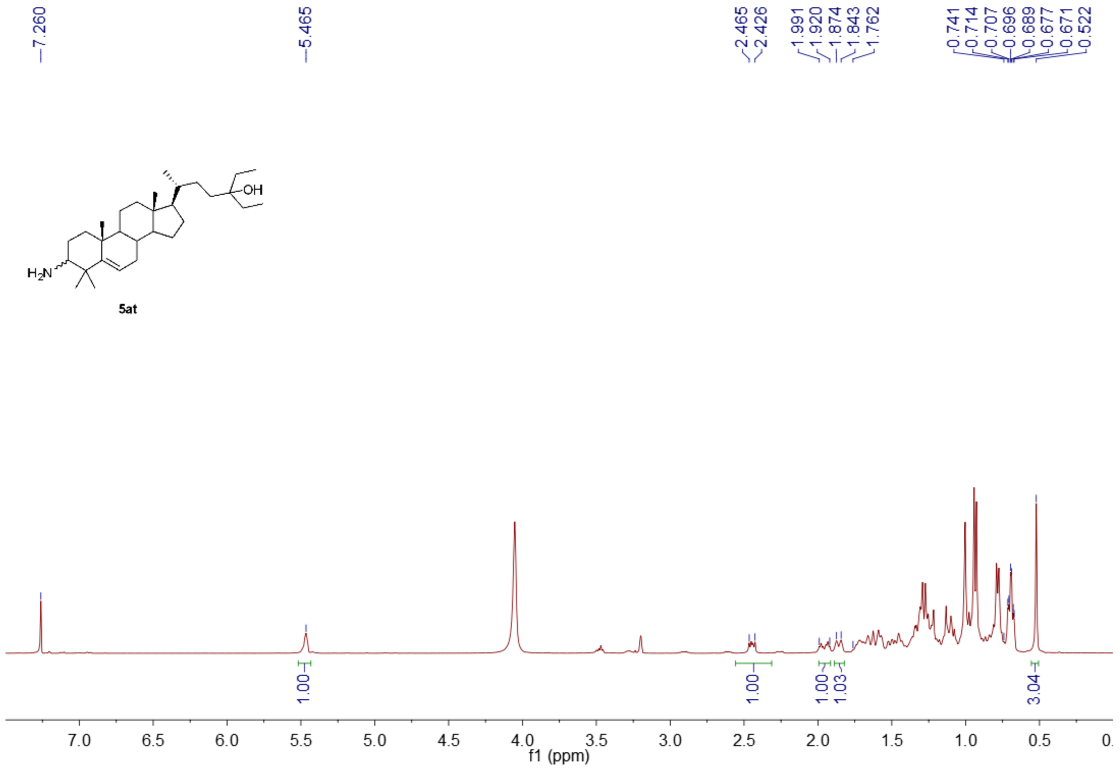

Compound **5at** (^{13}C NMR)

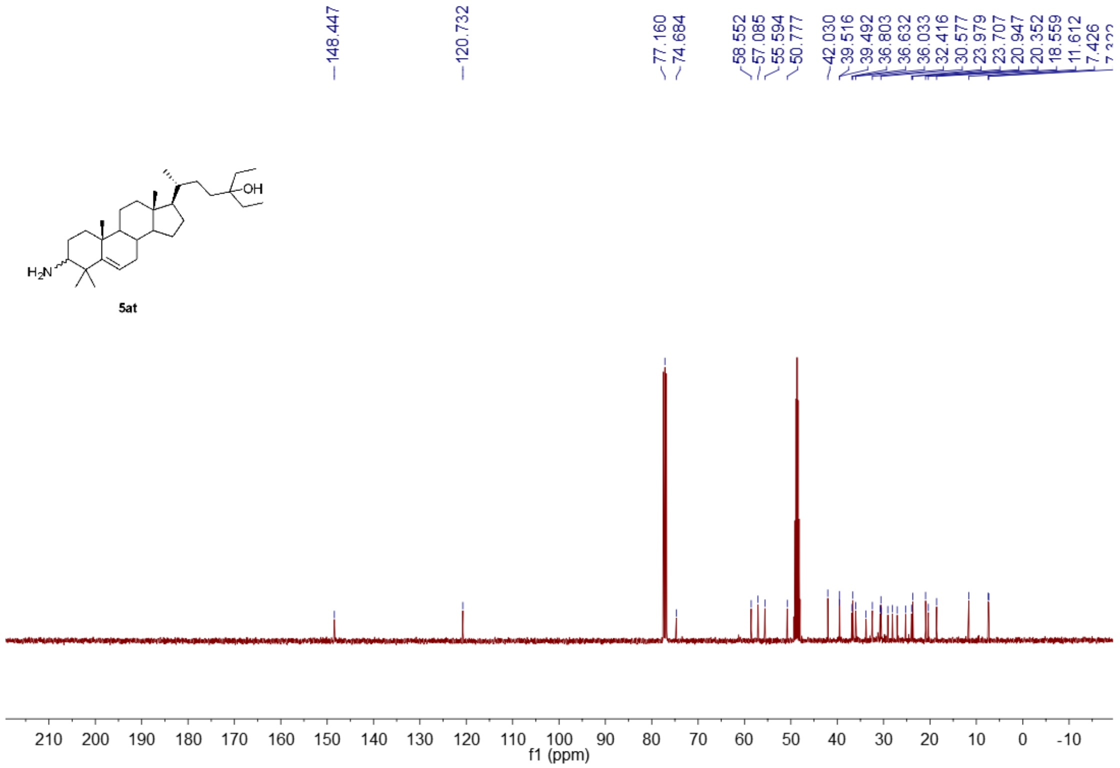

Compound **5au** (^{1}H NMR)

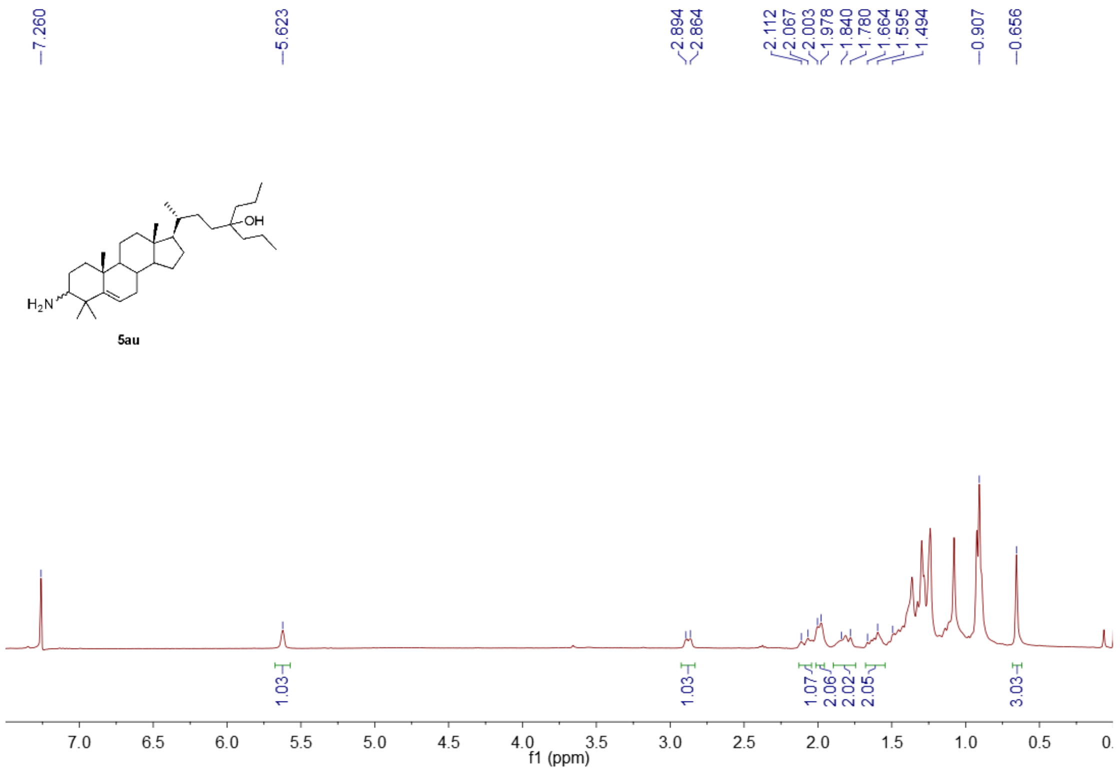

Compound **5au** (^{13}C NMR)

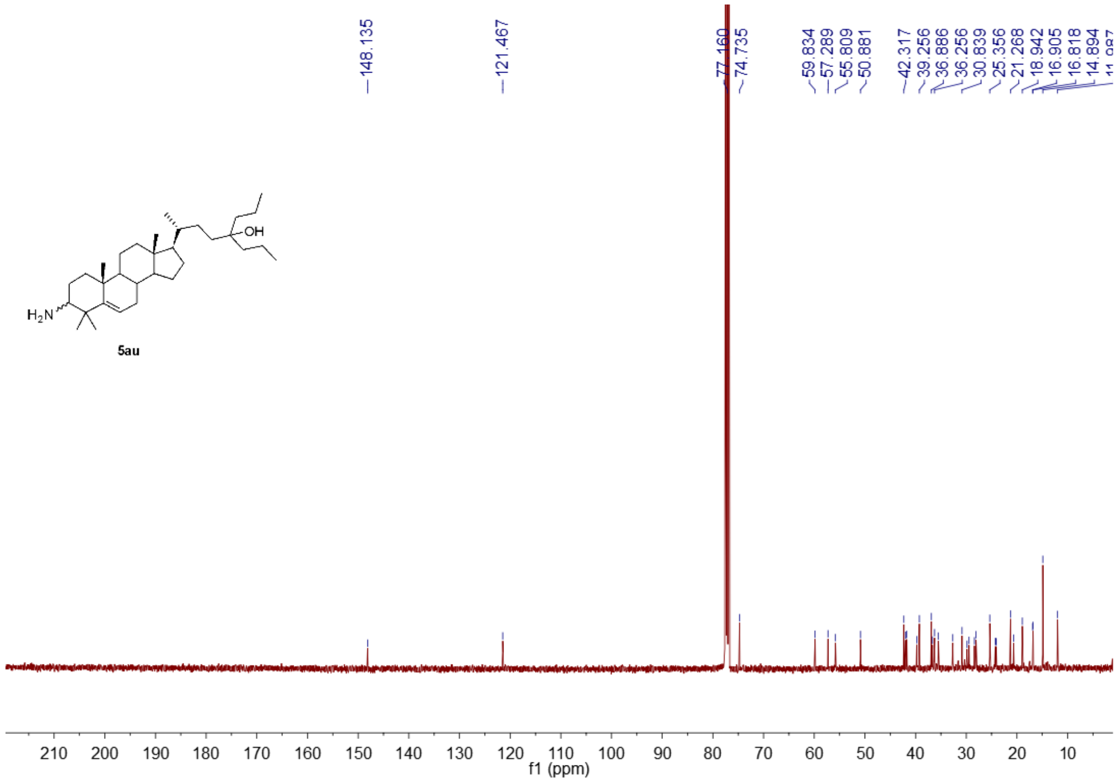

Compound **5av** (^{1}H NMR)

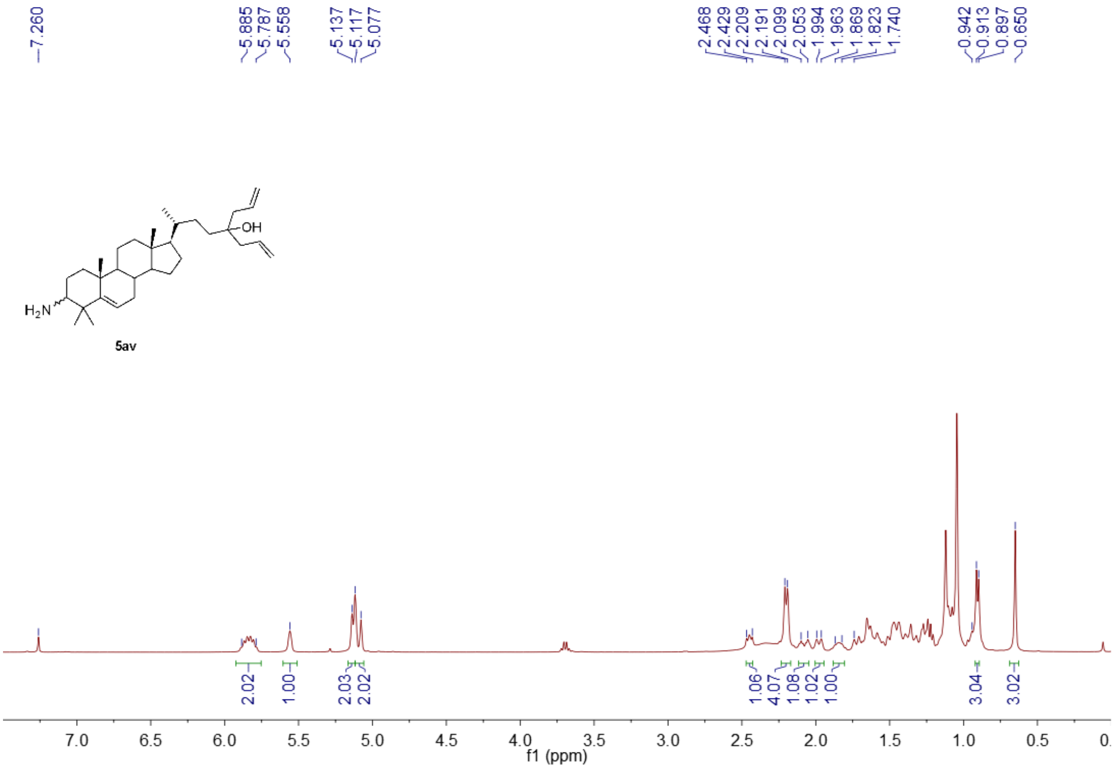

Compound **5av** (^{13}C NMR)

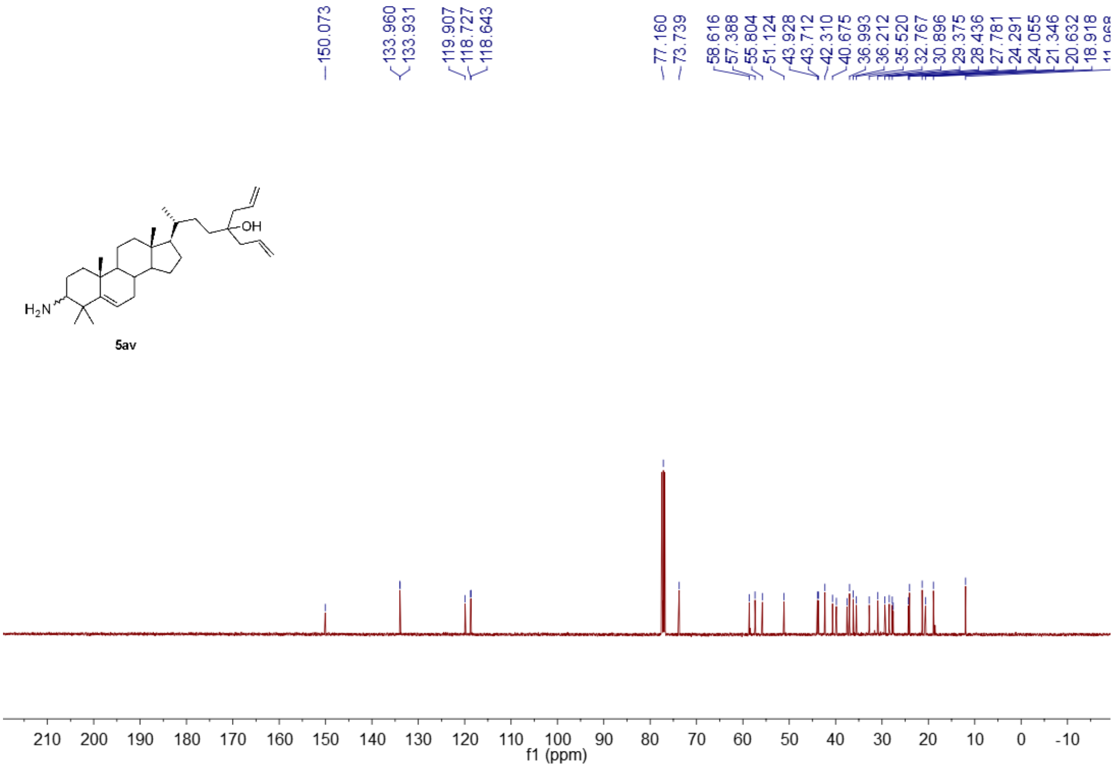

Compound **31** (^{1}H NMR)

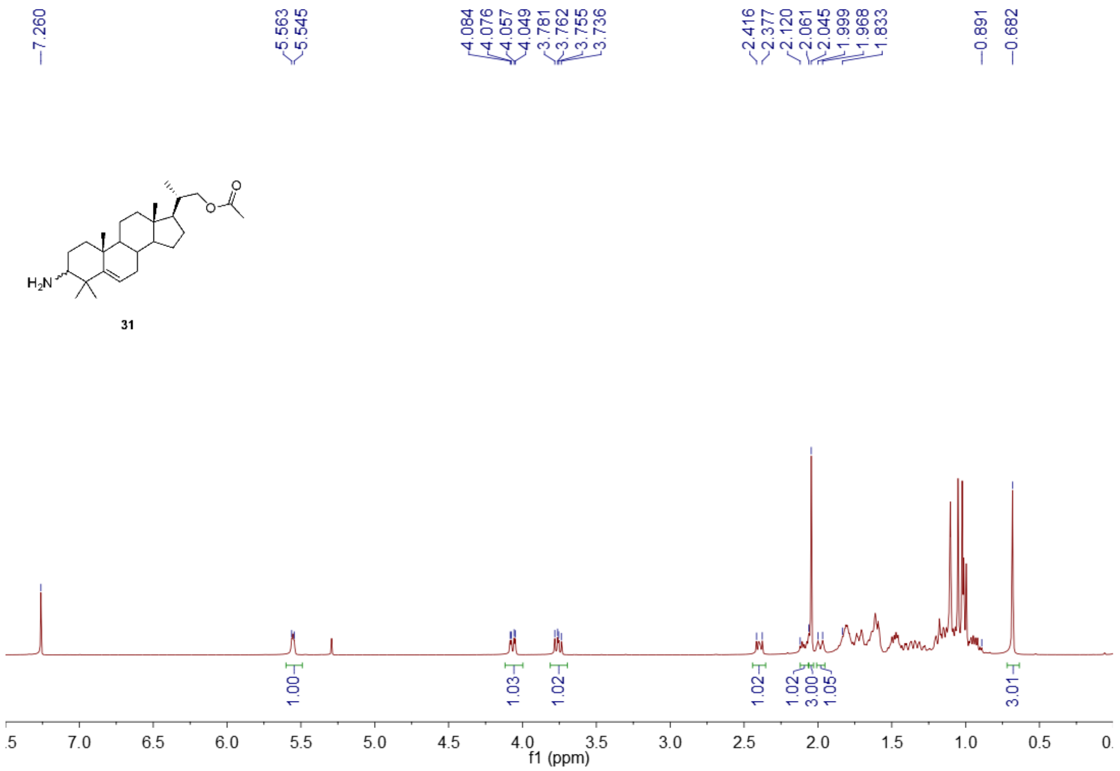

Compound **5aw** (^{1}H NMR)

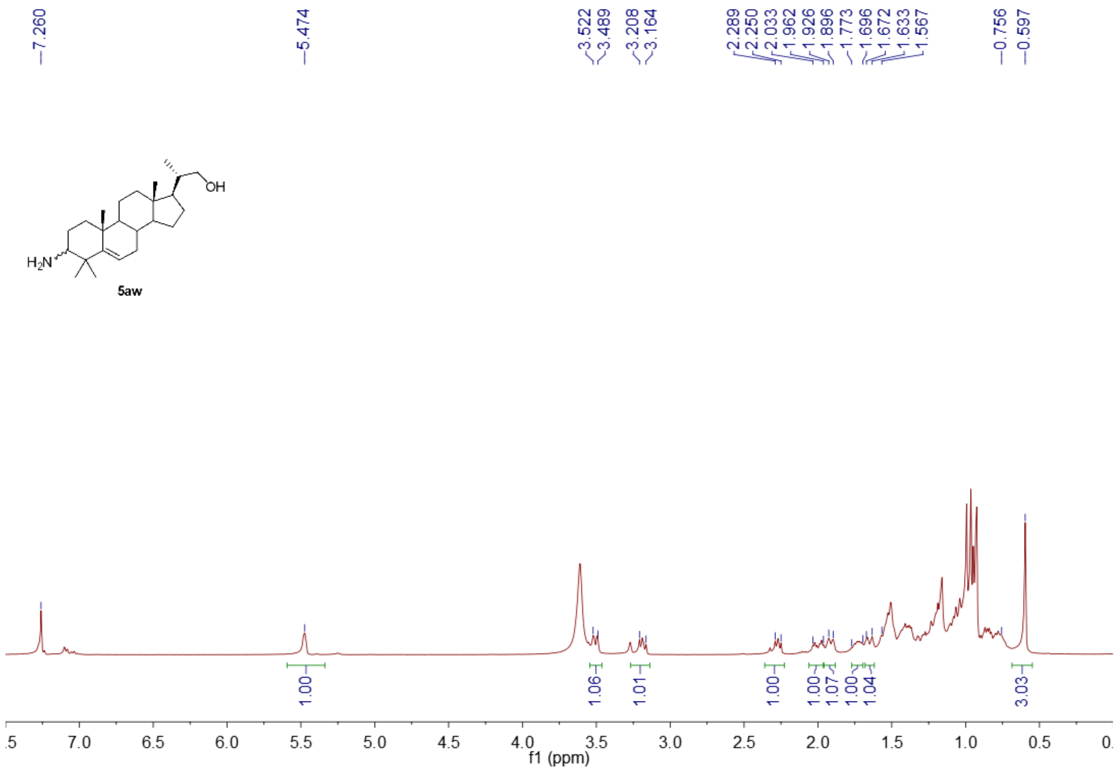

Compound **5aw** (^{13}C NMR)

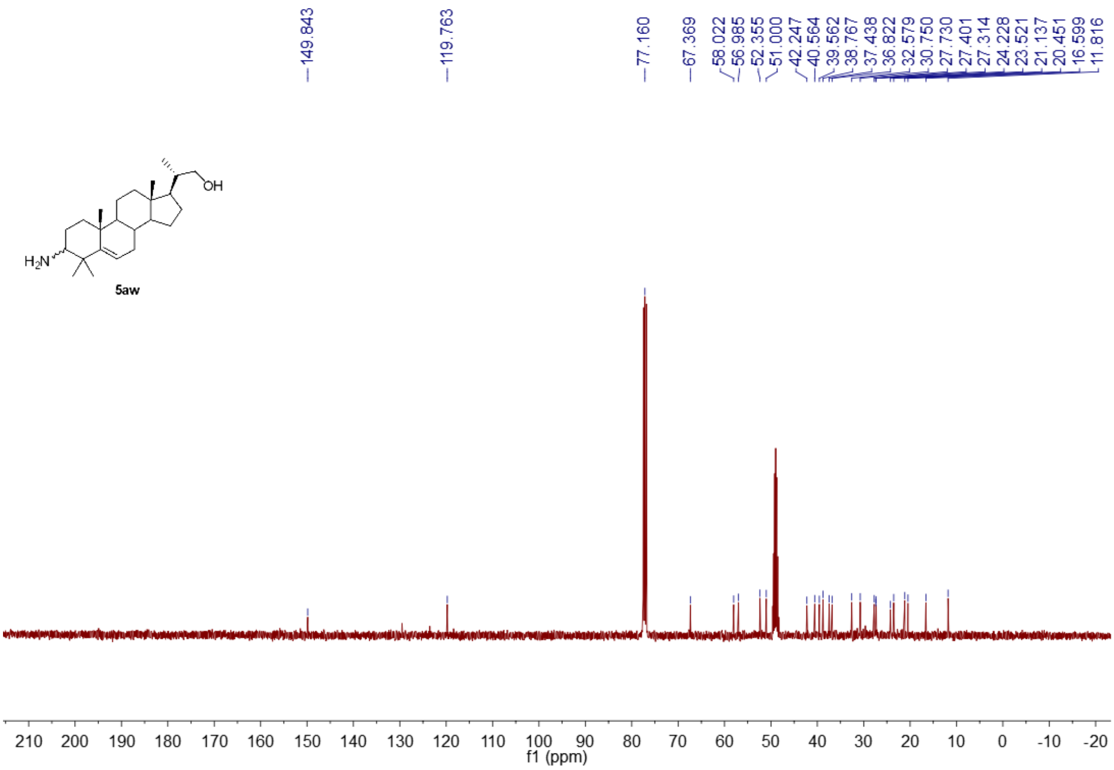

Compound **34** (^{1}H NMR)

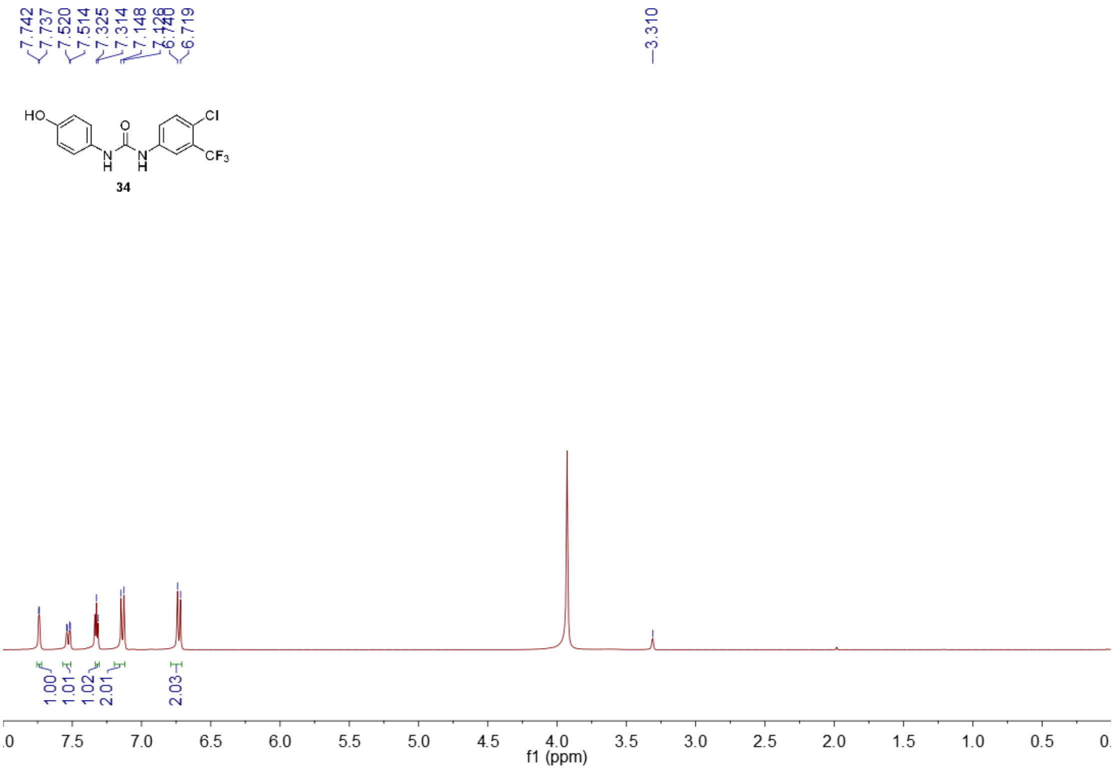

Compound **5ax** (^{1}H NMR)

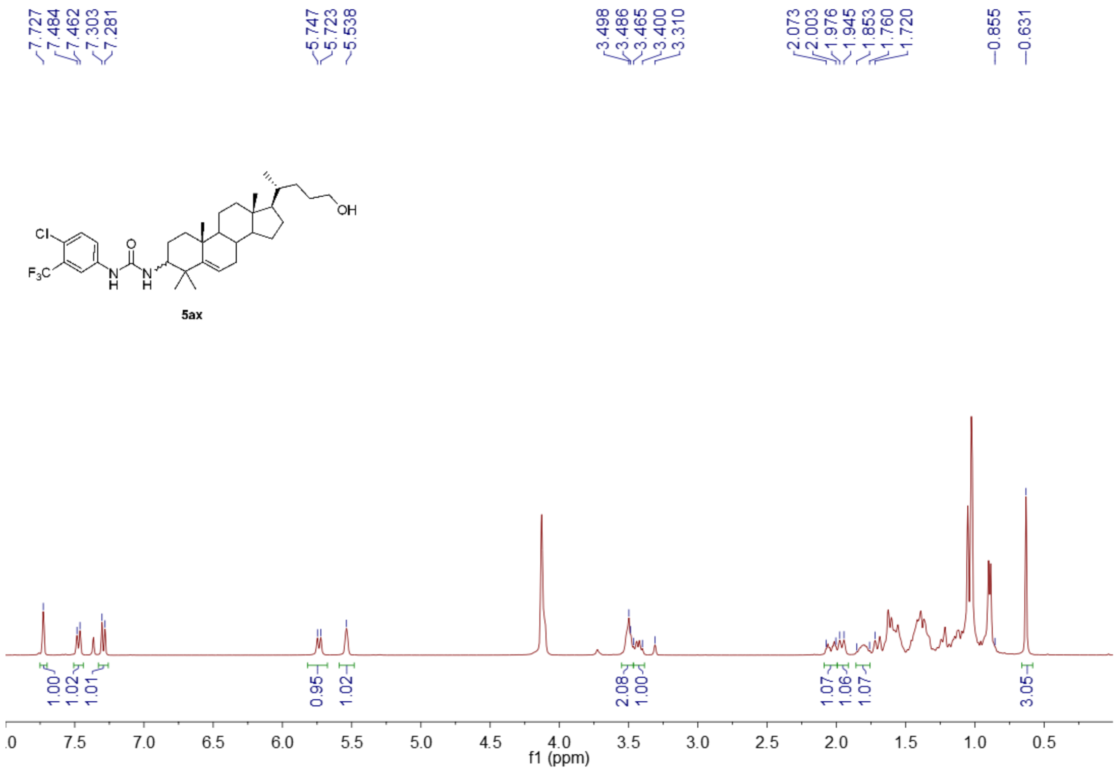

Compound **5ax** (^{13}C NMR)

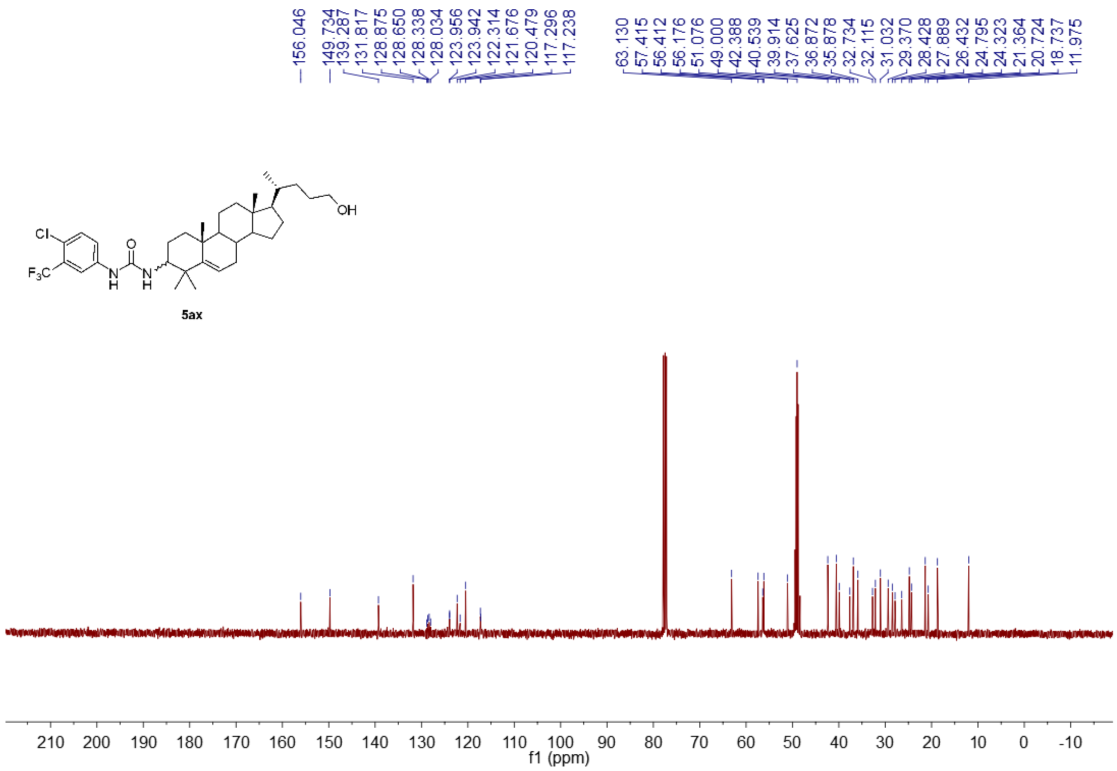

Compound **5ax** (^{19}F NMR)

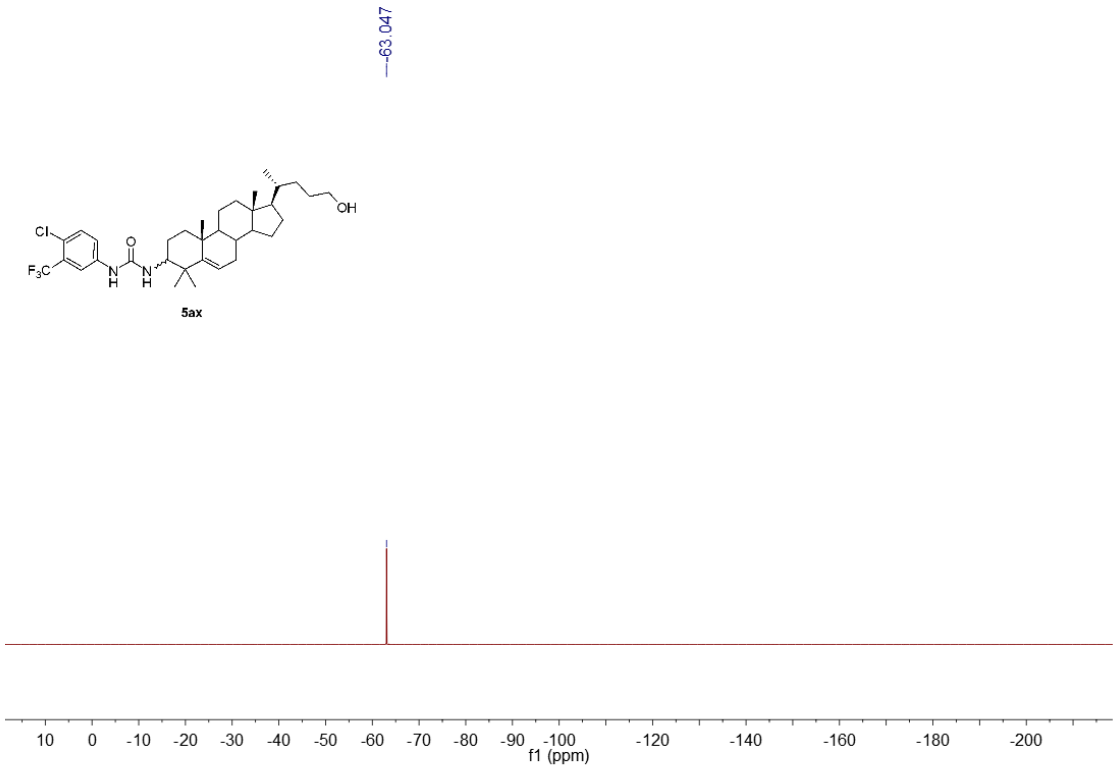

Compound **35** (^{1}H NMR)

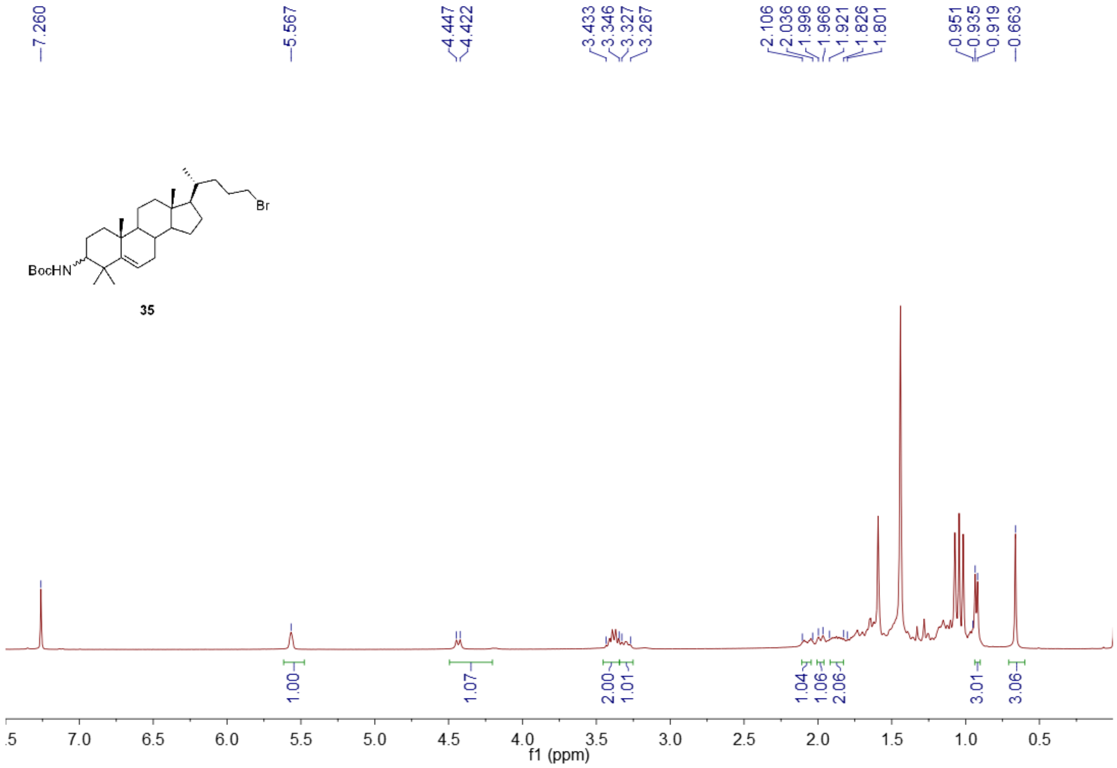

Compound **5ay** (^{1}H NMR)

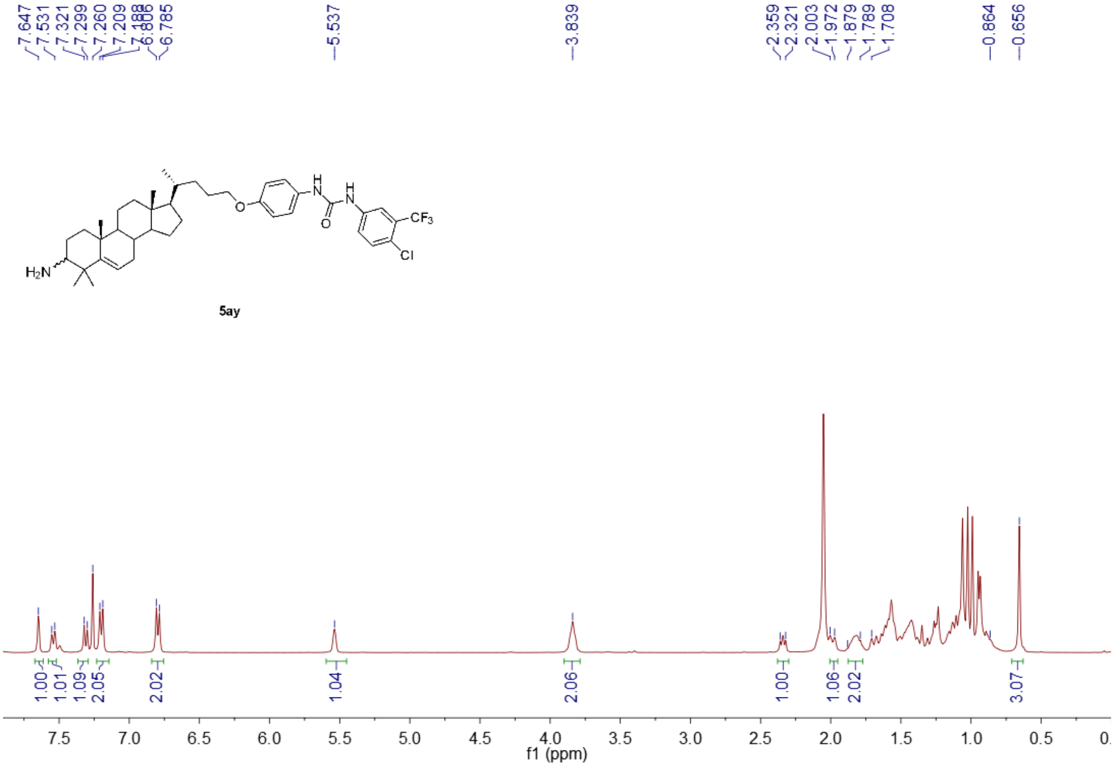

Compound **5ay** (^{13}C NMR)

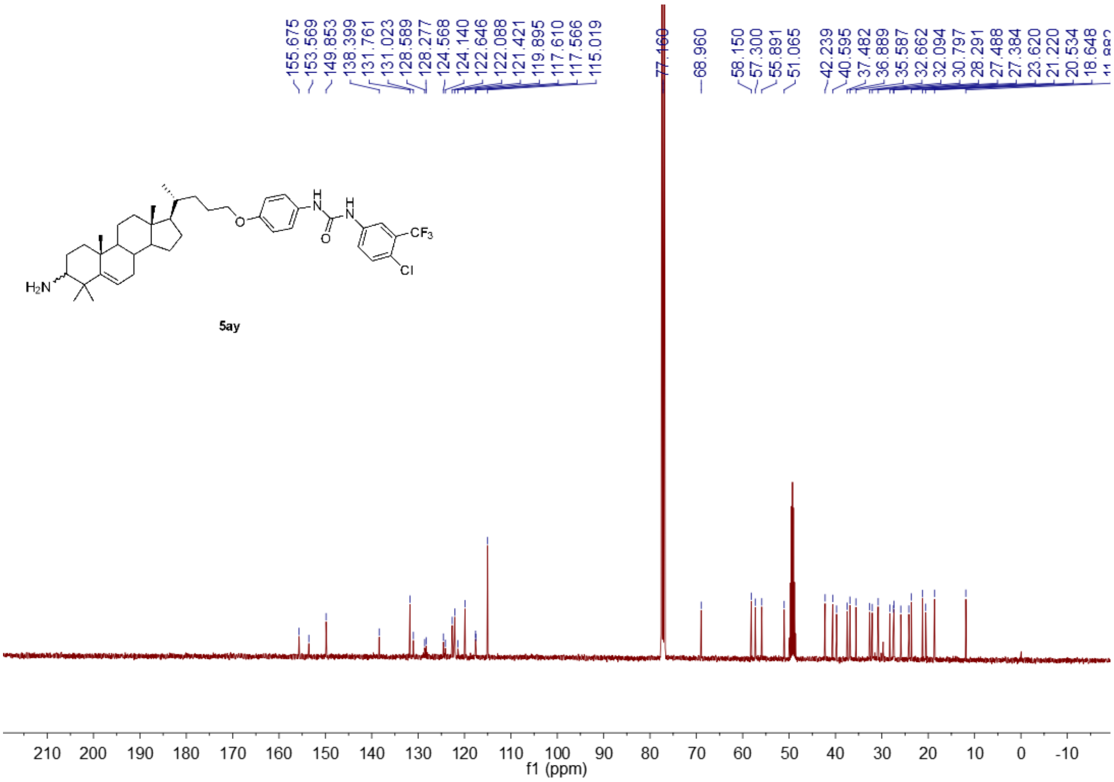

Compound **5ay** (^{19}F NMR)

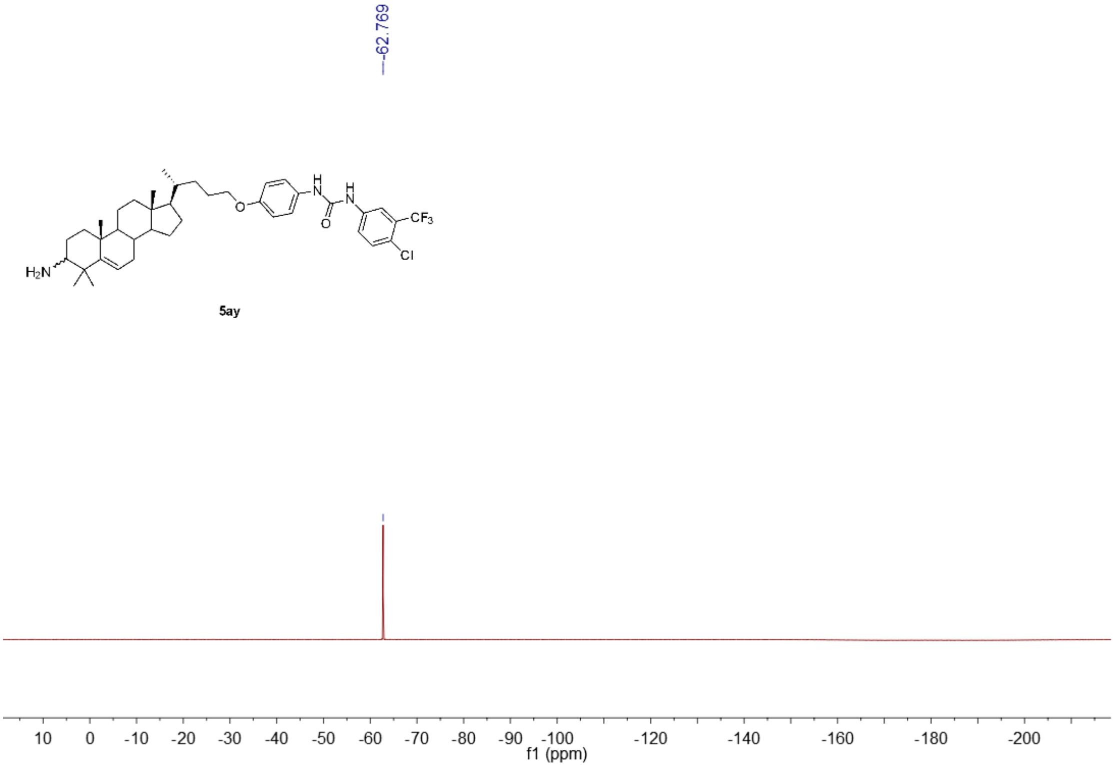

Compound **37** (^{1}H NMR)

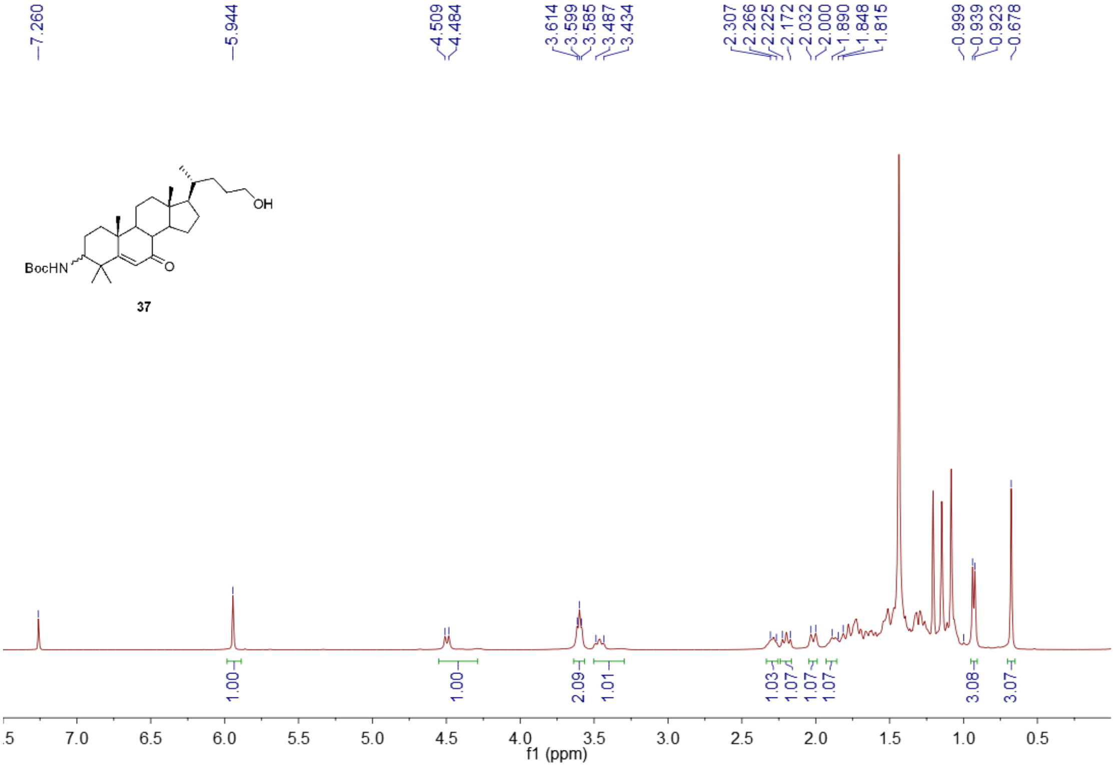

Compound **38** (^{1}H NMR)

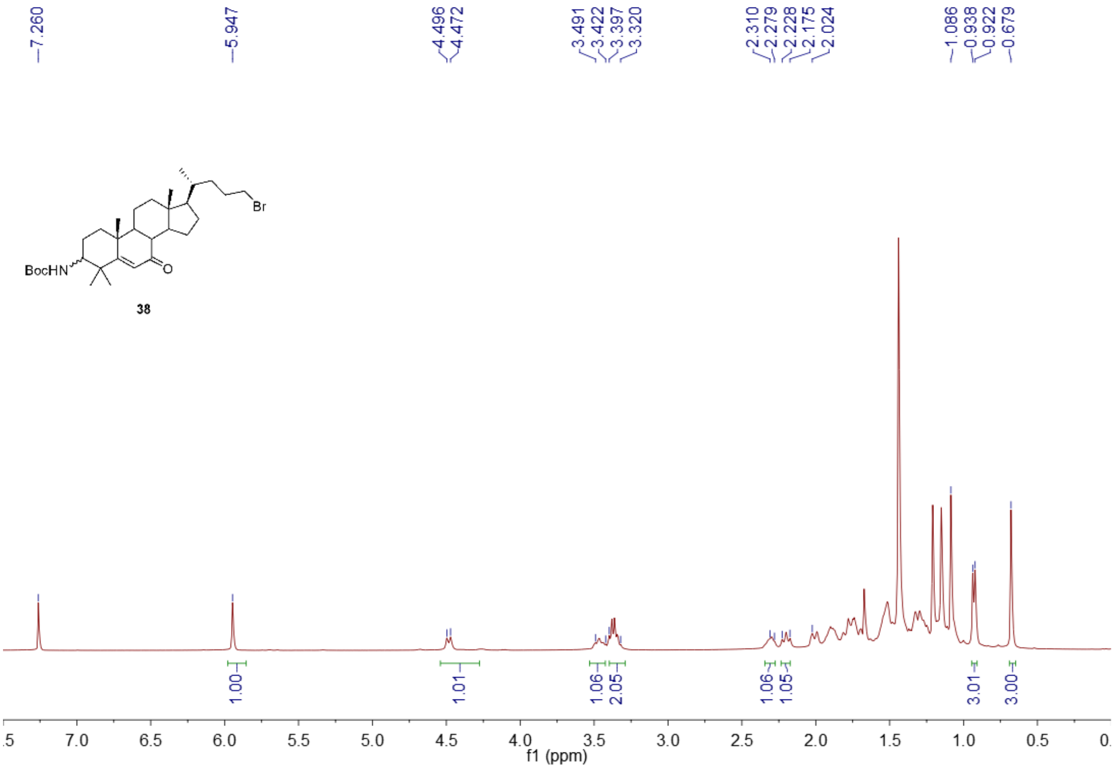

Compound **39** (^{1}H NMR)

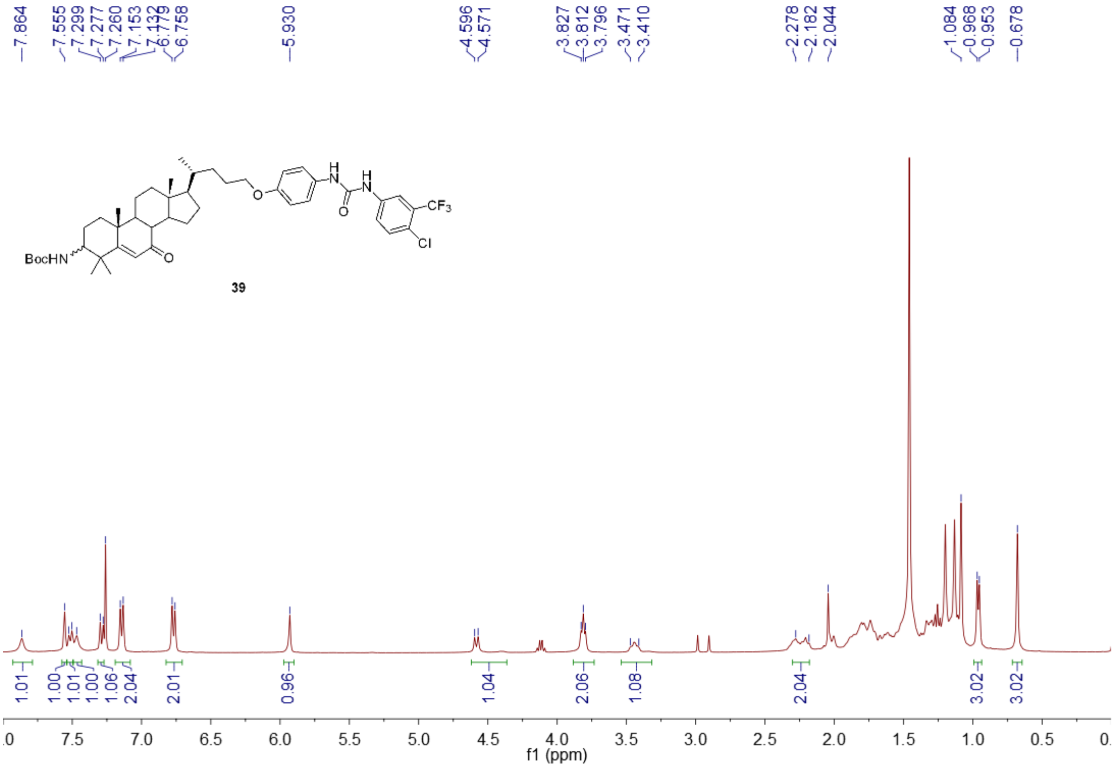

Compound **40** (^{1}H NMR)

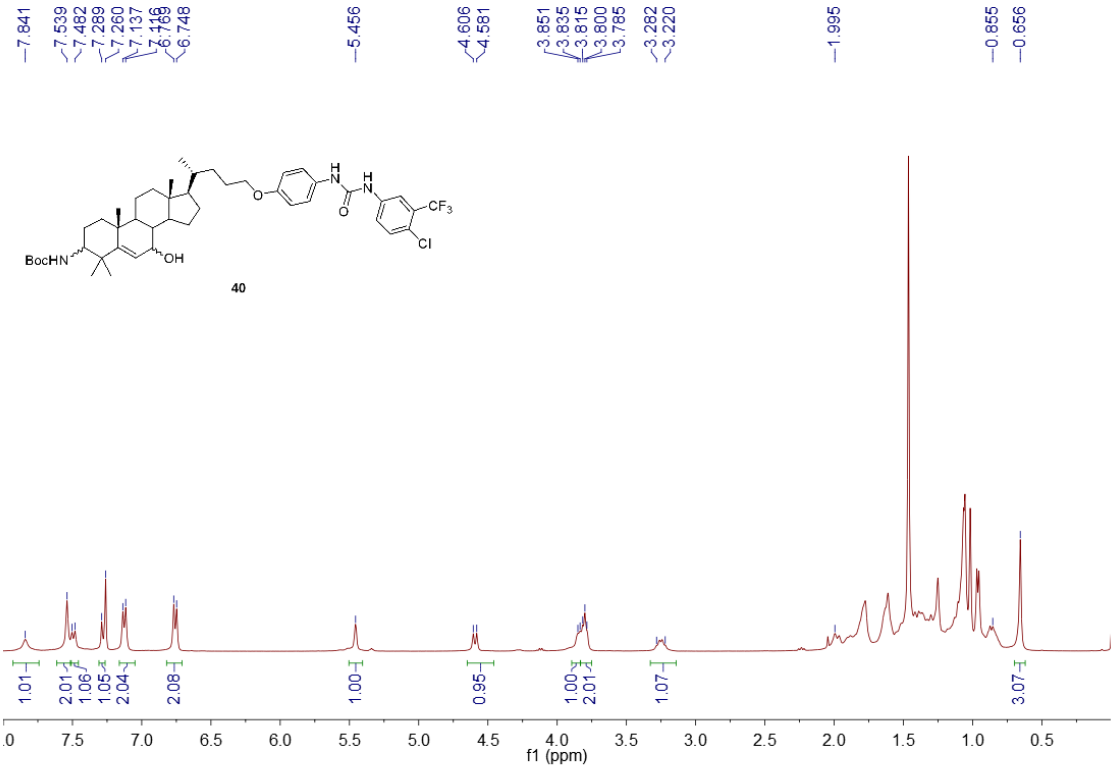

Compound **5az** (^{1}H NMR)

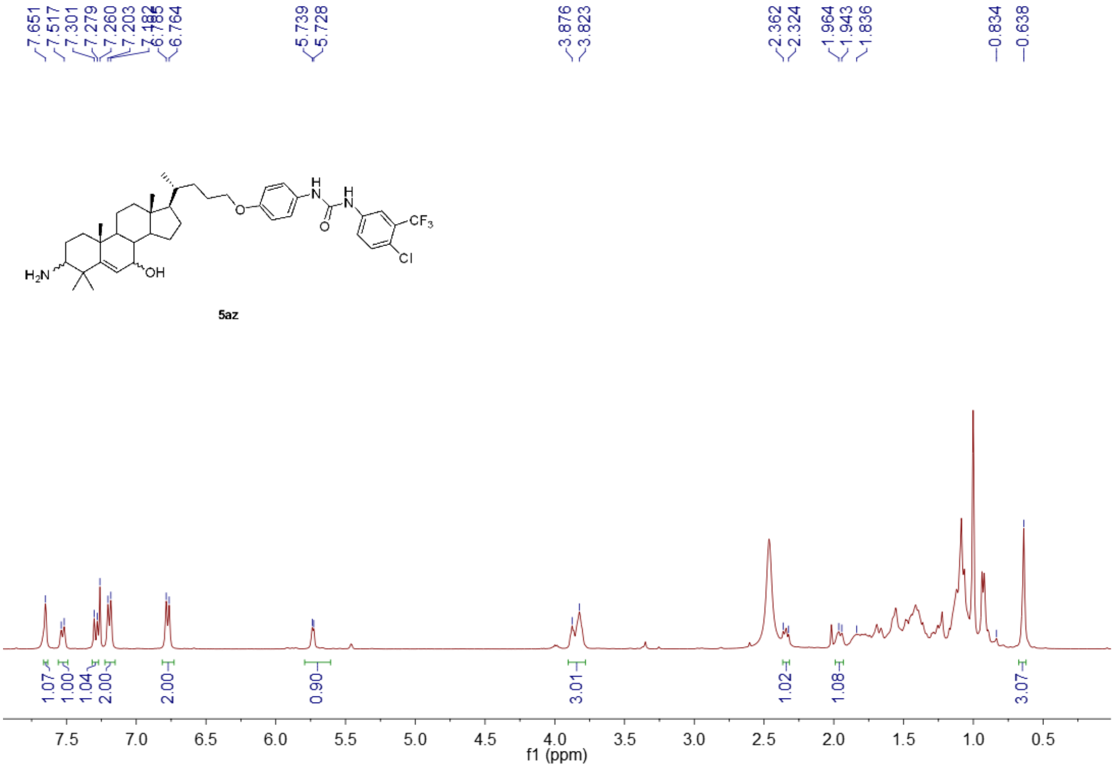

Compound **5az** (^{13}C NMR)

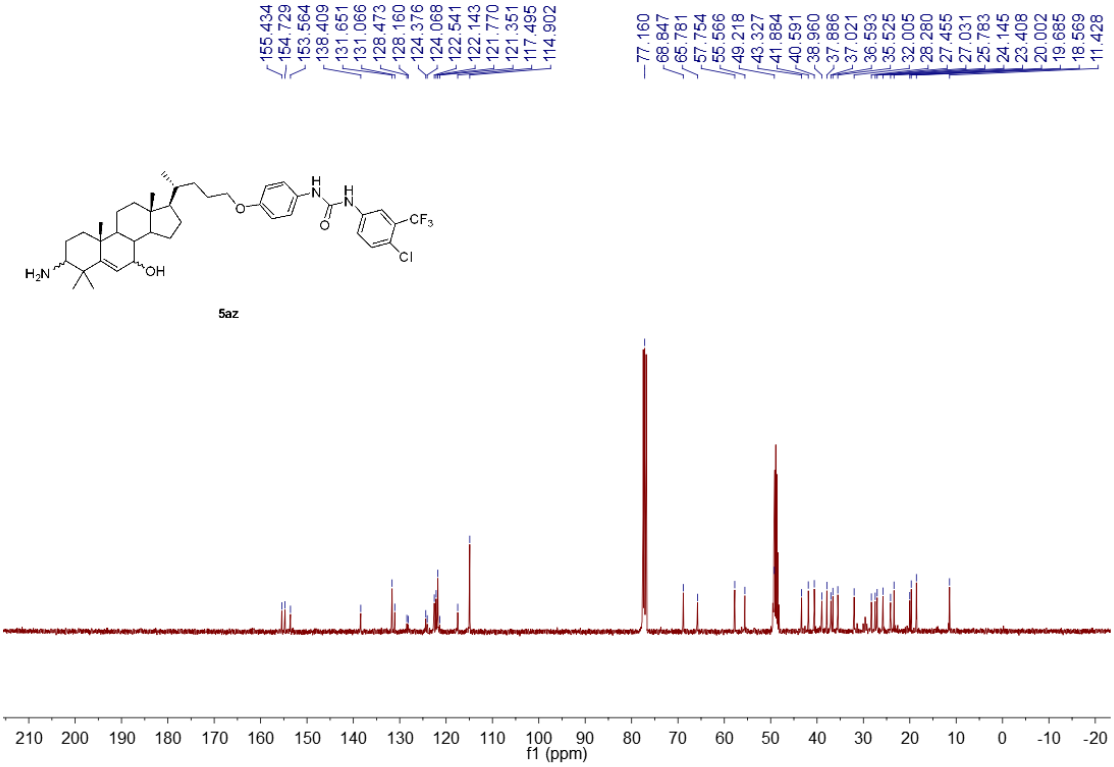

Compound **5az** (^{19}F NMR)

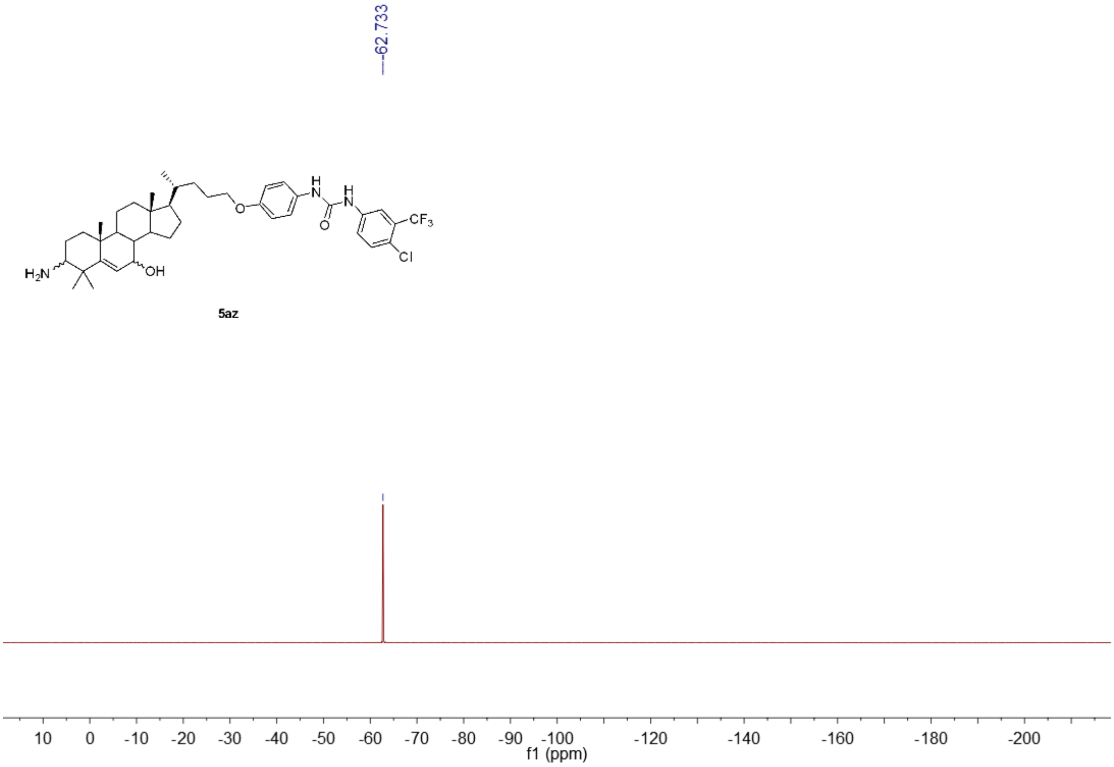

Compound **41** (^{1}H NMR)

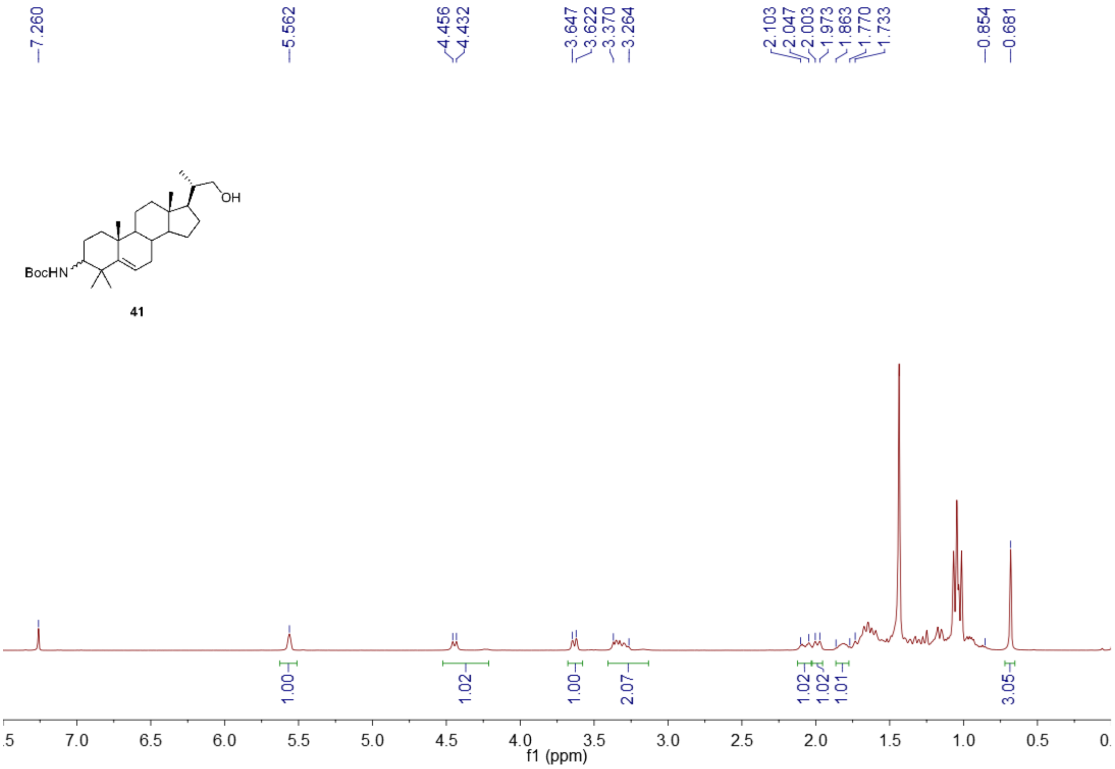

Compound **42** (^{1}H NMR)

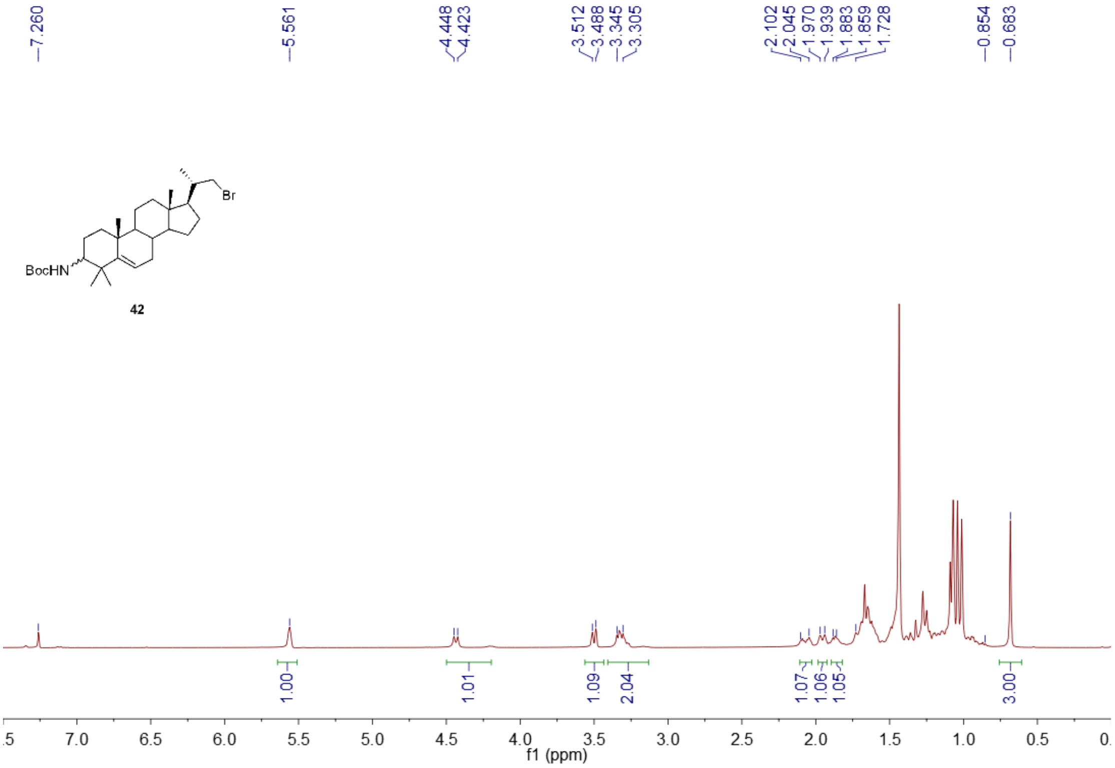

Compound **5ba** (^{1}H NMR)

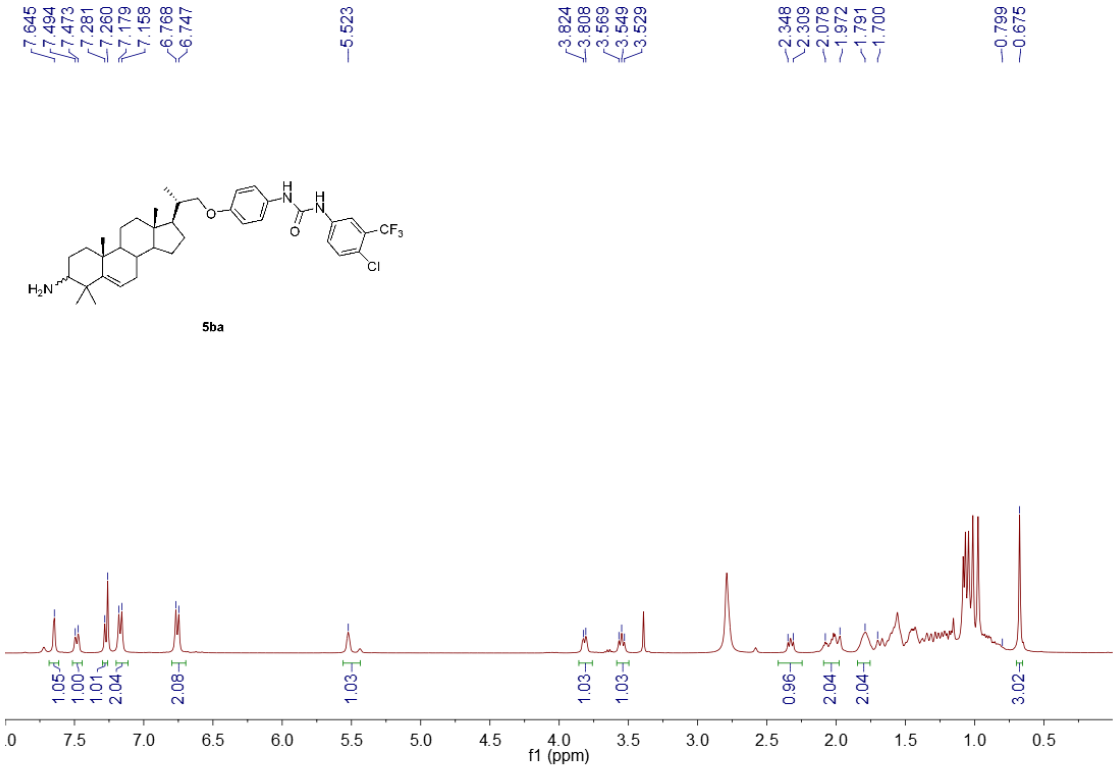

Compound **5ba** (^{13}C NMR)

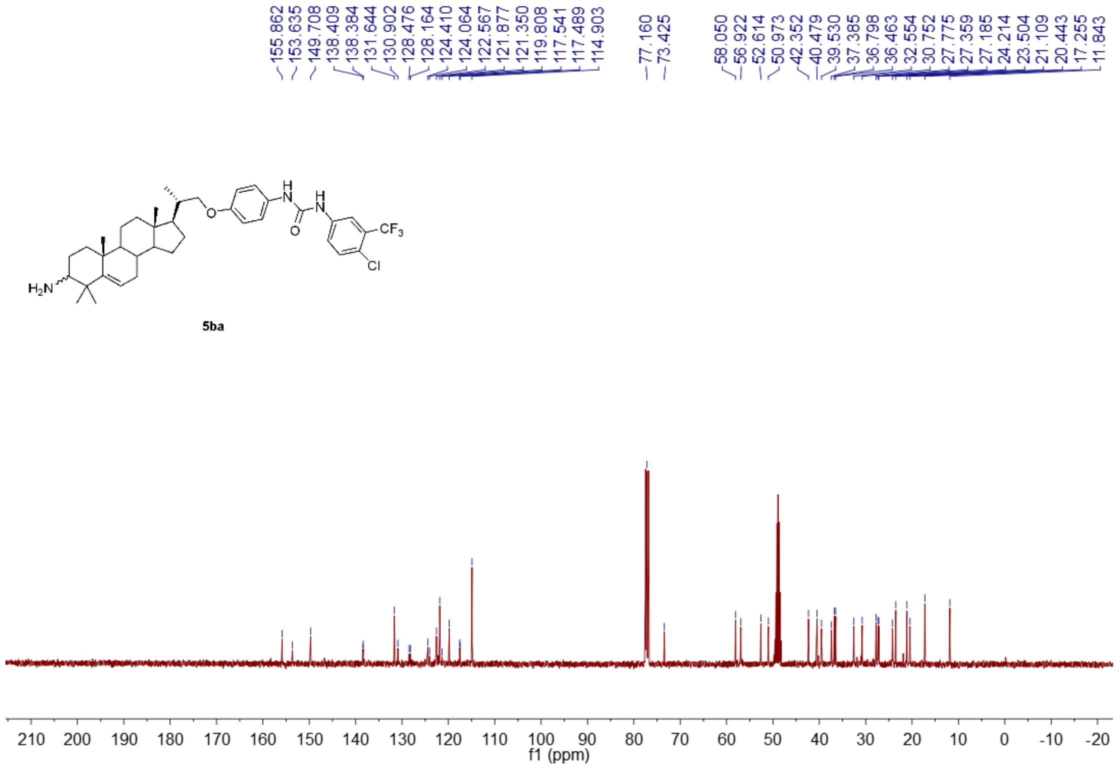

Compound **5ba** (^{19}F NMR)

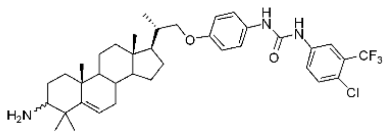

5ba

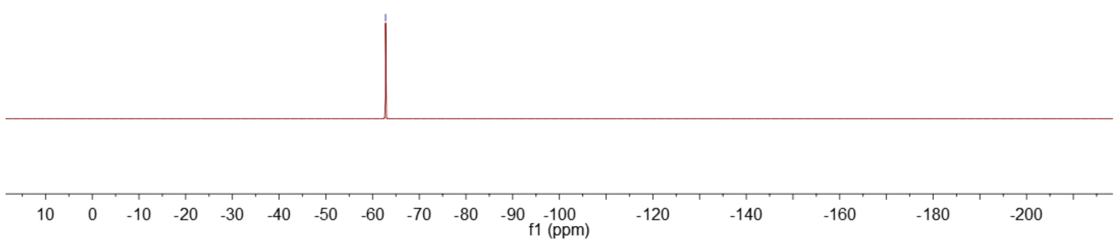

High-resolution mass spectroscopy

Compound **7** (HRMS)

Mass Spectrum SmartFormula Report

Analysis Info

Analysis Name	D:\Data\tj&yf-20221118\CHQ-7_36_01_4664.d	Acquisition Date	11/18/2022 12:09:28 PM
Method	new_tune_wide_3min_pos.m	Operator and analyser	ECNU.CHEM. G.D.Yang
Sample Name	CHQ-7	Instrument / Ser#	micrOTOF 10293
Comment			

Acquisition Parameter

Source Type	ESI	Ion Polarity	Positive	Set Nebulizer	1.5 Bar
Focus	Active			Set Dry Heater	180 °C
Scan Begin	100 m/z	Set Capillary	4500 V	Set Dry Gas	6.0 l/min
Scan End	3000 m/z	Set End Plate Offset	-500 V	Set Divert Valve	Waste

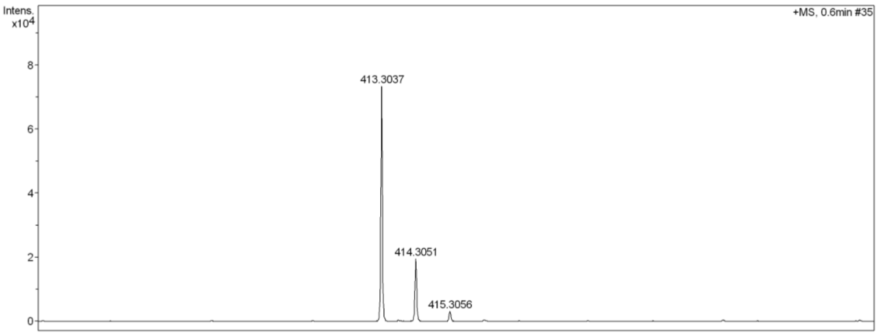

Meas. m/z	#	Formula	m/z	err [ppm]	Mean err [ppm]	rdb	N-Rule	e⁻ Conf	mSigma	Std I	Std Mean m/z	Std I VarNorm	Std m/z Diff	Std Comb Dev
413.3037														
	1	C 25 H 42 Na O 3	413.3026	-2.7	-1.3	4.5	ok	even	4.60	0.0065	0.0013	0.0029	0.0020	0.4978

Bruker Compass DataAnalysis 4.0 printed: 5 11/18/2022 3:35:28 PM Page1 of 1

Compound **8** (HRMS)

Mass Spectrum SmartFormula Report

Analysis Info

Analysis Name	D:\Data\tj&yf-20221205\8_28_01_4722.d	Acquisition Date	12/5/2022 3:15:48 PM
Method	new_tune_wide_3min_pos.m	Operator and analyser	ECNU.CHEM. G.D.Yang
Sample Name	8	Instrument / Ser#	micrOTOF 10293
Comment			

Acquisition Parameter

Source Type	ESI	Ion Polarity	Positive	Set Nebulizer	1.5 Bar
Focus	Active			Set Dry Heater	180 °C
Scan Begin	100 m/z	Set Capillary	4500 V	Set Dry Gas	6.0 l/min
Scan End	3000 m/z	Set End Plate Offset	-500 V	Set Divert Valve	Waste

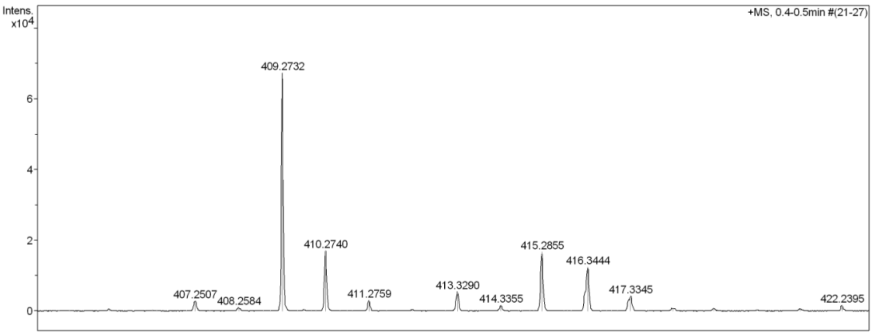

Meas. m/z	#	Formula	m/z	err [ppm]	Mean err [ppm]	rdb	N-Rule	e⁻ Conf	mSigma	Std I	Std Mean m/z	Std I VarNorm	Std m/z Diff	Std Comb Dev
409.2732														
	1	C 25 H 38 Na O 3	409.2713	-4.7	-3.1	6.5	ok	even	12.64	0.0178	0.0018	0.0081	0.0026	0.6783

Bruker Compass DataAnalysis 4.0 printed: 5 12/5/2022 4:52:15 PM Page1 of 1

Compound **9** (HRMS)

Mass Spectrum SmartFormula Report

Analysis Info

Analysis Name: D:\Data\tj&yf-20221118\CHQ-9_43_01_4671.d
Method: new_tune_wide_3min_pos.m
Sample Name: CHQ-9
Comment

Acquisition Date: 11/18/2022 12:41:53 PM
Operator and analyser: ECNU.CHEM. G.D.Yang
Instrument / Ser#: micrOTOF 10293

Acquisition Parameter

Source Type	ESI	Ion Polarity	Positive	Set Nebulizer	1.5 Bar
Focus	Active			Set Dry Heater	180 °C
Scan Begin	100 m/z	Set Capillary	4500 V	Set Dry Gas	6.0 l/min
Scan End	3000 m/z	Set End Plate Offset	-500 V	Set Divert Valve	Waste

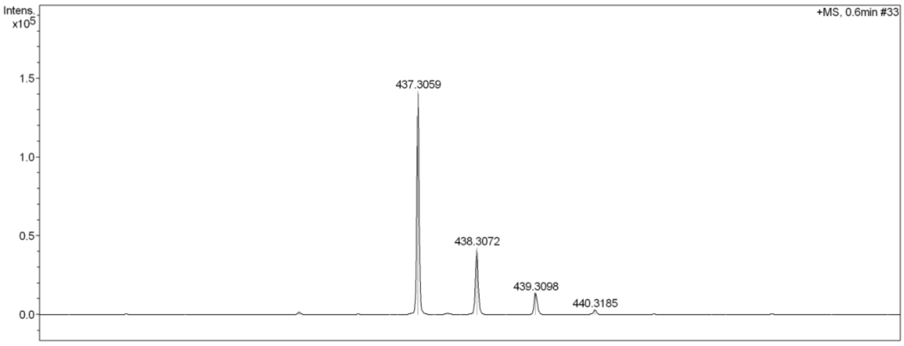

Meas. m/z	#	Formula	m/z	err [ppm]	Mean err [ppm]	rdb	N-Rule	e⁻ Conf	mSigma	Std I	Std Mean m/z	Std I VarNorm	Std m/z Diff	Std Comb Dev
437.3059														
	1	C 27 H 42 Na O 3	437.3026	-7.4	-6.1	6.5	ok	even	25.71	0.0499	0.0029	0.0222	0.0021	0.7069

Bruker Compass DataAnalysis 4.0 printed: 5 11/18/2022 3:38:18 PM Page1 of 1

Compound **5aa** (HRMS)

Mass Spectrum SmartFormula Report

Analysis Info

Analysis Name: D:\Data\tj&yf-20221130\CHQ-5aa_61_01_4693.d
Method: new_tune_wide_3min_pos.m
Sample Name: CHQ-5aa
Comment

Acquisition Date: 11/30/2022 3:44:11 PM
Operator and analyser: ECNU.CHEM. G.D.Yang
Instrument / Ser#: micrOTOF 10293

Acquisition Parameter

Source Type	ESI	Ion Polarity	Positive	Set Nebulizer	1.5 Bar
Focus	Active			Set Dry Heater	180 °C
Scan Begin	100 m/z	Set Capillary	4500 V	Set Dry Gas	6.0 l/min
Scan End	3000 m/z	Set End Plate Offset	-500 V	Set Divert Valve	Waste

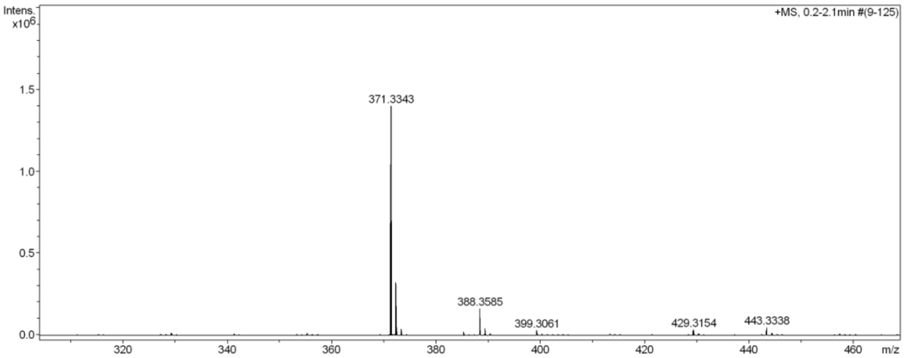

Meas. m/z	#	Formula	m/z	err [ppm]	Mean err [ppm]	rdb	N-Rule	e⁻ Conf	mSigma	Std I	Std Mean m/z	Std I VarNorm	Std m/z Diff	Std Comb Dev

Bruker Compass DataAnalysis 4.0 printed: 5 11/30/2022 3:51:07 PM Page1 of 1

Compound **11a** (HRMS)

Mass Spectrum SmartFormula Report

Analysis Info

Analysis Name	D:\Data\TJ&YF-20221125\CHQ-11a_60_01_4688.d	Acquisition Date	11/24/2022 3:05:14 PM
Method	new_tune_low_3min_pos.m	Operator and analyser	ECNU.CHEM. G.D.Yang
Sample Name	CHQ-11a	Instrument / Ser#	micrOTOF 10293
Comment			

Acquisition Parameter

Source Type	ESI	Ion Polarity	Positive	Set Nebulizer	1.5 Bar
Focus	Active			Set Dry Heater	180 °C
Scan Begin	50 m/z	Set Capillary	4500 V	Set Dry Gas	6.0 l/min
Scan End	3000 m/z	Set End Plate Offset	-500 V	Set Divert Valve	Waste

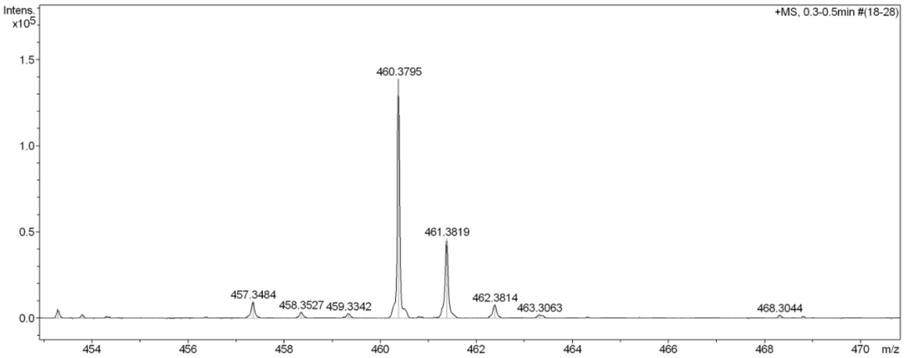

Meas. m/z	#	Formula	m/z	err [ppm]	Mean err [ppm]	rdb	N-Rule	e⁻ Conf	mSigma	Std I	Std Mean m/z	Std I VarNorm	Std m/z Diff	Std Comb Dev
460.3795														
	1	C 29 H 50 N O 3	460.3785	-2.1	-1.2	5.5	ok	even	5.49	0.0084	0.0011	0.0042	0.0029	0.5127

Bruker Compass DataAnalysis 4.0 printed: 5 11/25/2022 3:19:22 PM Page1 of 1

Compound **11b** (HRMS)

Mass Spectrum SmartFormula Report

Analysis Info

Analysis Name	D:\Data\tj&yf-20221118\CHQ-11b_33_01_4661.d	Acquisition Date	11/18/2022 11:55:46 AM
Method	new_tune_wide_3min_pos.m	Operator and analyser	ECNU.CHEM. G.D.Yang
Sample Name	CHQ-11b	Instrument / Ser#	micrOTOF 10293
Comment			

Acquisition Parameter

Source Type	ESI	Ion Polarity	Positive	Set Nebulizer	1.5 Bar
Focus	Active			Set Dry Heater	180 °C
Scan Begin	100 m/z	Set Capillary	4500 V	Set Dry Gas	6.0 l/min
Scan End	3000 m/z	Set End Plate Offset	-500 V	Set Divert Valve	Waste

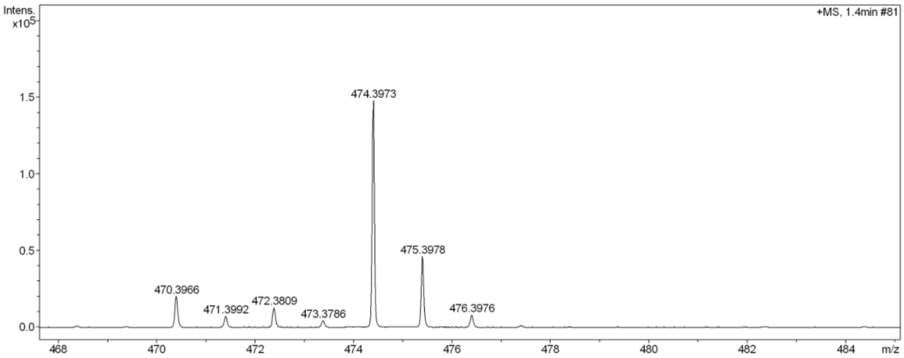

Meas. m/z	#	Formula	m/z	err [ppm]	Mean err [ppm]	rdb	N-Rule	e⁻ Conf	mSigma	Std I	Std Mean m/z	Std I VarNorm	Std m/z Diff	Std Comb Dev
474.3973														
	1	C 30 H 52 N O 3	474.3942	-6.6	-4.7	5.5	ok	even	12.06	0.0196	0.0028	0.0071	0.0041	0.7458

Bruker Compass DataAnalysis 4.0 printed: 5 11/18/2022 3:12:23 PM Page1 of 1

Compound **11c** (HRMS)

Mass Spectrum SmartFormula Report

Analysis Info

Analysis Name	D:\Data\TJ&YF-20220922\CHQ-15a_43_01_4603.d	Acquisition Date	9/22/2022 3:00:14 PM
Method	new_tune_wide_3min_pos.m	Operator and analyser	ECNU.CHEM. G.D.Yang
Sample Name	CHQ-15a	Instrument / Ser#	micrOTOF 10293
Comment			

Acquisition Parameter

Source Type	ESI	Ion Polarity	Positive	Set Nebulizer	1.5 Bar
Focus	Active			Set Dry Heater	180 °C
Scan Begin	100 m/z	Set Capillary	4500 V	Set Dry Gas	6.0 l/min
Scan End	3000 m/z	Set End Plate Offset	-500 V	Set Divert Valve	Waste

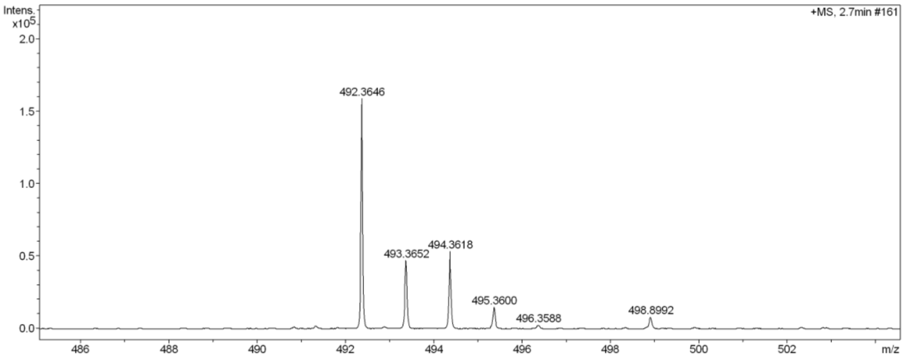

Meas. m/z	#	Formula	m/z	err [ppm]	Mean err [ppm]	rdb	N-Rule	e⁻ Conf	mSigma	Std I	Std Mean m/z	Std I VarNorm	Std m/z Diff	Std Comb Dev
492.3646														
	1	C 30 H 51 Cl N O 2	492.3603	-8.9	-7.1	5.5	ok	even	27.37	0.0378	0.0039	0.0111	0.0025	0.8952

Bruker Compass DataAnalysis 4.0 printed: 5 9/28/2022 2:57:31 PM Page1 of 1

Compound **11d** (HRMS)

Mass Spectrum SmartFormula Report

Analysis Info

Analysis Name	D:\Data\tj&yf-20221118\CHQ-11d_38_01_4666.d	Acquisition Date	11/18/2022 12:18:47 PM
Method	new_tune_wide_3min_pos.m	Operator and analyser	ECNU.CHEM. G.D.Yang
Sample Name	CHQ-11d	Instrument / Ser#	micrOTOF 10293
Comment			

Acquisition Parameter

Source Type	ESI	Ion Polarity	Positive	Set Nebulizer	1.5 Bar
Focus	Active			Set Dry Heater	180 °C
Scan Begin	100 m/z	Set Capillary	4500 V	Set Dry Gas	6.0 l/min
Scan End	3000 m/z	Set End Plate Offset	-500 V	Set Divert Valve	Waste

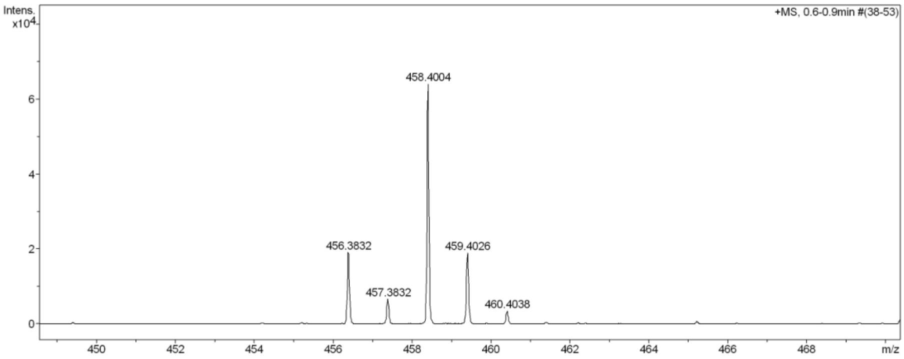

Meas. m/z	#	Formula	m/z	err [ppm]	Mean err [ppm]	rdb	N-Rule	e⁻ Conf	mSigma	Std I	Std Mean m/z	Std I VarNorm	Std m/z Diff	Std Comb Dev
458.4004														
	1	C 30 H 52 N O 2	458.3993	-2.5	-1.6	5.5	ok	even	18.82	0.0294	0.0011	0.0092	0.0020	0.7501

Bruker Compass DataAnalysis 4.0 printed: 5 11/18/2022 2:53:00 PM Page1 of 1

Compound **11e** (HRMS)

Mass Spectrum SmartFormula Report

Analysis Info

Analysis Name	D:\Data\tj&yf-20221118\CHQ-11e_31_01_4659.d	Acquisition Date	11/18/2022 11:46:44 AM
Method	new_tune_wide_3min_pos.m	Operator and analyser	ECNU.CHEM. G.D.Yang
Sample Name	CHQ-11e	Instrument / Ser#	micrOTOF 10293
Comment			

Acquisition Parameter

Source Type	ESI	Ion Polarity	Positive	Set Nebulizer	1.5 Bar
Focus	Active			Set Dry Heater	180 °C
Scan Begin	100 m/z	Set Capillary	4500 V	Set Dry Gas	6.0 l/min
Scan End	3000 m/z	Set End Plate Offset	-500 V	Set Divert Valve	Waste

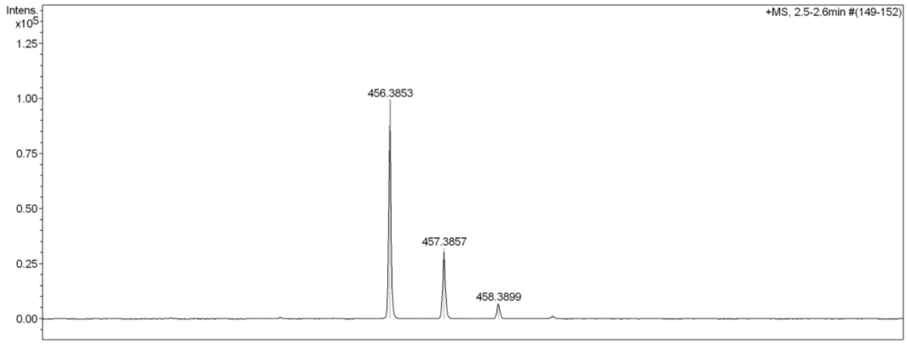

Meas. m/z	#	Formula	m/z	err [ppm]	Mean err [ppm]	rdb	N-Rule	e⁻ Conf	mSigma	Std I	Std Mean m/z	Std I VarNorm	Std m/z Diff	Std Comb Dev
456.3853														
	1	C 30 H 50 N O 2	456.3836	-3.7	-2.0	6.5	ok	even	9.12	0.0140	0.0016	0.0066	0.0025	0.8427

Bruker Compass DataAnalysis 4.0 printed: 5 11/18/2022 2:54:39 PM Page1 of 1

Compound **5ab** (HRMS)

Mass Spectrum SmartFormula Report

Analysis Info

Analysis Name	D:\Data\TJ&YF-20221125\CHQ-5ab_59_01_4687.d	Acquisition Date	11/24/2022 3:00:41 PM
Method	new_tune_low_3min_pos.m	Operator and analyser	ECNU.CHEM. G.D.Yang
Sample Name	CHQ-5ab	Instrument / Ser#	micrOTOF 10293
Comment			

Acquisition Parameter

Source Type	ESI	Ion Polarity	Positive	Set Nebulizer	1.5 Bar
Focus	Active			Set Dry Heater	180 °C
Scan Begin	50 m/z	Set Capillary	4500 V	Set Dry Gas	6.0 l/min
Scan End	3000 m/z	Set End Plate Offset	-500 V	Set Divert Valve	Waste

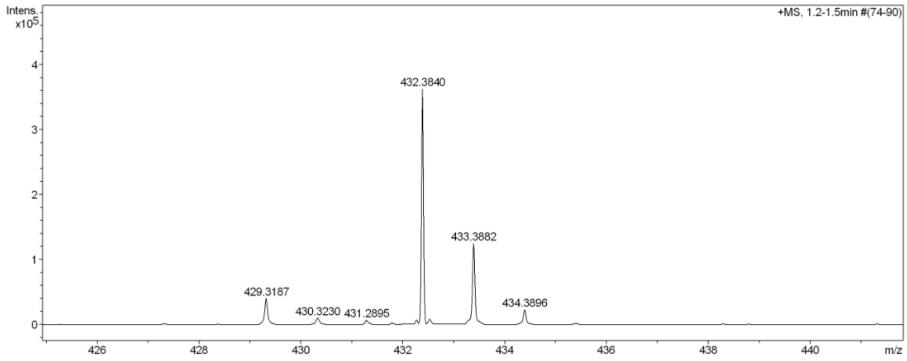

Meas. m/z	#	Formula	m/z	err [ppm]	Mean err [ppm]	rdb	N-Rule	e⁻ Conf	mSigma	Std I	Std Mean m/z	Std I VarNorm	Std m/z Diff	Std Comb Dev
432.3840														
	1	C 28 H 50 N O 2	432.3836	-0.9	-1.2	4.5	ok	even	17.73	0.0298	0.0007	0.0094	0.0011	0.4950

Bruker Compass DataAnalysis 4.0 printed: 5 11/25/2022 3:29:03 PM Page1 of 1

Compound **5ac** (HRMS)

Mass Spectrum SmartFormula Report

Analysis Info

Analysis Name	D:\Data\tj&yf-20221118\CHQ-5ac_35_01_4663.d	Acquisition Date	11/18/2022 12:04:51 PM
Method	new_tune_wide_3min_pos.m	Operator and analyser	ECNU.CHEM. G.D.Yang
Sample Name	CHQ-5ac	Instrument / Ser#	micrOTOF 10293
Comment			

Acquisition Parameter

Source Type	ESI	Ion Polarity	Positive	Set Nebulizer	1.5 Bar
Focus	Active			Set Dry Heater	180 °C
Scan Begin	100 m/z	Set Capillary	4500 V	Set Dry Gas	6.0 l/min
Scan End	3000 m/z	Set End Plate Offset	-500 V	Set Divert Valve	Waste

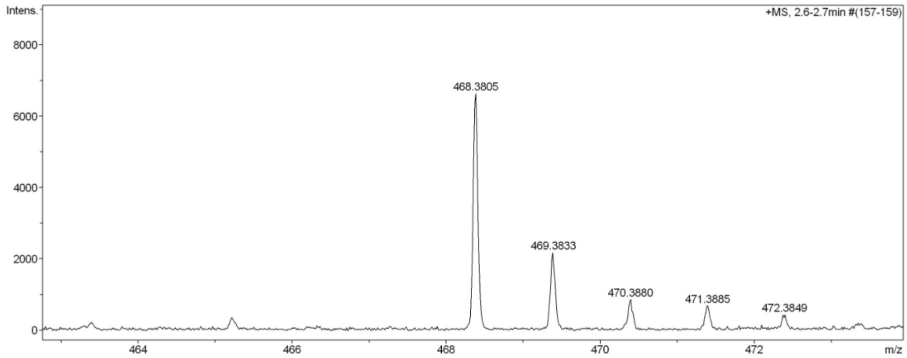

Meas. m/z	#	Formula	m/z	err [ppm]	Mean err [ppm]	rdb	N-Rule	e⁻ Conf	mSigma	Std I	Std Mean m/z	Std I VarNorm	Std m/z Diff	Std Comb Dev
468.3805														
	1	C 29 H 51 N Na O 2	468.3812	1.5	1.9	4.5	ok	even	62.11	0.1152	0.0011	0.0555	0.0008	0.6961

Bruker Compass DataAnalysis 4.0 printed: 5 11/18/2022 3:24:40 PM Page1 of 1

Compound **5ad** (HRMS)

Mass Spectrum SmartFormula Report

Analysis Info

Analysis Name	D:\Data\TJ&YF-20220922\CHQ-8aa_42_01_4602.d	Acquisition Date	9/22/2022 2:55:32 PM
Method	new_tune_wide_3min_pos.m	Operator and analyser	ECNU.CHEM. G.D.Yang
Sample Name	CHQ-8aa	Instrument / Ser#	micrOTOF 10293
Comment			

Acquisition Parameter

Source Type	ESI	Ion Polarity	Positive	Set Nebulizer	1.5 Bar
Focus	Active			Set Dry Heater	180 °C
Scan Begin	100 m/z	Set Capillary	4500 V	Set Dry Gas	6.0 l/min
Scan End	3000 m/z	Set End Plate Offset	-500 V	Set Divert Valve	Waste

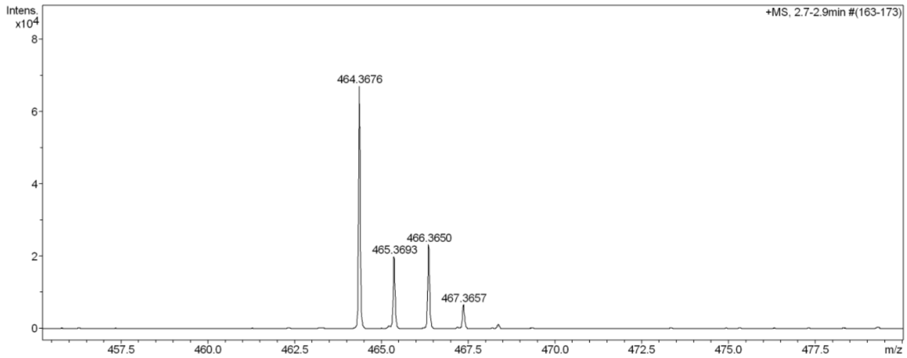

Meas. m/z	#	Formula	m/z	err [ppm]	Mean err [ppm]	rdb	N-Rule	e⁻ Conf	mSigma	Std I	Std Mean m/z	Std I VarNorm	Std m/z Diff	Std Comb Dev
464.3676														
	1	C 29 H 51 Cl N O	464.3654	-4.8	-4.0	4.5	ok	even	18.10	0.0244	0.0020	0.0091	0.0014	0.7540

Bruker Compass DataAnalysis 4.0 printed: 5 9/28/2022 3:26:08 PM Page1 of 1

Compound **5ae** (HRMS)

Mass Spectrum SmartFormula Report

Analysis Info

Analysis Name	D:\Data\tj&yf-20221130\CHQ-5ae_63_01_4695.d	Acquisition Date	11/30/2022 3:53:17 PM
Method	new_tune_wide_3min_pos.m	Operator and analyser	ECNU.CHEM. G.D.Yang
Sample Name	CHQ-5ae	Instrument / Ser#	micrOTOF 10293
Comment			

Acquisition Parameter

Source Type	ESI	Ion Polarity	Positive	Set Nebulizer	1.5 Bar
Focus	Active			Set Dry Heater	180 °C
Scan Begin	100 m/z	Set Capillary	4500 V	Set Dry Gas	6.0 l/min
Scan End	3000 m/z	Set End Plate Offset	-500 V	Set Divert Valve	Waste

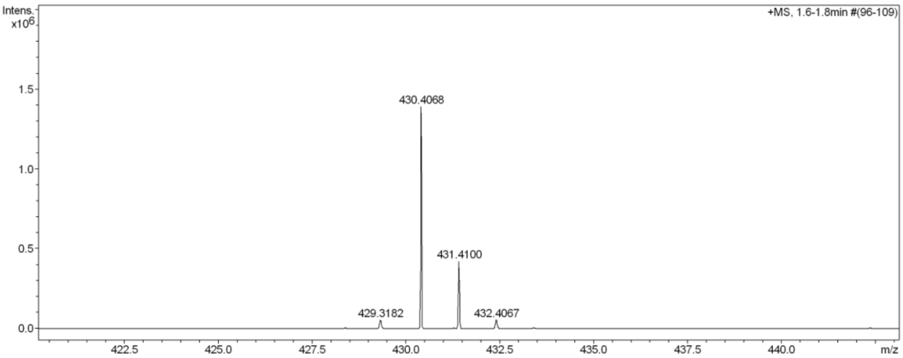

Meas. m/z	#	Formula	m/z	err [ppm]	Mean err [ppm]	rdb	N-Rule	e⁻ Conf	mSigma	Std I	Std Mean m/z	Std I VarNorm	Std m/z Diff	Std Comb Dev
430.4068														
	1	C 29 H 52 N O	430.4043	-5.7	-5.1	4.5	ok	even	12.13	0.0224	0.0026	0.0076	0.0041	0.7404

Bruker Compass DataAnalysis 4.0 printed: 5 12/1/2022 9:53:22 AM Page1 of 1

Compound **5af** (HRMS)

Mass Spectrum SmartFormula Report

Analysis Info

Analysis Name	D:\Data\tj&yf-20221118\CHQ-5af_32_01_4660.d	Acquisition Date	11/18/2022 11:51:18 AM
Method	new_tune_wide_3min_pos.m	Operator and analyser	ECNU.CHEM. G.D.Yang
Sample Name	CHQ-5af	Instrument / Ser#	micrOTOF 10293
Comment			

Acquisition Parameter

Source Type	ESI	Ion Polarity	Positive	Set Nebulizer	1.5 Bar
Focus	Active			Set Dry Heater	180 °C
Scan Begin	100 m/z	Set Capillary	4500 V	Set Dry Gas	6.0 l/min
Scan End	3000 m/z	Set End Plate Offset	-500 V	Set Divert Valve	Waste

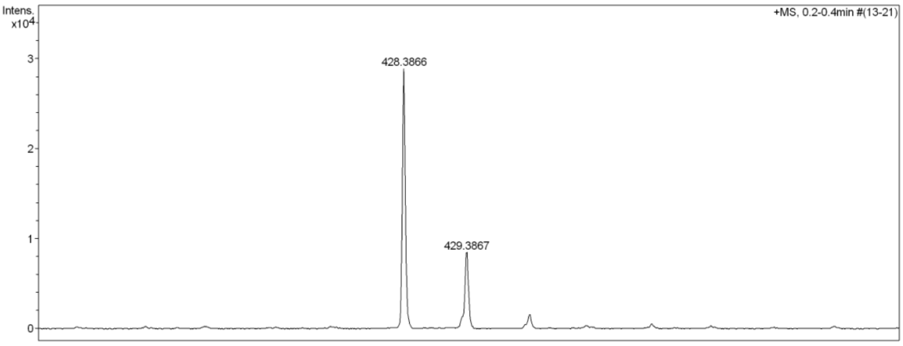

Meas. m/z	#	Formula	m/z	err [ppm]	Mean err [ppm]	rdb	N-Rule	e⁻ Conf	mSigma	Std I	Std Mean m/z	Std I VarNorm	Std m/z Diff	Std Comb Dev
428.3866														
	1	C 29 H 50 N O	428.3887	5.0	6.7	5.5	ok	even	29.34	0.0454	0.0032	0.0227	0.0032	0.8069

Bruker Compass DataAnalysis 4.0 printed: 5 11/18/2022 3:29:59 PM Page1 of 1

Compound **14** (HRMS)

Mass Spectrum SmartFormula Report

Analysis Info

Analysis Name	D:\Data\TJ&YF-20221028\CHQ-8_26_01_4633.d	Acquisition Date	10/28/2022 11:18:05 AM
Method	new_tune_wide_3min_pos.m	Operator and analyser	ECNU.CHEM. G.D.Yang
Sample Name	CHQ-8	Instrument / Ser#	micrOTOF 10293
Comment			

Acquisition Parameter

Source Type	ESI	Ion Polarity	Positive	Set Nebulizer	1.5 Bar
Focus	Active			Set Dry Heater	180 °C
Scan Begin	100 m/z	Set Capillary	4500 V	Set Dry Gas	6.0 l/min
Scan End	3000 m/z	Set End Plate Offset	-500 V	Set Divert Valve	Waste

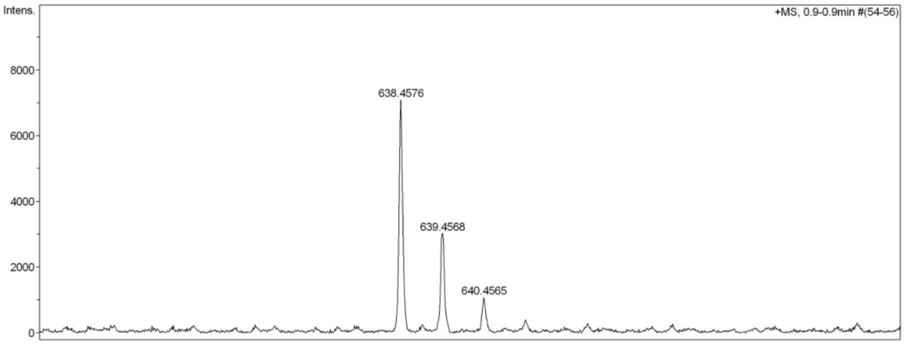

Meas. m/z	#	Formula	m/z	err [ppm]	Mean err [ppm]	rdb	N-Rule	e¯ Conf	mSigma	Std I	Std Mean m/z	Std I VarNorm	Std m/z Diff	Std Comb Dev
638.4576														
	1	C 37 H 65 N Na O 4 Si	638.4575	-0.2	2.6	6.5	ok	even	24.61	0.0298	0.0031	0.0167	0.0053	0.8036

Bruker Compass DataAnalysis 4.0 printed: 5 10/31/2022 2:26:50 PM Page1 of 1

Compound **5ag** (HRMS)

Mass Spectrum SmartFormula Report

Analysis Info

Analysis Name	D:\Data\TJ&YF-20221028\CHQ-5ag_32_01_4640.d	Acquisition Date	10/28/2022 12:39:57 PM
Method	new_tune_wide_3min_pos.m	Operator and analyser	ECNU.CHEM. G.D.Yang
Sample Name	CHQ-5ag	Instrument / Ser#	micrOTOF 10293
Comment			

Acquisition Parameter

Source Type	ESI	Ion Polarity	Positive	Set Nebulizer	1.5 Bar
Focus	Active			Set Dry Heater	180 °C
Scan Begin	100 m/z	Set Capillary	4500 V	Set Dry Gas	6.0 l/min
Scan End	3000 m/z	Set End Plate Offset	-500 V	Set Divert Valve	Waste

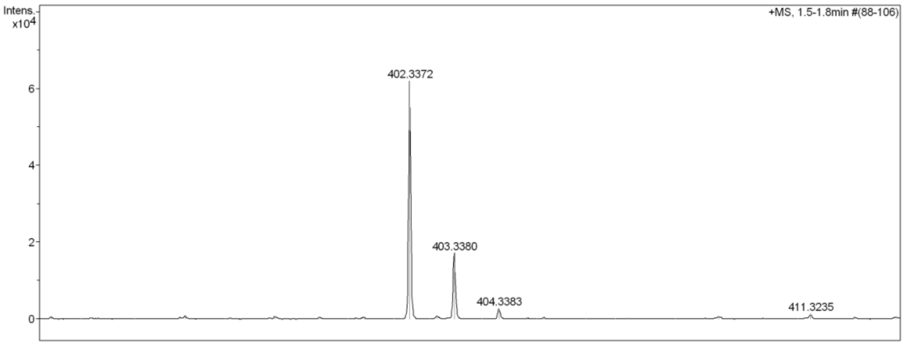

Meas. m/z	#	Formula	m/z	err [ppm]	Mean err [ppm]	rdb	N-Rule	e¯ Conf	mSigma	Std I	Std Mean m/z	Std I VarNorm	Std m/z Diff	Std Comb Dev
402.3372														
	1	C 26 H 44 N O 2	402.3367	-1.2	0.5	5.5	ok	even	7.62	0.0107	0.0014	0.0047	0.0025	0.5563

Bruker Compass DataAnalysis 4.0 printed: 5 10/31/2022 2:22:36 PM Page1 of 1

Compound **5ah** (HRMS)

Mass Spectrum SmartFormula Report

Analysis Info

Analysis Name	D:\Data\tj&yf-20221130\CHQ-5ah_70_01_4702.d	Acquisition Date	11/30/2022 4:25:32 PM
Method	new_tune_wide_3min_pos.m	Operator and analyser	ECNU.CHEM. G.D.Yang
Sample Name	CHQ-5ah	Instrument / Ser#	micrOTOF 10293
Comment			

Acquisition Parameter

Source Type	ESI	Ion Polarity	Positive	Set Nebulizer	1.5 Bar
Focus	Active			Set Dry Heater	180 °C
Scan Begin	100 m/z	Set Capillary	4500 V	Set Dry Gas	6.0 l/min
Scan End	3000 m/z	Set End Plate Offset	-500 V	Set Divert Valve	Waste

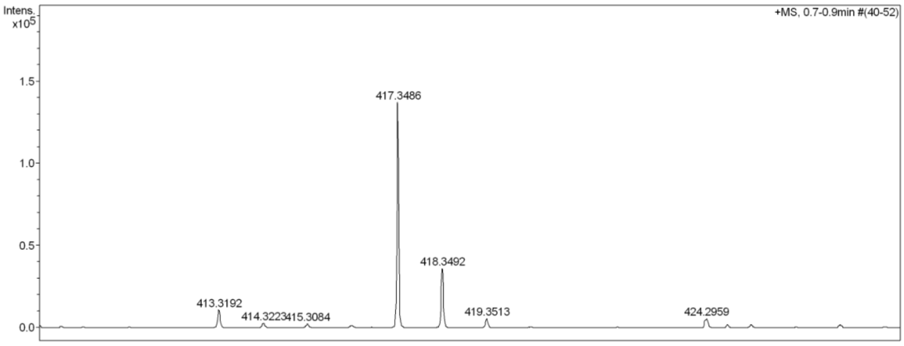

Meas. m/z	#	Formula	m/z	err [ppm]	Mean err [ppm]	rdb	N-Rule	e⁻ Conf	mSigma	Std I	Std Mean m/z	Std I VarNorm	Std m/z Diff	Std Comb Dev
417.3486														
	1	C 26 H 45 N 2 O 2	417.3476	-2.6	-0.9	5.5	ok	even	18.24	0.0276	0.0013	0.0119	0.0028	0.7891

Bruker Compass DataAnalysis 4.0 printed: 5 12/1/2022 9:57:32 AM Page1 of 1

Compound **5ai** (HRMS)

Mass Spectrum SmartFormula Report

Analysis Info

Analysis Name	D:\Data\tj&yf-20221130\CHQ-5ai_65_01_4697.d	Acquisition Date	11/30/2022 4:02:26 PM
Method	new_tune_wide_3min_pos.m	Operator and analyser	ECNU.CHEM. G.D.Yang
Sample Name	CHQ-5ai	Instrument / Ser#	micrOTOF 10293
Comment			

Acquisition Parameter

Source Type	ESI	Ion Polarity	Positive	Set Nebulizer	1.5 Bar
Focus	Active			Set Dry Heater	180 °C
Scan Begin	100 m/z	Set Capillary	4500 V	Set Dry Gas	6.0 l/min
Scan End	3000 m/z	Set End Plate Offset	-500 V	Set Divert Valve	Waste

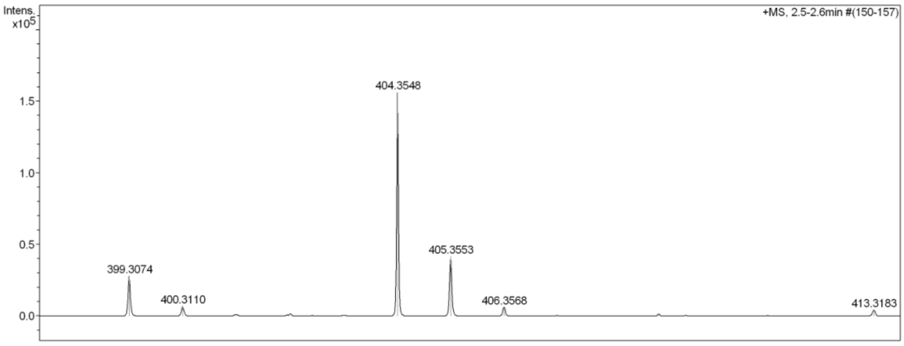

Meas. m/z	#	Formula	m/z	err [ppm]	Mean err [ppm]	rdb	N-Rule	e⁻ Conf	mSigma	Std I	Std Mean m/z	Std I VarNorm	Std m/z Diff	Std Comb Dev
404.3548														
	1	C 26 H 46 N O 2	404.3523	-6.2	-4.4	4.5	ok	even	13.69	0.0210	0.0022	0.0091	0.0029	0.7521

Bruker Compass DataAnalysis 4.0 printed: 5 12/1/2022 9:59:58 AM Page1 of 1

Compound **15** (HRMS)

Mass Spectrum SmartFormula Report

Analysis Info

Analysis Name	D:\Data\TJ&YF-20220922\CHQ-23_40_01_4600.d	Acquisition Date	9/22/2022 2:46:13 PM
Method	new_tune_wide_3min_pos.m	Operator and analyser	ECNU.CHEM. G.D.Yang
Sample Name	CHQ-23	Instrument / Ser#	micrOTOF 10293
Comment			

Acquisition Parameter

Source Type	ESI	Ion Polarity	Positive	Set Nebulizer	1.5 Bar
Focus	Active			Set Dry Heater	180 °C
Scan Begin	100 m/z	Set Capillary	4500 V	Set Dry Gas	6.0 l/min
Scan End	3000 m/z	Set End Plate Offset	-500 V	Set Divert Valve	Waste

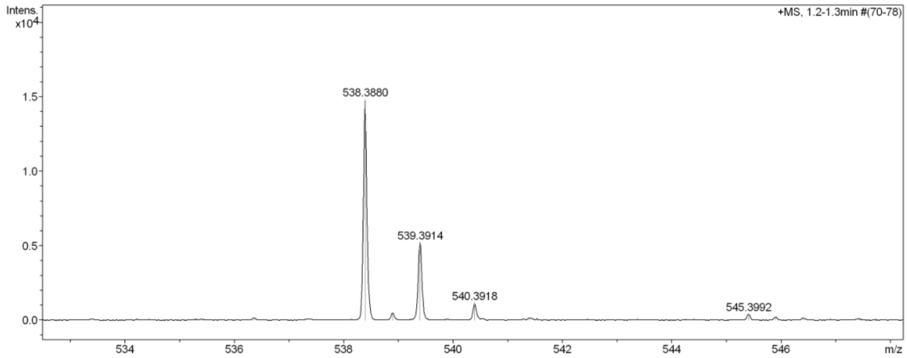

Meas. m/z	#	Formula	m/z	err [ppm]	Mean err [ppm]	rdb	N-Rule	e⁻ Conf	mSigma	Std I	Std Mean m/z	Std I VarNorm	Std m/z Diff	Std Comb Dev
538.3880														
	1	C 32 H 53 N Na O 4	538.3867	-2.5	-2.2	6.5	ok	even	4.82	0.0078	0.0014	0.0036	0.0019	0.5357

Bruker Compass DataAnalysis 4.0 printed: 5 9/28/2022 3:07:09 PM Page1 of 1

Compound **16** (HRMS)

Mass Spectrum SmartFormula Report

Analysis Info

Analysis Name	D:\Data\tj&yf-20220928\CHQ 34_47_01_4608.d	Acquisition Date	9/28/2022 2:34:23 PM
Method	new_tune_wide_3min_pos.m	Operator and analyser	ECNU.CHEM. G.D.Yang
Sample Name	CHQ 34	Instrument / Ser#	micrOTOF 10293
Comment			

Acquisition Parameter

Source Type	ESI	Ion Polarity	Positive	Set Nebulizer	1.5 Bar
Focus	Active			Set Dry Heater	180 °C
Scan Begin	100 m/z	Set Capillary	4500 V	Set Dry Gas	6.0 l/min
Scan End	3000 m/z	Set End Plate Offset	-500 V	Set Divert Valve	Waste

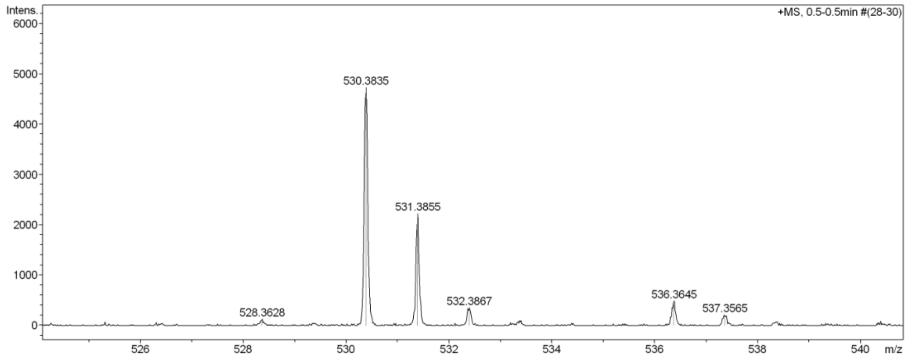

Meas. m/z	#	Formula	m/z	err [ppm]	Mean err [ppm]	rdb	N-Rule	e⁻ Conf	mSigma	Std I	Std Mean m/z	Std I VarNorm	Std m/z Diff	Std Comb Dev
530.3835														
	1	C 32 H 52 N O 5	530.3840	0.9	2.0	7.5	ok	even	57.29	0.0768	0.0014	0.0248	0.0022	0.6302

Bruker Compass DataAnalysis 4.0 printed: 5 9/28/2022 2:47:18 PM Page1 of 1

Compound **18** (HRMS)

Mass Spectrum SmartFormula Report

Analysis Info

Analysis Name	D:\Data\TJ&YF-20221028\CHQ-37_12_01_4619.d	Acquisition Date	10/28/2022 9:53:20 AM
Method	new_tune_wide_3min_pos.m	Operator and analyser	ECNU.CHEM. G.D.Yang
Sample Name	CHQ-37	Instrument / Ser#	micrOTOF 10293
Comment			

Acquisition Parameter

Source Type	ESI	Ion Polarity	Positive	Set Nebulizer	1.5 Bar
Focus	Active			Set Dry Heater	180 °C
Scan Begin	100 m/z	Set Capillary	4500 V	Set Dry Gas	6.0 l/min
Scan End	3000 m/z	Set End Plate Offset	-500 V	Set Divert Valve	Waste

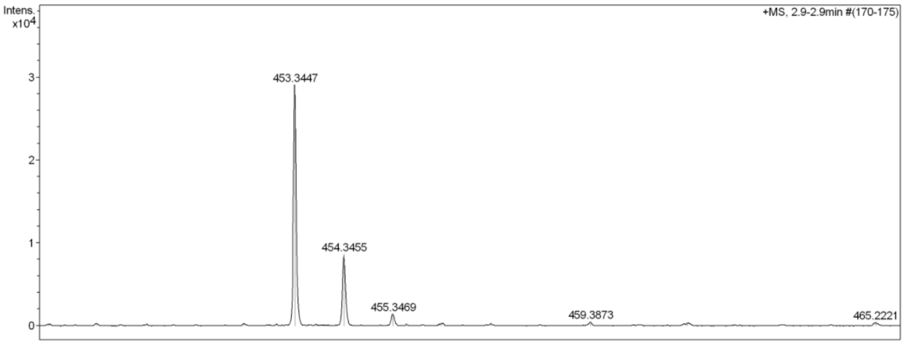

Meas. m/z	#	Formula	m/z	err [ppm]	Mean err [ppm]	rdb	N-Rule	e⁻ Conf	mSigma	Std I	Std Mean m/z	Std I VarNorm	Std m/z Diff	Std Comb Dev
453.3447														
	1	C 27 H 46 N 2 Na O 2	453.3451	1.0	2.9	5.5	ok	even	4.82	0.0069	0.0023	0.0035	0.0031	0.5698

Bruker Compass DataAnalysis 4.0 printed: 5 10/28/2022 12:42:37 PM Page1 of 1

Compound **19** (HRMS)

Mass Spectrum SmartFormula Report

Analysis Info

Analysis Name	D:\Data\TJ&YF-20221028\CHQ-196_30_01_4637.d	Acquisition Date	10/28/2022 11:36:47 AM
Method	new_tune_wide_3min_pos.m	Operator and analyser	ECNU.CHEM. G.D.Yang
Sample Name	CHQ-196	Instrument / Ser#	micrOTOF 10293
Comment			

Acquisition Parameter

Source Type	ESI	Ion Polarity	Positive	Set Nebulizer	1.5 Bar
Focus	Active			Set Dry Heater	180 °C
Scan Begin	100 m/z	Set Capillary	4500 V	Set Dry Gas	6.0 l/min
Scan End	3000 m/z	Set End Plate Offset	-500 V	Set Divert Valve	Waste

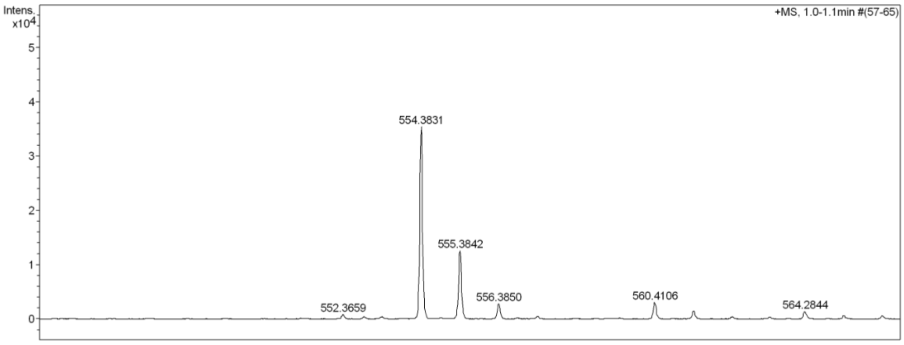

Meas. m/z	#	Formula	m/z	err [ppm]	Mean err [ppm]	rdb	N-Rule	e⁻ Conf	mSigma	Std I	Std Mean m/z	Std I VarNorm	Std m/z Diff	Std Comb Dev
554.3831														
	1	C 32 H 53 N Na O 5	554.3816	-2.7	-1.2	6.5	ok	even	4.65	0.0083	0.0016	0.0037	0.0032	0.5393

Bruker Compass DataAnalysis 4.0 printed: 5 10/31/2022 3:27:47 PM Page1 of 1

Compound **5ak** (HRMS)

Mass Spectrum SmartFormula Report

Analysis Info

Analysis Name	D:\Data\tj&yf-20221130\CHQ-5ak_62_01_4694.d	Acquisition Date	11/30/2022 3:48:44 PM
Method	new_tune_wide_3min_pos.m	Operator and analyser	ECNU.CHEM. G.D.Yang
Sample Name	CHQ-5ak	Instrument / Ser#	micrOTOF 10293
Comment			

Acquisition Parameter

Source Type	ESI	Ion Polarity	Positive	Set Nebulizer	1.5 Bar
Focus	Active			Set Dry Heater	180 °C
Scan Begin	100 m/z	Set Capillary	4500 V	Set Dry Gas	6.0 l/min
Scan End	3000 m/z	Set End Plate Offset	-500 V	Set Divert Valve	Waste

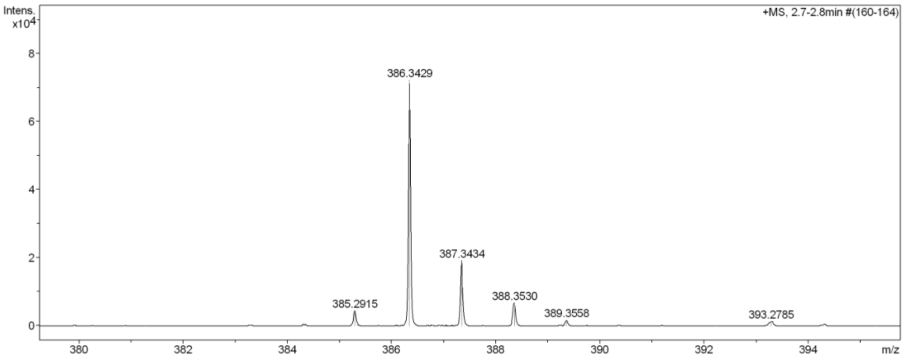

Meas. m/z	#	Formula	m/z	err [ppm]	Mean err [ppm]	rdb	N-Rule	e⁻ Conf	mSigma	Std I	Std Mean m/z	Std I VarNorm	Std m/z Diff	Std Comb Dev
386.3429	1	C 26 H 44 N O	386.3417	-3.0	-2.2	5.5	ok	even	33.72	0.0473	0.0017	0.0330	0.0028	0.7091

Bruker Compass DataAnalysis 4.0 printed: 5 12/1/2022 10:06:55 AM Page1 of 1

Compound **22** (HRMS)

Mass Spectrum SmartFormula Report

Analysis Info

Analysis Name	D:\Data\TJ&YF-20221028\CHQ-28_19_01_4626.d	Acquisition Date	10/28/2022 10:25:32 AM
Method	new_tune_wide_3min_pos.m	Operator and analyser	ECNU.CHEM. G.D.Yang
Sample Name	CHQ-28	Instrument / Ser#	micrOTOF 10293
Comment			

Acquisition Parameter

Source Type	ESI	Ion Polarity	Positive	Set Nebulizer	1.5 Bar
Focus	Active			Set Dry Heater	180 °C
Scan Begin	100 m/z	Set Capillary	4500 V	Set Dry Gas	6.0 l/min
Scan End	3000 m/z	Set End Plate Offset	-500 V	Set Divert Valve	Waste

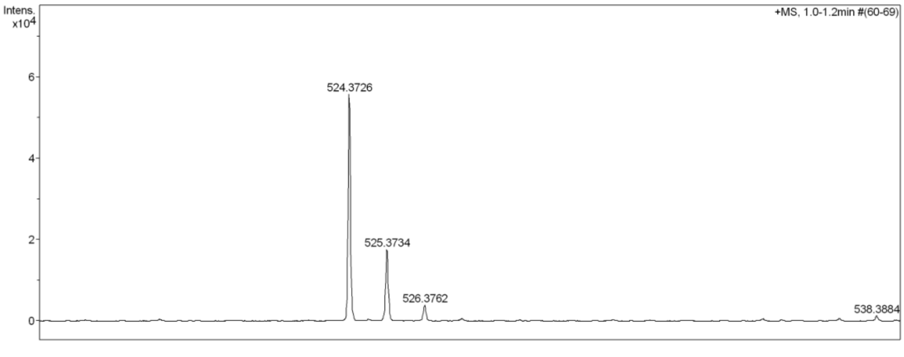

Meas. m/z	#	Formula	m/z	err [ppm]	Mean err [ppm]	rdb	N-Rule	e⁻ Conf	mSigma	Std I	Std Mean m/z	Std I VarNorm	Std m/z Diff	Std Comb Dev
524.3726	1	C 31 H 51 N Na O 4	524.3710	-2.9	-1.4	6.5	ok	even	14.74	0.0209	0.0017	0.0071	0.0025	0.6563

Bruker Compass DataAnalysis 4.0 printed: 5 10/28/2022 11:00:01 AM Page1 of 1

Compound **23** (HRMS)

Mass Spectrum SmartFormula Report

Analysis Info

Analysis Name	D:\Data\TJ&YF-20221028\CHQ-29_16_01_4623.d	Acquisition Date	10/28/2022 10:11:40 AM
Method	new_tune_wide_3min_pos.m	Operator and analyser	ECNU.CHEM. G.D.Yang
Sample Name	CHQ-29	Instrument / Ser#	micrOTOF 10293
Comment			

Acquisition Parameter

Source Type	ESI	Ion Polarity	Positive	Set Nebulizer	1.5 Bar
Focus	Active			Set Dry Heater	180 °C
Scan Begin	100 m/z	Set Capillary	4500 V	Set Dry Gas	6.0 l/min
Scan End	3000 m/z	Set End Plate Offset	-500 V	Set Divert Valve	Waste

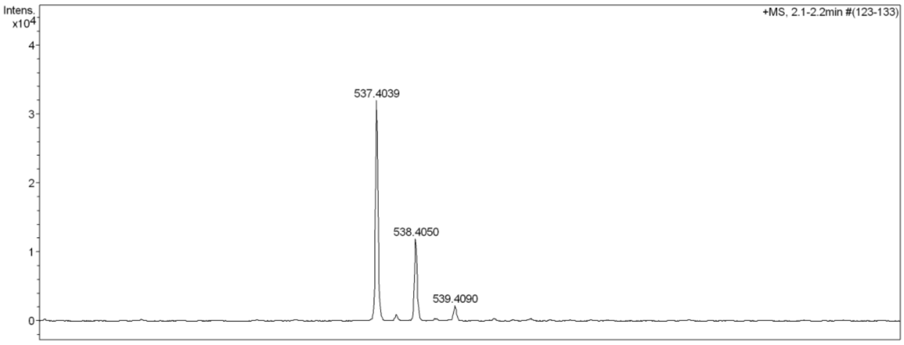

Meas. m/z	#	Formula	m/z	err [ppm]	Mean err [ppm]	rdb	N-Rule	e⁻ Conf	mSigma	Std I	Std Mean m/z	Std I VarNorm	Std m/z Diff	Std Comb Dev
537.4039														
	1	C 32 H 54 N 2 Na O 3	537.4027	-2.2	-1.0	6.5	ok	even	7.34	0.0113	0.0011	0.0036	0.0019	0.5556

Bruker Compass DataAnalysis 4.0 printed: 5 10/28/2022 11:02:50 AM Page1 of 1

Compound **5al** (HRMS)

Mass Spectrum SmartFormula Report

Analysis Info

Analysis Name	D:\Data\TJ&YF-20221202\5al_14_01_4709.d	Acquisition Date	12/2/2022 10:29:57 AM
Method	new_tune_wide_3min_pos.m	Operator and analyser	ECNU.CHEM. G.D.Yang
Sample Name	5al	Instrument / Ser#	micrOTOF 10293
Comment			

Acquisition Parameter

Source Type	ESI	Ion Polarity	Positive	Set Nebulizer	1.5 Bar
Focus	Active			Set Dry Heater	180 °C
Scan Begin	100 m/z	Set Capillary	4500 V	Set Dry Gas	6.0 l/min
Scan End	3000 m/z	Set End Plate Offset	-500 V	Set Divert Valve	Waste

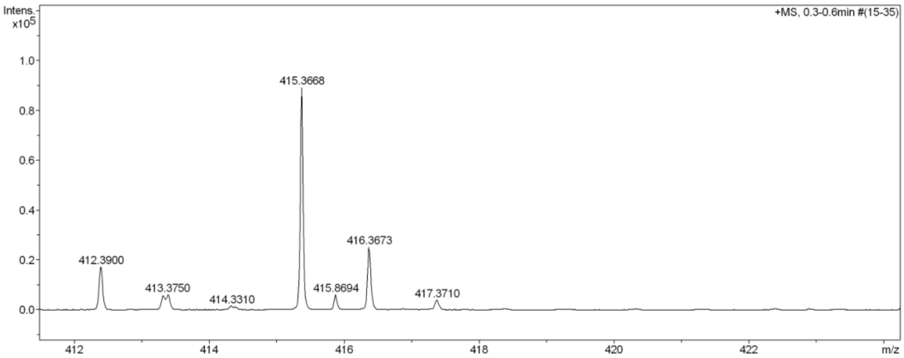

Meas. m/z	#	Formula	m/z	err [ppm]	Mean err [ppm]	rdb	N-Rule	e⁻ Conf	mSigma	Std I	Std Mean m/z	Std I VarNorm	Std m/z Diff	Std Comb Dev
415.3668														
	1	C 27 H 47 N 2 O	415.3683	3.5	5.2	5.5	ok	even	11.19	0.0182	0.0025	0.0060	0.0026	0.7117

Bruker Compass DataAnalysis 4.0 printed: 5 12/2/2022 10:52:12 AM Page1 of 1

Compound **24** (HRMS)

Mass Spectrum SmartFormula Report

Analysis Info

Analysis Name	D:\Data\TJ&YF-20220922\CHQ-26_37_01_4596.d	Acquisition Date	9/22/2022 2:27:32 PM
Method	new_tune_wide_3min_pos.m	Operator and analyser	ECNU.CHEM. G.D.Yang
Sample Name	CHQ-26	Instrument / Ser#	micrOTOF 10293
Comment			

Acquisition Parameter

Source Type	ESI	Ion Polarity	Positive	Set Nebulizer	1.5 Bar
Focus	Active			Set Dry Heater	180 °C
Scan Begin	100 m/z	Set Capillary	4500 V	Set Dry Gas	6.0 l/min
Scan End	3000 m/z	Set End Plate Offset	-500 V	Set Divert Valve	Waste

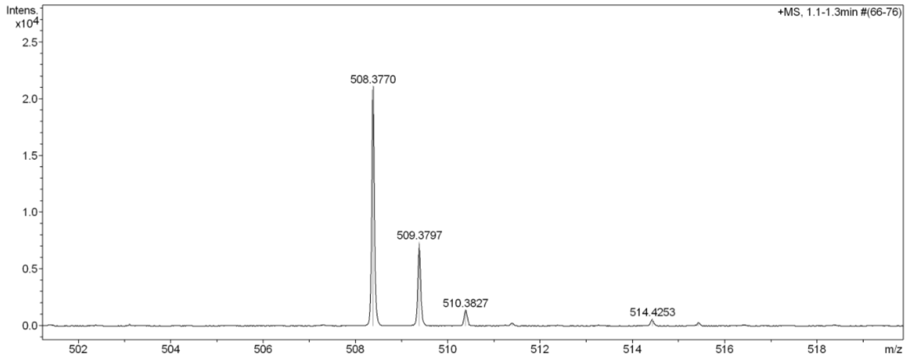

Meas. m/z	#	Formula	m/z	err [ppm]	Mean err [ppm]	rdb	N-Rule	e¯ Conf	mSigma	Std I	Std Mean m/z	Std I VarNorm	Std m/z Diff	Std Comb Dev
508.3770														
	1	C 31 H 51 N Na O 3	508.3761	-1.7	-1.3	6.5	ok	even	3.11	0.0059	0.0007	0.0023	0.0007	0.4196

Bruker Compass DataAnalysis 4.0 printed: 5 9/28/2022 3:09:39 PM Page1 of 1

Compound **25** (HRMS)

Mass Spectrum SmartFormula Report

Analysis Info

Analysis Name	D:\Data\tj&yf-20221118\CHQ-25_37_01_4665.d	Acquisition Date	11/18/2022 12:14:07 PM
Method	new_tune_wide_3min_pos.m	Operator and analyser	ECNU.CHEM. G.D.Yang
Sample Name	CHQ-25	Instrument / Ser#	micrOTOF 10293
Comment			

Acquisition Parameter

Source Type	ESI	Ion Polarity	Positive	Set Nebulizer	1.5 Bar
Focus	Active			Set Dry Heater	180 °C
Scan Begin	100 m/z	Set Capillary	4500 V	Set Dry Gas	6.0 l/min
Scan End	3000 m/z	Set End Plate Offset	-500 V	Set Divert Valve	Waste

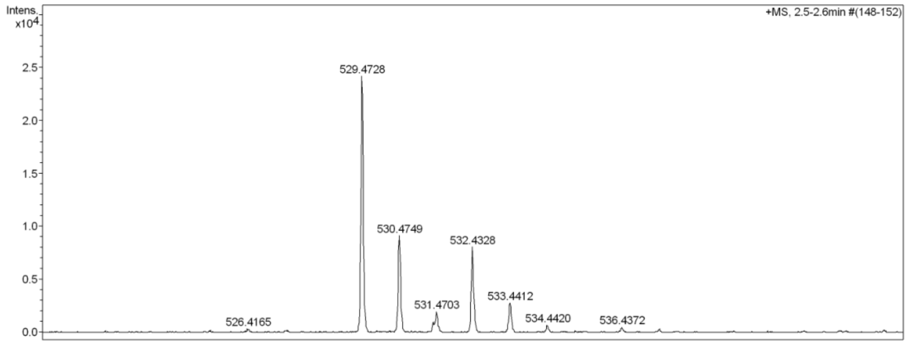

Meas. m/z	#	Formula	m/z	err [ppm]	Mean err [ppm]	rdb	N-Rule	e¯ Conf	mSigma	Std I	Std Mean m/z	Std I VarNorm	Std m/z Diff	Std Comb Dev
529.4728														
	1	C 34 H 61 N 2 O 2	529.4728	-0.1	1.5	5.5	ok	even	6.41	0.0089	0.0022	0.0057	0.0058	0.6398

Bruker Compass DataAnalysis 4.0 printed: 5 11/18/2022 2:57:53 PM Page1 of 1

Compound **5am** (HRMS)

Mass Spectrum SmartFormula Report

Analysis Info

Analysis Name	D:\Data\TJ&YF-20221202\5am_12_01_4707.d	Acquisition Date	12/2/2022 10:20:51 AM
Method	new_tune_wide_3min_pos.m	Operator and analyser	ECNU.CHEM. G.D.Yang
Sample Name	5am	Instrument / Ser#	micrOTOF 10293
Comment			

Acquisition Parameter

Source Type	ESI	Ion Polarity	Positive	Set Nebulizer	1.5 Bar
Focus	Active			Set Dry Heater	180 °C
Scan Begin	100 m/z	Set Capillary	4500 V	Set Dry Gas	6.0 l/min
Scan End	3000 m/z	Set End Plate Offset	-500 V	Set Divert Valve	Waste

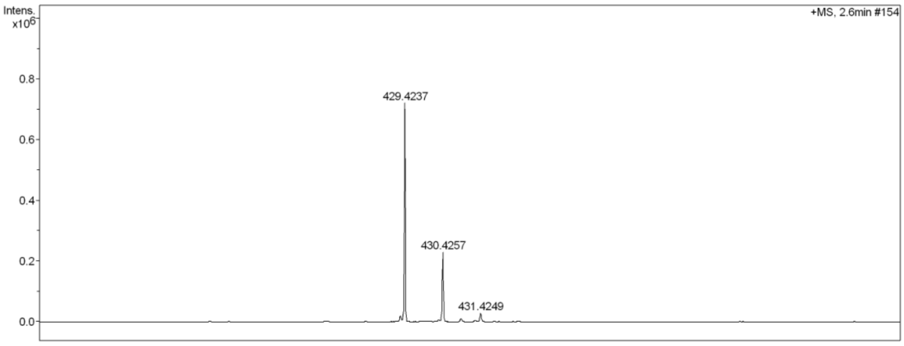

Meas. m/z	#	Formula	m/z	err [ppm]	Mean err [ppm]	rdb	N-Rule	e⁻ Conf	mSigma	Std I	Std Mean m/z	Std I VarNorm	Std m/z Diff	Std Comb Dev
429.4237														
	1	C 29 H 53 N 2	429.4203	-7.8	-6.7	4.5	ok	even	7.45	0.0146	0.0031	0.0056	0.0032	0.7065

Bruker Compass DataAnalysis 4.0 printed: 5 12/2/2022 10:55:46 AM Page1 of 1

Compound **26a** (HRMS)

Mass Spectrum SmartFormula Report

Analysis Info

Analysis Name	D:\Data\TJ&YF-20220922\CHQ-27a_35_01_4594.d	Acquisition Date	9/22/2022 2:18:15 PM
Method	new_tune_wide_3min_pos.m	Operator and analyser	ECNU.CHEM. G.D.Yang
Sample Name	CHQ-27a	Instrument / Ser#	micrOTOF 10293
Comment			

Acquisition Parameter

Source Type	ESI	Ion Polarity	Positive	Set Nebulizer	1.5 Bar
Focus	Active			Set Dry Heater	180 °C
Scan Begin	100 m/z	Set Capillary	4500 V	Set Dry Gas	6.0 l/min
Scan End	3000 m/z	Set End Plate Offset	-500 V	Set Divert Valve	Waste

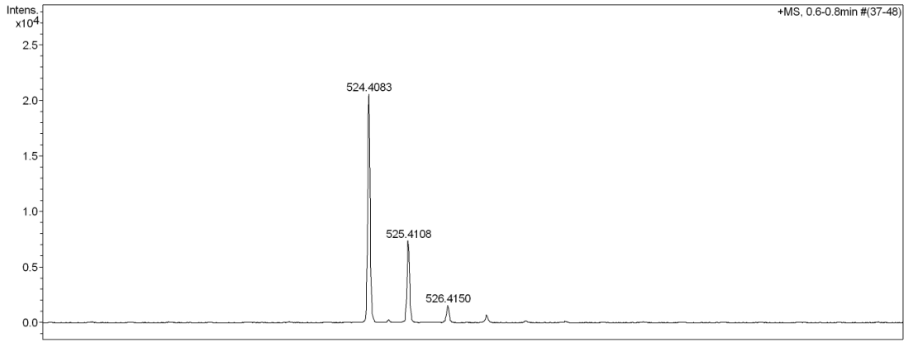

Meas. m/z	#	Formula	m/z	err [ppm]	Mean err [ppm]	rdb	N-Rule	e⁻ Conf	mSigma	Std I	Std Mean m/z	Std I VarNorm	Std m/z Diff	Std Comb Dev
524.4083														
	1	C 32 H 55 N Na O 3	524.4074	-1.7	-0.5	5.5	ok	even	14.07	0.0265	0.0028	0.0141	0.0008	0.7585

Bruker Compass DataAnalysis 4.0 printed: 5 9/28/2022 3:11:56 PM Page1 of 1

Compound **26b** (HRMS)

Mass Spectrum SmartFormula Report

Analysis Info

Analysis Name	D:\Data\TJ&YF-20220922\CHQ-27b_39_01_4599.d	Acquisition Date	9/22/2022 2:41:35 PM
Method	new_tune_wide_3min_pos.m	Operator and analyser	ECNU.CHEM. G.D.Yang
Sample Name	CHQ-27b	Instrument / Ser#	micrOTOF 10293
Comment			

Acquisition Parameter

Source Type	ESI	Ion Polarity	Positive	Set Nebulizer	1.5 Bar
Focus	Active			Set Dry Heater	180 °C
Scan Begin	100 m/z	Set Capillary	4500 V	Set Dry Gas	6.0 l/min
Scan End	3000 m/z	Set End Plate Offset	-500 V	Set Divert Valve	Waste

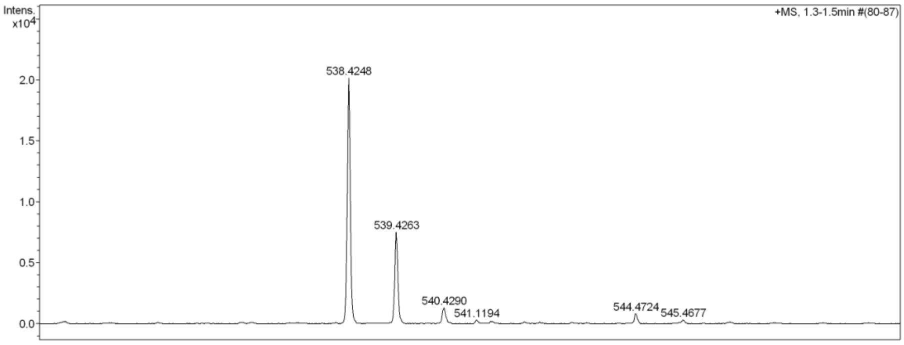

Meas. m/z	#	Formula	m/z	err [ppm]	Mean err [ppm]	rdb	N-Rule	e⁻ Conf	mSigma	Std I	Std Mean m/z	Std I VarNorm	Std m/z Diff	Std Comb Dev
538.4248														
	1	C 33 H 57 N Na O 3	538.4231	-3.3	-2.1	5.5	ok	even	4.36	0.0058	0.0015	0.0024	0.0019	0.5028

Bruker Compass DataAnalysis 4.0 printed: 5 9/28/2022 3:14:18 PM Page1 of 1

Compound **26c** (HRMS)

Mass Spectrum SmartFormula Report

Analysis Info

Analysis Name	D:\Data\TJ&YF-20220922\CHQ-27c_36_01_4595.d	Acquisition Date	9/22/2022 2:22:53 PM
Method	new_tune_wide_3min_pos.m	Operator and analyser	ECNU.CHEM. G.D.Yang
Sample Name	CHQ-27c	Instrument / Ser#	micrOTOF 10293
Comment			

Acquisition Parameter

Source Type	ESI	Ion Polarity	Positive	Set Nebulizer	1.5 Bar
Focus	Active			Set Dry Heater	180 °C
Scan Begin	100 m/z	Set Capillary	4500 V	Set Dry Gas	6.0 l/min
Scan End	3000 m/z	Set End Plate Offset	-500 V	Set Divert Valve	Waste

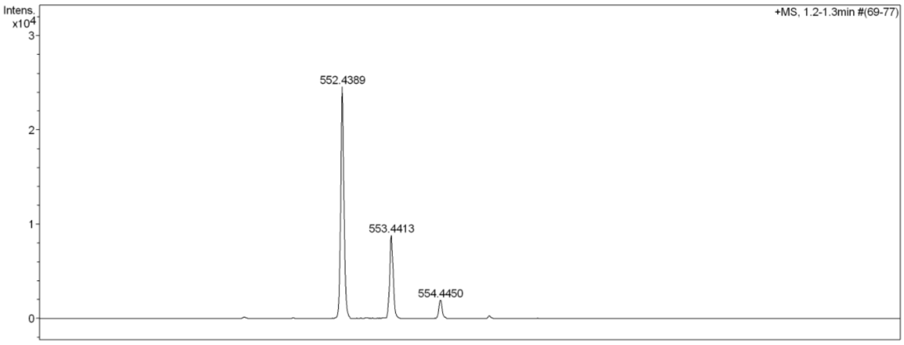

Meas. m/z	#	Formula	m/z	err [ppm]	Mean err [ppm]	rdb	N-Rule	e⁻ Conf	mSigma	Std I	Std Mean m/z	Std I VarNorm	Std m/z Diff	Std Comb Dev
552.4389														
	1	C 34 H 59 N Na O 3	552.4387	-0.3	0.2	5.5	ok	even	9.75	0.0128	0.0004	0.0047	0.0008	0.5017

Bruker Compass DataAnalysis 4.0 printed: 5 9/28/2022 3:16:11 PM Page1 of 1

Compound **26e** (HRMS)

Mass Spectrum SmartFormula Report

Analysis Info

Analysis Name	D:\Data\TJ&YF-20220922\CHQ-27d_41_01_4601.d	Acquisition Date	9/22/2022 2:50:51 PM
Method	new_tune_wide_3min_pos.m	Operator and analyser	ECNU.CHEM. G.D.Yang
Sample Name	CHQ-27d	Instrument / Ser#	micrOTOF 10293
Comment			

Acquisition Parameter

Source Type	ESI	Ion Polarity	Positive	Set Nebulizer	1.5 Bar
Focus	Active			Set Dry Heater	180 °C
Scan Begin	100 m/z	Set Capillary	4500 V	Set Dry Gas	6.0 l/min
Scan End	3000 m/z	Set End Plate Offset	-500 V	Set Divert Valve	Waste

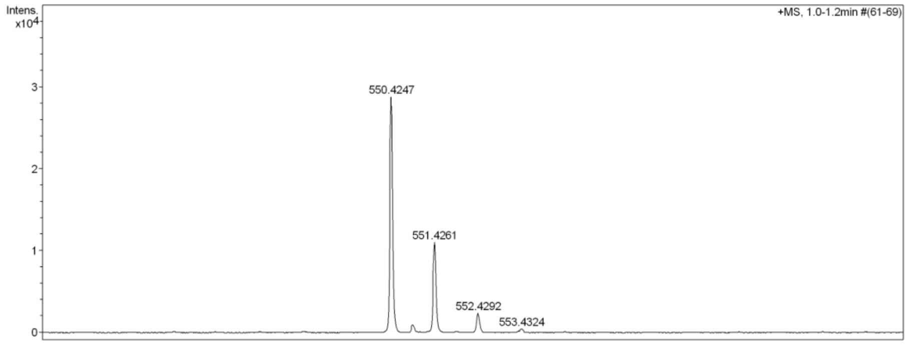

Meas. m/z	#	Formula	m/z	err [ppm]	Mean err [ppm]	rdb	N-Rule	e⁻ Conf	mSigma	Std I	Std Mean m/z	Std I VarNorm	Std m/z Diff	Std Comb Dev
550.4247														
	1	C 34 H 57 N Na O 3	550.4231	-3.0	-1.8	6.5	ok	even	5.76	0.0108	0.0014	0.0043	0.0019	0.5449

Bruker Compass DataAnalysis 4.0 printed: 5 9/28/2022 3:18:13 PM Page1 of 1

Compound **5an** (HRMS)

Mass Spectrum SmartFormula Report

Analysis Info

Analysis Name	D:\Data\TJ&YF-20221028\CHQ-3ao_13_01_4620.d	Acquisition Date	10/28/2022 9:57:50 AM
Method	new_tune_wide_3min_pos.m	Operator and analyser	ECNU.CHEM. G.D.Yang
Sample Name	CHQ-3ao	Instrument / Ser#	micrOTOF 10293
Comment			

Acquisition Parameter

Source Type	ESI	Ion Polarity	Positive	Set Nebulizer	1.5 Bar
Focus	Active			Set Dry Heater	180 °C
Scan Begin	100 m/z	Set Capillary	4500 V	Set Dry Gas	6.0 l/min
Scan End	3000 m/z	Set End Plate Offset	-500 V	Set Divert Valve	Waste

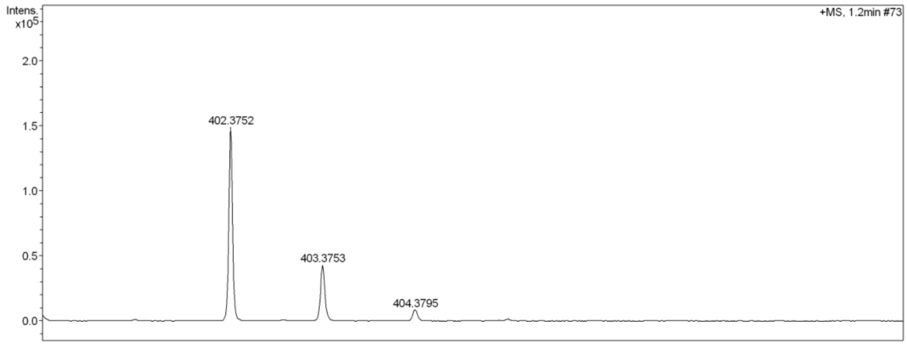

Meas. m/z	#	Formula	m/z	err [ppm]	Mean err [ppm]	rdb	N-Rule	e⁻ Conf	mSigma	Std I	Std Mean m/z	Std I VarNorm	Std m/z Diff	Std Comb Dev
402.3752														
	1	C 27 H 48 N O	402.3730	-5.5	-3.5	4.5	ok	even	9.16	0.0137	0.0020	0.0067	0.0028	0.6084

Bruker Compass DataAnalysis 4.0 printed: 5 10/28/2022 12:45:21 PM Page1 of 1

Compound **5ao** (HRMS)

Mass Spectrum SmartFormula Report

**Analysis Info**			
Analysis Name	D:\Data\TJ&YF-20221028\CHQ-8ap_17_01_4624.d	Acquisition Date	10/28/2022 10:16:20 AM
Method	new_tune_wide_3min_pos.m	Operator and analyser	ECNU.CHEM. G.D.Yang
Sample Name	CHQ-8ap	Instrument / Ser#	micrOTOF 10293
Comment			

**Acquisition Parameter**					
Source Type	ESI	Ion Polarity	Positive	Set Nebulizer	1.5 Bar
Focus	Active			Set Dry Heater	180 °C
Scan Begin	100 m/z	Set Capillary	4500 V	Set Dry Gas	6.0 l/min
Scan End	3000 m/z	Set End Plate Offset	-500 V	Set Divert Valve	Waste

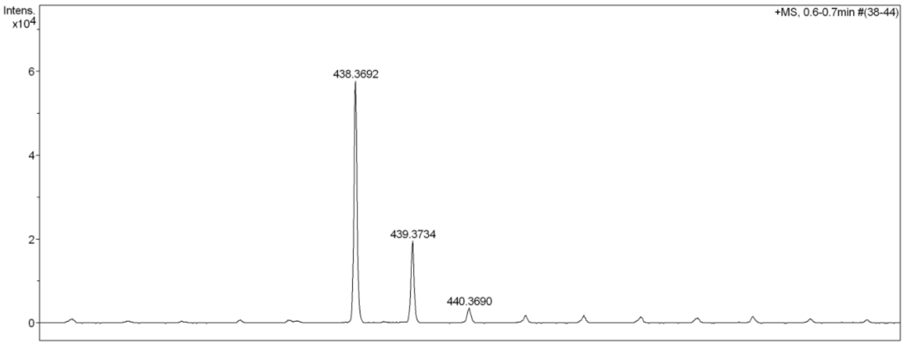

Meas. m/z	#	Formula	m/z	err [ppm]	Mean err [ppm]	rdb	N-Rule	e⁻ Conf	mSigma	Std I	Std Mean m/z	Std I VarNorm	Std m/z Diff	Std Comb Dev
438.3692														
	1	C 28 H 49 N Na O	438.3706	3.4	3.6	4.5	ok	even	16.34	0.0286	0.0022	0.0099	0.0046	0.6033

Bruker Compass DataAnalysis 4.0 printed: 5 10/28/2022 12:48:07 PM Page1 of 1

Compound **5ap** (HRMS)

Mass Spectrum SmartFormula Report

**Analysis Info**			
Analysis Name	D:\Data\TJ&YF-20221028\CHQ-8aq_15_01_4622.d	Acquisition Date	10/28/2022 10:07:04 AM
Method	new_tune_wide_3min_pos.m	Operator and analyser	ECNU.CHEM. G.D.Yang
Sample Name	CHQ-8aq	Instrument / Ser#	micrOTOF 10293
Comment			

**Acquisition Parameter**					
Source Type	ESI	Ion Polarity	Positive	Set Nebulizer	1.5 Bar
Focus	Active			Set Dry Heater	180 °C
Scan Begin	100 m/z	Set Capillary	4500 V	Set Dry Gas	6.0 l/min
Scan End	3000 m/z	Set End Plate Offset	-500 V	Set Divert Valve	Waste

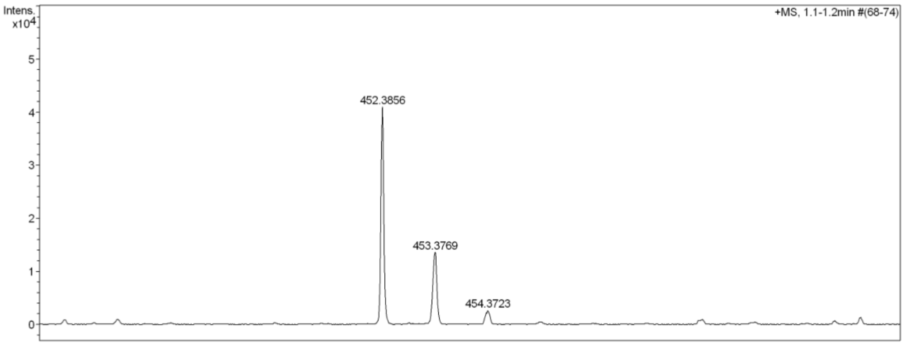

Meas. m/z	#	Formula	m/z	err [ppm]	Mean err [ppm]	rdb	N-Rule	e⁻ Conf	mSigma	Std I	Std Mean m/z	Std I VarNorm	Std m/z Diff	Std Comb Dev
452.3856														
	1	C 29 H 51 N Na O	452.3863	1.5	9.9	4.5	ok	even	8.90	0.0163	0.0077	0.0070	0.0141	0.7196

Bruker Compass DataAnalysis 4.0 printed: 5 10/28/2022 12:50:13 PM Page1 of 1

Compound **5aq** (HRMS)

Mass Spectrum SmartFormula Report

Analysis Info

Analysis Name	D:\Data\TJ&YF-20221028\CHQ-8as_20_01_4627.d	Acquisition Date	10/28/2022 10:30:10 AM
Method	new_tune_wide_3min_pos.m	Operator and analyser	ECNU.CHEM. G.D.Yang
Sample Name	CHQ-8as	Instrument / Ser#	micrOTOF 10293
Comment			

Acquisition Parameter

Source Type	ESI	Ion Polarity	Positive	Set Nebulizer	1.5 Bar
Focus	Active			Set Dry Heater	180 °C
Scan Begin	100 m/z	Set Capillary	4500 V	Set Dry Gas	6.0 l/min
Scan End	3000 m/z	Set End Plate Offset	-500 V	Set Divert Valve	Waste

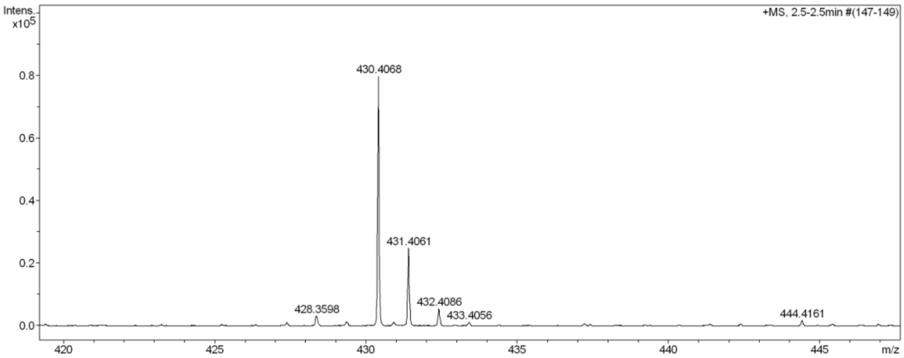

Meas. m/z	#	Formula	m/z	err [ppm]	Mean err [ppm]	rdb	N-Rule	e⁻ Conf	mSigma	Std I	Std Mean m/z	Std I VarNorm	Std m/z Diff	Std Comb Dev
430.4068														
	1	C 29 H 52 N O	430.4043	-5.6	-2.7	4.5	ok	even	11.93	0.0196	0.0024	0.0095	0.0040	0.6613

Bruker Compass DataAnalysis 4.0 printed: 5 10/31/2022 3:39:32 PM Page1 of 1

Compound **5ar** (HRMS)

Mass Spectrum SmartFormula Report

Analysis Info

Analysis Name	D:\Data\TJ&YF-20221028\CHQ-8ar_18_01_4625.d	Acquisition Date	10/28/2022 10:20:56 AM
Method	new_tune_wide_3min_pos.m	Operator and analyser	ECNU.CHEM. G.D.Yang
Sample Name	CHQ-8ar	Instrument / Ser#	micrOTOF 10293
Comment			

Acquisition Parameter

Source Type	ESI	Ion Polarity	Positive	Set Nebulizer	1.5 Bar
Focus	Active			Set Dry Heater	180 °C
Scan Begin	100 m/z	Set Capillary	4500 V	Set Dry Gas	6.0 l/min
Scan End	3000 m/z	Set End Plate Offset	-500 V	Set Divert Valve	Waste

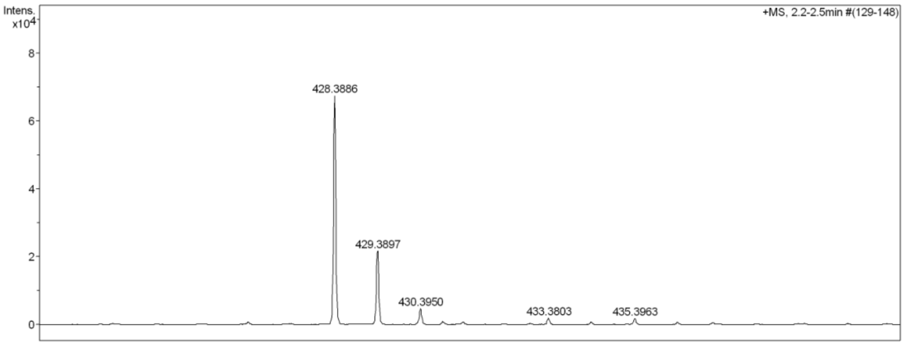

Meas. m/z	#	Formula	m/z	err [ppm]	Mean err [ppm]	rdb	N-Rule	e⁻ Conf	mSigma	Std I	Std Mean m/z	Std I VarNorm	Std m/z Diff	Std Comb Dev
428.3886														
	1	C 29 H 50 N O	428.3887	0.2	1.6	5.5	ok	even	10.39	0.0199	0.0013	0.0091	0.0018	0.5681

Bruker Compass DataAnalysis 4.0 printed: 5 10/28/2022 12:52:17 PM Page1 of 1

Compound **27a** (HRMS)

Mass Spectrum SmartFormula Report

Analysis Info

Analysis Name	D:\Data\TJ&YF-20221028\CHQ-24a_14_01_4621.d	Acquisition Date	10/28/2022 10:02:27 AM
Method	new_tune_wide_3min_pos.m	Operator and analyser	ECNU.CHEM. G.D.Yang
Sample Name	CHQ-24a	Instrument / Ser#	micrOTOF 10293
Comment			

Acquisition Parameter

Source Type	ESI	Ion Polarity	Positive	Set Nebulizer	1.5 Bar
Focus	Active			Set Dry Heater	180 °C
Scan Begin	100 m/z	Set Capillary	4500 V	Set Dry Gas	6.0 l/min
Scan End	3000 m/z	Set End Plate Offset	-500 V	Set Divert Valve	Waste

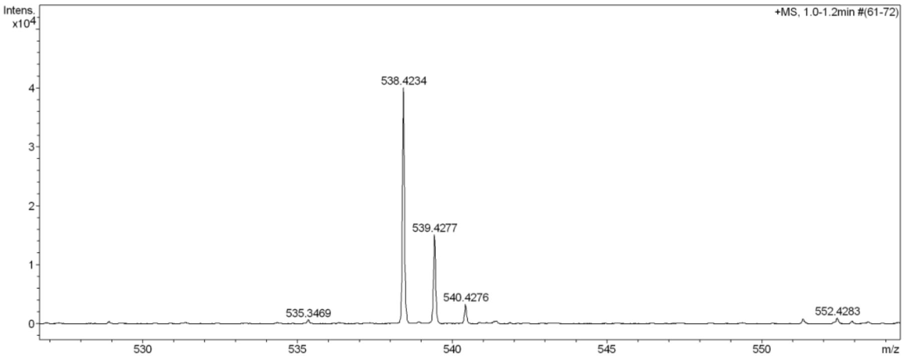

Meas. m/z	#	Formula	m/z	err [ppm]	Mean err [ppm]	rdb	N-Rule	e⁻ Conf	mSigma	Std I	Std Mean m/z	Std I VarNorm	Std m/z Diff	Std Comb Dev
538.4234	1	C 33 H 57 N Na O 3	538.4231	-0.6	-0.5	5.5	ok	even	8.51	0.0149	0.0019	0.0055	0.0021	0.5981

Bruker Compass DataAnalysis 4.0 printed: 5 10/28/2022 10:57:51 AM Page1 of 1

Compound **27b** (HRMS)

Mass Spectrum SmartFormula Report

Analysis Info

Analysis Name	D:\Data\tj&yf-20220928\CHQ 24b_48_01_4609.d	Acquisition Date	9/28/2022 2:39:12 PM
Method	new_tune_wide_3min_pos.m	Operator and analyser	ECNU.CHEM. G.D.Yang
Sample Name	CHQ 24b	Instrument / Ser#	micrOTOF 10293
Comment			

Acquisition Parameter

Source Type	ESI	Ion Polarity	Positive	Set Nebulizer	1.5 Bar
Focus	Active			Set Dry Heater	180 °C
Scan Begin	100 m/z	Set Capillary	4500 V	Set Dry Gas	6.0 l/min
Scan End	3000 m/z	Set End Plate Offset	-500 V	Set Divert Valve	Waste

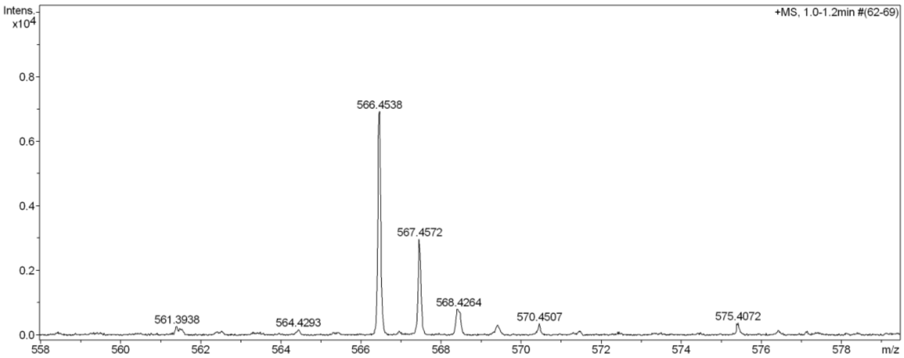

Meas. m/z	#	Formula	m/z	err [ppm]	Mean err [ppm]	rdb	N-Rule	e⁻ Conf	mSigma	Std I	Std Mean m/z	Std I VarNorm	Std m/z Diff	Std Comb Dev
566.4538	1	C 35 H 61 N Na O 3	566.4544	1.1	8.1	5.5	ok	even	31.85	0.0549	0.0131	0.0222	0.0231	0.8232

Bruker Compass DataAnalysis 4.0 printed: 5 9/28/2022 2:52:00 PM Page1 of 1

Compound **27c** (HRMS)

Mass Spectrum SmartFormula Report

Analysis Info

Analysis Name	D:\Data\tj&yf-20221118\CHQ-27c_39_01_4676.d	Acquisition Date	11/18/2022 3:19:22 PM
Method	new_tune_wide_3min_pos.m	Operator and analyser	ECNU.CHEM. G.D.Yang
Sample Name	CHQ-27c	Instrument / Ser#	micrOTOF 10293
Comment			

Acquisition Parameter

Source Type	ESI	Ion Polarity	Positive	Set Nebulizer	1.5 Bar
Focus	Active			Set Dry Heater	180 °C
Scan Begin	100 m/z	Set Capillary	4500 V	Set Dry Gas	6.0 l/min
Scan End	3000 m/z	Set End Plate Offset	-500 V	Set Divert Valve	Waste

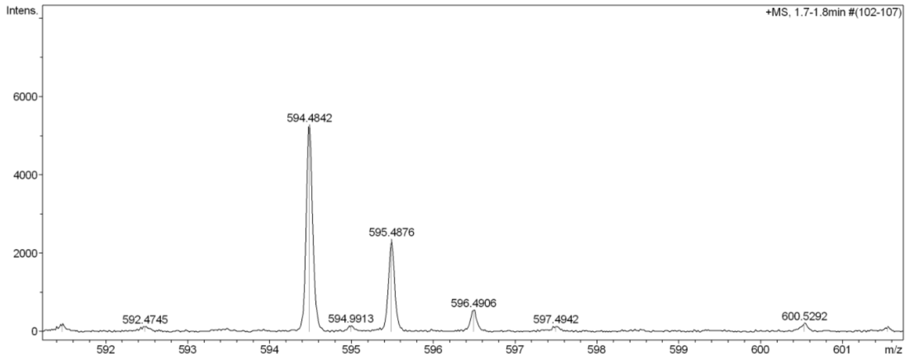

Meas. m/z	#	Formula	m/z	err [ppm]	Mean err [ppm]	rdb	N-Rule	e¯ Conf	mSigma	Std I	Std Mean m/z	Std I VarNorm	Std m/z Diff	Std Comb Dev
594.4842														
	1	C 37 H 65 N Na O 3	594.4857	2.4	2.5	5.5	ok	even	21.12	0.0321	0.0015	0.0112	0.0002	0.6230

Bruker Compass DataAnalysis 4.0 printed: 5 11/18/2022 3:46:50 PM Page1 of 1

Compound **5as** (HRMS)

Mass Spectrum SmartFormula Report

Analysis Info

Analysis Name	D:\Data\TJ&YF-20221202\5as_11_01_4706.d	Acquisition Date	12/2/2022 10:16:21 AM
Method	new_tune_wide_3min_pos.m	Operator and analyser	ECNU.CHEM. G.D.Yang
Sample Name	5as	Instrument / Ser#	micrOTOF 10293
Comment			

Acquisition Parameter

Source Type	ESI	Ion Polarity	Positive	Set Nebulizer	1.5 Bar
Focus	Active			Set Dry Heater	180 °C
Scan Begin	100 m/z	Set Capillary	4500 V	Set Dry Gas	6.0 l/min
Scan End	3000 m/z	Set End Plate Offset	-500 V	Set Divert Valve	Waste

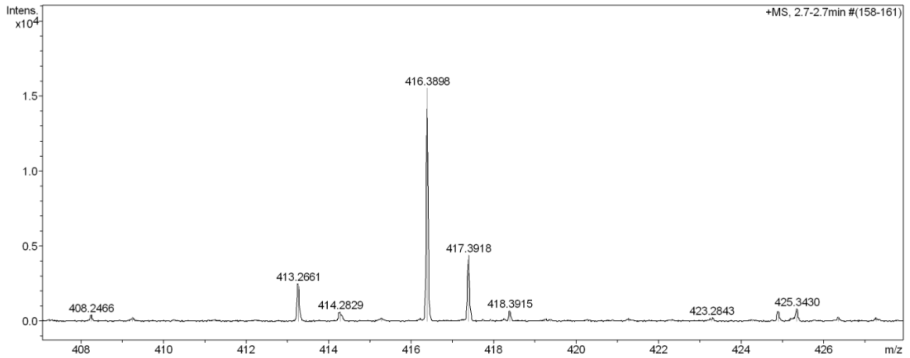

Meas. m/z	#	Formula	m/z	err [ppm]	Mean err [ppm]	rdb	N-Rule	e¯ Conf	mSigma	Std I	Std Mean m/z	Std I VarNorm	Std m/z Diff	Std Comb Dev
416.3898														
	1	C 28 H 50 N O	416.3887	-2.6	-1.2	4.5	ok	even	14.37	0.0219	0.0019	0.0073	0.0030	0.7065

Bruker Compass DataAnalysis 4.0 printed: 5 12/2/2022 10:23:15 AM Page1 of 1

Compound **5av** (HRMS)

Mass Spectrum SmartFormula Report

Analysis Info

Analysis Name	D:\Data\TJ&YF-20221202\5av_16_01_4711.d	Acquisition Date	12/2/2022 10:38:56 AM
Method	new_tune_wide_3min_pos.m	Operator and analyser	ECNU.CHEM. G.D.Yang
Sample Name	5av	Instrument / Ser#	micrOTOF 10293
Comment			

Acquisition Parameter

Source Type	ESI	Ion Polarity	Positive	Set Nebulizer	1.5 Bar
Focus	Active			Set Dry Heater	180 °C
Scan Begin	100 m/z	Set Capillary	4500 V	Set Dry Gas	6.0 l/min
Scan End	3000 m/z	Set End Plate Offset	-500 V	Set Divert Valve	Waste

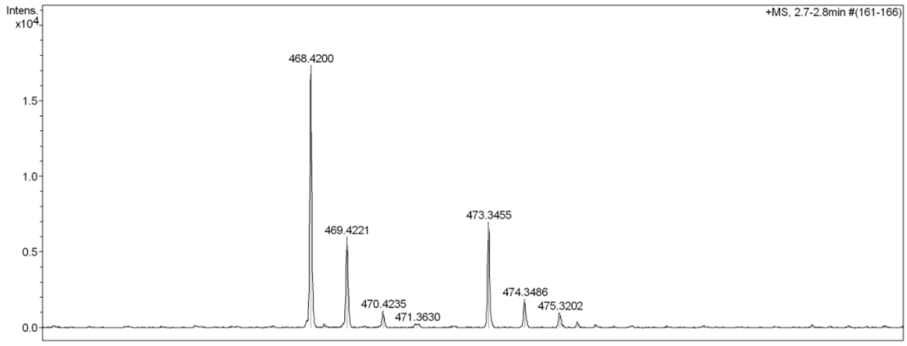

Meas. m/z	#	Formula	m/z	err [ppm]	Mean err [ppm]	rdb	N-Rule	e⁻ Conf	mSigma	Std I	Std Mean m/z	Std I VarNorm	Std m/z Diff	Std Comb Dev
468.4200														
	1	C 32 H 54 N O	468.4200	0.1	2.5	6.5	ok	even	6.43	0.0091	0.0068	0.0047	0.0020	0.7225

Bruker Compass DataAnalysis 4.0 printed: 5 12/2/2022 2:06:01 PM Page1 of 1

Compound **31** (HRMS)

Mass Spectrum SmartFormula Report

Analysis Info

Analysis Name	D:\Data\tj&yf-20221118\CHQ-31_41_01_4669.d	Acquisition Date	11/18/2022 12:32:40 PM
Method	new_tune_wide_3min_pos.m	Operator and analyser	ECNU.CHEM. G.D.Yang
Sample Name	CHQ-31	Instrument / Ser#	micrOTOF 10293
Comment			

Acquisition Parameter

Source Type	ESI	Ion Polarity	Positive	Set Nebulizer	1.5 Bar
Focus	Active			Set Dry Heater	180 °C
Scan Begin	100 m/z	Set Capillary	4500 V	Set Dry Gas	6.0 l/min
Scan End	3000 m/z	Set End Plate Offset	-500 V	Set Divert Valve	Waste

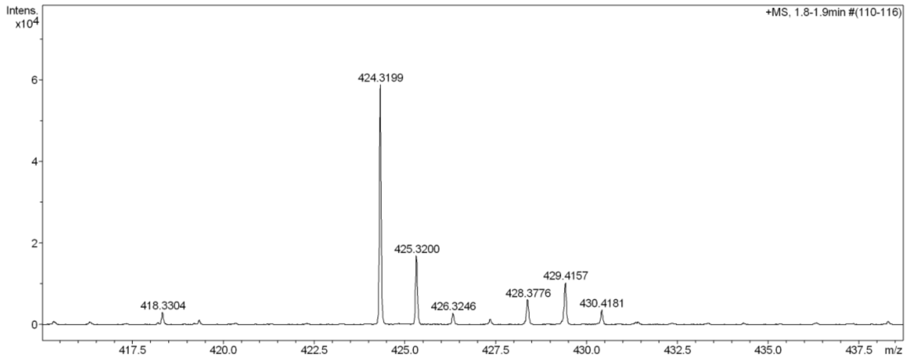

Meas. m/z	#	Formula	m/z	err [ppm]	Mean err [ppm]	rdb	N-Rule	e⁻ Conf	mSigma	Std I	Std Mean m/z	Std I VarNorm	Std m/z Diff	Std Comb Dev
424.3199														
	1	C 26 H 43 N Na O 2	424.3186	-3.0	-1.2	5.5	ok	even	2.60	0.0035	0.0015	0.0024	0.0033	0.4847

Bruker Compass DataAnalysis 4.0 printed: 5 11/18/2022 3:04:32 PM Page1 of 1

Compound **5aw** (HRMS)

Mass Spectrum SmartFormula Report

Analysis Info

Analysis Name	D:\Data\TJ&YF-20221202\5aw_15_01_4710.d	Acquisition Date	12/2/2022 10:34:26 AM
Method	new_tune_wide_3min_pos.m	Operator and analyser	ECNU.CHEM. G.D.Yang
Sample Name	5aw	Instrument / Ser#	micrOTOF 10293
Comment			

Acquisition Parameter

Source Type	ESI	Ion Polarity	Positive	Set Nebulizer	1.5 Bar
Focus	Active			Set Dry Heater	180 °C
Scan Begin	100 m/z	Set Capillary	4500 V	Set Dry Gas	6.0 l/min
Scan End	3000 m/z	Set End Plate Offset	-500 V	Set Divert Valve	Waste

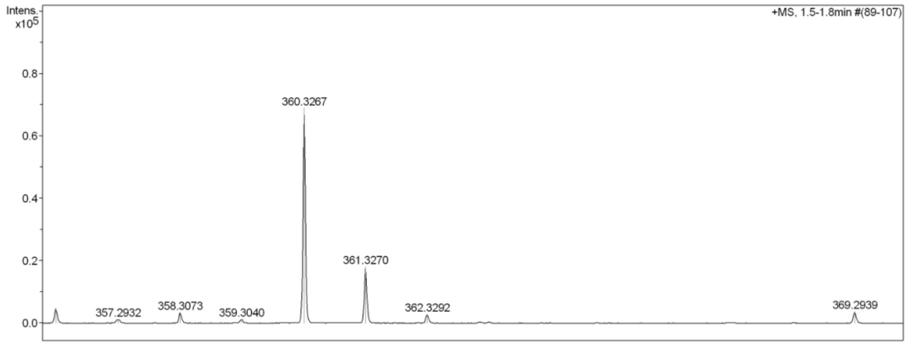

Meas. m/z	#	Formula	m/z	err [ppm]	Mean err [ppm]	rdb	N-Rule	e⁻ Conf	mSigma	Std I	Std Mean m/z	Std I VarNorm	Std m/z Diff	Std Comb Dev
360.3267														
	1	C 24 H 42 N O	360.3261	-1.7	0.4	4.5	ok	even	5.74	0.0077	0.0014	0.0052	0.0031	0.5443

Bruker Compass DataAnalysis 4.0 printed: 5 12/2/2022 2:08:03 PM Page1 of 1

Compound **34** (HRMS)

Mass Spectrum SmartFormula Report

Analysis Info

Analysis Name	D:\Data\tj&yf-20221205\34_29_01_4723.d	Acquisition Date	12/5/2022 3:20:22 PM
Method	new_tune_low_3min_pos.m	Operator and analyser	ECNU.CHEM. G.D.Yang
Sample Name	34	Instrument / Ser#	micrOTOF 10293
Comment			

Acquisition Parameter

Source Type	ESI	Ion Polarity	Positive	Set Nebulizer	1.5 Bar
Focus	Active			Set Dry Heater	180 °C
Scan Begin	50 m/z	Set Capillary	4500 V	Set Dry Gas	6.0 l/min
Scan End	3000 m/z	Set End Plate Offset	-500 V	Set Divert Valve	Waste

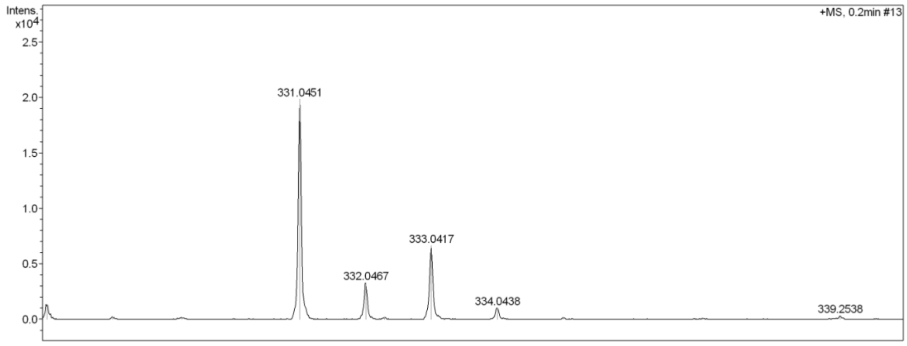

Meas. m/z	#	Formula	m/z	err [ppm]	Mean err [ppm]	rdb	N-Rule	e⁻ Conf	mSigma	Std I	Std Mean m/z	Std I VarNorm	Std m/z Diff	Std Comb Dev
331.0451														
	1	C 14 H 11 Cl F 3 N 2 O 2	331.0456	1.3	2.4	8.5	ok	even	3.32	0.0048	0.0010	0.0022	0.0012	0.4155

Bruker Compass DataAnalysis 4.0 printed: 5 12/5/2022 3:45:52 PM Page1 of 1

Compound **5ax** (HRMS)

Mass Spectrum SmartFormula Report

Analysis Info

Analysis Name	D:\Data\tj&yf-20221118\CHQ-5ax_44_01_4672.d	Acquisition Date	11/18/2022 12:46:30 PM
Method	new_tune_wide_3min_pos.m	Operator and analyser	ECNU.CHEM. G.D.Yang
Sample Name	CHQ-5ax	Instrument / Ser#	micrOTOF 10293
Comment			

Acquisition Parameter

Source Type	ESI	Ion Polarity	Positive	Set Nebulizer	1.5 Bar
Focus	Active			Set Dry Heater	180 °C
Scan Begin	100 m/z	Set Capillary	4500 V	Set Dry Gas	6.0 l/min
Scan End	3000 m/z	Set End Plate Offset	-500 V	Set Divert Valve	Waste

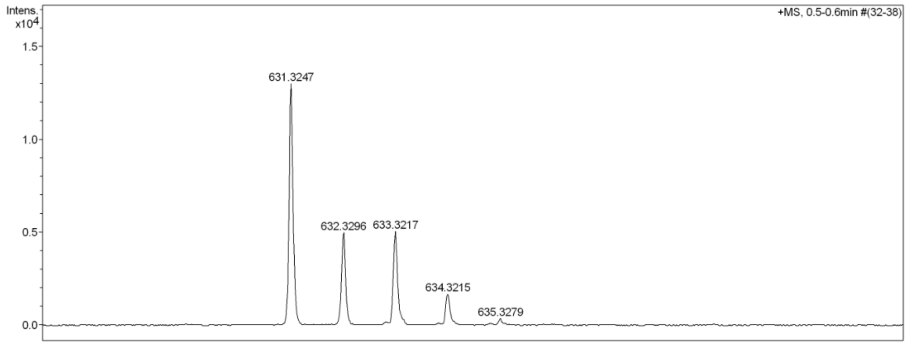

Meas. m/z	#	Formula	m/z	err [ppm]	Mean err [ppm]	rdb	N-Rule	e⁻ Conf	mSigma	Std I	Std Mean m/z	Std I VarNorm	Std m/z Diff	Std Comb Dev
631.3247														
	1	C 34 H 48 Cl F 3 N 2 Na O 2	631.3249	0.3	0.3	9.5	ok	even	3.21	0.0037	0.0012	0.0015	0.0023	0.4376

Bruker Compass DataAnalysis 4.0 printed: 5 11/18/2022 3:32:48 PM Page1 of 1

Compound **5ay** (HRMS)

Mass Spectrum SmartFormula Report

Analysis Info

Analysis Name	D:\Data\tj&yf-20221205\5ay_24_01_4718.d	Acquisition Date	12/5/2022 2:57:27 PM
Method	new_tune_wide_3min_pos.m	Operator and analyser	ECNU.CHEM. G.D.Yang
Sample Name	5ay	Instrument / Ser#	micrOTOF 10293
Comment			

Acquisition Parameter

Source Type	ESI	Ion Polarity	Positive	Set Nebulizer	1.5 Bar
Focus	Active			Set Dry Heater	180 °C
Scan Begin	100 m/z	Set Capillary	4500 V	Set Dry Gas	6.0 l/min
Scan End	3000 m/z	Set End Plate Offset	-500 V	Set Divert Valve	Waste

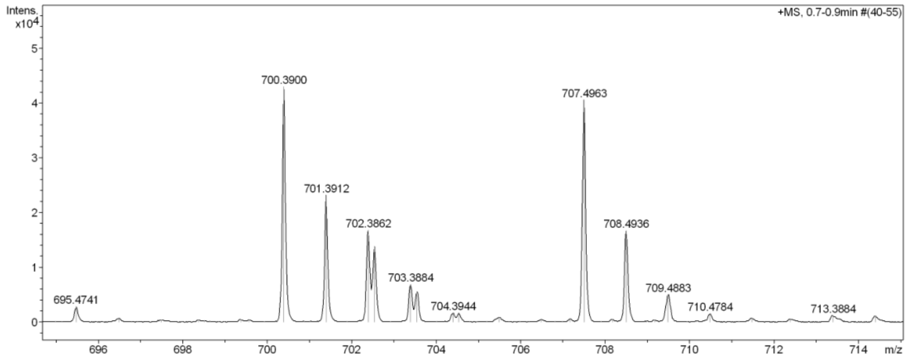

Meas. m/z	#	Formula	m/z	err [ppm]	Mean err [ppm]	rdb	N-Rule	e⁻ Conf	mSigma	Std I	Std Mean m/z	Std I VarNorm	Std m/z Diff	Std Comb Dev
700.3900														
	1	C 40 H 54 Cl F 3 N 3 O 2	700.3851	-7.0	-5.6	13.5	ok	even	38.54	0.0436	0.0041	0.0121	0.0015	0.7210

Bruker Compass DataAnalysis 4.0 printed: 5 12/5/2022 4:46:35 PM Page1 of 1

Compound **37** (HRMS)

Mass Spectrum SmartFormula Report

Analysis Info

Analysis Name	D:\Data\TJ&YF-20221028\CHQ-37_27_01_4634.d	Acquisition Date	10/28/2022 11:22:46 AM
Method	new_tune_wide_3min_pos.m	Operator and analyser	ECNU.CHEM. G.D.Yang
Sample Name	CHQ-37	Instrument / Ser#	micrOTOF 10293
Comment			

Acquisition Parameter

Source Type	ESI	Ion Polarity	Positive	Set Nebulizer	1.5 Bar
Focus	Active			Set Dry Heater	180 °C
Scan Begin	100 m/z	Set Capillary	4500 V	Set Dry Gas	6.0 l/min
Scan End	3000 m/z	Set End Plate Offset	-500 V	Set Divert Valve	Waste

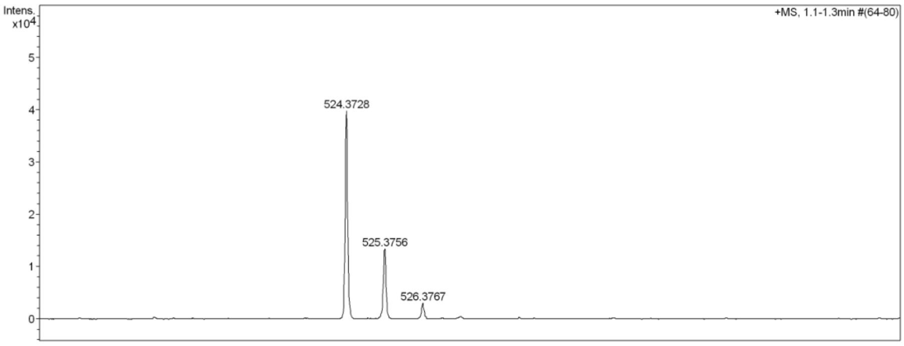

Meas. m/z	#	Formula	m/z	err [ppm]	Mean err [ppm]	rdb	N-Rule	e⁻ Conf	mSigma	Std I	Std Mean m/z	Std I VarNorm	Std m/z Diff	Std Comb Dev
524.3728														
	1	C 31 H 51 N Na O 4	524.3710	-3.4	-2.7	6.5	ok	even	6.93	0.0101	0.0019	0.0046	0.0018	0.5617

Bruker Compass DataAnalysis 4.0 printed: 5 10/31/2022 3:30:06 PM Page1 of 1

Compound **38** (HRMS)

Mass Spectrum SmartFormula Report

Analysis Info

Analysis Name	D:\Data\TJ&YF-20221028\CHQ-38_34_01_4642.d	Acquisition Date	10/28/2022 12:49:00 PM
Method	new_tune_wide_3min_pos.m	Operator and analyser	ECNU.CHEM. G.D.Yang
Sample Name	CHQ-38	Instrument / Ser#	micrOTOF 10293
Comment			

Acquisition Parameter

Source Type	ESI	Ion Polarity	Positive	Set Nebulizer	1.5 Bar
Focus	Active			Set Dry Heater	180 °C
Scan Begin	100 m/z	Set Capillary	4500 V	Set Dry Gas	6.0 l/min
Scan End	3000 m/z	Set End Plate Offset	-500 V	Set Divert Valve	Waste

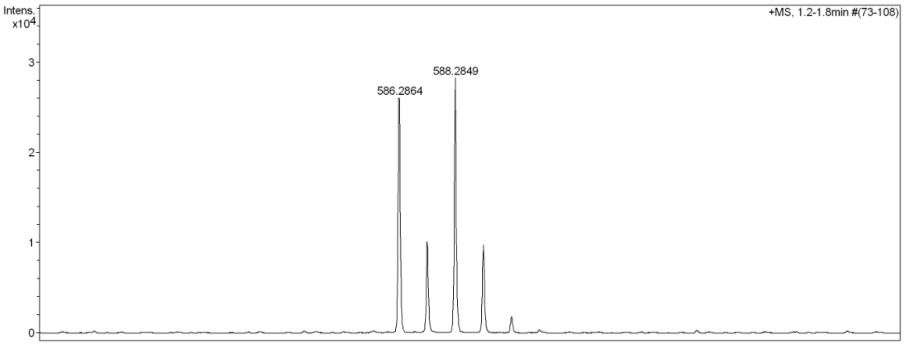

Meas. m/z	#	Formula	m/z	err [ppm]	Mean err [ppm]	rdb	N-Rule	e⁻ Conf	mSigma	Std I	Std Mean m/z	Std I VarNorm	Std m/z Diff	Std Comb Dev
586.2864														
	1	C 31 H 50 Br N Na O 3	586.2866	0.4	0.8	6.5	ok	even	21.07	0.0195	0.0010	0.0054	0.0016	0.5297

Bruker Compass DataAnalysis 4.0 printed: 5 10/31/2022 3:32:54 PM Page1 of 1

Compound **39** (HRMS)

Mass Spectrum SmartFormula Report

Analysis Info

Analysis Name	D:\Data\TJ&YF-20221028\CHQ-39_33_01_4641.d	Acquisition Date	10/28/2022 12:44:27 PM
Method	new_tune_wide_3min_pos.m	Operator and analyser	ECNU.CHEM. G.D.Yang
Sample Name	CHQ-39	Instrument / Ser#	micrOTOF 10293
Comment			

Acquisition Parameter

Source Type	ESI	Ion Polarity	Positive	Set Nebulizer	1.5 Bar
Focus	Active			Set Dry Heater	180 °C
Scan Begin	100 m/z	Set Capillary	4500 V	Set Dry Gas	6.0 l/min
Scan End	3000 m/z	Set End Plate Offset	-500 V	Set Divert Valve	Waste

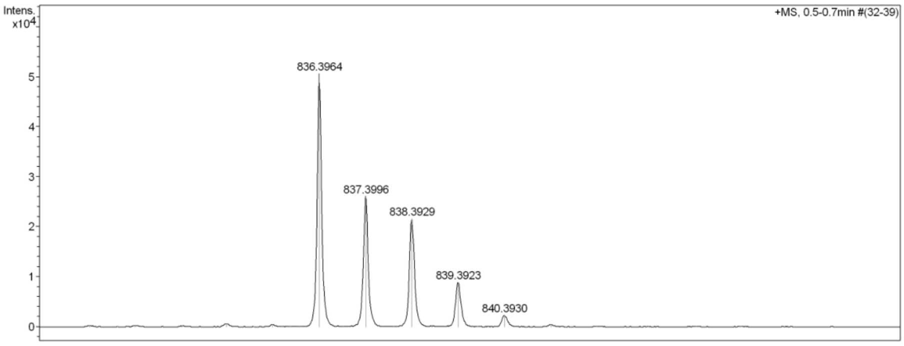

Meas. m/z	#	Formula	m/z	err [ppm]	Mean err [ppm]	rdb	N-Rule	e⁻ Conf	mSigma	Std I	Std Mean m/z	Std I VarNorm	Std m/z Diff	Std Comb Dev
836.3964														

Compound **40** (HRMS)

Mass Spectrum SmartFormula Report

Analysis Info

Analysis Name	D:\Data\tj&yf-20221118\CHQ-40_45_01_4673.d	Acquisition Date	11/18/2022 12:51:08 PM
Method	new_tune_wide_3min_pos.m	Operator and analyser	ECNU.CHEM. G.D.Yang
Sample Name	CHQ-40	Instrument / Ser#	micrOTOF 10293
Comment			

Acquisition Parameter

Source Type	ESI	Ion Polarity	Positive	Set Nebulizer	1.5 Bar
Focus	Active			Set Dry Heater	180 °C
Scan Begin	100 m/z	Set Capillary	4500 V	Set Dry Gas	6.0 l/min
Scan End	3000 m/z	Set End Plate Offset	-500 V	Set Divert Valve	Waste

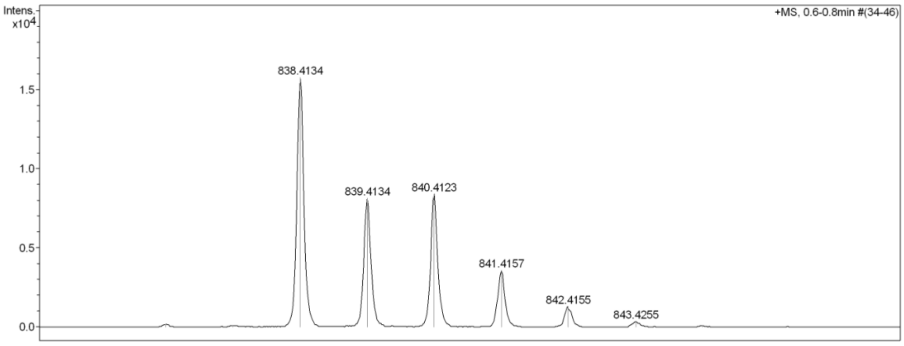

Meas. m/z	#	Formula	m/z	err [ppm]	Mean err [ppm]	rdb	N-Rule	e⁻ Conf	mSigma	Std I	Std Mean m/z	Std I VarNorm	Std m/z Diff	Std Comb Dev
838.4134														

Compound **5az** (HRMS)

Mass Spectrum SmartFormula Report

Analysis Info

Analysis Name	D:\Data\tj&yf-20221205\5az_25_01_4719.d	Acquisition Date	12/5/2022 3:01:58 PM
Method	new_tune_wide_3min_pos.m	Operator and analyser	ECNU.CHEM. G.D.Yang
Sample Name	5az	Instrument / Ser#	micrOTOF 10293
Comment			

Acquisition Parameter

Source Type	ESI	Ion Polarity	Positive	Set Nebulizer	1.5 Bar
Focus	Active			Set Dry Heater	180 °C
Scan Begin	100 m/z	Set Capillary	4500 V	Set Dry Gas	6.0 l/min
Scan End	3000 m/z	Set End Plate Offset	-500 V	Set Divert Valve	Waste

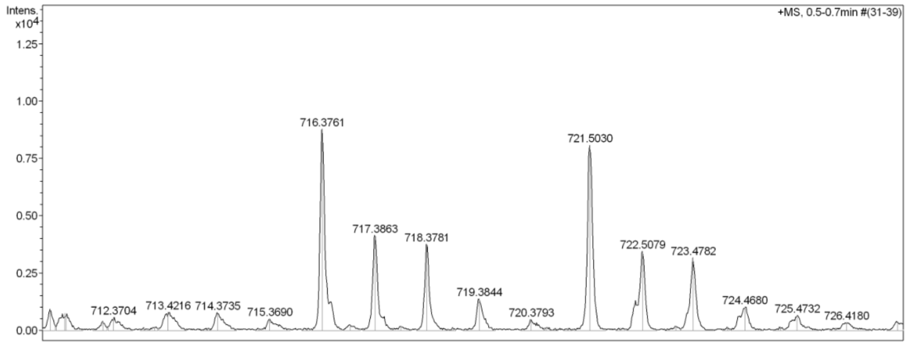

Meas. m/z	#	Formula	m/z	err [ppm]	Mean err [ppm]	rdb	N-Rule	e⁻ Conf	mSigma	Std I	Std Mean m/z	Std I VarNorm	Std m/z Diff	Std Comb Dev
716.3761														
	1	C 40 H 54 Cl F 3 N 3 O 3	716.3800	5.5	1.3	13.5	ok	even	12.96	0.0160	0.0033	0.0063	0.0049	0.6348

Bruker Compass DataAnalysis 4.0 printed: 5 12/5/2022 4:50:08 PM Page1 of 1

Compound **41** (HRMS)

Mass Spectrum SmartFormula Report

Analysis Info

Analysis Name	D:\Data\TJ&YF-20221028\CHQ-41_28_01_4635.d	Acquisition Date	10/28/2022 11:27:25 AM
Method	new_tune_wide_3min_pos.m	Operator and analyser	ECNU.CHEM. G.D.Yang
Sample Name	CHQ-41	Instrument / Ser#	micrOTOF 10293
Comment			

Acquisition Parameter

Source Type	ESI	Ion Polarity	Positive	Set Nebulizer	1.5 Bar
Focus	Active			Set Dry Heater	180 °C
Scan Begin	100 m/z	Set Capillary	4500 V	Set Dry Gas	6.0 l/min
Scan End	3000 m/z	Set End Plate Offset	-500 V	Set Divert Valve	Waste

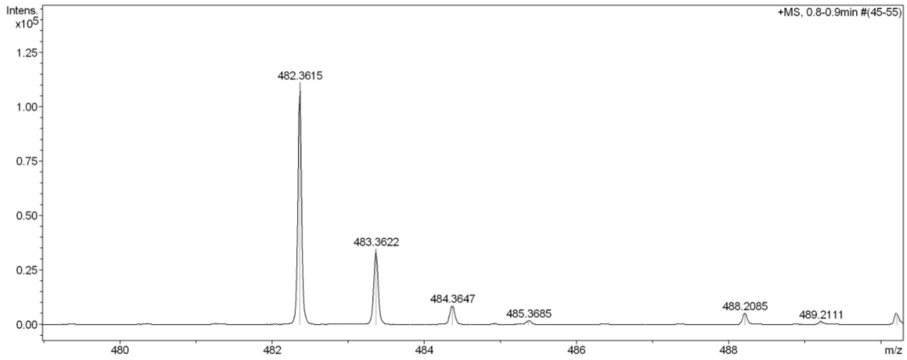

Meas. m/z	#	Formula	m/z	err [ppm]	Mean err [ppm]	rdb	N-Rule	e⁻ Conf	mSigma	Std I	Std Mean m/z	Std I VarNorm	Std m/z Diff	Std Comb Dev
482.3615														
	1	C 29 H 49 N Na O 3	482.3605	-2.2	-0.5	5.5	ok	even	12.90	0.0227	0.0013	0.0108	0.0028	0.6187

Bruker Compass DataAnalysis 4.0 printed: 5 10/31/2022 2:11:35 PM Page1 of 1

Compound **42** (HRMS)

Mass Spectrum SmartFormula Report

Analysis Info

Analysis Name	D:\Data\tj&yf-20221118\CHQ-42_46_01_4674.d	Acquisition Date	11/18/2022 12:55:48 PM
Method	new_tune_wide_3min_pos.m	Operator and analyser	ECNU.CHEM. G.D.Yang
Sample Name	CHQ-42	Instrument / Ser#	micrOTOF 10293
Comment			

Acquisition Parameter

Source Type	ESI	Ion Polarity	Positive	Set Nebulizer	1.5 Bar
Focus	Active			Set Dry Heater	180 °C
Scan Begin	100 m/z	Set Capillary	4500 V	Set Dry Gas	6.0 l/min
Scan End	3000 m/z	Set End Plate Offset	-500 V	Set Divert Valve	Waste

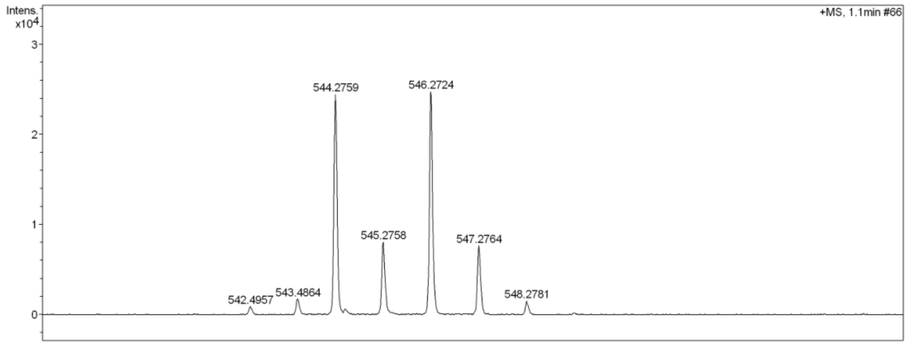

Meas. m/z	#	Formula	m/z	err [ppm]	Mean err [ppm]	rdb	N-Rule	e⁻ Conf	mSigma	Std I	Std Mean m/z	Std I VarNorm	Std m/z Diff	Std Comb Dev
544.2759														
	1	C 29 H 48 Br N Na O 2	544.2761	0.4	2.6	5.5	ok	even	8.85	0.0071	0.0019	0.0024	0.0021	0.5344

Bruker Compass DataAnalysis 4.0 printed: 5 11/18/2022 3:09:41 PM Page1 of 1

Compound **5ba** (HRMS)

Mass Spectrum SmartFormula Report

Analysis Info

Analysis Name	D:\Data\tj&yf-20220928\CHQ 8bd_46_01_4607.d	Acquisition Date	9/28/2022 2:29:39 PM
Method	new_tune_wide_3min_pos.m	Operator and analyser	ECNU.CHEM. G.D.Yang
Sample Name	CHQ 8bd	Instrument / Ser#	micrOTOF 10293
Comment			

Acquisition Parameter

Source Type	ESI	Ion Polarity	Positive	Set Nebulizer	1.5 Bar
Focus	Active			Set Dry Heater	180 °C
Scan Begin	100 m/z	Set Capillary	4500 V	Set Dry Gas	6.0 l/min
Scan End	3000 m/z	Set End Plate Offset	-500 V	Set Divert Valve	Waste

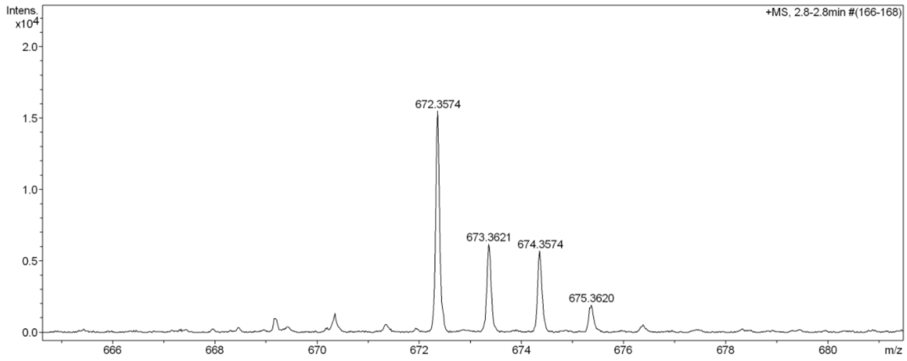

Meas. m/z	#	Formula	m/z	err [ppm]	Mean err [ppm]	rdb	N-Rule	e⁻ Conf	mSigma	Std I	Std Mean m/z	Std I VarNorm	Std m/z Diff	Std Comb Dev
672.3574														
	1	C 38 H 50 Cl F 3 N 3 O 2	672.3538	-5.3	-6.9	13.5	ok	even	30.07	0.0350	0.0048	0.0144	0.0020	0.8169

Bruker Compass DataAnalysis 4.0 printed: 5 9/28/2022 2:40:05 PM Page1 of 1
